# Gut Microbiota, Lipidome, and Metabolites Mediate Immune Dysregulation in Diabetic Microvascular Disease: A Two-sample Mendelian Randomization and Mediation Analysis

**DOI:** 10.2174/0118715303418611251125045911

**Published:** 2026-01-27

**Authors:** Jiaxing Zhao, Zenghui Liu, Lu Kuang, Huayu Yin, Heyue Gong, Yaolong Zheng, Shaoguo Wu, Dabin Liu, Shuyan Liu, Xuehui Liu, Limei Wu

**Affiliations:** 1 Department of Laboratory Medicine, The Affiliated Guangzhou Twelfth People's Hospital, Guangzhou Medical University, Guangzhou,Guangdong, China;; 2 Department of Immunology, Mudanjiang Medical University, Mudanjiang, Heilongjiang, China

**Keywords:** Immune dysregulation, gut microbiota, plasma lipidome, plasma metabolites, diabetic microvascular disease, Mendelian randomization, mediation analysis

## Abstract

**Introduction:**

Diabetic microvascular disease (DMiVD) involves dysregulated immune cell function, but the precise pathogenic mechanisms remain unclear.

**Materials and Methods:**

We conducted a two-sample Mendelian randomization (MR) study using comprehensive GWAS and FinnGen summary statistics, encompassing 731 immune cell phenotypes, 473 gut microbial taxa, 91 inflammatory proteins, 179 lipid types, 1,400 plasma metabolites, 20 micronutrients, and DMiVD cases. The analysis aimed to evaluate causal associations between these variables and DMiVD. We further explored potential mediating roles of gut microbiota, plasma lipidome, and metabolites using mediation analysis, with multiple sensitivity tests confirming the robustness of our findings.

**Results:**

We identified 20 immune cell phenotypes, 33 gut microbial taxa, 31 lipid types, and 83 plasma metabolites with significant causal associations with DMiVD. Mediation analysis revealed that the risk effect of CD3+ resting Tregs on diabetic nephropathy was partly mediated by phosphatidylcholine (16:0_18:2) (10.7%). Additionally, the protective effect of CX3CR1 on monocytes against DMiVD was partly mediated by Unclassified Bacilli A (35%), Species CAG-177 sp003538135 (22.6%), and triacylglycerol (52:6) (25.5%).

**Discussion:**

These findings advance understanding of DMiVD pathogenesis, highlighting that modulation of key metabolic pathways and immune regulatory nodes may represent promising therapeutic strategies. Further experimental studies are needed to validate these potential causal relationships.

**Conclusion:**

Using causal inference approaches, this study identifies immune cell-mediated mechanisms underlying DMiVD, involving gut microbiota, plasma lipids, and metabolites. The results suggest potential intervention targets for mechanistic studies and therapeutic development.

## 1 INTRODUCTION

Diabetic microvascular disease (DMiVD) is a systemic complication of diabetes characterized by endothelial dysfunction, basement membrane thickening, and capillary rarefaction, primarily affecting the retina, kidneys, and peripheral nerves, and potentially progressing to end-stage renal disease or irreversible blindness [1-3]. Affecting 30–40% of patients with diabetes, DMiVD imposes a substantial and growing burden within the global spectrum of non-communicable chronic diseases, causing significant morbidity, disability, and mortality [2, 4-7]. Diabetic kidney disease is the leading cause of end-stage renal disease worldwide, while diabetic retinopathy remains a major cause of acquired blindness [3, 5, 8]. Current management strategies often fail to halt disease progression, resulting in persistent morbidity despite treatment [2, 9]. These outcomes are partly due to an incomplete understanding of key drivers, particularly oxidative stress and unresolved immune and metabolic dysregulation [10-12].

Immune cells are essential components of the human immune system, orchestrating immune and metabolic crosstalk critical for tissue homeostasis [13, 14]. Their functions include pathogen recognition, cytotoxic elimination, and modulation of inflammatory responses [12, 14]. In DMiVD, dysregulated immune activity contributes directly to microvascular damage through inflammatory pathways and metabolic disturbances [3, 8, 10-12]. Macrophages, T cells, and complement activation drive renal inflammation in diabetic nephropathy, while similar immune and inflammatory mechanisms operate in retinopathy [3, 8, 10-12]. Regulatory T cells (Tregs) and mesenchymal stem cells (MSCs) attempt to counterbalance this dysregulation but are often insufficient [14]. Consequently, targeting immune and metabolic interactions, including inflammation, oxidative stress, and endothelial dysfunction, represents a promising therapeutic strategy [10, 15, 16]. However, mechanistic insights remain fragmented, with most studies focusing on isolated cell types rather than the integrated immune and metabolic axis [10, 14, 16].

Traditional observational studies are limited by confounding factors and reverse causation [17-19]. Mendelian randomization (MR) analysis addresses these limitations by using genetic variants as instrumental variables (IVs), enabling more robust causal inference [20-24]. In this study, we employed a comprehensive MR approach to investigate causal relationships between immune cells, gut microbiota, plasma lipidome, metabolites, micronutrients, and DMiVD risk. Mediation analyses were conducted to identify potential intermediate pathways, and reverse MR analyses were performed to confirm causal directions, thereby enhancing the robustness and validity of our findings.

## 2 MATERIALS AND METHODS

### 2.1 Study Design and Type of Study

This study employed a two-sample MR design, a genetic epidemiological approach that uses instrumental variable analysis to infer causal relationships. The analysis used publicly available genome-wide association study (GWAS) summary statistics and FinnGen statistics from European-ancestry populations. This study adheres to the Strengthening the Reporting of Observational Studies in Epidemiology using Mendelian Randomization (STROBE-MR) guidelines [25]. Fig. (**1**) illustrates the flowchart of this study.

### 2.2 Data Sources

GWAS summary data for DMiVD were obtained from the FinnGen project's large-scale European population cohort study. Specifically, we adopted the latest version of the R12 DMiVD dataset released on November 4, 2024. The dataset covers 3,862 cases of diabetic neuropathy and 92,612 control samples, 5,579 cases of diabetic nephropathy and 90,951 control samples, and 14,142 cases of diabetic retinopathy and 82,287 control samples providing abundant data support for the study [26]. For immune cell trait data, we accessed GWAS summary statistics from Orrù *et al.*, which included 731 immune cell traits derived from 3,757 European individuals [26]. These categories comprehensively covered multiple immune cell subpopulations, including B cells, conventional dendritic cells (cDCs), T cells, monocytes, Myeloid cells, TBNK cells (T cells, B cells, and natural killer cells), and Tregs. The dataset IDs ranged from GCST90001391 to GCST90002121. The GWAS data for gut microbiota were obtained from the EBI database (https://www.ebi.ac.uk/gwas/), based on analysis of fecal samples from 5,959 Finnish individuals [27]. This dataset provides the characteristics of each gut microbiota. A total of 473 taxa were included in the analysis, encompassing 10 phyla, 18 classes, 24 orders, 58 families, 143 genera, 213 species, and 7 unclassified taxa. Microbial taxa were annotated to the highest achievable taxonomic resolution using reference databases, notwithstanding limitations in metagenomic sequencing resolution. However, 21.3% (101/473) of taxa remained unclassified at species level due to limitations in metagenomic resolution. The registered IDs range from GCST90032172 to GCST90032644. Inflammatory protein data were obtained from Zhao *et al.*'s study of 14,824 individuals of European descent [28]. Using genome-wide protein quantitative trait locus analysis, the study systematically detected 91 inflammation-related plasma proteins. The dataset IDs ranged from GCST90274758 to GCST90274848. 179 Plasma lipidome data were derived from Ottensmann *et al.*'s study involving 7,174 European participants [29]. The dataset IDs ranged from GCST90277238 to GCST90277416. 1,400 Plasma metabolite data were sourced from Chen *et al.*'s study of 8,299 European individuals in the Canadian Longitudinal Study on Aging (CLSA) [30]. The study utilized the ultra-performance liquid chromatography-tandem mass spectrometry metabolon high-definition mass spectrometry (HD4) platform to quantitatively detect 1,091 plasma metabolites and identified 309 metabolite ratios based on the human metabolome database (HMDB). The dataset IDs ranged from GCST90199621 to GCST90201020.

All GWAS and FinnGen data used in this study were obtained from publicly available summary-level datasets [31, 32]. The original studies providing these data had received ethical approval from relevant institutional review boards, and all participants had given informed consent [33]. Since our analysis involved only the secondary use of existing datasets, additional ethical approval was not required [34]. To avoid potential bias from sample overlap, we ensured that the DMiVD case-control cohorts were entirely independent from the datasets used for immune cells, gut microbiota, inflammatory proteins, lipidomics, metabolomics, and micronutrients. This approach strengthened the reliability of our causal inferences.

### 2.3 SNP Selection and Instrumental Variable Validation

Adhering to widely accepted MR selection criteria, we selected single-nucleotide polymorphisms (SNPs) significantly associated with immune cells, inflammatory proteins, plasma lipids, metabolites, micronutrients, and DMiVD status (*P* < 5×10^−8^) as well as with gut microbiota (*P* < 5×10^−6^) to serve as IVs [35-37]. To ensure independence between IVs, we applied stringent linkage disequilibrium (LD) clumping (r^2^ < 0.001 within a 10,000 kb window) [38]. Variants with minor allele frequency (MAF) < 0.01 were excluded to avoid population stratification bias [39]. IV strength was evaluated using the F-statistic, and SNPs with F < 10 were removed to prevent weak instrument bias [40]. Outcome-associated SNPs were extracted from the IEU OpenGWAS and FinnGen databases [41]. Exposure and outcome datasets were harmonized, and ambiguous palindromic SNPs (A/T or G/C) were excluded [42]. Potential pleiotropic variants (**e.g.*,* how specific metabolites mediate immune cell function) were identified using LDlink (https://ldlink.nih.gov) and subsequently removed from the analysis [43].

For each SNP, we extracted the following information: chromosomal position, effect allele (EA), other allele (OA), effect allele frequency (EAF), effect size (β), standard error (SE), and Association *P*-value [44]. The proportion of variance explained (R^2^) was calculated as: R^2^ = 2×EAF× (1-EAF) ×β^2^ / (2×EAF×(1-EAF) ×β^2^ + 2×EAF×(1-EAF) ×N×SE^2^), where N represents the effective sample size. Instrument strength was assessed using: F = R^2^×(N-2)/(1-R^2^) [45].

### 2.4 MR Analysis

All analyses were performed in R (v4.4.2) with the MR- PRESSO (v1.0) and TwoSampleMR (v0.6.8) packages. Causal estimation methods were selected based on SNP availability: When only one SNP was available, we employed the Wald ratio method for estimation; whereas in cases where two or more SNPs were available, the inverse variance weighted (IVW) method was used as the primary approach to assess the causal relationship between exposure and outcome [24, 46]. Complementary MR approaches were implemented for sensitivity analyses, including MR-Egger regression, simple mode, weighted median, and weighted mode [23, 47]. The Steiger directionality test was used to assess whether the hypothesized causal direction was consistent with the data. Quality control measures included: heterogeneity assessment (Cochran's Q test, *P*<0.05 threshold), pleiotropy evaluation (MR-PRESSO global test and MR-Egger intercept, *P*>0.05 indicating no bias), and leave-one-out analysis for influential SNP identification [48]. We implemented multiple testing corrections using two complementary approaches. First, multiple testing correction employed Bonferroni-adjusted thresholds: setting the following adjusted *P*-value thresholds: 6.84×10^−5^ (0.05/731), 1.06×10^−4^ (0.05/473), 3.57×10^−5^ (0.05/1400), 2.79×10^−4^ (0.05/179), 5.49×10^−4^ (0.05/91), and 2.50×10^−3^ (0.05/20) [49]. Associations meeting *P*<0.05 but not surpassing corrected thresholds were classified as suggestive. Second, to account for potential inflation of type I error rates due to multiple testing, we adjusted the primary IVW estimates using the Benjamini-Hochberg false discovery rate (FDR) correction. Associations with FDR-adjusted *P*-values < 0.1 were considered statistically significant, while those with nominal *p*-values < 0.05 but FDR ≥ 0.1 were regarded as suggestive evidence [50].

### 2.5 Mediation Analysis

Selected immune cells, gut microbiota, plasma lipids, and metabolites showing: First, significant causal associations with DMiVD, ensuring no heterogeneity and pleiotropy among these factors. Second, assessed immune cell effects on candidate mediators using identical selection criteria. Third, conducted two-step MR analysis examining: the total effect of immune cells on DMiVD (βall), the effect of immune cells on potential mediators (including gut microbiota, plasma lipidome, and metabolites) (β1), and the effect of these mediators on DMiVD (β2). For positive βall: β1 and β2 must share direction (both positive/negative) [51]. Conversely, for negative βall: β1 and β2 must have opposing signs. Subsequently, the mediation effect was calculated using the product of coefficients method (β1×β2), and the proportion mediated was estimated as the ratio of the mediation effect to the total effect: (β1×β2) / βall. The 95% confidence interval (CI) for the mediation effect was determined using the Delta method [52]. Additionally, the direct effect was obtained by subtracting the mediation effect from the total effect: (βall – β1×β2). Mediation effects with *P*<0.05 were considered statistically significant.

### 2.6 Enrichment Analysis

We first identified SNPs significantly associated with both immune cells and DMiVD risk, then annotated them using g:Profiler (https://biit.cs.ut.ee/gprofiler/snpense), a versatile bioinformatics resource that enables SNP-to-gene mapping, functional enrichment assessment, and cross-species ortholog alignment [53]. Subsequent functional analyses of corresponding genes were performed *via* the Bioinformatics Platform (https://www.bioinformatics.com.cn/), employing R packages including clusterProfiler for gene ontology (GO) / Kyoto encyclopedia of genes and genomes (KEGG) enrichment (*P* < 0.05 threshold), org.Hs.eg.db for gene annotation, and visualization tools (ggplot2, ComplexHeatmap) for data interpretation.

## 3 RESULTS

### 3.1 Diabetic Nephropathy: Causal Effects of Immune Cells, Gut Microbiota, Plasma Lipidome, Plasma Metabolites, Micronutrients, and Mediation Analysis

As illustrated in Figs. (**2A**-**E**), forward MR analysis identified 2 immune cells, 7 gut microbiota, 11 lipid species, 36 plasma metabolites, and 1 micronutrient significantly associated with diabetic nephropathy. Using the IVW method, Family *Enterobacteriaceae* (OR = 0.755, 95% CI: 0.625 ~ 0.912), 5,6-dihydrouridine (OR = 0.770, 95% CI: 0.632 ~ 0.939), and Sterol ester (27:1/18:3) (OR = 0.869, 95% CI: 0.755 ~ 0.999) were found to be negatively associated with diabetic nephropathy risk. While Genus *Citrobacter A* (OR = 1.583, 95% CI: 1.079 ~ 2.322), Phosphatidylinositol (18:1_18:2) (OR = 1.271, 95% CI:1.075 ~ 1.504), and 5-methylthioadenosine (MTA) to phosphate (OR = 1.231, 95% CI: 1.001 ~ 1.513) were found to be positively associated with diabetic nephropathy risk, acting risk factors. These findings suggest that members of the Family *Enterobacteriaceae (*e.g.*, Escherichia, Klebsiella)* may confer protection against diabetic nephropathy, potentially through modulating gut barrier function and endotoxin tolerance. In contrast, members of the Genus *Citrobacter A (*e.g.*, Citrobacter freundii)* could exacerbate disease risk by promoting inflammatory pathways. This provides genetic support for microbiota- targeted interventions in diabetic complications. Consistent results were observed across supplementary MR methods, including MR-Egger, weighted median, simple mode, and weighted mode analyses (Supplementary Figs. **1A**-**E**). Additionally, reverse MR analysis was performed to evaluate the potential causal effects of diabetic nephropathy on these factors (Supplementary Figs. **1F**-**J**). The results indicated that Genus *Syntrophorhabdia, X-18922,* and *1-(1-enyl-palmitoyl)-2-arachidonoyl-gpc (p-16:0/20:4)* show a significant influence of diabetic nephropathy on these factors. Sensitivity analyses confirmed the robustness of these findings, with no evidence of significant heterogeneity and horizontal pleiotropy (Supplementary Tables **1**, **2**).

**Fig. (2):**
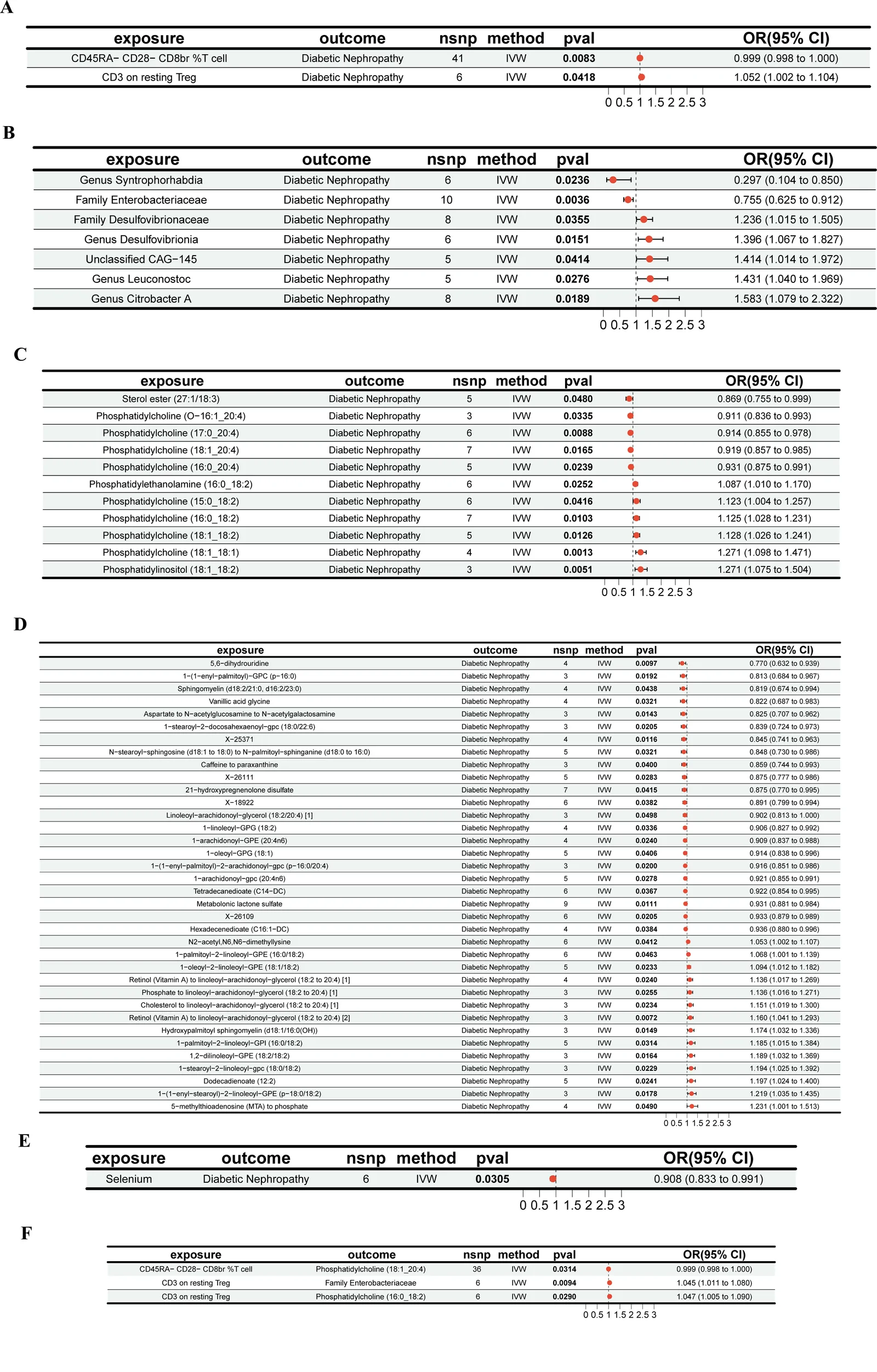
Bidirectional MR results for diabetic nephropathy. (**A**) Immune cell associations (**B**) Gut microbiota associations (**C**) Plasma lipidome associations (**D**) Plasma metabolite associations (**E**) Micronutrient associations (**F**) Mediation analysis. The odds ratios (ORs), 95% confidence intervals (CIs), and *P*-values were calculated for each Mendelian randomization analysis method. A *P*-value < 0.05 was considered statistically significant. **Abbreviations:** NSNP: number of single nucleotide polymorphisms; IVW: inverse-variance weighted.

Using variables selected for robustness and causal validity, we performed a two-step MR analysis to evaluate potential mediation effects. Based on screening criteria, specific gut microbiota and plasma lipid species mediated immune cell effects in diabetic nephropathy (Fig. **2F**). Sensitivity analyses, including assessments of heterogeneity and horizontal pleiotropy, confirmed the robustness and reliability of these results (Supplementary Tables **3**, **4**). Subsequently, we performed a mediation analysis to assess the impact of immune cells on these mediators and their contribution to modulation of diabetic nephropathy risk (Table **1**). Our results revealed that the detrimental effect of CD3 on resting Treg cells in diabetic nephropathy was partially mediated by phosphatidylcholine (16:0_18:2), with the proportion of 10.7%.

### 3.2 Diabetic Retinopathy: Causal Effects of Immune Cells, Gut Microbiota, Inflammatory Proteins, Plasma Lipidome, and Metabolites

Through forward MR analysis, 16 immune cells, 14 gut microbiota, 2 inflammatory proteins, 20 lipid species and 31 plasma metabolites were identified as having causal relationships with diabetic retinopathy in Figs. (**3A**-**E**). Using the IVW method as the primary analytical approach, we observed that the lower level of species *Clostridium M sp001304855* (OR = 0.776, 95% CI: 0.605 ~ 0.995), 2-stearoyl-GPE (18:0) (OR = 0.817, 95% CI: 0.714 ~ 0.935), Triacylglycerol (54:7) (OR = 0.832, 95% CI: 0.742 ~ 0.933) were associated with an increased risk of diabetic retinopathy, while the higher level of unclassified *Bacilli A* (OR = 7.099, 95% CI: 1.930 ~ 26.104), (2 or 3)-decenoate (10:1n7 or n8) (OR = 1.189, 95% CI: 1.026 ~ 1.377), CD25 on IgD- CD38- B cell (OR = 1.148, 95% CI: 1.050 ~ 1.254), were associated with an increased risk of diabetic retinopathy. These results indicate that *Clostridium M sp001304855* (a Clostridium species) is associated with protective effects in diabetic retinopathy, potentially *via* modulating retinal inflammation, while unclassified *Bacilli A* (an unclassified *Bacilli* taxon) is linked to disease progression, possibly through promoting vascular disruption. Highlighting these specific microbial taxa enhances biological interpretability by associating individual species with distinct clinical phenotypes. The robustness of these findings was supported by consistent results from four additional MR methods (Supplementary Figs. **2A**-**E**). To evaluate potential reverse causation, we performed reverse MR analysis to assess the effect of diabetic retinopathy on these factors. The results revealed that *species Lachnospira rogosae*, *genus An181,* and (S)-a-amino-omega-caprolactam show significant causal impacts of diabetic retinopathy (Supplementary Figs. **2F**-**J**). Sensitivity analyses further validated the results, demonstrating no significant heterogeneity or horizontal pleiotropy (Supplementary Tables **5**, **6**).

**Fig. (3):**
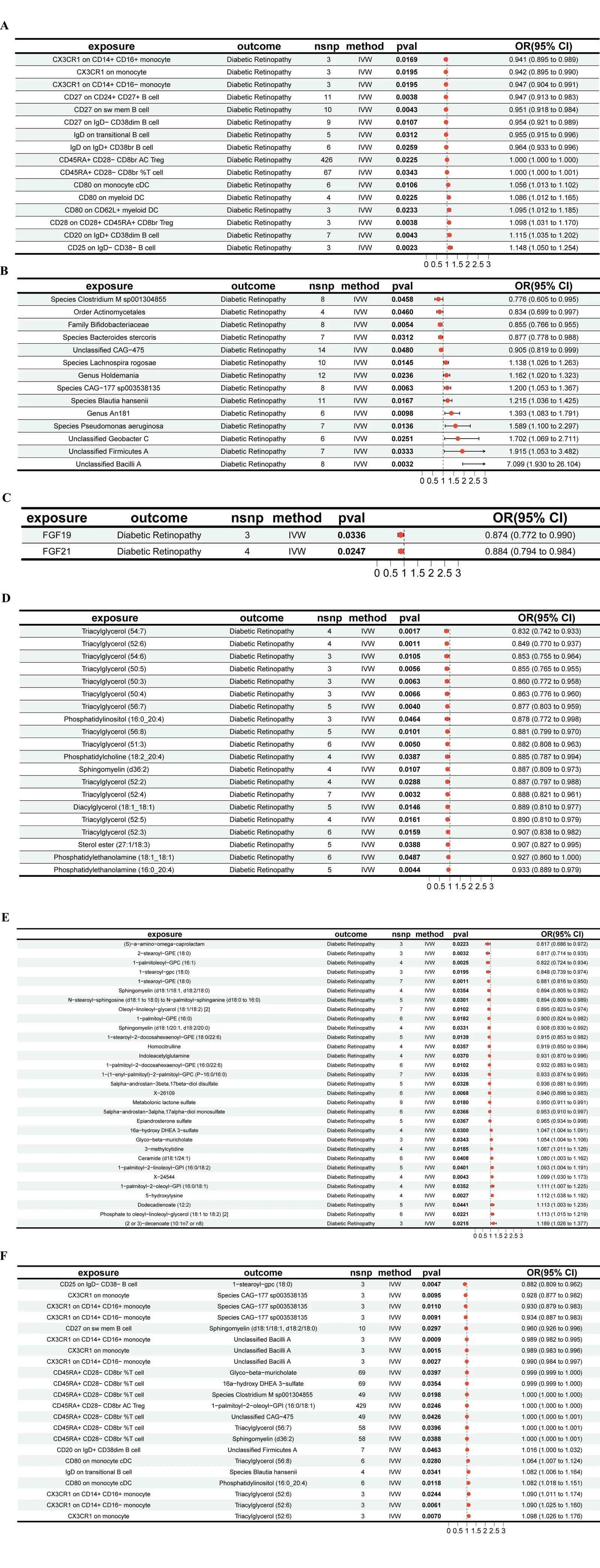
Bidirectional MR results for diabetic retinopathy. (**A**) Immune cell associations (**B**) Gut microbiota associations (**C**) Inflammatory proteins associations (**D**) Plasma lipidome associations (**E**) Plasma metabolite associations (**F**) Mediation analysis. The odds ratios (ORs), 95% confidence intervals (CIs), and *P*-values were calculated for each Mendelian randomization analysis method. A *P*-value < 0.05 was considered statistically significant. **Abbreviations:** NSNP: number of single nucleotide polymorphisms; IVW: inverse-variance weighted.

Using those selected for robustness and causal validity in variables, we performed a two-step MR analysis to evaluate potential mediation effects. Based on screening criteria, specific gut microbiota, plasma lipid species and metabolites mediated immune cell effects in diabetic retinopathy (Fig. **3F**). Sensitivity assessments, which incorporated analyses of heterogeneity and horizontal pleiotropy, validated the consistency and trustworthiness of these results (Supplementary Tables **7**, **8**). Subsequently, we performed mediation analysis to assess the impact of immune cells on these mediators and their contribution to diabetic retinopathy risk modulation (Table **2**). Our results revealed that the protective effects of CX3CR1 on total monocytes as well as CD14+ CD16− and CD14+ CD16+ monocyte subsets against diabetic retinopathy appeared to be jointly mediated through two distinct mechanisms: an elevation of triacylglycerol 52:6, which accounted for 25.5%, 25.9% and 23% of the mediation effect in these cell populations respectively, coupled with a suppression of Unclassified *Bacilli A* and species *CAG-177 sp003538135*, demonstrating mediation proportions of 35%, 34.4% and 35.8% for the former and 22.6%, 22.8% and 21.7% for the latter across the same monocyte subsets.

### 3.3 Diabetic Neuropathy: Causal Effects of Immune Cells, Gut Microbiota, Inflammatory Proteins, Plasma Metabolites, and Micronutrients

**Fig. (4):**
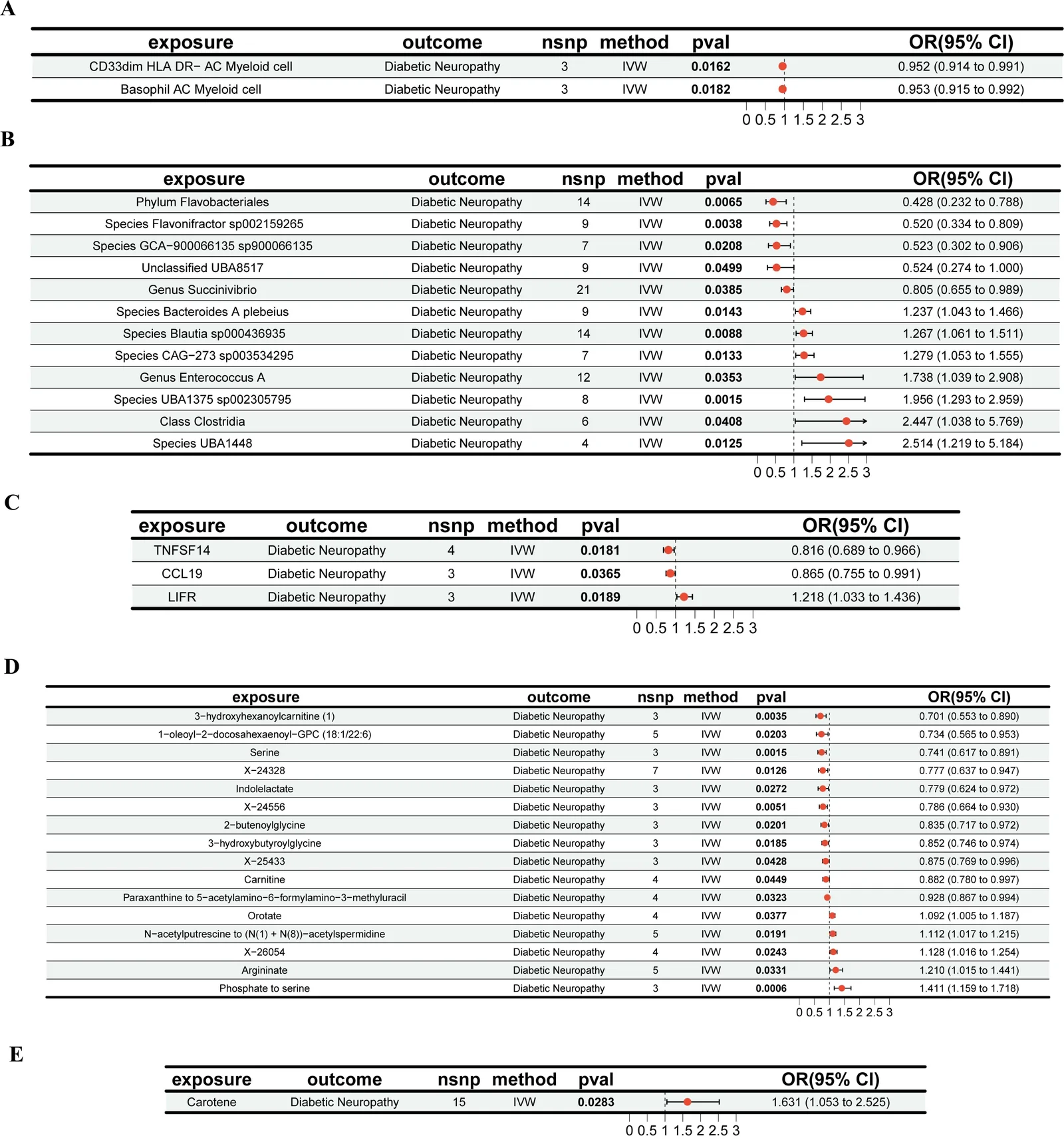
Bidirectional MR results for diabetic neuropathy. (**A**) Immune cell associations (**B**) Gut microbiota associations (**C**) Inflammatory protein associations (**D**) Plasma metabolite associations (**E**) Micronutrient associations. The odds ratios (ORs), 95% confidence intervals (CIs), and *P*-values were calculated for each Mendelian randomization analysis method. A *P*-value < 0.05 was considered statistically significant. **Abbreviations:** NSNP: number of single nucleotide polymorphisms; IVW: inverse-variance weighted.

Using bidirectional MR analysis, we revealed that 2 immune cell phenotypes, 12 gut microbiota, 3 inflammatory proteins, 16 plasma metabolites and 1 micronutrient were significantly associated with diabetic neuropathy (Figs. **4A**-**E**). However, based on the available evidence, we observed no significant indication of a causal association between plasma lipidome and diabetic neuropathy. Notably, Protective phenotypes such as *Phylum Flavobacteriales*, 3-hydroxyhexanoylcarnitine (1), and tumor necrosis factor superfamily member 14 (TNFSF14) were associated with a reduced risk of diabetic neuropathy, with ORs of 0.428 (95% CI: 0.232 ~ 0.788), 0.701 (95% CI: 0.553 ~ 0.890), and 0.816 (95% CI: 0.689 ~ 0.966), respectively. These results suggest a significant protective effect against diabetic neuropathy. In contrast, the risk phenotypes including species *UBA1448*, phosphate to serine, and leukemia inhibitory factor receptor (LIFR) were associated with an increased risk of diabetic neuropathy, with ORs of 2.514 (95% CI: 1.219 ~ 5.184), 1.411 (95% CI: 1.159 ~ 1.718), and 1.218 (95% CI: 1.033 ~ 01.436), respectively. *Phylum Flavobacteriales* may protect against diabetic neuropathy, possibly *via* modulating neuroinflammation, while species *UBA1448* is linked to disease progression, possibly by driving pro-inflammatory signaling. Highlighting these taxa enhances biological interpretability by associating specific microbes with distinct outcomes. Furthermore, the causal relationships were consistent across four additional MR methods (Supplementary Figs. **3A**-**E**). We also assessed the reverse causal relationship between diabetic neuropathy and these factors. Reverse MR analysis indicated that unclassified *UBA8517* and c-c motif chemokine ligand 19 (CCL19) showed significant effects (Supplementary Figs. **3F**-**J**) and sensitivity analyses revealed no significant heterogeneity and pleiotropy, further supporting the robustness and reliability of the results (Supplementary Tables **9**, **10**).

However, no significant mediators were found for diabetic neuropathy, suggesting that gut microbiota, inflammatory proteins, plasma lipid species, metabolites, and micronutrients may not participate in immune cell-related mechanisms of diabetic neuropathy pathogenesis.

After bidirectional MR and mediation analysis of immune cell phenotypes, gut microbial taxa, inflammatory proteins, lipid types, plasma metabolites, micronutrients and DMiVD cases, we have summarized a table listing all significant immune-microbial-lipid-metabolite associations, with effect sizes, CIs, and FDR-adjusted *P*-values in Supplementary Table **11**.

### 3.4 Results of Enrichment Analysis

**Fig. (5):**
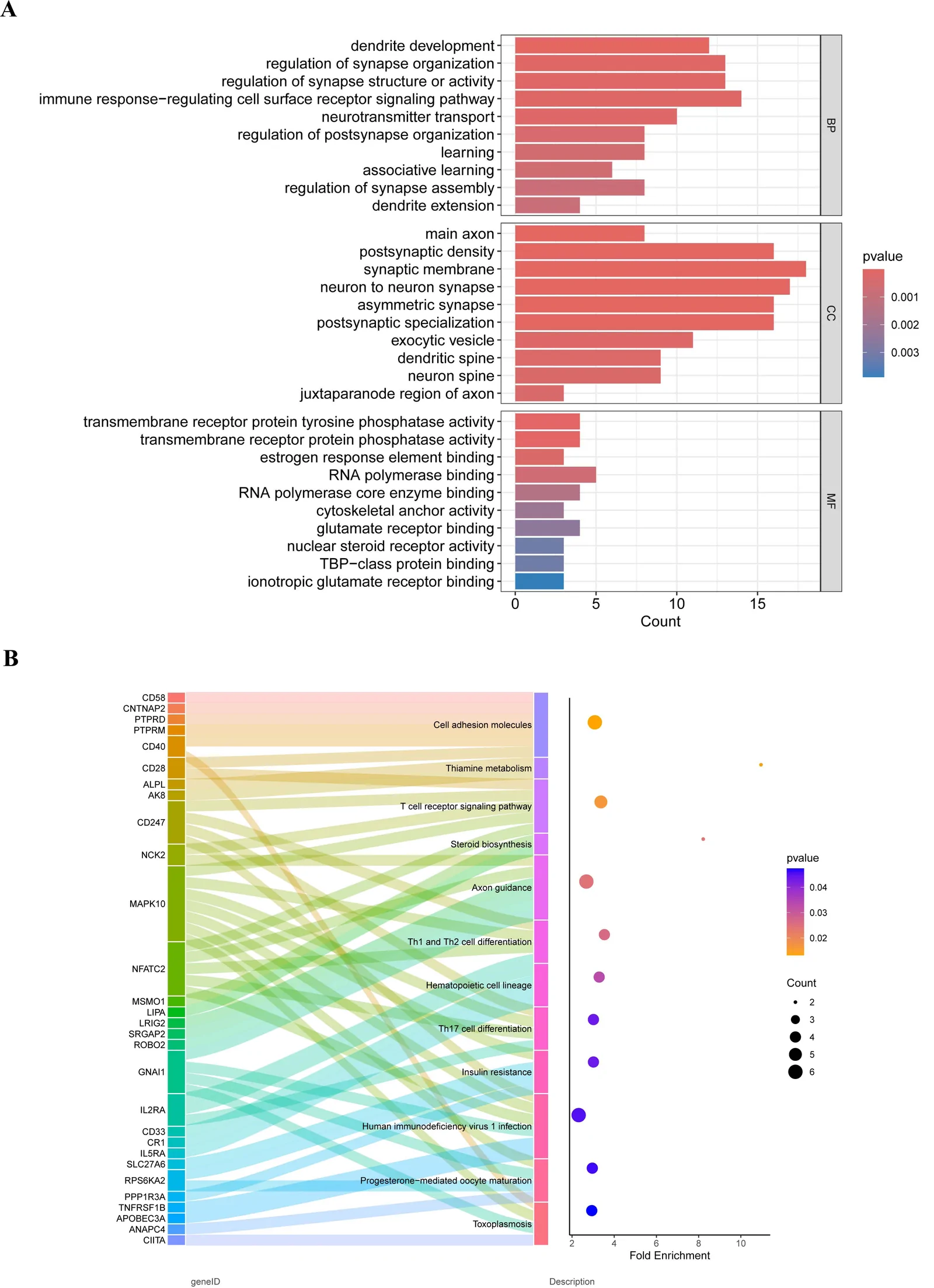
Enrichment analysis. (**A**) GO analysis, including biological processes (BPs), molecular functions (MFs), and cellular components (CCs). (**B**) KEGG pathway enrichment analysis.

Converting the SNPs significantly associated with immune cells and DMiVD into genes, enrichment analysis was performed, as detailed in Fig. (**5**) and Supplementary Table **12**. The top five clusters included synaptic membrane, neuron to neuron synapses, postsynaptic density, asymmetric synapse and postsynaptic specialization (Fig. **5A**). The KEGG pathway enrichment analysis revealed that the genetic influence of immune cells on DMiVD primarily centered on cell adhesion molecules, thiamine metabolism, T cell receptor signaling pathway, insulin resistance and toxoplasmosis (Fig. **5B**).

## 4 DISCUSSION

The integration of two-sample MR and mediation analysis in this study elucidates the causal pathways through which gut microbiota, plasma lipidome, and metabolites contribute to immune dysregulation in DMiVD. We identified specific microbial taxa, lipid types, and metabolites, such as phosphatidylcholine and triacylglycerols, that mediate immune cell dysfunction, including Treg depletion and CX3CR1+ monocyte alterations, providing mechanistic insights into DMiVD pathogenesis. However, several critical aspects, including the broader relevance of these findings to DMiVD and the translational potential of microbiota-targeted interventions, merit further discussion to strengthen the novelty and biological plausibility of our work.

Our study advances current understanding by employing a comprehensive MR and mediation framework to dissect the interplay between gut dysbiosis, metabolic perturbations, and immune dysfunction, which has not been concurrently explored in prior MR-based investigations. Existing work often focuses on isolated aspects, such as gut microbiota associations with diabetes complications or lipid metabolites influencing neuropathy risk. We uniquely integrate mediation analysis to demonstrate how immune cells act as intermediaries [17, 22, 54, 55]. For example, bidirectional MR analyses have been applied to infer causality between gut microbiota and diabetic complications such as nephropathy or neuropathy, but few studies incorporate mediators like immune cells to establish causal pathways [17, 22]. These pathways and mediation mechanisms represent a significant novelty, moving beyond correlational observations to causal inference, thereby addressing gaps in inflammation-driven microvascular complications.

Phosphatidylcholine and Tregs are both implicated in promoting the progression of diabetic nephropathy. Phosphatidylcholine species may mediate the detrimental effects of Tregs in diabetic nephropathy pathogenesis, as lipid metabolism alterations such as phosphatidylcholine dysregulation are established drivers of renal injury in diabetes [56-58]. Specifically, studies on intrarenal lipid accumulation indicate that locally elevated phosphatidylcholine derivatives contribute to tubular lipotoxicity, a key mechanism in diabetic nephropathy progression [59]. Dysregulated adipokines in diabetic nephropathy drive immune cell infiltration, including Tregs, and lipid metabolic reprogramming, further exacerbating renal damage [56].

In contrast, triacylglycerol and CX3CR1+ monocytes exhibit protective roles in diabetic retinopathy. Triacylglycerol likely mediates the beneficial effects of CX3CR1+ monocytes, as lipidomic analyses reveal that specific triacylglycerol isoforms (*e.g.*, TAG 51:4, TAG 53:4) are associated with reduced inflammatory and vascular damage in diabetic retinopathy [57, 60]. CX3CR1+ monocytes modulate retinal inflammation and vascular integrity, processes influenced by systemic lipid profiles [61, 62]. Triacylglycerol metabolism is linked to immune cell-mediated protection against microangiopathies, including reduced monocyte infiltration and cytokine release in diabetic retinopathy models [60, 62, 63].

These findings underscore the tissue-specific duality of lipid-immune crosstalk: phosphatidylcholine exacerbates diabetic nephropathy through Treg-mediated pathways, while triacylglycerol attenuates diabetic retinopathy *via* CX3CR1+ monocyte-dependent mechanisms [56-61].

Our findings provide a framework for novel therapeutic strategies targeting the gut microbiota and lipidome to mitigate DMiVD. By demonstrating causal links, including the mediation of immune dysregulation by gut microbiota-derived metabolites, our work underscores the potential for microbiota-based interventions, which have been proposed as promising approaches for improving outcomes in DMiVD [54, 64]. For example, modulating gut microbiota diversity has been causally linked to reduced nephropathy risk, and similar strategies could extend to immune-focused therapies [22, 65]. Targeted lipid modulation, such as enhancing protective phosphatidylcholine species, could offer personalized therapeutic avenues, potentially slowing disease progression through immune restoration [59, 66, 67]. This approach advances current paradigms by highlighting integrative targets beyond glucose control, guiding future clinical trials to test microbiota or metabolite modulators as adjuvants in DMiVD management [64, 67].

The singular association of carnitene, a vitamin A-related metabolite, with neuropathy risk contrasts with observational reports on vitamin B. This can be rationalized by our MR design’s focus on genetic proxies, which may prioritize specific pathways. While vitamin B links to neuropathy are documented observationally, our approach identified carnitene as a causal metabolite after rigorous adjustment for multiple comparisons, reflecting its potential role in lipid metabolism and immune regulation [54, 55, 68]. This divergence highlights how MR isolates direct genetic effects, potentially uncovering novel targets not apparent in observational contexts.

The lack of conclusions on anthocyanins improving retinal lesions may stem from the genetic instruments used, as anthocyanins were not included in our metabolite panel due to data availability constraints. While dietary studies suggest benefits, MR analyses require specific metabolite GWAS data, which were not captured in our dataset [69]. Future work should prioritize high-throughput metabolomics to test such compounds.

While MR minimizes reverse causality by leveraging genetic instruments, potential limitations remain. Our analysis explicitly tested reverse causation using bidirectional MR, sensitivity analyses, horizontal pleiotropy tests, and heterogeneity assessments. However, unmeasured confounders, such as unassessed environmental factors, may persist, which is an inherent limitation of MR designs [70, 71].

Restriction to European ancestry datasets limits the generalizability of our findings. Although large-scale GWAS data provide robust instruments for MR, they predominantly represent European cohorts, which may not capture population-specific variations in genetic architecture or microbiota composition [17, 25, 54]. Caution is warranted when extrapolating these associations to non-European populations, as disparities in microbiota-disease links could alter risk estimates. Future research should prioritize multi-ethnic studies to validate and refine these causal pathways.

The reliance on genus-level categorization for gut microbiota may obscure species- or strain-specific effects, potentially masking finer mechanistic details. This limitation is common in MR studies utilizing summary-level GWAS data, which aggregate microbial data at higher taxonomic levels [18, 72]. While this approach identifies broad causal links, species-level resolution could enhance biological insights, underscoring the need for refined genetic instruments in future work.

As MR relies on genetic prediction, it does not capture real-time environmental influences such as diet or medication on the microbiota-metabolic axis [67, 73]. Nonetheless, our findings provide genetic evidence for causal pathways, which environmental modulators may amplify or mitigate. For example, diet-induced microbiota changes can alter metabolite profiles, influencing diabetes progression [68, 73]. Integrating MR with experimental designs could elucidate these interactions [67, 74].

To strengthen causal inferences, we recommend validating key findings through animal models or clinical trials. For example, animal studies demonstrate that gut microbiota interventions modulate metabolites and ameliorate diabetic phenotypes, and clinical trials testing microbiota-targeted therapies have shown promise [75, 76]. Implementing such studies could confirm the therapeutic potential of our identified microbiota-immune axes.

## CONCLUSION

This study, using multidimensional MR analysis, identified causal relationships and mediation mechanisms among immune cells, gut microbiota, inflammatory proteins, plasma lipidome, metabolites, and micronutrients in DMiVD, providing novel insights into disease pathogenesis. These findings support the integration of DMiVD management into existing diabetes care frameworks. For instance, they can guide microbiome-modulating therapies, such as restoring protective microbial compositions, or precision lipid-targeted strategies to address tissue-specific lipid effects, while highlighting immune regulatory hubs as actionable targets. Targeting key gut microbiota, lipidome pathways, and immune genes may represent promising therapeutic strategies. However, experimental validation, including animal models and clinical trials, is essential to confirm causality and translate these findings into clinical practice.

## STUDY LIMITATIONS

This study has certain limitations. Although Mendelian randomization reduces confounding and reverse causation, it relies on the validity of genetic instrumental variables, and residual pleiotropy or unmeasured confounders cannot be completely excluded. In addition, the use of summary-level data precluded individual-level and environmental interaction analyses. Therefore, further experimental and clinical studies are needed to validate the biological mechanisms and translational relevance of these findings.

## AUTHORS’ CONTRIBUTIONS

The authors confirm their contributions the paper as follows: J.Z.: contributed to conceptualization, methodology, formal analysis, and writing. Z.L. and L.K.: contributed to conceptualization, methodology, and data curation. H.Y., H.G., and Y.Z.: were responsible for software development and validation. S.W., D.L., S.L. and X.L.: contributed to the investigation and data validation. L.W. contributed to conceptualization, project administration, and supervision. All authors have meticulously reviewed the results and approved the final version for publication.

## Figures and Tables

**Fig. (1):**
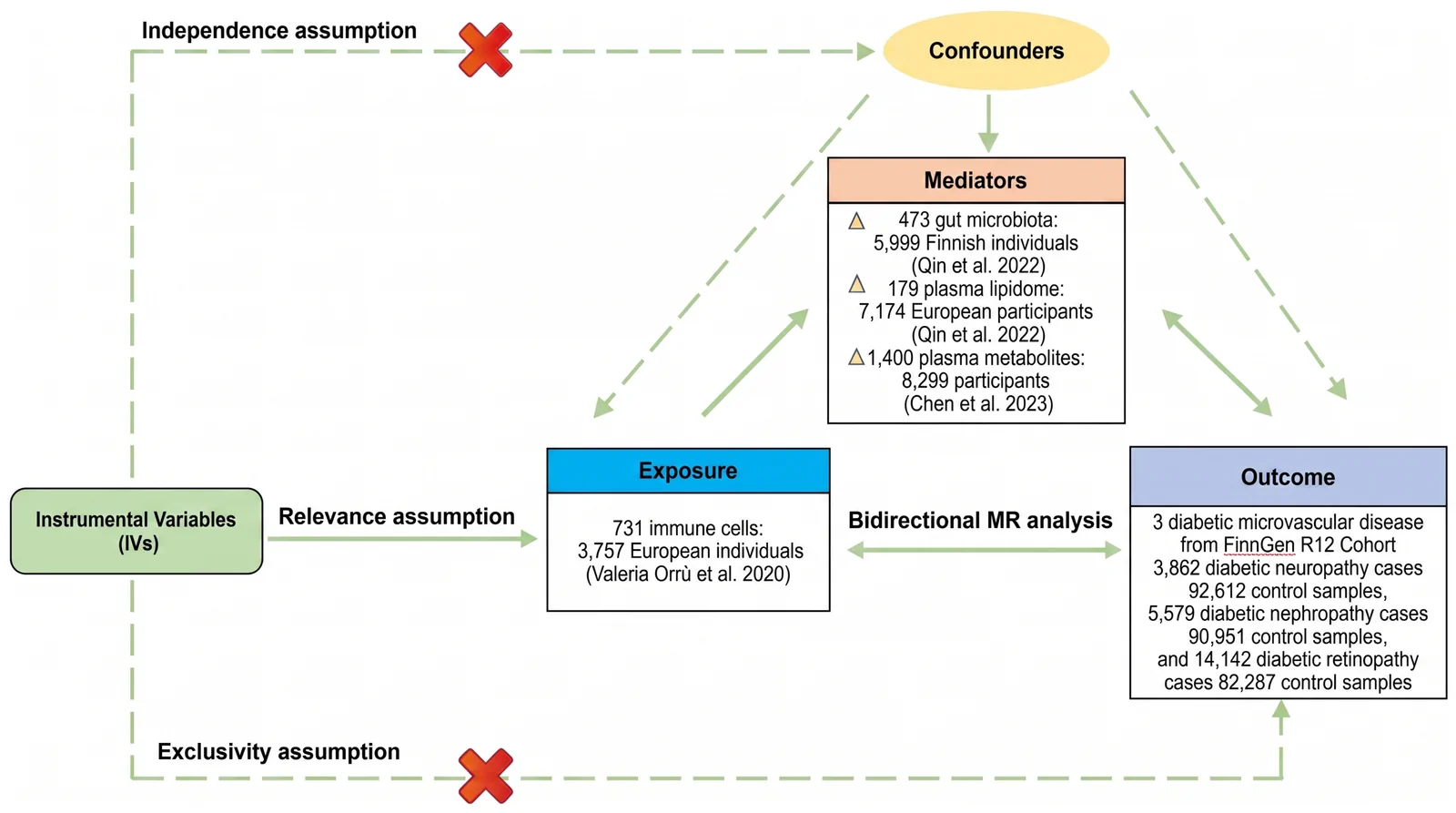
Study design. Relevance: IVs show a significant exposure association. Independence: IVs must remain unconfounded by exposure-outcome covariates. Exclusivity: IVs influence outcome exclusively through exposure pathways.

**Table 1:** Mediation effects and proportions of each mediator in diabetic nephropathy.

**Exposure** **(Immune Cell)**	**Mediator** **(Gut Microbiota)**	**Outcome**	**Mediated Effect**	**Mediated Proportion**	**Direct Effect**	***P*-value**
CD3 on resting Treg	Family Enterobacteriaceae	diabetic nephropathy	-0.0123 (-0.0216, -0.00289)	-24.3% (-42.9%, -5.74%)	0.063	0.0103
**Exposure** **(Immune Cells)**	**Mediator** **(Plasma Lipidome)**	**Outcome**	**Mediated Effect**	**Mediated Proportion**	**Direct Effect**	***P*-value**
CD3 on resting Treg	Phosphatidylcholine (16:0_18:2)	diabetic nephropathy	0.00537 (0.000203, 0.0105)	10.7% (0.403%, 20.9%)	0.045	0.0417
CD45RA- CD28- CD8br %T cell	Phosphatidylcholine (18:1_20:4)	diabetic nephropathy	7.31e-05 (6.52e-06, 0.00014)	-7.12% (-13.6%, -0.634%)	-0.001	0.0314

**Table 2:** Mediation effects and proportions of each mediator in diabetic retinopathy.

**Exposure** **(Immune Cells)**	**Mediator** **(Gut Microbiota)**	**Outcome**	**Mediated Effect**	**Mediated Proportion**	**Direct Effect**	***P*-value**
CD20 on IgD+ CD38dim B cell	Unclassified Firmicutes A	diabetic retinopathy	0.0104 (0.000167, 0.0206)	9.51% (0.153%, 18.9%)	0.099	0.0464
CD45RA+ CD28- CD8br %T cell	Species Clostridium M sp001304855	diabetic retinopathy	6.21e-05 (9.84e-06, 0.000114)	17.3% (2.74%, 31.9%)	<0.001	0.0198
CD45RA+ CD28- CD8br %T cell	Unclassified CAG-475	diabetic retinopathy	-4.14e-05 (-8.15e-05, -1.38e-06)	-11.6% (-22.7%, -0.386%)	<0.001	0.0426
CX3CR1 on monocyte	Unclassified Bacilli A	diabetic retinopathy	-0.021 (-0.034, -0.00804)	35% (13.4%, 56.5%)	-0.039	0.0015
CX3CR1 on monocyte	Species CAG-177 sp003538135	diabetic retinopathy	-0.0136 (-0.0247, -0.00249)	22.6% (4.15%, 41.1%)	-0.047	0.0164
CX3CR1 on CD14+ CD16- monocyte	Unclassified Bacilli A	diabetic retinopathy	-0.0188 (-0.0311, -0.00651)	34.4% (11.9%, 57%)	-0.036	0.0027
CX3CR1 on CD14+ CD16- monocyte	Species CAG-177 sp003538135	diabetic retinopathy	-0.0124 (-0.0224, -0.00246)	22.8% (4.5%, 41%)	-0.042	0.0146
CX3CR1 on CD14+ CD16+ monocyte	Unclassified Bacilli A	diabetic retinopathy	-0.0219 (-0.0348, -0.009)	35.8% (14.7%, 57%)	-0.039	0.0009
CX3CR1 on CD14+ CD16+ monocyte	Species CAG-177 sp003538135	diabetic retinopathy	-0.0133 (-0.0243, -0.00225)	21.7% (3.68%, 39.7%)	-0.048	0.0183
IgD on transitional B cell	Species Blautia hansenii	diabetic retinopathy	0.0153 (3.53e-05, 0.0306)	-32.9% (-65.8%, -0.0758%)	-0.062	0.0495
CD45RA+ CD28- CD8br %T cell	Triacylglycerol (56:7)	diabetic retinopathy	-6.24e-05 (-0.000122, -2.98e-06)	-17.4% (-34%, -0.831%)	<0.001	0.0396
CD45RA+ CD28- CD8br %T cell	Sphingomyelin (d36:2)	diabetic retinopathy	-5.73e-05 (-0.000122, -2.96e-06)	-16% (-31.1%, -0.825%)	<0.001	0.0388
CD80 on monocyte	Phosphatidylinositol (16:0_20:4)	diabetic retinopathy	-0.0103 (-0.0197, -0.000929)	-18.9% (-36.1%, -1.7%)	0.065	0.0313
CD80 on monocyte	Triacylglycerol (56:8)	diabetic retinopathy	-0.00783 (-0.0156, -7.07e-05)	-14.3% (-28.5%, -0.129%)	0.063	0.0479
CX3CR1 on monocyte	Triacylglycerol (52:6)	diabetic retinopathy	-0.0154 (-0.0282, -0.0025)	25.5% (4.16%, 46.9%)	-0.045	0.0192
CX3CR1 on CD14+ CD16- monocyte	Triacylglycerol (52:6)	diabetic retinopathy	-0.0142 (-0.0256, -0.00271)	25.9% (4.95%, 46.9%)	-0.040	0.0154
CX3CR1 on CD14+ CD16+ monocyte	Triacylglycerol (52:6)	diabetic retinopathy	-0.014 (-0.0278, -0.00023)	23% (0.376%, 45.5%)	-0.047	0.0463
CD25 on IgD- CD38- B cell	1-stearoyl-gpc (18:0)	diabetic retinopathy	0.0206 (0.00269, 0.0385)	15% (1.95%, 28%)	0.117	0.0242
CD27 on sw mem B cell	Sphingomyelin (d18:1/18:1, d18:2/18:0)	diabetic retinopathy	0.00455 (0.000187, 0.00892)	-9% (-0.37%, -17.6%)	-0.055	0.0410
CD45RA+ CD28- CD8br AC Treg	1-palmitoyl-2-oleoyl-GPI (16:0/18:1)	diabetic retinopathy	-1.26e-06 (-2.36e-06, -1.57e-07	-15.6% (-29.3%, -1.95%)	<0.001	0.0252
CD45RA+ CD28- CD8br %T cell	16a-hydroxy DHEA 3-sulfate	diabetic retinopathy	-2.3e-05 (-4.44e-05, -1.57e-06)	-6.41% (-12.4%, -0.439%)	<0.001	0.0354
CD45RA+ CD28- CD8br %T cell	Glyco-beta-muricholate	diabetic retinopathy	-2.9e-05 (-5.67e-05, -1.36e-06)	-8.09% (-15.8%, -0.379%)	<0.001	0.0397

## Data Availability

The data and supporting information are available within the article.

## LIST OF ABBREVIATIONS

β: Effect Sizes
BPs: Biological Processes
CCs: Cellular Components
CCL19: c-c Motif Chemokine Ligand 19
CI: Confidence Interval
CLSA: Canadian Longitudinal Study on Aging
cDCs: conventional Dendritic Cells
DMiVD: Diabetic Microvascular Disease
EA: Effect Allele
EAF: Effect Allele Frequency
FDR: False Discovery Rate
GO: Gene Ontology
GWAS: Genome-Wide Association Studies
HD4: High-Definition Mass Spectrometry
HMDB: Human Metabolome Database
IVs: Instrumental Variables
IVW: Inverse Variance Weighted
KEGG: Kyoto Encyclopedia of Genes and Genomes
LIFR: Leukemia Inhibitory Factor Receptor
LD: Linkage Disequilibrium
MAF: Minor Allele Frequency
MFs: Molecular Functions
MR: Mendelian Randomization
MSCs: Mesenchymal Stem Cells
MTA: Methylthioadenosine
OA: Other Allele
OR: Odds Ratio
SE: Standard Error
SNPs: Single Nucleotide Polymorphisms
STROBE-MR: Strengthening the Reporting of Observational Studies in Epidemiology using Mendelian Randomization
TBNK cells: T cells, B cells, and Natural Killer cells
TNFSF14: Tumor Necrosis Factor Superfamily Member 14
Tregs: Regulatory T Cells

## STANDARDS OF REPORTING

STROBE guidelines were followed.

## References

[1] Horton W.B., Barrett E.J. (2021). Microvascular dysfunction in diabetes mellitus and cardiometabolic disease.. Endocr. Rev..

[2] Xu R., Fang Z., Wang H., Gu Y., Yu L., Zhang B., Xu J. (2024). Molecular mechanism and intervention measures of microvascular complications in diabetes.. Open Med..

[3] Saha B., Roy A., Beltramo E., Sahoo O.S. (2023). Stem cells and diabetic retinopathy: From models to treatment.. Mol. Biol. Rep..

[4] Zooravar D., Soltani P., Khezri S. (2025). Mediterranean diet and diabetic microvascular complications: A systematic review and meta-analysis.. BMC Nutr..

[5] Van Roy N., Speeckaert M.M. (2024). The potential use of targeted proteomics and metabolomics for the identification and monitoring of diabetic kidney disease.. J. Pers. Med..

[6] He F., Ng Yin Ling C., Nusinovici S., Cheng C.Y., Wong T.Y., Li J., Sabanayagam C. (2024). Development and external validation of machine learning models for diabetic microvascular complications: Cross-sectional study with metabolites.. J. Med. Internet Res..

[7] Zhang S., Yang X., Jiang M., Ma L., Hu J., Zhang H.H. (2022). Post- transcriptional control by RNA-binding proteins in diabetes and its related complications.. Front. Physiol..

[8] Zhou T., Fang Y.L., Tian T.T., Wang G.X. (2024). Pathological mechanism of immune disorders in diabetic kidney disease and intervention strategies.. World J. Diabetes.

[9] Shu Z., Chen S., Xiang H., Wu R., Wang X., Ouyang J., Zhang J., Liu H., Chen A.F., Lu H. (2022). AKT/PACS2 participates in renal vascular hyperpermeability by regulating endothelial fatty acid oxidation in diabetic mice.. Front. Pharmacol..

[10] Zhu H., Chen Y., Gong W., Lai J., Yao B., Zhao Z., Lu Q., Ye K., Ji L., Xu J. (2022). Shared and specific biological signalling pathways for diabetic retinopathy, peripheral neuropathy and nephropathy by high-throughput sequencing analysis.. Diab. Vasc. Dis. Res..

[11] Hou G., Dong Y., Jiang Y., Zhao W., Zhou L., Cao S., Li W. (2025). Immune inflammation and metabolic interactions in the pathogenesis of diabetic nephropathy.. Front. Endocrinol..

[12] Tan S.M., Snelson M., Østergaard J.A., Coughlan M.T. (2022). The complement pathway: New insights into immunometabolic signaling in diabetic kidney disease.. Antioxid. Redox Signal..

[13] Qu L., Jiao B. (2023). The interplay between immune and metabolic pathways in kidney disease.. Cells.

[14] Peng Q.Y., An Y., Jiang Z.Z., Xu Y. (2024). The role of immune cells in DKD: Mechanisms and targeted therapies.. J. Inflamm. Res..

[15] Li Y., Wang B., Xu M.T., Wang Y.Y., Liu W.Q., Fu S.J., Li B.W., Ling H., Liu X.T., Zhang X.Y., Li A.L., Zhang X., Liu M.M. (2025). Interdisciplinary perspectives on diabetes and microcirculatory dysfunction: A global bibliometric analysis.. World J. Diabetes.

[16] Ren Y., Zhang H. (2024). The causal effect of inflammatory proteins and immune cell populations on diabetic nephropathy: Evidence from Mendelian randomization.. Int. Urol. Nephrol..

[17] Xu M., Hao J., Qi Y., Wu B., Li R., Yang X., Zhang Y., Liu Y. (2024). Causal effects of gut microbiota on diabetic neuropathy: A two-sample Mendelian randomization study.. Front. Endocrinol..

[18] Han S., Chen Y., Lu Y., Jia M., Xu Y., Wang Y. (2024). Association between gut microbiota and diabetic nephropathy: A two-sample Mendelian randomization study.. BMC Endocr. Disord..

[19] Ye Z., So T., Zhang T., Gao X. (2024). Association between gut microbiota and diabetic nephropathy: A two-sample Mendelian randomization study.. Front. Endocrinol..

[20] Song S., Ning L., Yu J. (2025). Elucidating the causal relationship between gut microbiota, metabolites, and diabetic nephropathy in European patients: Revelations from genome-wide bidirectional Mendelian randomization analysis.. Front. Endocrinol..

[21] Cao B.N., Zhang C.Y., Wang Z., Wang Y.X. (2025). Causal relationship between 412 gut microbiota, 1,400 blood metabolites, and diabetic nephropathy: A randomized Mendelian study.. Front. Endocrinol..

[22] Yan W., Ge Y., Wang L., Wang Y., He D. (2024). Causal relationship of gut microbiota with diabetic nephropathy: A Mendelian randomization analysis.. Front. Microbiol..

[23] Song S., Zhang Q., Zhang L., Zhou X., Yu J. (2024). A two-sample bidirectional Mendelian randomization analysis investigates associations between gut microbiota and type 2 diabetes mellitus.. Front. Endocrinol..

[24] Skrivankova V.W., Richmond R.C., Woolf B.A.R., Yarmolinsky J., Davies N.M., Swanson S.A., VanderWeele T.J., Higgins J.P.T., Timpson N.J., Dimou N., Langenberg C., Golub R.M., Loder E.W., Gallo V., Tybjaerg-Hansen A., Davey Smith G., Egger M., Richards J.B. (2021). Strengthening the reporting of observational studies in epidemiology using Mendelian randomization: The STROBE-MR statement.. JAMA.

[25] Kurki M.I., Karjalainen J., Palta P., Sipilä T.P., Kristiansson K., Donner K.M., Reeve M.P., Laivuori H., Aavikko M., Kaunisto M.A., Loukola A., Lahtela E., Mattsson H., Laiho P., Della Briotta Parolo P., Lehisto A.A., Kanai M., Mars N., Rämö J., Kiiskinen T., Heyne H.O., Veerapen K., Rüeger S., Lemmelä S., Zhou W., Ruotsalainen S., Pärn K., Hiekkalinna T., Koskelainen S., Paajanen T., Llorens V., Gracia-Tabuenca J., Siirtola H., Reis K., Elnahas A.G., Sun B., Foley C.N., Aalto-Setälä K., Alasoo K., Arvas M., Auro K., Biswas S., Bizaki- Vallaskangas A., Carpen O., Chen C.Y., Dada O.A., Ding Z., Ehm M.G., Eklund K., Färkkilä M., Finucane H., Ganna A., Ghazal A., Graham R.R., Green E.M., Hakanen A., Hautalahti M., Hedman Å.K., Hiltunen M., Hinttala R., Hovatta I., Hu X., Huertas-Vazquez A., Huilaja L., Hunkapiller J., Jacob H., Jensen J.N., Joensuu H., John S., Julkunen V., Jung M., Junttila J., Kaarniranta K., Kähönen M., Kajanne R., Kallio L., Kälviäinen R., Kaprio J., Kerimov N., Kettunen J., Kilpeläinen E., Kilpi T., Klinger K., Kosma V.M., Kuopio T., Kurra V., Laisk T., Laukkanen J., Lawless N., Liu A., Longerich S., Mägi R., Mäkelä J., Mäkitie A., Malarstig A., Mannermaa A., Maranville J., Matakidou A., Meretoja T., Mozaffari S.V., Niemi M.E.K., Niemi M., Niiranen T., O´Donnell C.J., Obeidat M., Okafo G., Ollila H.M., Palomäki A., Palotie T., Partanen J., Paul D.S., Pelkonen M., Pendergrass R.K., Petrovski S., Pitkäranta A., Platt A., Pulford D., Punkka E., Pussinen P., Raghavan N., Rahimov F., Rajpal D., Renaud N.A., Riley-Gillis B., Rodosthenous R., Saarentaus E., Salminen A., Salminen E., Salomaa V., Schleutker J., Serpi R., Shen H., Siegel R., Silander K., Siltanen S., Soini S., Soininen H., Sul J.H., Tachmazidou I., Tasanen K., Tienari P., Toppila-Salmi S., Tukiainen T., Tuomi T., Turunen J.A., Ulirsch J.C., Vaura F., Virolainen P., Waring J., Waterworth D., Yang R., Nelis M., Reigo A., Metspalu A., Milani L., Esko T., Fox C., Havulinna A.S., Perola M., Ripatti S., Jalanko A., Laitinen T., Mäkelä T.P., Plenge R., McCarthy M., Runz H., Daly M.J., Palotie A. (2023). FinnGen provides genetic insights from a well-phenotyped isolated population.. Nature.

[26] Orrù V., Steri M., Sidore C., Marongiu M., Serra V., Olla S., Sole G., Lai S., Dei M., Mulas A., Virdis F., Piras M.G., Lobina M., Marongiu M., Pitzalis M., Deidda F., Loizedda A., Onano S., Zoledziewska M., Sawcer S., Devoto M., Gorospe M., Abecasis G.R., Floris M., Pala M., Schlessinger D., Fiorillo E., Cucca F. (2020). Complex genetic signatures in immune cells underlie autoimmunity and inform therapy.. Nat. Genet..

[27] Qin Y., Havulinna A.S., Liu Y., Jousilahti P., Ritchie S.C., Tokolyi A., Sanders J.G., Valsta L., Brożyńska M., Zhu Q., Tripathi A., Vázquez-Baeza Y., Loomba R., Cheng S., Jain M., Niiranen T., Lahti L., Knight R., Salomaa V., Inouye M., Méric G. (2022). Combined effects of host genetics and diet on human gut microbiota and incident disease in a single population cohort.. Nat. Genet..

[28] Zhao J.H., Stacey D., Eriksson N., Macdonald-Dunlop E., Hedman Å.K., Kalnapenkis A., Enroth S., Cozzetto D., Digby-Bell J., Marten J., Folkersen L., Herder C., Jonsson L., Bergen S.E., Gieger C., Needham E.J., Surendran P., Metspalu A., Milani L., Mägi R., Nelis M., Hudjašov G., Paul D.S., Polasek O., Thorand B., Grallert H., Roden M., Võsa U., Esko T., Hayward C., Johansson Å., Gyllensten U., Powell N., Hansson O., Mattsson-Carlgren N., Joshi P.K., Danesh J., Padyukov L., Klareskog L., Landén M., Wilson J.F., Siegbahn A., Wallentin L., Mälarstig A., Butterworth A.S., Peters J.E. (2023). Genetics of circulating inflammatory proteins identifies drivers of immune-mediated disease risk and therapeutic targets.. Nat. Immunol..

[29] Ottensmann L., Tabassum R., Ruotsalainen S.E., Gerl M.J., Klose C., Widén E., Simons K., Ripatti S., Pirinen M. (2023). Genome-wide association analysis of plasma lipidome identifies 495 genetic associations.. Nat. Commun..

[30] Chen Y., Lu T., Pettersson-Kymmer U., Stewart I.D., Butler-Laporte G., Nakanishi T., Cerani A., Liang K.Y.H., Yoshiji S., Willett J.D.S., Su C.Y., Raina P., Greenwood C.M.T., Farjoun Y., Forgetta V., Langenberg C., Zhou S., Ohlsson C., Richards J.B. (2023). Genomic atlas of the plasma metabolome prioritizes metabolites implicated in human diseases.. Nat. Genet..

[31] Yan S., Sha Q., Zhang S. (2022). Control for population stratification in genetic association studies based on GWAS summary statistics.. Genet. Epidemiol..

[32] Beck T., Rowlands T., Shorter T., Brookes A.J. (2023). GWAS Central: An expanding resource for finding and visualising genotype and phenotype data from genome-wide association studies.. Nucleic Acids Res..

[33] Amin S.M., Atta M.H.R., Khedr M.A., El-Gazar H.E., Zoromba M.A. (2025). Impact of moral resilience and interprofessional collaboration on nurses’ ethical competence.. Nurs. Ethics.

[34] Lee S.W., Hwang I.S., Jung G., Kang H.J., Chung Y.H. (2022). Relationship between metabolic syndrome and follicle-stimulating hormone in postmenopausal women.. Medicine.

[35] Zhang X., Zhou H., Meng Q., Wang W., Zhou L., Xu Y., Zhang H., Zhang Z., Cheng J., Zhan X., Li J., Hu C., Zhou F., Yang D., Wang L., Chu Y., Peng H. (2025). The causal relationship between immune cell phenotypes and esophageal cancer development: A bidirectional Mendelian randomization study.. Discov. Oncol..

[36] Jia P., Hao Z., Yiu K., Tsoi K. (2025). Causal effects between blood pressure variability and Alzheimer’s disease: A two-sample Mendelian randomization study.. J. Clin. Hypertens..

[37] Wang Y., Li W., Ha C. (2024). A large-scale causal analysis of gut microbiota and endometriosis associated infertility: A Mendelian randomization study.. Medicine.

[38] Liu C.G., Li D.Y., Gao X., Ma T., Zhang K., Liu D.Y. (2024). Examining the causal relationship between circulating immune cells and the susceptibility to osteomyelitis: A Mendelian randomization study.. Int. Immunopharmacol..

[39] Wu Z., Peng Q., Ren Z., Xu X., Jiang X., Yang W., Han Y., Oyang L., Lin J., Peng M., Wu N., Tang Y., Liao Q., Zhou Y. (2025). Causal association between oral microbiota and oral cancer: A Mendelian randomization study.. Sci. Rep..

[40] Cheng G., Xu L., Li H., Cheng Y., Jiang T., Xu Q., Wang J. (2025). Causal links between personality disorders and schizophrenia: A Mendelian randomization study.. Medicine.

[41] Lu C., Chen Q., Tao H., Xu L., Li J., Wang C., Yu L. (2023). The causal effect of inflammatory bowel disease on diffuse large B- cell lymphoma: Two-sample Mendelian randomization study.. Front. Immunol..

[42] Wu L., Liao F., Guo X., Li N. (2024). The causal effect of adipose tissue on Hodgkin’s lymphoma: Two-sample Mendelian randomization study and validation.. Front. Immunol..

[43] Jia Y., Chen H., Huang S., Huo Z., Xu B. (2024). Causal effects of skin microbiota on intervertebral disk degeneration, low back pain and sciatica: A two-sample Mendelian randomization study.. J. Orthop. Surg. Res..

[44] Jiao R., Li W., Song J., Chen Z. (2024). Causal association between asthma and periodontitis: A two-sample Mendelian randomization analysis.. Oral Dis..

[45] Xiao X., Wu X., Yi L., You F., Li X., Xiao C. (2024). Causal linkage between type 2 diabetes mellitus and inflammatory bowel disease: An integrated Mendelian randomization study and bioinformatics analysis.. Front. Endocrinol..

[46] Wang C., Xie W., Wang C., Zhu Y., Zhong D. (2025). Causal relationships between environmental exposures, iron metabolism, hematuria markers, and rheumatoid arthritis: An investigation using Mendelian randomization.. Biomedicines.

[47] Yang H., Shi P., Li M., Kong L., Liu S., Jiang L., Yang J., Xu B., Yang T., Xi S., Liu W. (2023). Mendelian-randomization study reveals causal relationships between nitrogen dioxide and gut microbiota.. Ecotoxicol. Environ. Saf..

[48] Yu L., Long Q., Zhang Y., Liu Y., Guo Z., Cao X., Qin F., Xu Y., Qian Q., Gao B., Chen J., Liu J., Zeng Y., Teng Z. (2025). Bidirectional Mendelian randomization analysis of plasma lipidome and psychiatric disorders.. J. Affect. Disord..

[49] Liu Z., Zhou X., Kuang L., Chen Q., Zhao J., Yin H., Zhou Z., Liu X., Liu D., Wu S., Wu L. (2025). Novel insights into immune-gut microbiota interactions in colorectal cancer: A Mendelian randomization study.. Infect. Agent. Cancer.

[50] Guo W., Hong E., Ma H., Wang J., Wang Q. (2025). Effect of the gut microbiome, skin microbiome, plasma metabolome, white blood cells subtype, immune cells, inflammatory proteins, and inflammatory cytokines on asthma: A two-sample Mendelian randomized study and mediation analysis.. Front. Immunol..

[51] Pan H., Liu C., Zhu H., Zhang G. (2024). Immune cells mediate the effects of gut microbiota on neuropathic pain: A Mendelian randomization study.. J. Headache Pain.

[52] Xiao X.Y., Chen Q., Shi Y.Z., Li L.W., Hua C., Zheng H. (2023). Risk factors of systemic lupus erythematosus: An overview of systematic reviews and Mendelian randomization studies.. Adv. Rheumatol..

[53] Kolberg L., Raudvere U., Kuzmin I., Vilo J., Peterson H. (2020). gprofiler2- an R package for gene list functional enrichment analysis and namespace conversion toolset g:Profiler.. F1000 Res..

[54] Tang F., Shen L., Gu Z., Zhang L., Fang L., Sun H., Ma D., Guo Y., Yang Y., Lu B., Li Q., Zhong S., Wang Z. (2024). Causal relationships between gut microbiota, gut metabolites, and diabetic neuropathy: A Mendelian randomization study.. Clin. Nutr. ESPEN.

[55] Wang Z., Liu Z., Yang Q., Qiao H., Yin Y., Zhao Z., Shao X. (2025). Causal relationships between plasma lipidome and diabetic neuropathy: A Mendelian randomization study.. Front. Endocrinol..

[56] Wang X., Abu Bakar M.H., Kassim M.A., Shariff K.A., Wang J., Xu M. (2025). Exploring the interplay between adipokine-mediated celastrol target genes and T cells in diabetic nephropathy: A Mendelian randomization-based causal inference.. Diabetol. Metab. Syndr..

[57] Jing Y., Zhu H., Yao P., Chen Y., Lai X., He Q., Yu L., Lin Y., Kang D. (2025). IgD-CD38-B cell partially mediates the protective effect of higher serum triacylglycerol (53:4) levels against Parkinson’s disease.. J. Neurochem..

[58] Li Y., Wang H., Xiao Y., Yang H., Wang S., Liu L., Cai H., Zhang X., Tang H., Wu T., Qiu G. (2024). Lipidomics identified novel cholesterol-independent predictors for risk of incident coronary heart disease: Mediation of risk from diabetes and aggravation of risk by ambient air pollution.. J. Adv. Res..

[59] Pressly J.D., Ge M., Fornoni A. (2022). LPCing through the nephron accelerates diabetic kidney disease.. Kidney Int..

[60] Blot G., Karadayi R., Przegralek L., Sartoris T.M., Charles-Messance H., Augustin S., Negrier P., Blond F., Muñiz-Ruvalcaba F.P., Rivera-de la Parra D., Vignaud L., Couturier A., Sahel J.A., Acar N., Jimenez-Corona A., Delarasse C., Garfias Y., Sennlaub F., Guillonneau X. (2023). Perilipin 2–positive mononuclear phagocytes accumulate in the diabetic retina and promote PPARγ-dependent vasodegeneration.. J. Clin. Invest..

[61] Mickael M.E., Kubick N., Miftari K., Horbańczuk J.O., Atanasov A.G., Binçe K., Religa P., Kamińska A., Sacharczuk M., Ławiński M. (2025). The role of Th17/treg axis in retinal pathology associated with diabetes and treatment options.. Biology (Basel).

[62] Li Q., Zhang M., Gao Q., Chen X. (2025). High fat-induced the upregulation of LOX-1 in RF/6A cells under high glucose condition.. J. Diabetes Complications.

[63] Zhang S., Wei X., Bowers M., Jessberger S., Golczak M., Semenkovich C.F., Rajagopal R. (2023). Increasing energetic demands on photoreceptors in diabetes corrects retinal lipid dysmetabolism and reduces subsequent microvascular damage.. Am. J. Pathol..

[64] Zhou P., Hao Z., Chen Y., Zhang Z., Xu W., Yu J. (2024). Association between gut microbiota and diabetic microvascular complications: A two-sample Mendelian randomization study.. Front. Endocrinol..

[65] Chen K., Wang X., Shang Z., Li Q., Yao W., Guo S., Guan Y. (2025). Exploring the causal effects of gut microbiota on diabetic nephropathy: A two-sample Mendelian randomization study.. Comb. Chem. High Throughput Screen..

[66] Wei Y., Yu J. (2024). The association between plasma lipidome and diabetic microangiopathy: A Mendelian randomization study.. Acta Diabetol..

[67] Garcia-Gutierrez E., O’Mahony A.K., Dos Santos R.S., Marroquí L., Cotter P.D. (2024). Gut microbial metabolic signatures in diabetes mellitus and potential preventive and therapeutic applications.. Gut Microbes.

[68] Liu B., Zhang L., Yang H., Zheng H., Liao X. (2023). Microbiota: A potential orchestrator of antidiabetic therapy.. Front. Endocrinol..

[69] Serban D., Dascalu A., Arsene A., Tribus L., Vancea G., Pantea Stoian A., Costea D., Tudosie M., Stana D., Cristea B., Nicolae V., Tudor C., Costea A., Comandasu M., Faur M., Tanasescu C. (2023). Gut microbiota dysbiosis in diabetic retinopathy-current knowledge and future therapeutic targets.. Life.

[70] Koneru H.M., Sarwar H., Bandi V.V., Sinha M., Tarar P., Bishara R., Malasevskaia I. (2024). A systematic review of gut microbiota diversity: A key player in the management and prevention of diabetes mellitus.. Cureus.

[71] Jing F., Zhou J., Zhang F., Zhao G., Fang F., Pan X. (2025). Exploring the relationship between gut microbiota and aortic stenosis: Role of inflammatory proteins, blood metabolites, and immune cells.. Int. J. Med. Sci..

[72] Liu J., Chen Y., Peng C. (2024). Causal relationship between gut microbiota and diabetic complications: A two-sample Mendelian randomization study.. Diabetol. Metab. Syndr..

[73] Donati Zeppa S., Gervasi M., Bartolacci A., Ferrini F., Patti A., Sestili P., Stocchi V., Agostini D. (2024). Targeting the gut microbiota for prevention and management of type 2 diabetes.. Nutrients.

[74] Li P., Tong T., Shao X., Han Y., Zhang M., Li Y., Lv X., Li H., Li Z. (2024). The synergism of *Lactobacillaceae*, inulin, polyglucose, and aerobic exercise ameliorates hyperglycemia by modulating the gut microbiota community and the metabolic profiles in db/db mice.. Food Funct..

[75] Wu Q., Wu S., Cheng Y., Zhang Z., Mao G., Li S., Yang Y., Zhang X., Wu M., Tong H. (2021). *Sargassum fusiforme* fucoidan modifies gut microbiota and intestinal metabolites during alleviation of hyperglycemia in type 2 diabetic mice.. Food Funct..

[76] Wang S., Ren H., Zhong H., Zhao X., Li C., Ma J., Gu X., Xue Y., Huang S., Yang J., Chen L., Chen G., Qu S., Liang J., Qin L., Huang Q., Peng Y., Li Q., Wang X., Zou Y., Shi Z., Li X., Li T., Yang H., Lai S., Xu G., Li J., Zhang Y., Gu Y., Wang W. (2022). Combined berberine and probiotic treatment as an effective regimen for improving postprandial hyperlipidemia in type 2 diabetes patients: A double blinded placebo controlled randomized study.. Gut Microbes.

