# Supplementary material for: Gut Microbiota, Lipidome, and Metabolites Mediate Immune Dysregulation in Diabetic Microvascular Disease: A Two-sample Mendelian Randomization and Mediation Analysis
Source: Endocr Metab Immune Disord Drug Targets. 2026 Jan 27;26:E18715303418611. doi: 10.2174/0118715303418611251125045911 (PMC13531661; doi:10.2174/0118715303418611251125045911)
Supplement: Supplementary file 1 [file EMIDDT-26-E18715303418611_SD1.pdf]

Supplementary Material

Gut Microbiota, Lipidome, and Metabolites Mediate Immune Dysregulation in Diabetic Microvascular Disease: A Two-sample Mendelian Randomization and Mediation Analysis

Jiaxing Zhao[2,#], Zenghui Liu[2,#], Lu Kuang[1], Huayu Yin[2], Heyue Gong[2], Yaolong Zheng[2], Shaoguo Wu[1], Dabin Liu[1], Shuyan Liu[1], Xuehui Liu[1] and Limei Wu[1,2,#, *]

[1]Department of Laboratory Medicine, the Affiliated Guangzhou Twelfth People's Hospital, Guangzhou Medical University, Guangzhou, Guangdong, China; [2]Department of Immunology, Mudanjiang Medical University, Mudanjiang, Heilongjiang, China

(A)

exposure	outcome	nsnp	method	pval	OR(95% CI)
CD3 on resting Treg	Diabetic Nephropathy	6	MR Egger	0.4465	1.037 (0.952 to 1.130)
		6	Weighted median	0.1268	1.045 (0.988 to 1.105)
		6	Inverse variance weighted	**0.0418**	1.052 (1.002 to 1.104)
		6	Simple mode	0.2689	1.054 (0.970 to 1.146)
		6	Weighted mode	0.2376	1.043 (0.981 to 1.110)
CD45RA− CD28− CD8br %T cell	Diabetic Nephropathy	41	MR Egger	0.1467	0.999 (0.998 to 1.000)
		41	Weighted median	0.0979	0.999 (0.998 to 1.000)
		41	Inverse variance weighted	**0.0083**	0.999 (0.998 to 1.000)
		41	Simple mode	0.4027	0.999 (0.998 to 1.001)
		41	Weighted mode	**0.0352**	0.999 (0.998 to 1.000)

1

(B)

exposure	outcome	nsnp	method	pval	OR(95% CI)
Unclassified CAG−145	Diabetic Nephropathy	5	MR Egger	0.5732	1.482 (0.436 to 5.032)
		5	Weighted median	0.0612	1.460 (0.982 to 2.170)
		5	Inverse variance weighted	**0.0414**	1.414 (1.014 to 1.972)
		5	Simple mode	0.2030	1.512 (0.887 to 2.577)
		5	Weighted mode	0.1746	1.496 (0.927 to 2.416)
Genus Citrobacter A	Diabetic Nephropathy	8	MR Egger	0.0527	2.291 (1.167 to 4.500)
		8	Weighted median	0.1877	1.392 (0.851 to 2.279)
		8	Inverse variance weighted	**0.0189**	1.583 (1.079 to 2.322)
		8	Simple mode	0.4985	1.285 (0.645 to 2.559)
		8	Weighted mode	0.4780	1.295 (0.659 to 2.544)
Family Desulfovibrionaceae	Diabetic Nephropathy	8	MR Egger	0.9676	1.014 (0.534 to 1.925)
		8	Weighted median	0.3093	1.146 (0.881 to 1.492)
		8	Inverse variance weighted	**0.0355**	1.236 (1.015 to 1.505)
		8	Simple mode	0.6687	1.099 (0.726 to 1.665)
		8	Weighted mode	0.6200	1.106 (0.755 to 1.620)
Genus Desulfovibrionia	Diabetic Nephropathy	6	MR Egger	0.5870	1.321 (0.523 to 3.337)
		6	Weighted median	0.2923	1.218 (0.844 to 1.759)
		6	Inverse variance weighted	**0.0151**	1.396 (1.067 to 1.827)
		6	Simple mode	0.5201	1.208 (0.707 to 2.065)
		6	Weighted mode	0.5090	1.190 (0.736 to 1.924)
Family Enterobacteriaceae	Diabetic Nephropathy	10	MR Egger	0.1451	0.668 (0.410 to 1.090)
		10	Weighted median	**0.0336**	0.761 (0.591 to 0.979)
		10	Inverse variance weighted	**0.0036**	0.755 (0.625 to 0.912)
		10	Simple mode	0.2198	0.758 (0.503 to 1.144)
		10	Weighted mode	0.2343	0.764 (0.505 to 1.156)
Genus Leuconostoc	Diabetic Nephropathy	5	MR Egger	0.3346	2.206 (0.571 to 8.524)
		5	Weighted median	0.1263	1.342 (0.920 to 1.957)
		5	Inverse variance weighted	**0.0276**	1.431 (1.040 to 1.969)
		5	Simple mode	0.1261	1.823 (0.990 to 3.358)
		5	Weighted mode	0.6644	1.145 (0.648 to 2.023)
Genus Syntrophorhabdia	Diabetic Nephropathy	6	MR Egger	**0.0397**	0.032 (0.003 to 0.303)
		6	Weighted median	0.0870	0.361 (0.112 to 1.159)
		6	Inverse variance weighted	**0.0236**	0.297 (0.104 to 0.850)
		6	Simple mode	0.2484	0.349 (0.072 to 1.694)
		6	Weighted mode	0.2774	0.376 (0.078 to 1.814)

1

(C)

exposure	outcome	nsnp	method	pval	OR(95% CI)
Phosphatidylcholine (15:0_18:2)	Diabetic Nephropathy	6	MR Egger	0.2569	1.201 (0.915 to 1.575)
		6	Weighted median	**0.0217**	1.136 (1.019 to 1.267)
		6	Inverse variance weighted	**0.0416**	1.123 (1.004 to 1.257)
		6	Simple mode	0.8048	1.031 (0.821 to 1.294)
		6	Weighted mode	0.0766	1.147 (1.017 to 1.294)
Phosphatidylcholine (16:0_18:2)	Diabetic Nephropathy	7	MR Egger	0.2192	1.168 (0.940 to 1.451)
		7	Weighted median	**0.0171**	1.140 (1.023 to 1.269)
		7	Inverse variance weighted	**0.0103**	1.125 (1.028 to 1.231)
		7	Simple mode	0.2972	1.113 (0.926 to 1.336)
		7	Weighted mode	0.0831	1.137 (1.007 to 1.283)
Phosphatidylcholine (16:0_20:4)	Diabetic Nephropathy	5	MR Egger	0.2476	0.926 (0.834 to 1.029)
		5	Weighted median	**0.0326**	0.931 (0.872 to 0.994)
		5	Inverse variance weighted	**0.0239**	0.931 (0.875 to 0.991)
		5	Simple mode	0.4435	0.943 (0.824 to 1.080)
		5	Weighted mode	0.0945	0.931 (0.873 to 0.993)
Phosphatidylcholine (17:0_20:4)	Diabetic Nephropathy	6	MR Egger	0.5524	0.957 (0.839 to 1.092)
		6	Weighted median	**0.0202**	0.921 (0.858 to 0.987)
		6	Inverse variance weighted	**0.0088**	0.914 (0.855 to 0.978)
		6	Simple mode	0.2253	0.892 (0.759 to 1.049)
		6	Weighted mode	0.0867	0.922 (0.856 to 0.994)
Phosphatidylcholine (18:1_18:1)	Diabetic Nephropathy	4	MR Egger	0.6239	1.159 (0.700 to 1.921)
		4	Weighted median	**0.0094**	1.256 (1.058 to 1.491)
		4	Inverse variance weighted	**0.0013**	1.271 (1.098 to 1.471)
		4	Simple mode	0.0807	1.364 (1.079 to 1.725)
		4	Weighted mode	0.1179	1.235 (1.021 to 1.495)
Phosphatidylcholine (18:1_18:2)	Diabetic Nephropathy	5	MR Egger	0.3624	1.121 (0.910 to 1.380)
		5	Weighted median	**0.0292**	1.120 (1.012 to 1.241)
		5	Inverse variance weighted	**0.0126**	1.128 (1.026 to 1.241)
		5	Simple mode	0.1702	1.185 (0.971 to 1.446)
		5	Weighted mode	0.0940	1.123 (1.012 to 1.246)
Phosphatidylcholine (18:1_20:4)	Diabetic Nephropathy	7	MR Egger	0.4730	0.950 (0.835 to 1.081)
		7	Weighted median	**0.0253**	0.917 (0.850 to 0.989)
		7	Inverse variance weighted	**0.0165**	0.919 (0.857 to 0.985)
		7	Simple mode	0.4950	0.935 (0.779 to 1.122)
		7	Weighted mode	0.0602	0.919 (0.856 to 0.987)
Phosphatidylcholine (O−16:1_20:4)	Diabetic Nephropathy	3	MR Egger	0.2859	0.828 (0.692 to 0.990)
		3	Weighted median	**0.0142**	0.899 (0.826 to 0.979)
		3	Inverse variance weighted	**0.0335**	0.911 (0.836 to 0.993)
		3	Simple mode	0.2254	0.902 (0.803 to 1.014)
		3	Weighted mode	0.1369	0.898 (0.823 to 0.980)
Phosphatidylethanolamine (16:0_18:2)	Diabetic Nephropathy	6	MR Egger	0.4541	1.103 (0.874 to 1.392)
		6	Weighted median	**0.0403**	1.102 (1.004 to 1.210)
		6	Inverse variance weighted	**0.0252**	1.087 (1.010 to 1.170)
		6	Simple mode	0.0924	1.145 (1.008 to 1.302)
		6	Weighted mode	0.1146	1.125 (0.997 to 1.270)
Phosphatidylinositol (18:1_18:2)	Diabetic Nephropathy	3	MR Egger	0.7183	1.486 (0.289 to 7.640)
		3	Weighted median	**0.0124**	1.290 (1.057 to 1.576)
		3	Inverse variance weighted	**0.0051**	1.271 (1.075 to 1.504)
		3	Simple mode	0.1332	1.364 (1.065 to 1.746)
		3	Weighted mode	0.1431	1.339 (1.050 to 1.708)
Sterol ester (27:1/18:3)	Diabetic Nephropathy	5	MR Egger	0.2556	0.765 (0.526 to 1.113)
		5	Weighted median	0.1573	0.879 (0.735 to 1.051)
		5	Inverse variance weighted	**0.0480**	0.869 (0.755 to 0.999)
		5	Simple mode	0.7149	0.947 (0.720 to 1.245)
		5	Weighted mode	0.1106	0.802 (0.649 to 0.991)

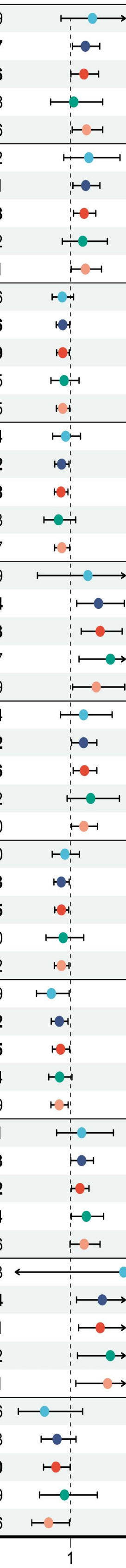

(D)

exposure	outcome	nsnp	method	pval	OR(95% CI)
1-(1-enyl-palmitoyl)-2-arachidonoyl-gpc (p-16:0/20:4)	Diabetic Nephropathy	3	MR Egger	0.3826	0.891 (0.763 to 1.041)
		3	Weighted median	**0.0182**	0.913 (0.847 to 0.985)
		3	Inverse variance weighted	**0.0200**	0.916 (0.851 to 0.986)
		3	Simple mode	0.2346	0.914 (0.823 to 1.015)
		3	Weighted mode	0.1579	0.913 (0.842 to 0.990)
1-(1-enyl-palmitoyl)-GPC (p-16:0)	Diabetic Nephropathy	3	MR Egger	0.6299	0.774 (0.361 to 1.661)
		3	Weighted median	**0.0391**	0.813 (0.668 to 0.990)
		3	Inverse variance weighted	**0.0192**	0.813 (0.684 to 0.967)
		3	Simple mode	0.2482	0.822 (0.647 to 1.043)
		3	Weighted mode	0.2071	0.808 (0.644 to 1.014)
1-(1-enyl-stearoyl)-2-linoleoyl-GPE (p-18:0/18:2)	Diabetic Nephropathy	3	MR Egger	0.3571	1.608 (0.896 to 2.887)
		3	Weighted median	**0.0323**	1.226 (1.017 to 1.478)
		3	Inverse variance weighted	**0.0178**	1.219 (1.035 to 1.435)
		3	Simple mode	0.1841	1.264 (1.004 to 1.590)
		3	Weighted mode	0.1782	1.229 (1.008 to 1.499)
1,2-dilinoleoyl-GPE (18:2/18:2)	Diabetic Nephropathy	3	MR Egger	0.4949	1.246 (0.815 to 1.906)
		3	Weighted median	**0.0253**	1.193 (1.022 to 1.392)
		3	Inverse variance weighted	**0.0164**	1.189 (1.032 to 1.369)
		3	Simple mode	0.2436	1.184 (0.967 to 1.450)
		3	Weighted mode	0.1653	1.198 (1.016 to 1.413)
1-arachidonoyl-gpc (20:4n6)	Diabetic Nephropathy	5	MR Egger	0.1660	0.890 (0.786 to 1.009)
		5	Weighted median	**0.0289**	0.916 (0.847 to 0.991)
		5	Inverse variance weighted	**0.0278**	0.921 (0.855 to 0.991)
		5	Simple mode	0.1630	0.845 (0.697 to 1.025)
		5	Weighted mode	0.0854	0.915 (0.847 to 0.988)
1-arachidonoyl-GPE (20:4n6)	Diabetic Nephropathy	4	MR Egger	0.1835	0.832 (0.695 to 0.996)
		4	Weighted median	**0.0198**	0.899 (0.823 to 0.983)
		4	Inverse variance weighted	**0.0240**	0.909 (0.837 to 0.988)
		4	Simple mode	0.2494	0.908 (0.794 to 1.037)
		4	Weighted mode	0.1133	0.897 (0.816 to 0.987)
1-linoleoyl-GPG (18:2)	Diabetic Nephropathy	4	MR Egger	0.2764	0.838 (0.664 to 1.058)
		4	Weighted median	**0.0266**	0.894 (0.810 to 0.987)
		4	Inverse variance weighted	**0.0336**	0.906 (0.827 to 0.992)
		4	Simple mode	0.1896	0.885 (0.769 to 1.020)
		4	Weighted mode	0.1270	0.894 (0.806 to 0.993)
1-oleoyl-2-linoleoyl-GPE (18:1/18:2)	Diabetic Nephropathy	5	MR Egger	0.1889	1.174 (0.975 to 1.413)
		5	Weighted median	**0.0244**	1.105 (1.013 to 1.205)
		5	Inverse variance weighted	**0.0233**	1.094 (1.012 to 1.182)
		5	Simple mode	0.2146	1.098 (0.970 to 1.242)
		5	Weighted mode	0.0918	1.103 (1.011 to 1.204)
1-oleoyl-GPG (18:1)	Diabetic Nephropathy	5	MR Egger	0.1671	0.852 (0.717 to 1.013)
		5	Weighted median	**0.0321**	0.899 (0.816 to 0.991)
		5	Inverse variance weighted	**0.0406**	0.914 (0.838 to 0.996)
		5	Simple mode	0.1756	0.888 (0.771 to 1.023)
		5	Weighted mode	0.1129	0.897 (0.808 to 0.997)
1-palmitoyl-2-linoleoyl-GPE (16:0/18:2)	Diabetic Nephropathy	6	MR Egger	0.5995	1.044 (0.900 to 1.212)
		6	Weighted median	0.0915	1.070 (0.989 to 1.157)
		6	Inverse variance weighted	**0.0463**	1.068 (1.001 to 1.139)
		6	Simple mode	0.6348	1.033 (0.912 to 1.169)
		6	Weighted mode	0.1497	1.069 (0.990 to 1.155)
1-palmitoyl-2-linoleoyl-GPI (16:0/18:2)	Diabetic Nephropathy	5	MR Egger	0.7589	1.103 (0.622 to 1.958)
		5	Weighted median	0.0735	1.157 (0.986 to 1.357)
		5	Inverse variance weighted	**0.0314**	1.185 (1.015 to 1.384)
		5	Simple mode	0.4425	1.114 (0.869 to 1.427)
		5	Weighted mode	0.2458	1.134 (0.946 to 1.360)
1-stearoyl-2-docosahexaenoyl-gpc (18:0/22:6)	Diabetic Nephropathy	3	MR Egger	0.8623	0.949 (0.593 to 1.518)
		3	Weighted median	**0.0381**	0.857 (0.741 to 0.992)
		3	Inverse variance weighted	**0.0205**	0.839 (0.724 to 0.973)
		3	Simple mode	0.3651	0.889 (0.730 to 1.084)
		3	Weighted mode	0.1877	0.860 (0.740 to 0.999)
1-stearoyl-2-linoleoyl-gpc (18:0/18:2)	Diabetic Nephropathy	3	MR Egger	0.4411	1.263 (0.864 to 1.845)
		3	Weighted median	**0.0244**	1.208 (1.025 to 1.424)
		3	Inverse variance weighted	**0.0229**	1.194 (1.025 to 1.392)
		3	Simple mode	0.1826	1.251 (1.005 to 1.557)
		3	Weighted mode	0.1584	1.214 (1.022 to 1.441)
21-hydroxypregnenolone disulfate	Diabetic Nephropathy	7	MR Egger	0.1946	0.744 (0.505 to 1.096)
		7	Weighted median	0.0801	0.858 (0.723 to 1.019)
		7	Inverse variance weighted	**0.0415**	0.875 (0.770 to 0.995)
		7	Simple mode	0.1754	0.824 (0.643 to 1.055)
		7	Weighted mode	0.1506	0.827 (0.660 to 1.037)
5,6-dihydrouridine	Diabetic Nephropathy	4	MR Egger	0.5700	0.891 (0.637 to 1.246)
		4	Weighted median	**0.0128**	0.757 (0.608 to 0.942)
		4	Inverse variance weighted	**0.0097**	0.770 (0.632 to 0.939)
		4	Simple mode	0.1323	0.715 (0.518 to 0.985)
		4	Weighted mode	0.1082	0.751 (0.586 to 0.962)
5-methylthioadenosine (MTA) to phosphate	Diabetic Nephropathy	4	MR Egger	0.6858	1.533 (0.256 to 9.166)
		4	Weighted median	0.2132	1.172 (0.913 to 1.504)
		4	Inverse variance weighted	**0.0490**	1.231 (1.001 to 1.513)
		4	Simple mode	0.5221	1.141 (0.798 to 1.632)
		4	Weighted mode	0.5863	1.110 (0.794 to 1.551)
Aspartate to N-acetylglucosamine to N-acetylgalactosamine	Diabetic Nephropathy	3	MR Egger	0.8015	0.926 (0.580 to 1.478)
		3	Weighted median	**0.0388**	0.840 (0.712 to 0.991)
		3	Inverse variance weighted	**0.0143**	0.825 (0.707 to 0.962)
		3	Simple mode	0.2491	0.850 (0.697 to 1.036)
		3	Weighted mode	0.2302	0.845 (0.697 to 1.025)
Caffeine to paraxanthine	Diabetic Nephropathy	3	MR Egger	0.3928	0.574 (0.266 to 1.242)
		3	Weighted median	0.0588	0.852 (0.722 to 1.006)
		3	Inverse variance weighted	**0.0400**	0.859 (0.744 to 0.993)
		3	Simple mode	0.3376	0.873 (0.705 to 1.080)
		3	Weighted mode	0.1971	0.832 (0.689 to 1.005)
Cholesterol to linoleoyl-arachidonoyl-glycerol (18:2 to 20:4) [1]	Diabetic Nephropathy	3	MR Egger	0.8568	1.068 (0.608 to 1.874)
		3	Weighted median	**0.0249**	1.149 (1.018 to 1.298)
		3	Inverse variance weighted	**0.0234**	1.151 (1.019 to 1.300)
		3	Simple mode	0.2186	1.173 (0.983 to 1.400)
		3	Weighted mode	0.2056	1.143 (0.992 to 1.317)
Dodecadienoate (12:2)	Diabetic Nephropathy	5	MR Egger	0.2743	1.228 (0.908 to 1.661)
		5	Weighted median	**0.0238**	1.252 (1.030 to 1.521)
		5	Inverse variance weighted	**0.0241**	1.197 (1.024 to 1.400)
		5	Simple mode	0.1027	1.312 (1.019 to 1.688)
		5	Weighted mode	0.1284	1.293 (0.994 to 1.684)
Hexadecenedioate (C16:1-DC)	Diabetic Nephropathy	4	MR Egger	0.3082	0.894 (0.760 to 1.052)
		4	Weighted median	**0.0284**	0.928 (0.867 to 0.992)
		4	Inverse variance weighted	**0.0384**	0.936 (0.880 to 0.996)
		4	Simple mode	0.1713	0.910 (0.821 to 1.009)
		4	Weighted mode	0.1392	0.927 (0.861 to 0.998)
Hydroxypalmitoyl sphingomyelin (d18:1/16:0(OH))	Diabetic Nephropathy	3	MR Egger	0.3830	1.240 (0.928 to 1.655)
		3	Weighted median	**0.0222**	1.173 (1.023 to 1.345)
		3	Inverse variance weighted	**0.0149**	1.174 (1.032 to 1.336)
		3	Simple mode	0.2748	1.167 (0.952 to 1.431)
		3	Weighted mode	0.1657	1.172 (1.013 to 1.355)
Linoleoyl-arachidonoyl-glycerol (18:2/20:4) [1]	Diabetic Nephropathy	3	MR Egger	0.6351	0.860 (0.544 to 1.359)
		3	Weighted median	0.0577	0.902 (0.811 to 1.003)
		3	Inverse variance weighted	**0.0498**	0.902 (0.813 to 1.000)
		3	Simple mode	0.2770	0.899 (0.782 to 1.035)
		3	Weighted mode	0.2282	0.900 (0.798 to 1.015)
Metabolonic lactone sulfate	Diabetic Nephropathy	9	MR Egger	**0.0243**	0.875 (0.799 to 0.959)
		9	Weighted median	**0.0074**	0.915 (0.857 to 0.976)
		9	Inverse variance weighted	**0.0111**	0.931 (0.881 to 0.984)
		9	Simple mode	0.0738	0.901 (0.816 to 0.995)
		9	Weighted mode	**0.0272**	0.911 (0.851 to 0.975)
N-stearoyl-sphingosine (d18:1 to 18:0) to N-palmitoyl-sphinganine (d18:0 to 16:0)	Diabetic Nephropathy	5	MR Egger	0.5723	0.851 (0.517 to 1.402)
		5	Weighted median	0.0917	0.859 (0.720 to 1.025)
		5	Inverse variance weighted	**0.0321**	0.848 (0.730 to 0.986)
		5	Simple mode	0.2590	0.858 (0.682 to 1.078)
		5	Weighted mode	0.2209	0.858 (0.697 to 1.056)
N2-acetyl,N6,N6-dimethyllysine	Diabetic Nephropathy	6	MR Egger	0.7460	1.028 (0.881 to 1.198)
		6	Weighted median	**0.0424**	1.063 (1.002 to 1.127)
		6	Inverse variance weighted	**0.0412**	1.053 (1.002 to 1.107)
		6	Simple mode	0.2655	1.063 (0.966 to 1.169)
		6	Weighted mode	0.1511	1.050 (0.992 to 1.112)
Phosphate to linoleoyl-arachidonoyl-glycerol (18:2 to 20:4) [1]	Diabetic Nephropathy	3	MR Egger	0.8276	1.068 (0.671 to 1.700)
		3	Weighted median	**0.0348**	1.135 (1.009 to 1.276)
		3	Inverse variance weighted	**0.0255**	1.136 (1.016 to 1.271)
		3	Simple mode	0.2155	1.160 (0.986 to 1.364)
		3	Weighted mode	0.2102	1.129 (0.991 to 1.286)
Retinol (Vitamin A) to linoleoyl-arachidonoyl-glycerol (18:2 to 20:4) [1]	Diabetic Nephropathy	4	MR Egger	0.4328	1.235 (0.808 to 1.887)
		4	Weighted median	**0.0403**	1.139 (1.006 to 1.290)
		4	Inverse variance weighted	**0.0240**	1.136 (1.017 to 1.269)
		4	Simple mode	0.1904	1.157 (0.977 to 1.372)
		4	Weighted mode	0.1622	1.141 (0.992 to 1.311)
Retinol (Vitamin A) to linoleoyl-arachidonoyl-glycerol (18:2 to 20:4) [2]	Diabetic Nephropathy	3	MR Egger	0.7271	1.111 (0.707 to 1.746)
		3	Weighted median	**0.0173**	1.151 (1.025 to 1.293)
		3	Inverse variance weighted	**0.0072**	1.160 (1.041 to 1.293)
		3	Simple mode	0.2226	1.152 (0.983 to 1.350)
		3	Weighted mode	0.1467	1.151 (1.022 to 1.296)
Sphingomyelin (d18:2/21:0, d16:2/23:0)	Diabetic Nephropathy	4	MR Egger	0.7323	0.917 (0.594 to 1.415)
		4	Weighted median	0.1561	0.843 (0.666 to 1.067)
		4	Inverse variance weighted	**0.0438**	0.819 (0.674 to 0.994)
		4	Simple mode	0.4279	0.861 (0.624 to 1.187)
		4	Weighted mode	0.3338	0.849 (0.643 to 1.122)
Tetradecanedioate (C14-DC)	Diabetic Nephropathy	6	MR Egger	0.0998	0.851 (0.733 to 0.987)
		6	Weighted median	**0.0303**	0.907 (0.830 to 0.991)
		6	Inverse variance weighted	**0.0367**	0.922 (0.854 to 0.995)
		6	Simple mode	0.1388	0.892 (0.785 to 1.013)
		6	Weighted mode	0.1027	0.905 (0.820 to 0.998)
Vanillic acid glycine	Diabetic Nephropathy	4	MR Egger	0.5811	0.855 (0.533 to 1.370)
		4	Weighted median	0.2503	0.881 (0.711 to 1.093)
		4	Inverse variance weighted	**0.0321**	0.822 (0.687 to 0.983)
		4	Simple mode	0.5661	0.902 (0.659 to 1.235)
		4	Weighted mode	0.5374	0.905 (0.683 to 1.200)
X-18922	Diabetic Nephropathy	6	MR Egger	0.5821	0.918 (0.692 to 1.216)
		6	Weighted median	**0.0395**	0.869 (0.761 to 0.993)
		6	Inverse variance weighted	**0.0382**	0.891 (0.799 to 0.994)
		6	Simple mode	0.2810	0.879 (0.713 to 1.084)
		6	Weighted mode	0.1517	0.864 (0.728 to 1.024)
X-25371	Diabetic Nephropathy	4	MR Egger	0.9961	0.999 (0.740 to 1.349)
		4	Weighted median	**0.0405**	0.859 (0.743 to 0.993)
		4	Inverse variance weighted	**0.0116**	0.845 (0.741 to 0.963)
		4	Simple mode	0.6068	0.938 (0.754 to 1.168)
		4	Weighted mode	0.1366	0.849 (0.724 to 0.995)
X-26109	Diabetic Nephropathy	6	MR Egger	0.0833	0.911 (0.841 to 0.986)
		6	Weighted median	**0.0279**	0.930 (0.872 to 0.992)
		6	Inverse variance weighted	**0.0205**	0.933 (0.879 to 0.989)
		6	Simple mode	0.3901	0.936 (0.814 to 1.075)
		6	Weighted mode	0.0797	0.927 (0.867 to 0.992)
X-26111	Diabetic Nephropathy	5	MR Egger	0.2335	0.791 (0.582 to 1.077)
		5	Weighted median	**0.0219**	0.857 (0.751 to 0.978)
		5	Inverse variance weighted	**0.0283**	0.875 (0.777 to 0.986)
		5	Simple mode	0.1860	0.856 (0.707 to 1.036)
		5	Weighted mode	0.1328	0.857 (0.729 to 1.006)

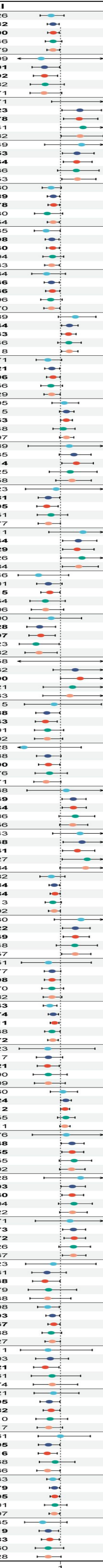

(E)

exposure	outcome	nsnp	method	pval	OR(95% CI)
Selenium	Diabetic Nephropathy	6	MR Egger	0.5433	0.939 (0.781 to 1.130)
		6	Weighted median	0.1319	0.919 (0.824 to 1.026)
		6	Inverse variance weighted	**0.0305**	0.908 (0.833 to 0.991)
		6	Simple mode	0.2996	0.915 (0.788 to 1.063)
		6	Weighted mode	0.2518	0.918 (0.807 to 1.045)

(F)

exposure	outcome	nsnp	method	pval	OR(95% CI)
Diabetic Nephropathy	CD45RA− CD28− CD8br %T cell	3	IVW	0.9219	0.962 (0.447 to 2.072)
Diabetic Nephropathy	CD3 on resting Treg	3	IVW	0.6311	1.046 (0.872 to 1.254)

(G)

exposure	outcome	nsnp	method	pval	OR(95% CI)
Diabetic Nephropathy	Unclassified CAG−145	3	IVW	0.0672	0.954 (0.907 to 1.003)
Diabetic Nephropathy	Family Enterobacteriaceae	3	IVW	0.6808	0.981 (0.894 to 1.076)
Diabetic Nephropathy	Genus Citrobacter A	3	IVW	0.4070	0.982 (0.939 to 1.026)
Diabetic Nephropathy	Family Enterobacteriaceae	3	IVW	0.9856	0.999 (0.907 to 1.100)
Diabetic Nephropathy	Genus Desulfovibrionia	3	IVW	0.9898	1.001 (0.920 to 1.088)
Diabetic Nephropathy	Genus Leuconostoc	3	IVW	0.6890	1.012 (0.953 to 1.076)
Diabetic Nephropathy	Genus Syntrophorhabdia	3	IVW	**0.0258**	1.022 (1.003 to 1.042)

(H)

exposure	outcome	nsnp	method	pval	OR(95% CI)
Diabetic Nephropathy	Phosphatidylcholine (16:0_18:2)	3	IVW	0.4407	0.948 (0.827 to 1.086)
Diabetic Nephropathy	Phosphatidylethanolamine (16:0_18:2)	3	IVW	0.3084	0.951 (0.863 to 1.048)
Diabetic Nephropathy	Phosphatidylcholine (15:0_18:2)	3	IVW	0.5918	0.958 (0.817 to 1.122)
Diabetic Nephropathy	Sterol ester (27:1/18:3)	3	IVW	0.5027	0.972 (0.893 to 1.057)
Diabetic Nephropathy	Phosphatidylcholine (17:0_20:4)	3	IVW	0.9514	0.997 (0.917 to 1.085)
Diabetic Nephropathy	Phosphatidylcholine (16:0_20:4)	3	IVW	0.9944	1.000 (0.902 to 1.107)
Diabetic Nephropathy	Phosphatidylinositol (18:1_18:2)	3	IVW	0.9180	1.005 (0.920 to 1.097)
Diabetic Nephropathy	Phosphatidylcholine (18:1_18:2)	3	IVW	0.8501	1.008 (0.924 to 1.100)
Diabetic Nephropathy	Phosphatidylcholine (O−16:1_20:4)	3	IVW	0.7025	1.028 (0.892 to 1.185)
Diabetic Nephropathy	Phosphatidylcholine (18:1_18:1)	3	IVW	0.5223	1.029 (0.942 to 1.124)
Diabetic Nephropathy	Phosphatidylcholine (18:1_20:4)	3	IVW	0.3140	1.044 (0.960 to 1.135)

(I)

exposure	outcome	nsnp	method	pval	OR(95% CI)
Diabetic Nephropathy	1−palmitoyl−2−linoleoyl−GPI (16:0/18:2)	3	IVW	0.0897	0.909 (0.813 to 1.015)
Diabetic Nephropathy	Linoleoyl−arachidonoyl−glycerol (18:2/20:4) [1]	3	IVW	0.1269	0.937 (0.862 to 1.019)
Diabetic Nephropathy	1−oleoyl−2−linoleoyl−GPE (18:1/18:2)	3	IVW	0.3858	0.948 (0.841 to 1.069)
Diabetic Nephropathy	Aspartate to N−acetylglucosamine to N−acetylgalactosamine	3	IVW	0.2163	0.952 (0.880 to 1.029)
Diabetic Nephropathy	1−palmitoyl−2−linoleoyl−GPE (16:0/18:2)	3	IVW	0.4318	0.954 (0.847 to 1.074)
Diabetic Nephropathy	1,2−dilinoleoyl−GPE (18:2/18:2)	3	IVW	0.4041	0.957 (0.864 to 1.061)
Diabetic Nephropathy	Vanillic acid glycine	3	IVW	0.3196	0.958 (0.880 to 1.043)
Diabetic Nephropathy	1−stearoyl−2−linoleoyl−gpc (18:0/18:2)	3	IVW	0.3281	0.959 (0.881 to 1.043)
Diabetic Nephropathy	Sphingomyelin (d18:2/21:0, d16:2/23:0)	3	IVW	0.9230	0.997 (0.932 to 1.065)
Diabetic Nephropathy	1−linoleoyl−GPG (18:2)	3	IVW	0.9545	0.997 (0.892 to 1.113)
Diabetic Nephropathy	1−stearoyl−2−docosahexaenoyl−gpc (18:0/22:6)	3	IVW	0.9701	0.998 (0.900 to 1.107)
Diabetic Nephropathy	Dodecadienoate (12:2)	3	IVW	0.9586	1.002 (0.927 to 1.084)
Diabetic Nephropathy	Hexadecenedioate (C16:1−DC)	3	IVW	0.8542	1.008 (0.923 to 1.102)
Diabetic Nephropathy	Caffeine to paraxanthine	3	IVW	0.8121	1.010 (0.930 to 1.097)
Diabetic Nephropathy	1−arachidonoyl−GPE (20:4n6)	3	IVW	0.7636	1.012 (0.934 to 1.097)
Diabetic Nephropathy	N2−acetyl,N6,N6−dimethyllysine	3	IVW	0.7342	1.014 (0.935 to 1.101)
Diabetic Nephropathy	N−stearoyl−sphingosine (d18:1 to 18:0) to N−palmitoyl−sphinganine (d18:0 to 16:0)	3	IVW	0.8354	1.016 (0.877 to 1.176)
Diabetic Nephropathy	1−(1−enyl−palmitoyl)−GPC (p−16:0)	3	IVW	0.7014	1.017 (0.932 to 1.111)
Diabetic Nephropathy	Tetradecanedioate (C14−DC)	3	IVW	0.6579	1.018 (0.940 to 1.102)
Diabetic Nephropathy	X−26111	3	IVW	0.6497	1.019 (0.940 to 1.105)
Diabetic Nephropathy	Hydroxypalmitoyl sphingomyelin (d18:1/16:0(OH))	3	IVW	0.6285	1.020 (0.943 to 1.103)
Diabetic Nephropathy	1−arachidonoyl−gpc (20:4n6)	3	IVW	0.4744	1.030 (0.950 to 1.115)
Diabetic Nephropathy	21−hydroxypregnenolone disulfate	3	IVW	0.4552	1.032 (0.950 to 1.122)
Diabetic Nephropathy	Retinol (Vitamin A) to linoleoyl−arachidonoyl−glycerol (18:2 to 20:4) [1]	3	IVW	0.4220	1.035 (0.952 to 1.125)
Diabetic Nephropathy	Retinol (Vitamin A) to linoleoyl−arachidonoyl−glycerol (18:2 to 20:4) [2]	3	IVW	0.3862	1.038 (0.955 to 1.128)
Diabetic Nephropathy	X−25371	3	IVW	0.3536	1.038 (0.959 to 1.125)
Diabetic Nephropathy	Cholesterol to linoleoyl−arachidonoyl−glycerol (18:2 to 20:4) [1]	3	IVW	0.3171	1.043 (0.960 to 1.133)
Diabetic Nephropathy	X−26109	3	IVW	0.4459	1.044 (0.935 to 1.164)
Diabetic Nephropathy	1−(1−enyl−stearoyl)−2−linoleoyl−GPE (p−18:0/18:2)	3	IVW	0.2950	1.044 (0.963 to 1.130)
Diabetic Nephropathy	1−oleoyl−GPG (18:1)	3	IVW	0.2286	1.055 (0.967 to 1.152)
Diabetic Nephropathy	Phosphate to linoleoyl−arachidonoyl−glycerol (18:2 to 20:4) [1]	3	IVW	0.1500	1.063 (0.978 to 1.155)
Diabetic Nephropathy	5−methylthioadenosine (MTA) to phosphate	3	IVW	0.2671	1.065 (0.953 to 1.190)
Diabetic Nephropathy	Metabolonic lactone sulfate	3	IVW	0.3620	1.066 (0.929 to 1.223)
Diabetic Nephropathy	5,6−dihydrouridine	3	IVW	0.0890	1.091 (0.987 to 1.205)
Diabetic Nephropathy	1−(1−enyl−palmitoyl)−2−arachidonoyl−gpc (p−16:0/20:4)	3	IVW	**0.0121**	1.108 (1.023 to 1.200)
Diabetic Nephropathy	X−18922	3	IVW	**0.0085**	1.114 (1.028 to 1.208)

0 0.5 1 1.5 2 2.5 3

(J)

exposure	outcome	nsnp	method	pval	OR(95% CI)
Diabetic Nephropathy	Selenium	2	IVW	0.4411	1.062 (0.912 to 1.236)

0 0.5 1 1.5 2 2.5 3

Fig. (S1). MR analyses for diabetic nephropathy across multiple traits. Forward MR sensitivity analyses using five methods (MR-Egger, weighted median, IVW, simple mode, and weighted mode) are shown in panels A-E, and reverse MR analyses using the IVW method are shown in panels F-J. The five traits are ordered as follows: immune cells, gut microbiota, plasma lipidome, plasma metabolites, and micronutrients. (**A-E**) Forward MR results for each trait. (**F-J**) Reverse MR results for each trait.

(A)

exposure	outcome	nsnp	method	pval	OR(95% CI)
CD20 on IgD+ CD38dim B cell	Diabetic Retinopathy	7	MR Egger	0.5442	1.165 (0.735 to 1.845)
		7	Weighted median	0.0987	1.067 (0.988 to 1.154)
		7	Inverse variance weighted	**0.0043**	1.115 (1.035 to 1.202)
		7	Simple mode	0.3078	1.062 (0.955 to 1.181)
		7	Weighted mode	0.2662	1.062 (0.965 to 1.169)
CD25 on IgD− CD38− B cell	Diabetic Retinopathy	3	MR Egger	0.2488	1.443 (1.073 to 1.940)
		3	Weighted median	**0.0026**	1.146 (1.049 to 1.253)
		3	Inverse variance weighted	**0.0023**	1.148 (1.050 to 1.254)
		3	Simple mode	0.1424	1.213 (1.033 to 1.425)
		3	Weighted mode	0.0833	1.173 (1.065 to 1.292)
CD27 on CD24+ CD27+ B cell	Diabetic Retinopathy	11	MR Egger	0.9565	0.998 (0.926 to 1.075)
		11	Weighted median	0.0872	0.961 (0.919 to 1.006)
		11	Inverse variance weighted	**0.0038**	0.947 (0.913 to 0.983)
		11	Simple mode	0.1038	0.924 (0.848 to 1.008)
		11	Weighted mode	0.2015	0.968 (0.923 to 1.014)
CD27 on IgD− CD38dim B cell	Diabetic Retinopathy	9	MR Egger	0.9932	1.000 (0.932 to 1.072)
		9	Weighted median	0.0779	0.964 (0.926 to 1.004)
		9	Inverse variance weighted	**0.0107**	0.954 (0.921 to 0.989)
		9	Simple mode	0.1362	0.930 (0.853 to 1.013)
		9	Weighted mode	0.3441	0.977 (0.933 to 1.023)
CD27 on sw mem B cell	Diabetic Retinopathy	10	MR Egger	0.7390	0.985 (0.905 to 1.073)
		10	Weighted median	0.0528	0.959 (0.919 to 1.001)
		10	Inverse variance weighted	**0.0043**	0.951 (0.918 to 0.984)
		10	Simple mode	0.1470	0.942 (0.874 to 1.014)
		10	Weighted mode	0.2978	0.973 (0.927 to 1.021)
CD28 on CD28+ CD45RA+ CD8br Treg	Diabetic Retinopathy	3	MR Egger	0.3054	1.350 (0.994 to 1.833)
		3	Weighted median	**0.0026**	1.104 (1.035 to 1.178)
		3	Inverse variance weighted	**0.0038**	1.098 (1.031 to 1.170)
		3	Simple mode	0.2474	1.092 (0.981 to 1.215)
		3	Weighted mode	0.0851	1.106 (1.040 to 1.177)
CD45RA+ CD28− CD8br %T cell	Diabetic Retinopathy	67	MR Egger	0.1757	1.000 (1.000 to 1.001)
		67	Weighted median	0.4588	1.000 (1.000 to 1.001)
		67	Inverse variance weighted	**0.0343**	1.000 (1.000 to 1.001)
		67	Simple mode	0.6777	1.000 (0.999 to 1.001)
		67	Weighted mode	0.5081	1.000 (1.000 to 1.001)
CD45RA+ CD28− CD8br AC Treg	Diabetic Retinopathy	426	MR Egger	**0.0047**	1.000 (1.000 to 1.000)
		426	Weighted median	0.2642	1.000 (1.000 to 1.000)
		426	Inverse variance weighted	**0.0225**	1.000 (1.000 to 1.000)
		426	Simple mode	0.3497	1.000 (1.000 to 1.000)
		426	Weighted mode	0.1003	1.000 (1.000 to 1.000)
CD80 on CD62L+ myeloid DC	Diabetic Retinopathy	3	MR Egger	0.4350	1.132 (0.929 to 1.381)
		3	Weighted median	0.0855	1.086 (0.988 to 1.194)
		3	Inverse variance weighted	**0.0233**	1.095 (1.012 to 1.185)
		3	Simple mode	0.3913	1.071 (0.946 to 1.213)
		3	Weighted mode	0.3566	1.074 (0.955 to 1.208)
CD80 on monocyte cDC	Diabetic Retinopathy	6	MR Egger	0.0970	1.078 (1.007 to 1.154)
		6	Weighted median	**0.0084**	1.073 (1.018 to 1.131)
		6	Inverse variance weighted	**0.0106**	1.056 (1.013 to 1.102)
		6	Simple mode	0.3244	1.044 (0.966 to 1.128)
		6	Weighted mode	0.0913	1.069 (1.004 to 1.138)
CD80 on myeloid DC	Diabetic Retinopathy	4	MR Egger	0.3534	1.127 (0.927 to 1.370)
		4	Weighted median	0.0993	1.074 (0.987 to 1.170)
		4	Inverse variance weighted	**0.0225**	1.086 (1.012 to 1.165)
		4	Simple mode	0.3778	1.065 (0.945 to 1.201)
		4	Weighted mode	0.4249	1.056 (0.940 to 1.187)
CX3CR1 on CD14+ CD16+ monocyte	Diabetic Retinopathy	3	MR Egger	0.5268	0.943 (0.833 to 1.068)
		3	Weighted median	**0.0169**	0.940 (0.894 to 0.989)
		3	Inverse variance weighted	**0.0169**	0.941 (0.895 to 0.989)
		3	Simple mode	0.2464	0.937 (0.866 to 1.014)
		3	Weighted mode	0.1629	0.939 (0.888 to 0.994)
CX3CR1 on CD14+ CD16− monocyte	Diabetic Retinopathy	3	MR Egger	0.5405	0.904 (0.722 to 1.132)
		3	Weighted median	**0.0331**	0.948 (0.903 to 0.996)
		3	Inverse variance weighted	**0.0195**	0.947 (0.904 to 0.991)
		3	Simple mode	0.3910	0.963 (0.900 to 1.031)
		3	Weighted mode	0.1593	0.938 (0.886 to 0.993)
CX3CR1 on monocyte	Diabetic Retinopathy	3	MR Egger	0.5627	0.915 (0.740 to 1.131)
		3	Weighted median	**0.0325**	0.942 (0.892 to 0.995)
		3	Inverse variance weighted	**0.0195**	0.942 (0.895 to 0.990)
		3	Simple mode	0.3916	0.959 (0.890 to 1.034)
		3	Weighted mode	0.1397	0.932 (0.880 to 0.988)
IgD on IgD+ CD38br B cell	Diabetic Retinopathy	6	MR Egger	0.2481	0.956 (0.896 to 1.020)
		6	Weighted median	**0.0278**	0.962 (0.929 to 0.996)
		6	Inverse variance weighted	**0.0259**	0.964 (0.933 to 0.996)
		6	Simple mode	0.2530	0.965 (0.914 to 1.019)
		6	Weighted mode	0.1165	0.962 (0.924 to 1.001)
IgD on transitional B cell	Diabetic Retinopathy	5	MR Egger	0.3647	0.950 (0.864 to 1.044)
		5	Weighted median	**0.0356**	0.953 (0.911 to 0.997)
		5	Inverse variance weighted	**0.0312**	0.955 (0.915 to 0.996)
		5	Simple mode	0.3938	0.964 (0.895 to 1.039)
		5	Weighted mode	0.1385	0.953 (0.906 to 1.003)

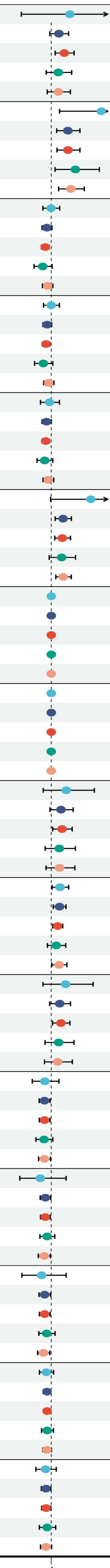

(B)

exposure	outcome	nsnp	method	pval	OR(95% CI)
Order Actinomycetales	Diabetic Retinopathy	4	MR Egger	0.4905	0.778 (0.433 to 1.400)
		4	Weighted median	0.1748	0.871 (0.714 to 1.063)
		4	Inverse variance weighted	**0.0460**	0.834 (0.699 to 0.997)
		4	Simple mode	0.4030	0.856 (0.625 to 1.172)
		4	Weighted mode	0.3209	0.874 (0.700 to 1.092)
Genus An181	Diabetic Retinopathy	6	MR Egger	0.8114	1.084 (0.583 to 2.017)
		6	Weighted median	0.0835	1.339 (0.962 to 1.865)
		6	Inverse variance weighted	**0.0098**	1.393 (1.083 to 1.791)
		6	Simple mode	0.3003	1.347 (0.812 to 2.234)
		6	Weighted mode	0.3399	1.330 (0.783 to 2.258)
Unclassified Bacilli A	Diabetic Retinopathy	8	MR Egger	0.1128	24.512 (0.836 to 718.692)
		8	Weighted median	**0.0109**	7.266 (1.578 to 33.451)
		8	Inverse variance weighted	**0.0032**	7.099 (1.930 to 26.104)
		8	Simple mode	0.1082	10.570 (0.859 to 130.142)
		8	Weighted mode	0.1259	9.858 (0.746 to 130.247)
Species Bacteroides stercoris	Diabetic Retinopathy	7	MR Egger	0.5219	0.897 (0.659 to 1.222)
		7	Weighted median	0.0957	0.889 (0.773 to 1.021)
		7	Inverse variance weighted	**0.0312**	0.877 (0.778 to 0.988)
		7	Simple mode	0.3497	0.892 (0.715 to 1.112)
		7	Weighted mode	0.3830	0.891 (0.700 to 1.133)
Family Bifidobacteriaceae	Diabetic Retinopathy	8	MR Egger	0.4713	0.889 (0.659 to 1.199)
		8	Weighted median	**0.0315**	0.858 (0.746 to 0.987)
		8	Inverse variance weighted	**0.0054**	0.855 (0.766 to 0.955)
		8	Simple mode	**0.0475**	0.737 (0.574 to 0.946)
		8	Weighted mode	0.1597	0.879 (0.749 to 1.032)
Species Blautia hansenii	Diabetic Retinopathy	11	MR Egger	0.5241	1.115 (0.809 to 1.537)
		11	Weighted median	**0.0306**	1.260 (1.022 to 1.555)
		11	Inverse variance weighted	**0.0167**	1.215 (1.036 to 1.425)
		11	Simple mode	0.1613	1.308 (0.924 to 1.852)
		11	Weighted mode	0.1803	1.298 (0.910 to 1.850)
Species CAG−177 sp003538135	Diabetic Retinopathy	8	MR Egger	0.4306	1.134 (0.847 to 1.519)
		8	Weighted median	**0.0196**	1.232 (1.034 to 1.468)
		8	Inverse variance weighted	**0.0063**	1.200 (1.053 to 1.367)
		8	Simple mode	0.1244	1.245 (0.973 to 1.593)
		8	Weighted mode	0.1781	1.248 (0.934 to 1.667)
Unclassified CAG−475	Diabetic Retinopathy	14	MR Egger	0.3018	0.881 (0.701 to 1.109)
		14	Weighted median	0.2743	0.933 (0.825 to 1.056)
		14	Inverse variance weighted	**0.0480**	0.905 (0.819 to 0.999)
		14	Simple mode	0.7511	0.964 (0.773 to 1.203)
		14	Weighted mode	0.7685	0.965 (0.767 to 1.215)
Species Clostridium M sp001304855	Diabetic Retinopathy	8	MR Egger	0.0960	0.569 (0.325 to 0.996)
		8	Weighted median	0.0921	0.757 (0.548 to 1.047)
		8	Inverse variance weighted	**0.0458**	0.776 (0.605 to 0.995)
		8	Simple mode	0.2465	0.713 (0.422 to 1.204)
		8	Weighted mode	0.2017	0.710 (0.440 to 1.144)
Unclassified Firmicutes A	Diabetic Retinopathy	7	MR Egger	0.1546	5.897 (0.740 to 46.981)
		7	Weighted median	0.0769	2.050 (0.925 to 4.543)
		7	Inverse variance weighted	**0.0333**	1.915 (1.053 to 3.482)
		7	Simple mode	0.2395	2.330 (0.654 to 8.292)
		7	Weighted mode	0.1582	2.850 (0.797 to 10.184)
Unclassified Geobacter C	Diabetic Retinopathy	6	MR Egger	0.7225	1.250 (0.397 to 3.929)
		6	Weighted median	0.0568	1.776 (0.984 to 3.208)
		6	Inverse variance weighted	**0.0251**	1.702 (1.069 to 2.711)
		6	Simple mode	0.2395	1.848 (0.750 to 4.552)
		6	Weighted mode	0.2525	1.805 (0.737 to 4.416)
Genus Holdemania	Diabetic Retinopathy	12	MR Egger	0.9586	1.010 (0.708 to 1.440)
		12	Weighted median	0.0691	1.187 (0.987 to 1.429)
		12	Inverse variance weighted	**0.0236**	1.162 (1.020 to 1.323)
		12	Simple mode	0.1608	1.287 (0.926 to 1.789)
		12	Weighted mode	0.1626	1.262 (0.930 to 1.713)
Species Lachnospira rogosae	Diabetic Retinopathy	10	MR Egger	0.2396	1.223 (0.897 to 1.668)
		10	Weighted median	0.1083	1.126 (0.974 to 1.301)
		10	Inverse variance weighted	**0.0145**	1.138 (1.026 to 1.263)
		10	Simple mode	0.5623	1.070 (0.858 to 1.336)
		10	Weighted mode	0.5669	1.069 (0.859 to 1.330)
Species Pseudomonas aeruginosa	Diabetic Retinopathy	7	MR Egger	0.7019	1.146 (0.594 to 2.211)
		7	Weighted median	0.1206	1.466 (0.904 to 2.376)
		7	Inverse variance weighted	**0.0136**	1.589 (1.100 to 2.297)
		7	Simple mode	0.5018	1.291 (0.641 to 2.602)
		7	Weighted mode	0.5432	1.246 (0.638 to 2.431)

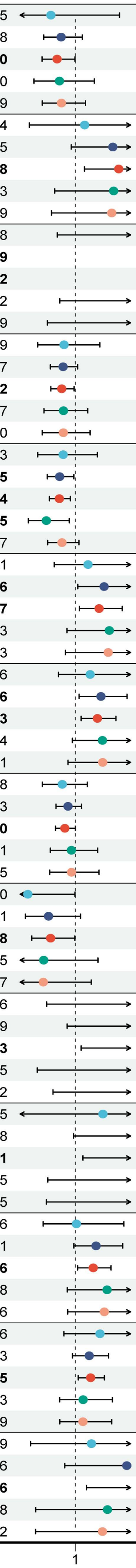

(C)

exposure	outcome	nsnp	method	pval	OR(95% CI)
FGF19	Diabetic Retinopathy	3	MR Egger	0.5592	0.783 (0.439 to 1.396)
		3	Weighted median	**0.0359**	0.863 (0.752 to 0.990)
		3	Inverse variance weighted	**0.0336**	0.874 (0.772 to 0.990)
		3	Simple mode	0.2140	0.844 (0.702 to 1.015)
		3	Weighted mode	0.2025	0.858 (0.731 to 1.007)
FGF21	Diabetic Retinopathy	4	MR Egger	0.4007	0.772 (0.477 to 1.247)
		4	Weighted median	0.0938	0.897 (0.790 to 1.019)
		4	Inverse variance weighted	**0.0247**	0.884 (0.794 to 0.984)
		4	Simple mode	0.2844	0.901 (0.770 to 1.054)
		4	Weighted mode	0.2235	0.896 (0.779 to 1.031)

1

(D)

exposure	outcome	nsnp	method	pval	OR(95% CI)
Diacylglycerol (18:1_18:1)	Diabetic Retinopathy	5	MR Egger	0.2833	0.834 (0.636 to 1.095)
		5	Weighted median	**0.0363**	0.884 (0.788 to 0.992)
		5	Inverse variance weighted	**0.0146**	0.889 (0.810 to 0.977)
		5	Simple mode	0.3722	0.923 (0.790 to 1.079)
		5	Weighted mode	0.1571	0.888 (0.777 to 1.015)
Phosphatidylcholine (18:2_20:4)	Diabetic Retinopathy	4	MR Egger	0.3684	0.718 (0.408 to 1.262)
		4	Weighted median	**0.0277**	0.871 (0.771 to 0.985)
		4	Inverse variance weighted	**0.0387**	0.885 (0.787 to 0.994)
		4	Simple mode	0.1739	0.835 (0.685 to 1.019)
		4	Weighted mode	0.1645	0.867 (0.744 to 1.010)
Phosphatidylethanolamine (16:0_20:4)	Diabetic Retinopathy	5	MR Egger	0.0933	0.873 (0.783 to 0.974)
		5	Weighted median	**0.0077**	0.927 (0.876 to 0.980)
		5	Inverse variance weighted	**0.0044**	0.933 (0.889 to 0.979)
		5	Simple mode	0.0898	0.920 (0.855 to 0.990)
		5	Weighted mode	0.0550	0.927 (0.877 to 0.980)
Phosphatidylethanolamine (18:1_18:1)	Diabetic Retinopathy	6	MR Egger	0.5676	0.885 (0.601 to 1.302)
		6	Weighted median	**0.0079**	0.890 (0.817 to 0.970)
		6	Inverse variance weighted	**0.0487**	0.927 (0.860 to 1.000)
		6	Simple mode	0.1308	0.889 (0.783 to 1.010)
		6	Weighted mode	0.0976	0.890 (0.795 to 0.996)
Phosphatidylinositol (16:0_20:4)	Diabetic Retinopathy	3	MR Egger	0.8115	0.826 (0.241 to 2.829)
		3	Weighted median	0.0760	0.876 (0.758 to 1.014)
		3	Inverse variance weighted	**0.0464**	0.878 (0.772 to 0.998)
		3	Simple mode	0.2554	0.876 (0.743 to 1.033)
		3	Weighted mode	0.2816	0.874 (0.730 to 1.047)
Sphingomyelin (d36:2)	Diabetic Retinopathy	4	MR Egger	0.9848	0.996 (0.720 to 1.379)
		4	Weighted median	**0.0169**	0.874 (0.782 to 0.976)
		4	Inverse variance weighted	**0.0107**	0.887 (0.809 to 0.973)
		4	Simple mode	0.1588	0.858 (0.730 to 1.008)
		4	Weighted mode	0.1373	0.864 (0.750 to 0.996)
Sterol ester (27:1/18:3)	Diabetic Retinopathy	5	MR Egger	0.2063	0.816 (0.637 to 1.046)
		5	Weighted median	0.0950	0.903 (0.800 to 1.018)
		5	Inverse variance weighted	**0.0388**	0.907 (0.827 to 0.995)
		5	Simple mode	0.2580	0.895 (0.758 to 1.056)
		5	Weighted mode	0.1983	0.899 (0.786 to 1.029)
Triacylglycerol (50:3)	Diabetic Retinopathy	3	MR Egger	0.3859	0.613 (0.315 to 1.192)
		3	Weighted median	**0.0259**	0.869 (0.768 to 0.983)
		3	Inverse variance weighted	**0.0063**	0.860 (0.772 to 0.958)
		3	Simple mode	0.2521	0.866 (0.726 to 1.034)
		3	Weighted mode	0.2516	0.878 (0.749 to 1.030)
Triacylglycerol (50:4)	Diabetic Retinopathy	3	MR Egger	0.3724	0.554 (0.257 to 1.193)
		3	Weighted median	**0.0246**	0.868 (0.767 to 0.982)
		3	Inverse variance weighted	**0.0066**	0.863 (0.776 to 0.960)
		3	Simple mode	0.1663	0.838 (0.712 to 0.986)
		3	Weighted mode	0.2534	0.882 (0.756 to 1.030)
Triacylglycerol (50:5)	Diabetic Retinopathy	3	MR Egger	0.3822	0.656 (0.373 to 1.155)
		3	Weighted median	**0.0122**	0.849 (0.747 to 0.965)
		3	Inverse variance weighted	**0.0056**	0.855 (0.765 to 0.955)
		3	Simple mode	0.1264	0.817 (0.698 to 0.955)
		3	Weighted mode	0.1476	0.820 (0.693 to 0.971)
Triacylglycerol (51:3)	Diabetic Retinopathy	6	MR Egger	0.0842	0.730 (0.558 to 0.956)
		6	Weighted median	**0.0326**	0.882 (0.786 to 0.990)
		6	Inverse variance weighted	**0.0050**	0.882 (0.808 to 0.963)
		6	Simple mode	0.1393	0.869 (0.743 to 1.016)
		6	Weighted mode	0.1098	0.877 (0.767 to 1.001)
Triacylglycerol (52:2)	Diabetic Retinopathy	4	MR Egger	0.3071	0.785 (0.554 to 1.113)
		4	Weighted median	**0.0366**	0.877 (0.776 to 0.992)
		4	Inverse variance weighted	**0.0288**	0.887 (0.797 to 0.988)
		4	Simple mode	0.2102	0.867 (0.727 to 1.034)
		4	Weighted mode	0.1336	0.872 (0.765 to 0.994)
Triacylglycerol (52:3)	Diabetic Retinopathy	6	MR Egger	0.2138	0.848 (0.681 to 1.055)
		6	Weighted median	**0.0215**	0.895 (0.814 to 0.984)
		6	Inverse variance weighted	**0.0159**	0.907 (0.838 to 0.982)
		6	Simple mode	0.2028	0.898 (0.778 to 1.037)
		6	Weighted mode	0.1314	0.895 (0.793 to 1.010)
Triacylglycerol (52:4)	Diabetic Retinopathy	7	MR Egger	0.1540	0.822 (0.653 to 1.033)
		7	Weighted median	**0.0280**	0.890 (0.803 to 0.988)
		7	Inverse variance weighted	**0.0032**	0.888 (0.821 to 0.961)
		7	Simple mode	0.1655	0.886 (0.763 to 1.030)
		7	Weighted mode	0.1005	0.890 (0.791 to 1.001)
Triacylglycerol (52:5)	Diabetic Retinopathy	4	MR Egger	0.1632	0.716 (0.529 to 0.969)
		4	Weighted median	**0.0372**	0.888 (0.794 to 0.993)
		4	Inverse variance weighted	**0.0161**	0.890 (0.810 to 0.979)
		4	Simple mode	0.1966	0.879 (0.755 to 1.024)
		4	Weighted mode	0.1390	0.883 (0.782 to 0.997)
Triacylglycerol (52:6)	Diabetic Retinopathy	4	MR Egger	0.2702	0.756 (0.526 to 1.087)
		4	Weighted median	**0.0064**	0.850 (0.756 to 0.955)
		4	Inverse variance weighted	**0.0011**	0.849 (0.770 to 0.937)
		4	Simple mode	0.1296	0.851 (0.731 to 0.991)
		4	Weighted mode	0.1242	0.850 (0.732 to 0.988)
Triacylglycerol (54:6)	Diabetic Retinopathy	3	MR Egger	0.5874	0.871 (0.609 to 1.245)
		3	Weighted median	**0.0296**	0.857 (0.746 to 0.985)
		3	Inverse variance weighted	**0.0105**	0.853 (0.755 to 0.964)
		3	Simple mode	0.2349	0.863 (0.726 to 1.025)
		3	Weighted mode	0.1888	0.859 (0.738 to 1.000)
Triacylglycerol (54:7)	Diabetic Retinopathy	4	MR Egger	0.2561	0.705 (0.456 to 1.090)
		4	Weighted median	**0.0157**	0.845 (0.738 to 0.969)
		4	Inverse variance weighted	**0.0017**	0.832 (0.742 to 0.933)
		4	Simple mode	0.2230	0.865 (0.718 to 1.041)
		4	Weighted mode	0.1963	0.863 (0.724 to 1.028)
Triacylglycerol (56:7)	Diabetic Retinopathy	5	MR Egger	0.1589	0.761 (0.572 to 1.014)
		5	Weighted median	**0.0081**	0.865 (0.778 to 0.963)
		5	Inverse variance weighted	**0.0040**	0.877 (0.803 to 0.959)
		5	Simple mode	0.1355	0.866 (0.745 to 1.007)
		5	Weighted mode	0.1127	0.868 (0.757 to 0.995)
Triacylglycerol (56:8)	Diabetic Retinopathy	5	MR Egger	0.1111	0.650 (0.446 to 0.948)
		5	Weighted median	**0.0118**	0.857 (0.759 to 0.966)
		5	Inverse variance weighted	**0.0101**	0.881 (0.799 to 0.970)
		5	Simple mode	0.1075	0.838 (0.709 to 0.991)
		5	Weighted mode	0.0944	0.843 (0.723 to 0.983)

1

(E)

exposure	outcome	nsnp	method	pval	OR(95% CI)
(2 or 3)−decenoate (10:1n7 or n8)	Diabetic Retinopathy	3	MR Egger	0.4157	1.394 (0.847 to 2.295)
		3	Weighted median	0.0642	1.169 (0.991 to 1.380)
		3	Inverse variance weighted	**0.0215**	1.189 (1.026 to 1.377)
		3	Simple mode	0.2821	1.183 (0.944 to 1.483)
		3	Weighted mode	0.2728	1.154 (0.957 to 1.392)
(S)−a−amino−omega−caprolactam	Diabetic Retinopathy	3	MR Egger	0.3421	0.675 (0.427 to 1.068)
		3	Weighted median	0.1200	0.849 (0.690 to 1.044)
		3	Inverse variance weighted	**0.0223**	0.817 (0.686 to 0.972)
		3	Simple mode	0.3683	0.864 (0.674 to 1.108)
		3	Weighted mode	0.3424	0.862 (0.680 to 1.091)
1−(1−enyl−palmitoyl)−2−palmitoyl−GPC (P−16:0/16:0)	Diabetic Retinopathy	7	MR Egger	0.6278	0.964 (0.838 to 1.109)
		7	Weighted median	**0.0425**	0.935 (0.876 to 0.998)
		7	Inverse variance weighted	**0.0335**	0.933 (0.874 to 0.995)
		7	Simple mode	0.0817	0.863 (0.752 to 0.991)
		7	Weighted mode	0.0989	0.938 (0.879 to 1.000)
1−palmitoleoyl−GPC (16:1)	Diabetic Retinopathy	4	MR Egger	0.2708	0.664 (0.390 to 1.131)
		4	Weighted median	**0.0234**	0.840 (0.722 to 0.977)
		4	Inverse variance weighted	**0.0025**	0.822 (0.724 to 0.934)
		4	Simple mode	0.2360	0.851 (0.687 to 1.054)
		4	Weighted mode	0.2451	0.860 (0.700 to 1.056)
1−palmitoyl−2−docosahexaenoyl−GPE (16:0/22:6)	Diabetic Retinopathy	6	MR Egger	0.5821	0.968 (0.871 to 1.076)
		6	Weighted median	**0.0424**	0.942 (0.888 to 0.998)
		6	Inverse variance weighted	**0.0102**	0.932 (0.883 to 0.983)
		6	Simple mode	0.3530	0.947 (0.852 to 1.051)
		6	Weighted mode	0.1239	0.944 (0.888 to 1.004)
1−palmitoyl−2−linoleoyl−GPI (16:0/18:2)	Diabetic Retinopathy	5	MR Egger	0.4535	1.129 (0.856 to 1.487)
		5	Weighted median	0.1260	1.081 (0.978 to 1.195)
		5	Inverse variance weighted	**0.0401**	1.093 (1.004 to 1.191)
		5	Simple mode	0.3135	1.069 (0.954 to 1.198)
		5	Weighted mode	0.2172	1.081 (0.974 to 1.200)
1−palmitoyl−2−oleoyl−GPI (16:0/18:1)	Diabetic Retinopathy	4	MR Egger	0.6025	1.156 (0.727 to 1.840)
		4	Weighted median	0.0872	1.111 (0.985 to 1.254)
		4	Inverse variance weighted	**0.0352**	1.111 (1.007 to 1.225)
		4	Simple mode	0.5497	1.055 (0.903 to 1.233)
		4	Weighted mode	0.1639	1.138 (0.991 to 1.306)
1−palmitoyl−GPE (16:0)	Diabetic Retinopathy	6	MR Egger	0.4317	0.855 (0.601 to 1.215)
		6	Weighted median	**0.0228**	0.884 (0.794 to 0.983)
		6	Inverse variance weighted	**0.0182**	0.900 (0.824 to 0.982)
		6	Simple mode	0.1580	0.894 (0.784 to 1.020)
		6	Weighted mode	0.1179	0.893 (0.794 to 1.004)
1−stearoyl−2−docosahexaenoyl−GPE (18:0/22:6)	Diabetic Retinopathy	5	MR Egger	0.3752	0.909 (0.759 to 1.088)
		5	Weighted median	**0.0454**	0.922 (0.852 to 0.998)
		5	Inverse variance weighted	**0.0139**	0.915 (0.853 to 0.982)
		5	Simple mode	0.3418	0.936 (0.829 to 1.056)
		5	Weighted mode	0.1442	0.920 (0.841 to 1.007)
1−stearoyl−gpc (18:0)	Diabetic Retinopathy	3	MR Egger	0.7290	0.754 (0.223 to 2.550)
		3	Weighted median	0.1387	0.883 (0.748 to 1.041)
		3	Inverse variance weighted	**0.0195**	0.848 (0.739 to 0.974)
		3	Simple mode	0.3375	0.885 (0.731 to 1.072)
		3	Weighted mode	0.3346	0.885 (0.733 to 1.070)
1−stearoyl−GPE (18:0)	Diabetic Retinopathy	7	MR Egger	0.9948	0.999 (0.787 to 1.268)
		7	Weighted median	**0.0268**	0.900 (0.820 to 0.988)
		7	Inverse variance weighted	**0.0011**	0.881 (0.816 to 0.950)
		7	Simple mode	0.1163	0.888 (0.783 to 1.008)
		7	Weighted mode	0.0764	0.901 (0.819 to 0.991)
16a−hydroxy DHEA 3−sulfate	Diabetic Retinopathy	4	MR Egger	0.2491	1.044 (0.991 to 1.099)
		4	Weighted median	**0.0347**	1.046 (1.003 to 1.091)
		4	Inverse variance weighted	**0.0300**	1.047 (1.004 to 1.091)
		4	Simple mode	0.1196	1.098 (1.009 to 1.195)
		4	Weighted mode	0.1539	1.042 (0.999 to 1.087)
2−stearoyl−GPE (18:0)	Diabetic Retinopathy	3	MR Egger	0.7522	0.476 (0.014 to 16.520)
		3	Weighted median	**0.0116**	0.820 (0.702 to 0.957)
		3	Inverse variance weighted	**0.0032**	0.817 (0.714 to 0.935)
		3	Simple mode	0.1883	0.827 (0.685 to 0.999)
		3	Weighted mode	0.1637	0.824 (0.692 to 0.982)
3−methylcytidine	Diabetic Retinopathy	4	MR Egger	0.4986	1.040 (0.946 to 1.144)
		4	Weighted median	**0.0251**	1.070 (1.009 to 1.135)
		4	Inverse variance weighted	**0.0185**	1.067 (1.011 to 1.126)
		4	Simple mode	0.2517	1.067 (0.976 to 1.166)
		4	Weighted mode	0.1124	1.069 (1.008 to 1.134)
5−hydroxylysine	Diabetic Retinopathy	4	MR Egger	0.3903	1.157 (0.890 to 1.503)
		4	Weighted median	**0.0015**	1.135 (1.050 to 1.227)
		4	Inverse variance weighted	**0.0027**	1.112 (1.038 to 1.192)
		4	Simple mode	0.0907	1.137 (1.027 to 1.260)
		4	Weighted mode	0.0619	1.136 (1.043 to 1.238)
5alpha−androstan−3alpha,17alpha−diol monosulfate	Diabetic Retinopathy	6	MR Egger	0.0633	0.895 (0.821 to 0.975)
		6	Weighted median	**0.0492**	0.946 (0.895 to 1.000)
		6	Inverse variance weighted	**0.0366**	0.953 (0.910 to 0.997)
		6	Simple mode	0.1934	0.942 (0.872 to 1.018)
		6	Weighted mode	0.1346	0.945 (0.888 to 1.006)
5alpha−androstan−3beta,17beta−diol disulfate	Diabetic Retinopathy	5	MR Egger	0.2291	0.934 (0.856 to 1.021)
		5	Weighted median	0.0649	0.939 (0.879 to 1.004)
		5	Inverse variance weighted	**0.0328**	0.936 (0.881 to 0.995)
		5	Simple mode	0.3454	0.937 (0.831 to 1.056)
		5	Weighted mode	0.1570	0.940 (0.876 to 1.008)
Ceramide (d18:1/24:1)	Diabetic Retinopathy	6	MR Egger	0.4756	1.063 (0.913 to 1.238)
		6	Weighted median	0.1445	1.064 (0.979 to 1.157)
		6	Inverse variance weighted	**0.0408**	1.080 (1.003 to 1.162)
		6	Simple mode	0.3733	1.063 (0.940 to 1.202)
		6	Weighted mode	0.2902	1.063 (0.961 to 1.177)
Dodecadienoate (12:2)	Diabetic Retinopathy	5	MR Egger	0.3121	1.132 (0.926 to 1.384)
		5	Weighted median	0.1926	1.083 (0.961 to 1.221)
		5	Inverse variance weighted	**0.0441**	1.113 (1.003 to 1.235)
		5	Simple mode	0.5245	1.066 (0.890 to 1.277)
		5	Weighted mode	0.4480	1.067 (0.917 to 1.243)
Epiandrosterone sulfate	Diabetic Retinopathy	5	MR Egger	0.2357	0.969 (0.930 to 1.010)
		5	Weighted median	**0.0425**	0.966 (0.933 to 0.999)
		5	Inverse variance weighted	**0.0367**	0.965 (0.934 to 0.998)
		5	Simple mode	0.7671	0.985 (0.899 to 1.079)
		5	Weighted mode	0.1368	0.967 (0.933 to 1.002)
Glyco−beta−muricholate	Diabetic Retinopathy	3	MR Egger	0.6425	1.042 (0.917 to 1.182)
		3	Weighted median	0.0544	1.052 (0.999 to 1.108)
		3	Inverse variance weighted	**0.0343**	1.054 (1.004 to 1.106)
		3	Simple mode	0.4210	1.043 (0.961 to 1.131)
		3	Weighted mode	0.2219	1.047 (0.995 to 1.103)
Homocitrulline	Diabetic Retinopathy	4	MR Egger	0.7595	0.964 (0.783 to 1.186)
		4	Weighted median	0.0867	0.928 (0.852 to 1.011)
		4	Inverse variance weighted	**0.0357**	0.919 (0.850 to 0.994)
		4	Simple mode	0.2894	0.925 (0.822 to 1.042)
		4	Weighted mode	0.2388	0.928 (0.841 to 1.025)
Indoleacetylglutamine	Diabetic Retinopathy	4	MR Egger	0.4124	0.915 (0.773 to 1.084)
		4	Weighted median	0.0841	0.935 (0.866 to 1.009)
		4	Inverse variance weighted	**0.0370**	0.931 (0.870 to 0.996)
		4	Simple mode	0.2581	0.938 (0.858 to 1.026)
		4	Weighted mode	0.1836	0.936 (0.868 to 1.009)
Metabolonic lactone sulfate	Diabetic Retinopathy	9	MR Egger	0.0712	0.926 (0.863 to 0.994)
		9	Weighted median	**0.0109**	0.942 (0.899 to 0.986)
		9	Inverse variance weighted	**0.0180**	0.950 (0.911 to 0.991)
		9	Simple mode	0.4222	0.967 (0.893 to 1.046)
		9	Weighted mode	**0.0373**	0.944 (0.902 to 0.988)
N−stearoyl−sphingosine (d18:1 to 18:0) to N−palmitoyl−sphinganine (d18:0 to 16:0)	Diabetic Retinopathy	5	MR Egger	0.5111	0.865 (0.589 to 1.269)
		5	Weighted median	**0.0489**	0.883 (0.780 to 0.999)
		5	Inverse variance weighted	**0.0301**	0.894 (0.809 to 0.989)
		5	Simple mode	0.1726	0.868 (0.734 to 1.026)
		5	Weighted mode	0.1602	0.881 (0.762 to 1.018)
Oleoyl−linoleoyl−glycerol (18:1/18:2) [2]	Diabetic Retinopathy	7	MR Egger	0.1875	0.849 (0.687 to 1.048)
		7	Weighted median	0.0575	0.898 (0.803 to 1.003)
		7	Inverse variance weighted	**0.0102**	0.895 (0.823 to 0.974)
		7	Simple mode	0.4317	0.931 (0.789 to 1.099)
		7	Weighted mode	0.1701	0.898 (0.784 to 1.028)
Phosphate to oleoyl−linoleoyl−glycerol (18:1 to 18:2) [2]	Diabetic Retinopathy	6	MR Egger	0.2504	1.158 (0.935 to 1.434)
		6	Weighted median	**0.0444**	1.126 (1.003 to 1.264)
		6	Inverse variance weighted	**0.0221**	1.113 (1.015 to 1.219)
		6	Simple mode	0.2037	1.145 (0.955 to 1.373)
		6	Weighted mode	0.1190	1.140 (0.994 to 1.307)
Sphingomyelin (d18:1/18:1, d18:2/18:0)	Diabetic Retinopathy	4	MR Egger	0.5974	0.899 (0.643 to 1.257)
		4	Weighted median	**0.0422**	0.883 (0.783 to 0.996)
		4	Inverse variance weighted	**0.0354**	0.894 (0.805 to 0.992)
		4	Simple mode	0.1942	0.877 (0.752 to 1.023)
		4	Weighted mode	0.1820	0.881 (0.764 to 1.017)
Sphingomyelin (d18:1/20:1, d18:2/20:0)	Diabetic Retinopathy	4	MR Egger	0.5776	0.931 (0.751 to 1.153)
		4	Weighted median	**0.0369**	0.904 (0.822 to 0.994)
		4	Inverse variance weighted	**0.0331**	0.908 (0.830 to 0.992)
		4	Simple mode	0.1808	0.891 (0.782 to 1.015)
		4	Weighted mode	0.1727	0.904 (0.809 to 1.010)
X−24544	Diabetic Retinopathy	4	MR Egger	0.2210	1.100 (0.989 to 1.224)
		4	Weighted median	**0.0021**	1.094 (1.033 to 1.158)
		4	Inverse variance weighted	**0.0043**	1.099 (1.030 to 1.173)
		4	Simple mode	0.3373	1.073 (0.951 to 1.211)
		4	Weighted mode	0.0673	1.093 (1.027 to 1.164)
X−26109	Diabetic Retinopathy	6	MR Egger	0.1473	0.939 (0.877 to 1.006)
		6	Weighted median	**0.0046**	0.942 (0.904 to 0.982)
		6	Inverse variance weighted	**0.0068**	0.940 (0.898 to 0.983)
		6	Simple mode	0.2666	0.950 (0.878 to 1.029)
		6	Weighted mode	**0.0386**	0.942 (0.903 to 0.982)

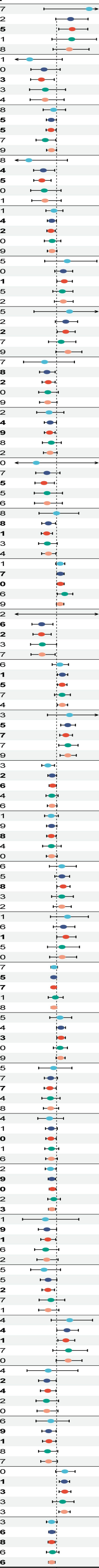

(F)

exposure	outcome	nsnp	method	pval	OR(95% CI)
Diabetic Retinopathy	CD45RA+ CD28− CD8br AC Treg	11	IVW	0.9710	0.641 (0.000 to 16745340049.000)
Diabetic Retinopathy	CD80 on myeloid DC	11	IVW	0.1117	0.897 (0.785 to 1.025)
Diabetic Retinopathy	CD28 on CD28+ CD45RA+ CD8br Treg	11	IVW	0.1567	0.906 (0.790 to 1.039)
Diabetic Retinopathy	CD80 on CD62L+ myeloid DC	11	IVW	0.2812	0.929 (0.813 to 1.062)
Diabetic Retinopathy	CD25 on IgD− CD38− B cell	11	IVW	0.3742	0.947 (0.839 to 1.068)
Diabetic Retinopathy	CD27 on sw mem B cell	11	IVW	0.4960	0.958 (0.847 to 1.084)
Diabetic Retinopathy	CD27 on CD24+ CD27+ B cell	11	IVW	0.7475	0.980 (0.867 to 1.108)
Diabetic Retinopathy	CX3CR1 on CD14+ CD16− monocyte	11	IVW	0.8817	0.990 (0.867 to 1.130)
Diabetic Retinopathy	CX3CR1 on monocyte	11	IVW	0.9151	1.008 (0.864 to 1.177)
Diabetic Retinopathy	CD80 on monocyte cDC	11	IVW	0.8435	1.014 (0.885 to 1.161)
Diabetic Retinopathy	IgD on transitional B cell	11	IVW	0.7941	1.021 (0.876 to 1.189)
Diabetic Retinopathy	CD27 on IgD− CD38dim B cell	11	IVW	0.7515	1.021 (0.898 to 1.162)
Diabetic Retinopathy	CD20 on IgD+ CD38dim B cell	11	IVW	0.5291	1.040 (0.921 to 1.174)
Diabetic Retinopathy	CX3CR1 on CD14+ CD16+ monocyte	11	IVW	0.4933	1.054 (0.907 to 1.224)
Diabetic Retinopathy	IgD on IgD+ CD38br B cell	11	IVW	0.4920	1.056 (0.903 to 1.235)
Diabetic Retinopathy	CD45RA+ CD28− CD8br %T cell	11	IVW	0.7924	1.106 (0.524 to 2.335)

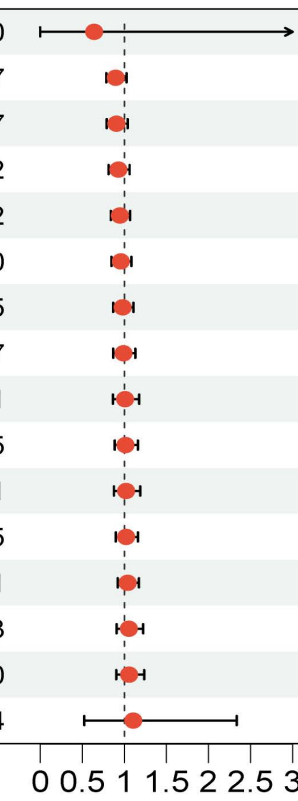

(G)

exposure	outcome	nsnp	method	pval	OR(95% CI)
Diabetic Retinopathy	Species Lachnospira rogosae	12	IVW	**0.0204**	0.938 (0.889 to 0.990)
Diabetic Retinopathy	Species Bacteroides stercoris	12	IVW	0.1392	0.951 (0.890 to 1.016)
Diabetic Retinopathy	Species CAG−177 sp003538135	12	IVW	0.0513	0.952 (0.905 to 1.000)
Diabetic Retinopathy	Unclassified CAG−475	12	IVW	0.4718	0.978 (0.920 to 1.040)
Diabetic Retinopathy	Genus Holdemania	12	IVW	0.4295	0.983 (0.941 to 1.026)
Diabetic Retinopathy	Species Clostridium M sp001304855	12	IVW	0.1992	0.983 (0.958 to 1.009)
Diabetic Retinopathy	Species Blautia hansenii	12	IVW	0.6942	0.992 (0.955 to 1.031)
Diabetic Retinopathy	Unclassified Bacilli A	12	IVW	0.9282	1.000 (0.992 to 1.007)
Diabetic Retinopathy	Unclassified Firmicutes A	12	IVW	0.2745	1.008 (0.994 to 1.021)
Diabetic Retinopathy	Unclassified Geobacter C	12	IVW	0.3166	1.008 (0.992 to 1.024)
Diabetic Retinopathy	Species Pseudomonas aeruginosa	12	IVW	0.0610	1.018 (0.999 to 1.037)
Diabetic Retinopathy	Order Actinomycetales	12	IVW	0.3190	1.019 (0.982 to 1.056)
Diabetic Retinopathy	Family Bifidobacteriaceae	12	IVW	0.2081	1.033 (0.982 to 1.086)
Diabetic Retinopathy	Genus An181	12	IVW	**0.0130**	1.037 (1.008 to 1.068)

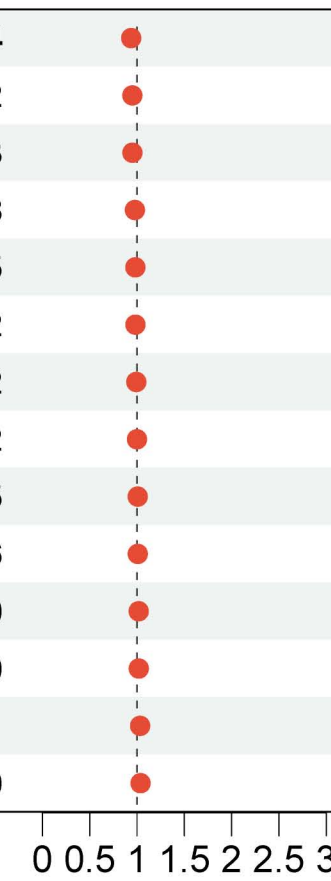

(H)

exposure	outcome	nsnp	method	pval	OR(95% CI)
Diabetic Retinopathy	FGF21	12	IVW	0.2040	0.973 (0.934 to 1.015)
Diabetic Retinopathy	FGF19	12	IVW	0.9366	0.998 (0.956 to 1.043)

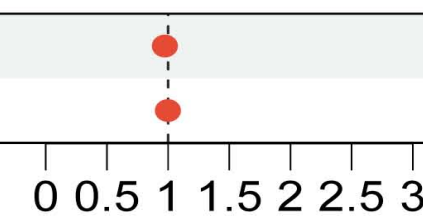

(I)

exposure	outcome	nsnp	method	pval	OR(95% CI)
Diabetic Retinopathy	Phosphatidylinositol (16:0_20:4)	12	IVW	0.4142	0.967 (0.892 to 1.048)
Diabetic Retinopathy	Triacylglycerol (56:7)	12	IVW	0.4347	0.971 (0.903 to 1.045)
Diabetic Retinopathy	Triacylglycerol (54:6)	12	IVW	0.3955	0.975 (0.921 to 1.033)
Diabetic Retinopathy	Triacylglycerol (54:7)	12	IVW	0.4062	0.976 (0.920 to 1.034)
Diabetic Retinopathy	Triacylglycerol (52:4)	12	IVW	0.3939	0.976 (0.922 to 1.033)
Diabetic Retinopathy	Triacylglycerol (52:3)	12	IVW	0.4773	0.978 (0.919 to 1.040)
Diabetic Retinopathy	Triacylglycerol (50:4)	12	IVW	0.4613	0.979 (0.925 to 1.036)
Diabetic Retinopathy	Phosphatidylcholine (18:2_20:4)	12	IVW	0.6529	0.981 (0.904 to 1.065)
Diabetic Retinopathy	Phosphatidylethanolamine (16:0_20:4)	12	IVW	0.6253	0.983 (0.916 to 1.054)
Diabetic Retinopathy	Triacylglycerol (52:5)	12	IVW	0.5585	0.983 (0.929 to 1.041)
Diabetic Retinopathy	Triacylglycerol (51:3)	12	IVW	0.5940	0.985 (0.930 to 1.043)
Diabetic Retinopathy	Triacylglycerol (52:2)	12	IVW	0.6455	0.985 (0.922 to 1.052)
Diabetic Retinopathy	Triacylglycerol (50:3)	12	IVW	0.7031	0.987 (0.924 to 1.055)
Diabetic Retinopathy	Diacylglycerol (18:1_18:1)	12	IVW	0.8076	0.991 (0.920 to 1.067)
Diabetic Retinopathy	Triacylglycerol (56:8)	12	IVW	0.8689	0.994 (0.931 to 1.062)
Diabetic Retinopathy	Sterol ester (27:1/18:3)	12	IVW	0.8893	0.995 (0.931 to 1.064)
Diabetic Retinopathy	Triacylglycerol (50:5)	12	IVW	0.9400	0.997 (0.933 to 1.067)
Diabetic Retinopathy	Sphingomyelin (d36:2)	12	IVW	0.7120	1.013 (0.947 to 1.084)
Diabetic Retinopathy	Triacylglycerol (52:6)	12	IVW	0.6795	1.014 (0.951 to 1.080)
Diabetic Retinopathy	Phosphatidylethanolamine (18:1_18:1)	12	IVW	0.3430	1.042 (0.957 to 1.135)

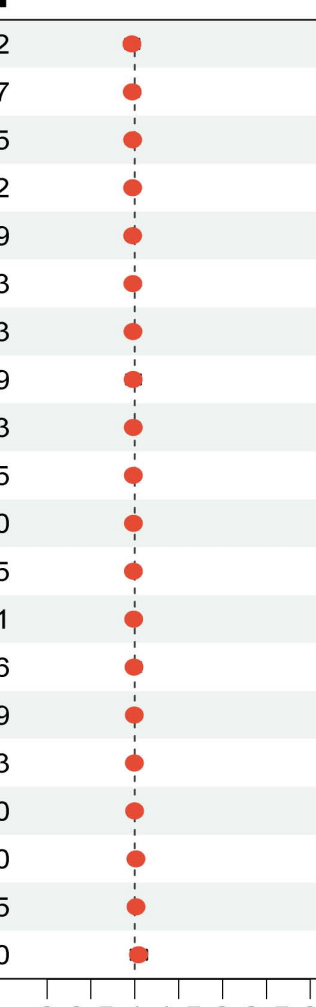

(J)

exposure	outcome	nsnp	method	pval	OR(95% CI)
Diabetic Retinopathy	Glyco−beta−muricholate	11	IVW	0.0538	0.938 (0.879 to 1.001)
Diabetic Retinopathy	X−26109	11	IVW	0.3924	0.972 (0.911 to 1.037)
Diabetic Retinopathy	1−palmitoyl−2−linoleoyl−GPI (16:0/18:2)	11	IVW	0.4181	0.975 (0.918 to 1.036)
Diabetic Retinopathy	Oleoyl−linoleoyl−glycerol (18:1/18:2) [2]	11	IVW	0.4548	0.976 (0.915 to 1.040)
Diabetic Retinopathy	Metabolonic lactone sulfate	11	IVW	0.8297	0.993 (0.936 to 1.055)
Diabetic Retinopathy	Dodecadienoate (12:2)	11	IVW	0.8411	0.994 (0.933 to 1.058)
Diabetic Retinopathy	16a−hydroxy DHEA 3−sulfate	11	IVW	0.8806	0.996 (0.940 to 1.055)
Diabetic Retinopathy	1−stearoyl−2−docosahexaenoyl−GPE (18:0/22:6)	11	IVW	0.8983	0.996 (0.940 to 1.056)
Diabetic Retinopathy	1−palmitoyl−2−docosahexaenoyl−GPE (16:0/22:6)	11	IVW	0.9028	1.004 (0.947 to 1.064)
Diabetic Retinopathy	X−24544	11	IVW	0.8944	1.004 (0.949 to 1.062)
Diabetic Retinopathy	Ceramide (d18:1/24:1)	11	IVW	0.9029	1.005 (0.932 to 1.082)
Diabetic Retinopathy	3−methylcytidine	11	IVW	0.8665	1.005 (0.949 to 1.064)
Diabetic Retinopathy	1−palmitoyl−2−oleoyl−GPI (16:0/18:1)	11	IVW	0.8945	1.005 (0.933 to 1.083)
Diabetic Retinopathy	1−stearoyl−gpc (18:0)	11	IVW	0.8810	1.005 (0.941 to 1.074)
Diabetic Retinopathy	5−hydroxylysine	11	IVW	0.8486	1.006 (0.947 to 1.068)
Diabetic Retinopathy	1−stearoyl−GPE (18:0)	11	IVW	0.8248	1.008 (0.939 to 1.083)
Diabetic Retinopathy	1−palmitoyl−GPE (16:0)	11	IVW	0.7420	1.011 (0.945 to 1.082)
Diabetic Retinopathy	N−stearoyl−sphingosine (d18:1 to 18:0) to N−palmitoyl−sphinganine (d18:0 to 16:0)	11	IVW	0.7472	1.012 (0.943 to 1.085)
Diabetic Retinopathy	Epiandrosterone sulfate	11	IVW	0.6421	1.013 (0.958 to 1.072)
Diabetic Retinopathy	(2 or 3)−decenoate (10:1n7 or n8)	11	IVW	0.6119	1.015 (0.957 to 1.077)
Diabetic Retinopathy	1−palmitoleoyl−GPC (16:1)	11	IVW	0.5902	1.022 (0.945 to 1.105)
Diabetic Retinopathy	Sphingomyelin (d18:1/18:1, d18:2/18:0)	11	IVW	0.4318	1.023 (0.967 to 1.081)
Diabetic Retinopathy	Phosphate to oleoyl−linoleoyl−glycerol (18:1 to 18:2) [2]	11	IVW	0.4046	1.026 (0.966 to 1.090)
Diabetic Retinopathy	Sphingomyelin (d18:1/20:1, d18:2/20:0)	11	IVW	0.2758	1.031 (0.976 to 1.090)
Diabetic Retinopathy	5alpha−androstan−3beta,17beta−diol disulfate	11	IVW	0.1496	1.039 (0.986 to 1.095)
Diabetic Retinopathy	5alpha−androstan−3alpha,17alpha−diol monosulfate	11	IVW	0.2273	1.044 (0.974 to 1.119)
Diabetic Retinopathy	2−stearoyl−GPE (18:0)	11	IVW	0.1306	1.048 (0.986 to 1.114)
Diabetic Retinopathy	Indoleacetylglutamine	11	IVW	0.1032	1.058 (0.989 to 1.132)
Diabetic Retinopathy	Homocitrulline	11	IVW	0.2062	1.060 (0.969 to 1.159)
Diabetic Retinopathy	(S)−a−amino−omega−caprolactam	11	IVW	**0.0351**	1.061 (1.004 to 1.122)
Diabetic Retinopathy	1−(1−enyl−palmitoyl)−2−palmitoyl−GPC (P−16:0/16:0)	11	IVW	0.1545	1.062 (0.977 to 1.155)

0 0.5 1 1.5 2 2.5 3

Fig. (S2). MR analyses for diabetic retinopathy across multiple traits. Forward MR sensitivity analyses using five methods (MR-Egger, weighted median, IVW, simple mode, and weighted mode) are shown in panels A-E, and reverse MR analyses using the IVW method are shown in panels F-J. The five traits are ordered as follows: immune cells, gut microbiota, inflammatory proteins, plasma lipidome, and plasma metabolites. **(A-E)** Forward MR results for each trait. **(F-J)** Reverse MR results for each trait.

(A)

exposure	outcome	nsnp	method	pval	OR(95% CI)
Basophil AC Myeloid cell	Diabetic Neuropathy	3	MR Egger	0.4516	0.964 (0.906 to 1.026)
		3	Weighted median	0.0529	0.955 (0.911 to 1.001)
		3	Inverse variance weighted	**0.0182**	0.953 (0.915 to 0.992)
		3	Simple mode	0.2685	0.953 (0.895 to 1.014)
		3	Weighted mode	0.2192	0.957 (0.911 to 1.005)
CD33dim HLA DR− AC Myeloid cell	Diabetic Neuropathy	3	MR Egger	0.4585	0.965 (0.907 to 1.026)
		3	Weighted median	**0.0362**	0.953 (0.911 to 0.997)
		3	Inverse variance weighted	**0.0162**	0.952 (0.914 to 0.991)
		3	Simple mode	0.2259	0.945 (0.886 to 1.008)
		3	Weighted mode	0.2190	0.956 (0.910 to 1.005)

1

(B)

exposure	outcome	nsnp	method	pval	OR(95% CI)
Species Bacteroides A plebeius	Diabetic Neuropathy	9	MR Egger	0.0876	1.451 (1.005 to 2.097)
		9	Weighted median	0.0680	1.235 (0.984 to 1.550)
		9	Inverse variance weighted	**0.0143**	1.237 (1.043 to 1.466)
		9	Simple mode	0.5000	1.141 (0.791 to 1.646)
		9	Weighted mode	0.4181	1.168 (0.818 to 1.668)
Species Blautia sp000436935	Diabetic Neuropathy	14	MR Egger	0.1512	1.304 (0.929 to 1.830)
		14	Weighted median	**0.0145**	1.362 (1.063 to 1.745)
		14	Inverse variance weighted	**0.0088**	1.267 (1.061 to 1.511)
		14	Simple mode	0.1837	1.393 (0.877 to 2.214)
		14	Weighted mode	0.1160	1.400 (0.946 to 2.069)
Species CAG−273 sp003534295	Diabetic Neuropathy	7	MR Egger	0.1728	1.511 (0.908 to 2.514)
		7	Weighted median	**0.0157**	1.395 (1.065 to 1.827)
		7	Inverse variance weighted	**0.0133**	1.279 (1.053 to 1.555)
		7	Simple mode	0.1092	1.432 (0.985 to 2.082)
		7	Weighted mode	0.1284	1.432 (0.961 to 2.134)
Class Clostridia	Diabetic Neuropathy	6	MR Egger	0.0974	7.865 (1.205 to 51.334)
		6	Weighted median	**0.0398**	3.213 (1.056 to 9.774)
		6	Inverse variance weighted	**0.0408**	2.447 (1.038 to 5.769)
		6	Simple mode	0.2096	3.397 (0.643 to 17.954)
		6	Weighted mode	0.2244	3.319 (0.608 to 18.106)
Genus Enterococcus A	Diabetic Neuropathy	12	MR Egger	0.5080	1.455 (0.499 to 4.244)
		12	Weighted median	**0.0353**	2.130 (1.053 to 4.308)
		12	Inverse variance weighted	**0.0353**	1.738 (1.039 to 2.908)
		12	Simple mode	0.2246	2.393 (0.634 to 9.039)
		12	Weighted mode	0.1729	2.393 (0.740 to 7.735)
Phylum Flavobacteriales	Diabetic Neuropathy	14	MR Egger	**0.0223**	0.177 (0.049 to 0.646)
		14	Weighted median	**0.0381**	0.416 (0.182 to 0.953)
		14	Inverse variance weighted	**0.0065**	0.428 (0.232 to 0.788)
		14	Simple mode	0.8209	0.839 (0.190 to 3.709)
		14	Weighted mode	0.8223	0.839 (0.188 to 3.754)
Species Flavonifractor sp002159265	Diabetic Neuropathy	9	MR Egger	0.2839	0.384 (0.076 to 1.932)
		9	Weighted median	0.0743	0.578 (0.316 to 1.055)
		9	Inverse variance weighted	**0.0038**	0.520 (0.334 to 0.809)
		9	Simple mode	0.3969	0.635 (0.235 to 1.717)
		9	Weighted mode	0.3961	0.630 (0.229 to 1.731)
Species GCA−900066135 sp900066135	Diabetic Neuropathy	7	MR Egger	0.9032	0.908 (0.207 to 3.989)
		7	Weighted median	0.2088	0.615 (0.288 to 1.313)
		7	Inverse variance weighted	**0.0208**	0.523 (0.302 to 0.906)
		7	Simple mode	0.5614	0.726 (0.262 to 2.015)
		7	Weighted mode	0.5628	0.715 (0.245 to 2.091)
Genus Succinivibrio	Diabetic Neuropathy	21	MR Egger	0.0673	0.624 (0.387 to 1.005)
		21	Weighted median	0.3264	0.867 (0.651 to 1.153)
		21	Inverse variance weighted	**0.0385**	0.805 (0.655 to 0.989)
		21	Simple mode	0.7440	0.914 (0.535 to 1.560)
		21	Weighted mode	0.7238	0.914 (0.557 to 1.498)
Species UBA1375 sp002305795	Diabetic Neuropathy	8	MR Egger	0.1958	1.692 (0.833 to 3.436)
		8	Weighted median	**0.0036**	2.135 (1.281 to 3.558)
		8	Inverse variance weighted	**0.0015**	1.956 (1.293 to 2.959)
		8	Simple mode	0.1052	2.177 (0.959 to 4.943)
		8	Weighted mode	0.1093	2.160 (0.948 to 4.921)
Species UBA1448	Diabetic Neuropathy	4	MR Egger	0.5276	10.406 (0.024 to 4444.990)
		4	Weighted median	0.0539	2.347 (0.986 to 5.587)
		4	Inverse variance weighted	**0.0125**	2.514 (1.219 to 5.184)
		4	Simple mode	0.2831	2.241 (0.667 to 7.536)
		4	Weighted mode	0.2768	2.241 (0.680 to 7.390)
Unclassified UBA8517	Diabetic Neuropathy	9	MR Egger	0.3157	0.413 (0.083 to 2.053)
		9	Weighted median	0.2523	0.618 (0.271 to 1.408)
		9	Inverse variance weighted	**0.0499**	0.524 (0.274 to 1.000)
		9	Simple mode	0.4554	0.618 (0.186 to 2.055)
		9	Weighted mode	0.4238	0.610 (0.193 to 1.924)

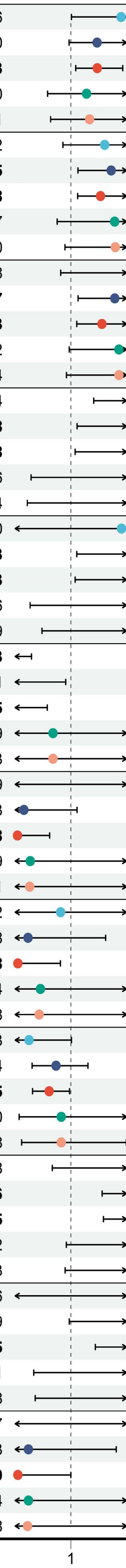

(C)

exposure	outcome	nsnp	method	pval	OR(95% CI)
CCL19	Diabetic Neuropathy	3	MR Egger	0.9540	0.989 (0.736 to 1.330)
		3	Weighted median	**0.0384**	0.854 (0.735 to 0.992)
		3	Inverse variance weighted	**0.0365**	0.865 (0.755 to 0.991)
		3	Simple mode	0.1645	0.772 (0.610 to 0.977)
		3	Weighted mode	0.3617	0.911 (0.780 to 1.064)
LIFR	Diabetic Neuropathy	3	MR Egger	0.6818	1.100 (0.781 to 1.551)
		3	Weighted median	**0.0445**	1.198 (1.004 to 1.429)
		3	Inverse variance weighted	**0.0189**	1.218 (1.033 to 1.436)
		3	Simple mode	0.2654	1.198 (0.951 to 1.511)
		3	Weighted mode	0.2032	1.197 (0.991 to 1.447)
TNFSF14	Diabetic Neuropathy	4	MR Egger	0.2479	0.811 (0.629 to 1.046)
		4	Weighted median	**0.0307**	0.818 (0.682 to 0.981)
		4	Inverse variance weighted	**0.0181**	0.816 (0.689 to 0.966)
		4	Simple mode	0.2901	0.836 (0.635 to 1.100)
		4	Weighted mode	0.1345	0.823 (0.683 to 0.993)

(D)

exposure	outcome	nsnp	method	pval	OR(95% CI)
1−oleoyl−2−docosahexaenoyl−GPC (18:1/22:6)	Diabetic Neuropathy	5	MR Egger	0.7579	0.743 (0.132 to 4.176)
		5	Weighted median	**0.0367**	0.735 (0.550 to 0.981)
		5	Inverse variance weighted	**0.0203**	0.734 (0.565 to 0.953)
		5	Simple mode	0.1246	0.642 (0.410 to 1.005)
		5	Weighted mode	0.1775	0.678 (0.426 to 1.080)
2−butenoylglycine	Diabetic Neuropathy	3	MR Egger	0.7726	0.897 (0.507 to 1.588)
		3	Weighted median	0.0597	0.853 (0.722 to 1.007)
		3	Inverse variance weighted	**0.0201**	0.835 (0.717 to 0.972)
		3	Simple mode	0.3747	0.882 (0.711 to 1.096)
		3	Weighted mode	0.2499	0.860 (0.715 to 1.034)
3−hydroxybutyroylglycine	Diabetic Neuropathy	3	MR Egger	0.5763	0.888 (0.659 to 1.195)
		3	Weighted median	**0.0430**	0.862 (0.746 to 0.995)
		3	Inverse variance weighted	**0.0185**	0.852 (0.746 to 0.974)
		3	Simple mode	0.3684	0.889 (0.728 to 1.086)
		3	Weighted mode	0.2275	0.875 (0.752 to 1.019)
3−hydroxyhexanoylcarnitine (1)	Diabetic Neuropathy	3	MR Egger	0.5096	0.493 (0.118 to 2.058)
		3	Weighted median	**0.0065**	0.677 (0.512 to 0.897)
		3	Inverse variance weighted	**0.0035**	0.701 (0.553 to 0.890)
		3	Simple mode	0.1481	0.671 (0.478 to 0.943)
		3	Weighted mode	0.1518	0.670 (0.473 to 0.948)
Argininate	Diabetic Neuropathy	5	MR Egger	0.3756	1.430 (0.728 to 2.807)
		5	Weighted median	0.0827	1.186 (0.978 to 1.438)
		5	Inverse variance weighted	**0.0331**	1.210 (1.015 to 1.441)
		5	Simple mode	0.2190	1.188 (0.942 to 1.498)
		5	Weighted mode	0.2576	1.183 (0.922 to 1.518)
Carnitine	Diabetic Neuropathy	4	MR Egger	0.2014	0.801 (0.636 to 1.010)
		4	Weighted median	0.0519	0.872 (0.759 to 1.001)
		4	Inverse variance weighted	**0.0449**	0.882 (0.780 to 0.997)
		4	Simple mode	0.3593	0.866 (0.666 to 1.125)
		4	Weighted mode	0.1100	0.854 (0.744 to 0.980)
Indolelactate	Diabetic Neuropathy	3	MR Egger	0.6990	0.729 (0.217 to 2.450)
		3	Weighted median	**0.0328**	0.756 (0.585 to 0.977)
		3	Inverse variance weighted	**0.0272**	0.779 (0.624 to 0.972)
		3	Simple mode	0.2064	0.733 (0.527 to 1.020)
		3	Weighted mode	0.1830	0.742 (0.554 to 0.993)
N−acetylputrescine to (N(1) + N(8))−acetylspermidine	Diabetic Neuropathy	5	MR Egger	0.5448	1.061 (0.895 to 1.258)
		5	Weighted median	**0.0355**	1.104 (1.007 to 1.211)
		5	Inverse variance weighted	**0.0191**	1.112 (1.017 to 1.215)
		5	Simple mode	0.0999	1.226 (1.017 to 1.479)
		5	Weighted mode	0.1189	1.103 (1.001 to 1.214)
Orotate	Diabetic Neuropathy	4	MR Egger	0.5845	1.051 (0.903 to 1.223)
		4	Weighted median	0.0716	1.087 (0.993 to 1.189)
		4	Inverse variance weighted	**0.0377**	1.092 (1.005 to 1.187)
		4	Simple mode	0.1970	1.100 (0.982 to 1.231)
		4	Weighted mode	0.2072	1.084 (0.982 to 1.196)
Paraxanthine to 5−acetylamino−6−formylamino−3−methyluracil	Diabetic Neuropathy	4	MR Egger	0.5608	0.953 (0.832 to 1.092)
		4	Weighted median	0.0656	0.935 (0.870 to 1.004)
		4	Inverse variance weighted	**0.0323**	0.928 (0.867 to 0.994)
		4	Simple mode	0.1917	0.915 (0.825 to 1.015)
		4	Weighted mode	0.2028	0.938 (0.868 to 1.013)
Phosphate to serine	Diabetic Neuropathy	3	MR Egger	0.9638	1.117 (0.025 to 50.056)
		3	Weighted median	**0.0096**	1.342 (1.074 to 1.676)
		3	Inverse variance weighted	**0.0006**	1.411 (1.159 to 1.718)
		3	Simple mode	0.2824	1.258 (0.924 to 1.714)
		3	Weighted mode	0.2666	1.264 (0.935 to 1.708)
Serine	Diabetic Neuropathy	3	MR Egger	0.7180	0.620 (0.086 to 4.475)
		3	Weighted median	**0.0126**	0.782 (0.644 to 0.949)
		3	Inverse variance weighted	**0.0015**	0.741 (0.617 to 0.891)
		3	Simple mode	0.2889	0.822 (0.628 to 1.076)
		3	Weighted mode	0.2710	0.817 (0.629 to 1.063)
X−24328	Diabetic Neuropathy	7	MR Egger	0.2501	0.539 (0.213 to 1.368)
		7	Weighted median	**0.0237**	0.746 (0.579 to 0.962)
		7	Inverse variance weighted	**0.0126**	0.777 (0.637 to 0.947)
		7	Simple mode	0.2003	0.754 (0.513 to 1.108)
		7	Weighted mode	0.0620	0.701 (0.518 to 0.950)
X−24556	Diabetic Neuropathy	3	MR Egger	0.3369	0.424 (0.158 to 1.134)
		3	Weighted median	**0.0067**	0.777 (0.647 to 0.932)
		3	Inverse variance weighted	**0.0051**	0.786 (0.664 to 0.930)
		3	Simple mode	0.3280	0.828 (0.620 to 1.105)
		3	Weighted mode	0.1071	0.755 (0.620 to 0.919)
X−25433	Diabetic Neuropathy	3	MR Egger	0.5567	0.851 (0.583 to 1.242)
		3	Weighted median	**0.0364**	0.863 (0.752 to 0.991)
		3	Inverse variance weighted	**0.0428**	0.875 (0.769 to 0.996)
		3	Simple mode	0.2108	0.821 (0.664 to 1.016)
		3	Weighted mode	0.1761	0.854 (0.735 to 0.993)
X−26054	Diabetic Neuropathy	4	MR Egger	0.5306	1.118 (0.836 to 1.494)
		4	Weighted median	**0.0049**	1.172 (1.049 to 1.310)
		4	Inverse variance weighted	**0.0243**	1.128 (1.016 to 1.254)
		4	Simple mode	0.2138	1.181 (0.960 to 1.454)
		4	Weighted mode	0.0696	1.178 (1.049 to 1.322)

(E)

exposure	outcome	nsnp	method	pval	OR(95% CI)
Carotene	Diabetic Neuropathy	15	MR Egger	0.6937	1.214 (0.472 to 3.121)
		15	Weighted median	0.4224	1.284 (0.697 to 2.366)
		15	Inverse variance weighted	**0.0283**	1.631 (1.053 to 2.525)
		15	Simple mode	0.9168	1.061 (0.358 to 3.139)
		15	Weighted mode	0.9682	1.021 (0.370 to 2.822)

(F)

exposure	outcome	nsnp	method	pval	OR(95% CI)
Diabetic Neuropathy	CD33dim HLA DR− AC Myeloid cell	3	IVW	0.3720	0.905 (0.726 to 1.127)
Diabetic Neuropathy	Basophil AC Myeloid cell	3	IVW	0.3963	0.910 (0.731 to 1.132)

(G)

exposure	outcome	nsnp	method	pval	OR(95% CI)
Diabetic Neuropathy	Genus Succinivibrio	3	IVW	0.0948	0.942 (0.878 to 1.010)
Diabetic Neuropathy	Species Flavonifractor sp002159265	3	IVW	0.0517	0.964 (0.930 to 1.000)
Diabetic Neuropathy	Species UBA1375 sp002305795	3	IVW	0.1265	0.969 (0.931 to 1.009)
Diabetic Neuropathy	Species UBA1448	3	IVW	0.7557	0.994 (0.954 to 1.035)
Diabetic Neuropathy	Class Clostridia	3	IVW	0.8678	0.998 (0.977 to 1.020)
Diabetic Neuropathy	Species CAG−273 sp003534295	3	IVW	0.9961	1.000 (0.908 to 1.101)
Diabetic Neuropathy	Genus Enterococcus A	3	IVW	0.2462	1.015 (0.990 to 1.041)
Diabetic Neuropathy	Phylum Flavobacteriales	3	IVW	0.1507	1.015 (0.995 to 1.036)
Diabetic Neuropathy	Species GCA−900066135 sp900066135	3	IVW	0.2789	1.018 (0.985 to 1.052)
Diabetic Neuropathy	Unclassified UBA8517	3	IVW	**0.0365**	1.030 (1.002 to 1.059)
Diabetic Neuropathy	Species Blautia sp000436935	3	IVW	0.3718	1.032 (0.963 to 1.107)
Diabetic Neuropathy	Species Bacteroides A plebeius	3	IVW	0.1018	1.077 (0.985 to 1.177)

(H)

exposure	outcome	nsnp	method	pval	OR(95% CI)
Diabetic Neuropathy	CCL19	4	IVW	**0.0332**	0.765 (0.597 to 0.979)
Diabetic Neuropathy	LIFR	4	IVW	0.7509	0.982 (0.877 to 1.100)
Diabetic Neuropathy	TNFSF14	4	IVW	0.5506	1.020 (0.956 to 1.089)

(I)

exposure	outcome	nsnp	method	pval	OR(95% CI)
Diabetic Neuropathy	Paraxanthine to 5−acetylamino−6−formylamino−3−methyluracil	3	IVW	0.2210	0.946 (0.866 to 1.034)
Diabetic Neuropathy	Argininate	3	IVW	0.6102	0.967 (0.850 to 1.100)
Diabetic Neuropathy	Phosphate to serine	3	IVW	0.4465	0.968 (0.890 to 1.053)
Diabetic Neuropathy	X−24556	3	IVW	0.6652	0.980 (0.895 to 1.074)
Diabetic Neuropathy	Orotate	3	IVW	0.9638	0.998 (0.918 to 1.085)
Diabetic Neuropathy	N−acetylputrescine to (N(1) + N(8))−acetylspermidine	3	IVW	0.7328	1.015 (0.934 to 1.102)
Diabetic Neuropathy	Serine	3	IVW	0.7234	1.015 (0.935 to 1.101)
Diabetic Neuropathy	Indolelactate	3	IVW	0.6749	1.017 (0.941 to 1.098)
Diabetic Neuropathy	Carnitine	3	IVW	0.4154	1.035 (0.953 to 1.125)
Diabetic Neuropathy	3−hydroxyhexanoylcarnitine (1)	3	IVW	0.4270	1.042 (0.941 to 1.155)
Diabetic Neuropathy	1−oleoyl−2−docosahexaenoyl−GPC (18:1/22:6)	3	IVW	0.2871	1.044 (0.964 to 1.132)
Diabetic Neuropathy	X−26054	3	IVW	0.2351	1.051 (0.968 to 1.140)
Diabetic Neuropathy	X−24328	3	IVW	0.1873	1.053 (0.975 to 1.137)
Diabetic Neuropathy	2−butenoylglycine	3	IVW	0.4935	1.053 (0.908 to 1.222)
Diabetic Neuropathy	3−hydroxybutyroylglycine	3	IVW	0.5025	1.053 (0.905 to 1.227)
Diabetic Neuropathy	X−25433	3	IVW	0.1573	1.070 (0.974 to 1.176)

(J)

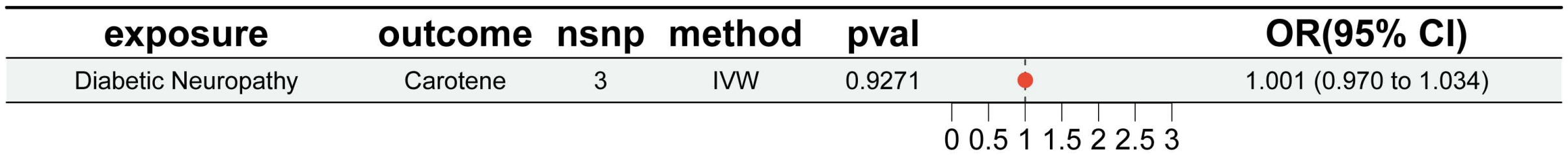

Fig. (3). MR analyses for diabetic neuropathy across multiple traits. Forward MR sensitivity analyses using five methods (MR-Egger, weighted median, IVW, simple mode, and weighted mode) are shown in panels A-E, and reverse MR analyses using the IVW method are shown in panels F-J. The five traits are ordered as follows: immune cells, gut microbiota, inflammatory proteins, plasma metabolites, and micronutrients. **(A-E)** Forward MR results for each trait. **(F-J)** Reverse MR results for each trait.

Supplementary Table 1. Heterogeneity of immune cells, gut microbiota, plasma lipidome, metabolites, micronutrients and diabetic nephropathy.

id.exposure	id.outcome	outcome	exposure	method	Q	Q_df	Q_pval
**b8pNod**	zq7DGg	Diabetic Nephropathy	CD45RA- CD28- CD8br %T cell	MR Egger	32.67478	39	0.752506
**b8pNod**	zq7DGg	Diabetic Nephropathy	CD45RA- CD28- CD8br %T cell	Inverse variance weighted	33.25838	40	0.765799
**e0JbIZ**	04TApx	Diabetic Nephropathy	CD3 on resting Treg	MR Egger	0.563929	4	0.966986
**e0JbIZ**	04TApx	Diabetic Nephropathy	CD3 on resting Treg	Inverse variance weighted	0.706938	5	0.982586
**MLfoHj**	yx6f7Z	Diabetic Nephropathy	Genus Syntrophorhab-dia	Inverse variance weighted	7.922876	5	0.160538
**vFKe0z**	fC41rX	Diabetic Nephropathy	Genus Leuconostoc	MR Egger	4.024008	3	0.258883
**vFKe0z**	fC41rX	Diabetic Nephropathy	Genus Leuconostoc	Inverse variance weighted	4.587911	4	0.33225
**qnEoRz**	tDKjbn	Diabetic Nephropathy	Genus Citrobacter A	Inverse variance weighted	7.203237	7	0.40803
**sHc98h**	K59SSw	Diabetic Nephropathy	Genus Desulfovibrionia	MR Egger	3.77747	4	0.436959
**MLfoHj**	yx6f7Z	Diabetic Nephropathy	Genus Syntrophorhab-dia	MR Egger	3.539433	4	0.471908
**qnEoRz**	tDKjbn	Diabetic Nephropathy	Genus Citrobacter A	MR Egger	5.520025	6	0.47904
**lkDeLm**	YrYYWs	Diabetic Nephropathy	Family Enterobacteri-aceae	MR Egger	7.083264	8	0.527676
**12zi3k**	w6VN2f	Diabetic Nephropathy	Family Desulfovibri-onaceae	MR Egger	5.101467	6	0.530867
**sHc98h**	K59SSw	Diabetic Nephropathy	Genus Desulfovibrionia	Inverse variance weighted	3.792204	5	0.579704
**12zi3k**	w6VN2f	Diabetic Nephropathy	Family Desulfovibri-onaceae	Inverse variance weighted	5.505252	7	0.59855
**lkDeLm**	YrYYWs	Diabetic Nephropathy	Family Enterobacteri-aceae	Inverse variance weighted	7.362141	9	0.599471

**KlvusN**	bsnceZ	Diabetic Nephropathy	Unclassified CAG-145	MR Egger	0.373623	3	0.945636
**KlvusN**	bsnceZ	Diabetic Nephropathy	Unclassified CAG-145	Inverse variance weighted	0.379722	4	0.984103
**QlCZrZ**	K8px4a	Diabetic Nephropathy	Phosphatidylcholine (18:1_18:1)	MR Egger	0.9448	2	0.623504
**QlCZrZ**	K8px4a	Diabetic Nephropathy	Phosphatidylcholine (18:1_18:1)	Inverse variance weighted	1.083704	3	0.781009
**mJ9Isk**	DNXS5L	Diabetic Nephropathy	Phosphatidylinositol (18:1_18:2)	MR Egger	1.23864	1	0.265733
**mJ9Isk**	DNXS5L	Diabetic Nephropathy	Phosphatidylinositol (18:1_18:2)	Inverse variance weighted	1.282205	2	0.526711
**Hf6Lz7**	TayJI7	Diabetic Nephropathy	Phosphatidylcholine (17:0_20:4)	MR Egger	0.554208	4	0.968014
**Hf6Lz7**	TayJI7	Diabetic Nephropathy	Phosphatidylcholine (17:0_20:4)	Inverse variance weighted	1.180566	5	0.946728
**pu3RUo**	ZfF81z	Diabetic Nephropathy	Phosphatidylcholine (16:0_18:2)	MR Egger	5.265394	5	0.384357
**pu3RUo**	ZfF81z	Diabetic Nephropathy	Phosphatidylcholine (16:0_18:2)	Inverse variance weighted	5.411986	6	0.492158
**bV04s8**	wSlm0c	Diabetic Nephropathy	Phosphatidylcholine (18:1_18:2)	MR Egger	4.452052	3	0.216607
**bV04s8**	wSlm0c	Diabetic Nephropathy	Phosphatidylcholine (18:1_18:2)	Inverse variance weighted	4.460943	4	0.347204
**8I91g7**	35h56X	Diabetic Nephropathy	Phosphatidylcholine (18:1_20:4)	MR Egger	3.916204	5	0.561543
**8I91g7**	35h56X	Diabetic Nephropathy	Phosphatidylcholine (18:1_20:4)	Inverse variance weighted	4.280768	6	0.638739
**LnmB8X**	CovL5e	Diabetic Nephropathy	Phosphatidylcholine (16:0_20:4)	MR Egger	0.356937	3	0.948987
**LnmB8X**	CovL5e	Diabetic Nephropathy	Phosphatidylcholine (16:0_20:4)	Inverse variance weighted	0.372459	4	0.984669
**Wa9V91**	UivEFd	Diabetic Nephropathy	Phosphatidylethanola-mine (16:0_18:2)	MR Egger	2.450271	4	0.653562
**Wa9V91**	UivEFd	Diabetic Nephropathy	Phosphatidylethanola-mine (16:0_18:2)	Inverse variance weighted	2.46704	5	0.781451
**fzRHpr**	AR3cUO	Diabetic Nephropathy	Phosphatidylcholine (O-16:1_20:4)	MR Egger	0.48908	1	0.484338
**fzRHpr**	AR3cUO	Diabetic Nephropathy	Phosphatidylcholine (O-16:1_20:4)	Inverse variance weighted	1.930686	2	0.380853
**KiVsEh**	rzihIK	Diabetic Nephropathy	Phosphatidylcholine (15:0_18:2)	MR Egger	5.979509	4	0.200684
**KiVsEh**	rzihIK	Diabetic Nephropathy	Phosphatidylcholine	Inverse variance	6.410128	5	0.268331

			(15:0_18:2)	weighted			
**gqNU3r**	1d4x8n	Diabetic Nephropathy	Sterol ester (27:1/18:3)	MR Egger	1.148154	3	0.765464
**gqNU3r**	1d4x8n	Diabetic Nephropathy	Sterol ester (27:1/18:3)	Inverse variance weighted	1.659393	4	0.79808
**TfZx8e**	iM3wrr	Diabetic Nephropathy	Retinol (Vitamin A) to linoleoyl-arachidonoyl-glycerol (18:2 to 20:4) [2]	MR Egger	0.199017	1	0.655515
**TfZx8e**	iM3wrr	Diabetic Nephropathy	Retinol (Vitamin A) to linoleoyl-arachidonoyl-glycerol (18:2 to 20:4) [2]	Inverse variance weighted	0.236142	2	0.888633
**1GgMwx**	qhK6WH	Diabetic Nephropathy	5,6-dihydrouridine	MR Egger	2.179267	2	0.33634
**1GgMwx**	qhK6WH	Diabetic Nephropathy	5,6-dihydrouridine	Inverse variance weighted	3.377704	3	0.336973
**624Hjg**	kPNPmj	Diabetic Nephropathy	Metabolonic lactone sulfate	MR Egger	2.515358	7	0.925936
**624Hjg**	kPNPmj	Diabetic Nephropathy	Metabolonic lactone sulfate	Inverse variance weighted	5.273478	8	0.727986
**nvDrXF**	JVrQx2	Diabetic Nephropathy	X-25371	MR Egger	1.571778	2	0.455714
**nvDrXF**	JVrQx2	Diabetic Nephropathy	X-25371	Inverse variance weighted	3.047455	3	0.384366
**VPey6M**	Gns5D9	Diabetic Nephropathy	Aspartate to N-acetylglucosamine to N-acetylgalactosamine	MR Egger	0.345024	1	0.556944
**VPey6M**	Gns5D9	Diabetic Nephropathy	Aspartate to N-acetylglucosamine to N-acetylgalactosamine	Inverse variance weighted	0.609545	2	0.737291
**lby2k7**	cUWds7	Diabetic Nephropathy	Hydroxypalmitoyl sphingomyelin (d18:1/16:0(OH))	MR Egger	0.601623	1	0.437959
**lby2k7**	cUWds7	Diabetic Nephropathy	Hydroxypalmitoyl sphingomyelin (d18:1/16:0(OH))	Inverse variance weighted	0.771492	2	0.679943
**MQKGTO**	JEdHr7	Diabetic Nephropathy	1,2-dilinoleoyl-GPE (18:2/18:2)	MR Egger	0.016379	1	0.898164
**MQKGTO**	JEdHr7	Diabetic Nephropathy	1,2-dilinoleoyl-GPE (18:2/18:2)	Inverse variance weighted	0.070136	2	0.96554
**E7reDr**	itw7YH	Diabetic Nephropathy	1-(1-enyl-stearoyl)-2-linoleoyl-GPE (p-18:0/18:2)	MR Egger	0.771058	1	0.37989
**E7reDr**	itw7YH	Diabetic Nephropathy	1-(1-enyl-stearoyl)-2-linoleoyl-GPE (p-18:0/18:2)	Inverse variance weighted	1.707785	2	0.425755

**4rgjda**	uAOeWQ	Diabetic Nephropathy	1-(1-enyl-palmitoyl)-GPC (p-16:0)	MR Egger	0.000588	1	0.980662
**4rgjda**	uAOeWQ	Diabetic Nephropathy	1-(1-enyl-palmitoyl)-GPC (p-16:0)	Inverse variance weighted	0.017669	2	0.991204
**4HOm9s**	WiqhIz	Diabetic Nephropathy	1-(1-enyl-palmitoyl)-2-arachidonoyl-gpc (p-16:0/20:4)	MR Egger	0.058469	1	0.808932
**4HOm9s**	WiqhIz	Diabetic Nephropathy	1-(1-enyl-palmitoyl)-2-arachidonoyl-gpc (p-16:0/20:4)	Inverse variance weighted	0.215539	2	0.897834
**9dqLhO**	piHxrh	Diabetic Nephropathy	X-26109	MR Egger	3.233829	4	0.519484
**9dqLhO**	piHxrh	Diabetic Nephropathy	X-26109	Inverse variance weighted	3.990052	5	0.550849
**hbRDqp**	dLppa6	Diabetic Nephropathy	1-stearoyl-2-docosahexaenoyl-gpc (18:0/22:6)	MR Egger	1.915096	1	0.166398
**hbRDqp**	dLppa6	Diabetic Nephropathy	1-stearoyl-2-docosahexaenoyl-gpc (18:0/22:6)	Inverse variance weighted	2.501204	2	0.286332
**jqwFI8**	mhn6RG	Diabetic Nephropathy	1-stearoyl-2-linoleoyl-gpc (18:0/18:2)	MR Egger	0.666581	1	0.414246
**jqwFI8**	mhn6RG	Diabetic Nephropathy	1-stearoyl-2-linoleoyl-gpc (18:0/18:2)	Inverse variance weighted	0.765243	2	0.682071
**7chK7S**	cKr0c0	Diabetic Nephropathy	1-oleoyl-2-linoleoyl-GPE (18:1/18:2)	MR Egger	2.515179	3	0.472554
**7chK7S**	cKr0c0	Diabetic Nephropathy	1-oleoyl-2-linoleoyl-GPE (18:1/18:2)	Inverse variance weighted	3.189494	4	0.526629
**LMiCpT**	l5T9GE	Diabetic Nephropathy	Cholesterol to linoleoyl-arachidonoyl-glycerol (18:2 to 20:4) [1]	MR Egger	0.017749	1	0.894016
**LMiCpT**	l5T9GE	Diabetic Nephropathy	Cholesterol to linoleoyl-arachidonoyl-glycerol (18:2 to 20:4) [1]	Inverse variance weighted	0.089466	2	0.956253
**ybDRip**	L2eZ9i	Diabetic Nephropathy	Retinol (Vitamin A) to linoleoyl-arachidonoyl-glycerol (18:2 to 20:4) [1]	MR Egger	0.588331	2	0.745153
**ybDRip**	L2eZ9i	Diabetic Nephropathy	Retinol (Vitamin A) to linoleoyl-arachidonoyl-glycerol (18:2 to 20:4) [1]	Inverse variance weighted	0.746769	3	0.862152
**h84UTY**	R77p1m	Diabetic Nephropathy	1-arachidonoyl-GPE (20:4n6)	MR Egger	0.844407	2	0.655601
**h84UTY**	R77p1m	Diabetic Nephropathy	1-arachidonoyl-GPE (20:4n6)	Inverse variance weighted	2.024186	3	0.567402

**kirmzQ**	6r7ZWj	Diabetic Nephropathy	Dodecadienoate (12:2)	MR Egger	2.257077	3	0.520794
**kirmzQ**	6r7ZWj	Diabetic Nephropathy	Dodecadienoate (12:2)	Inverse variance weighted	2.294366	4	0.681795
**g70kVI**	OYTDqq	Diabetic Nephropathy	Phosphate to linoleoyl-arachidonoyl-glycerol (18:2 to 20:4) [1]	MR Egger	0.023183	1	0.878983
**g70kVI**	OYTDqq	Diabetic Nephropathy	Phosphate to linoleoyl-arachidonoyl-glycerol (18:2 to 20:4) [1]	Inverse variance weighted	0.095787	2	0.953235
**iQutaT**	diVRYH	Diabetic Nephropathy	1-arachidonoyl-gpc (20:4n6)	MR Egger	3.607509	3	0.307084
**iQutaT**	diVRYH	Diabetic Nephropathy	1-arachidonoyl-gpc (20:4n6)	Inverse variance weighted	4.163871	4	0.384282
**HE7N3U**	5zswqr	Diabetic Nephropathy	X-26111	MR Egger	0.443317	3	0.931156
**HE7N3U**	5zswqr	Diabetic Nephropathy	X-26111	Inverse variance weighted	0.926414	4	0.920741
**B8DN0f**	avqS5X	Diabetic Nephropathy	1-palmitoyl-2-linoleoyl-GPI (16:0/18:2)	MR Egger	5.691457	3	0.127625
**B8DN0f**	avqS5X	Diabetic Nephropathy	1-palmitoyl-2-linoleoyl-GPI (16:0/18:2)	Inverse variance weighted	5.817604	4	0.21319
**TI2anZ**	BHYvte	Diabetic Nephropathy	N-stearoyl-sphingosine (d18:1 to 18:0) to N-palmitoyl-sphinganine (d18:0 to 16:0)	MR Egger	1.833428	3	0.607688
**TI2anZ**	BHYvte	Diabetic Nephropathy	N-stearoyl-sphingosine (d18:1 to 18:0) to N-palmitoyl-sphinganine (d18:0 to 16:0)	Inverse variance weighted	1.833664	4	0.766318
**hmWi4k**	AK7knv	Diabetic Nephropathy	Vanillic acid glycine	MR Egger	1.79934	2	0.406704
**hmWi4k**	AK7knv	Diabetic Nephropathy	Vanillic acid glycine	Inverse variance weighted	1.829518	3	0.608533
**wlc5W3**	PjxlH7	Diabetic Nephropathy	1-linoleoyl-GPG (18:2)	MR Egger	0.281528	2	0.868694
**wlc5W3**	PjxlH7	Diabetic Nephropathy	1-linoleoyl-GPG (18:2)	Inverse variance weighted	0.783931	3	0.853306
**y8jtl7**	jLHq91	Diabetic Nephropathy	Tetradecanedioate (C14-DC)	MR Egger	3.417564	4	0.490523
**y8jtl7**	jLHq91	Diabetic Nephropathy	Tetradecanedioate (C14-DC)	Inverse variance weighted	4.948411	5	0.422209
**iH0eU6**	8SByaG	Diabetic Nephropathy	X-18922	MR Egger	2.580929	4	0.630205
**iH0eU6**	8SByaG	Diabetic Nephropathy	X-18922	Inverse variance weighted	2.630912	5	0.756663
**5OtKhJ**	L7Hrog	Diabetic Nephropathy	Hexadecenedioate (C16:1-DC)	MR Egger	0.644344	2	0.724574

**5OtKhJ**	L7Hrog	Diabetic Nephropathy	Hexadecenedioate (C16:1-DC)	Inverse variance weighted	1.012511	3	0.798225
**Rkf61u**	4A4T3s	Diabetic Nephropathy	Caffeine to paraxan-thine	MR Egger	0.219219	1	0.639636
**Rkf61u**	4A4T3s	Diabetic Nephropathy	Caffeine to paraxan-thine	Inverse variance weighted	1.307471	2	0.520099
**fQECrK**	oNe8Yz	Diabetic Nephropathy	1-oleoyl-GPG (18:1)	MR Egger	0.316243	3	0.956945
**fQECrK**	oNe8Yz	Diabetic Nephropathy	1-oleoyl-GPG (18:1)	Inverse variance weighted	1.154102	4	0.885596
**9yvflm**	tDiacc	Diabetic Nephropathy	N2-acetyl,N6,N6-dimethyllysine	MR Egger	0.912611	4	0.922745
**9yvflm**	tDiacc	Diabetic Nephropathy	N2-acetyl,N6,N6-dimethyllysine	Inverse variance weighted	1.024759	5	0.960544
**BFLBzj**	aB2gv1	Diabetic Nephropathy	21-hydroxypregnenolone disulfate	MR Egger	3.874331	5	0.567647
**BFLBzj**	aB2gv1	Diabetic Nephropathy	21-hydroxypregnenolone disulfate	Inverse variance weighted	4.636596	6	0.591192
**ijpHQ5**	QNaR7f	Diabetic Nephropathy	Sphingomyelin (d18:2/21:0, d16:2/23:0)	MR Egger	0.642032	2	0.725412
**ijpHQ5**	QNaR7f	Diabetic Nephropathy	Sphingomyelin (d18:2/21:0, d16:2/23:0)	Inverse variance weighted	0.966244	3	0.809419
**l3zCQb**	4JnX0c	Diabetic Nephropathy	1-palmitoyl-2-linoleoyl-GPE (16:0/18:2)	MR Egger	4.689678	4	0.320645
**l3zCQb**	4JnX0c	Diabetic Nephropathy	1-palmitoyl-2-linoleoyl-GPE (16:0/18:2)	Inverse variance weighted	4.82085	5	0.438133
**C9rYc0**	MJnXUf	Diabetic Nephropathy	5-methylthioadenosine (MTA) to phosphate	MR Egger	2.368056	2	0.306043
**C9rYc0**	MJnXUf	Diabetic Nephropathy	5-methylthioadenosine (MTA) to phosphate	Inverse variance weighted	2.437734	3	0.486649
**BMc4vp**	uI4tyo	Diabetic Nephropathy	Linoleoyl-arachidonoyl-glycerol (18:2/20:4) [1]	MR Egger	0.236703	1	0.626598
**BMc4vp**	uI4tyo	Diabetic Nephropathy	Linoleoyl-arachidonoyl-glycerol (18:2/20:4) [1]	Inverse variance weighted	0.279567	2	0.869546
**ke3P4X**	P3ote7	Diabetic Nephropathy	Selenium	MR Egger	3.770977	4	0.437887
**ke3P4X**	P3ote7	Diabetic Nephropathy	Selenium	Inverse variance weighted	3.933355	5	0.559051

Supplementary Table 2. Pleiotropy of immune cells, gut microbiota, plasma lipidome, metabolites, micronutrients and diabetic nephropathy.

id.exposure	id.outcome	outcome	exposure	egger_intercept	se	pval
b8pNod	zq7DGg	Diabetic Nephropathy	CD45RA- CD28- CD8br %T cell	-0.0094	0.012301	0.449503
e0JbIZ	04TApx	Diabetic Nephropathy	CD3 on resting Treg	0.00822	0.021737	0.724521
MLfoHj	yx6f7Z	Diabetic Nephropathy	Genus Syntrophorhabdia	0.068331	0.032637	0.104397
qnEoRz	tDKjbn	Diabetic Nephropathy	Genus Citrobacter A	-0.03438	0.026499	0.242146
12zi3k	w6VN2f	Diabetic Nephropathy	Family Desulfovibrionaceae	0.021387	0.033656	0.548594
vFKe0z	fC41rX	Diabetic Nephropathy	Genus Leuconostoc	-0.03216	0.049607	0.562941
lkDeLm	YrYYWs	Diabetic Nephropathy	Family Enterobacteriaceae	0.014445	0.027353	0.611769
sHc98h	K59SSw	Diabetic Nephropathy	Genus Desulfovibrionia	0.00467	0.038471	0.909243
KlvusN	bsnceZ	Diabetic Nephropathy	Unclassified CAG-145	-0.00672	0.086053	0.942669
QlCZrZ	K8px4a	Diabetic Nephropathy	Phosphatidylcholine (18:1_18:1)	0.0136	0.036492	0.745163
mJ9Isk	DNXS5L	Diabetic Nephropathy	Phosphatidylinositol (18:1_18:2)	-0.02208	0.117717	0.881979
Hf6Lz7	TayJI7	Diabetic Nephropathy	Phosphatidylcholine (17:0_20:4)	-0.01956	0.024713	0.47298
pu3RUo	ZfF81z	Diabetic Nephropathy	Phosphatidylcholine (16:0_18:2)	-0.00749	0.020065	0.724369
bV04s8	wSlm0c	Diabetic Nephropathy	Phosphatidylcholine (18:1_18:2)	0.001723	0.022262	0.943176
8I91g7	35h56X	Diabetic Nephropathy	Phosphatidylcholine (18:1_20:4)	-0.01263	0.020924	0.572327
LnmB8X	CovL5e	Diabetic Nephropathy	Phosphatidylcholine (16:0_20:4)	0.00244	0.019584	0.908732
Wa9V91	UivEFd	Diabetic Nephropathy	Phosphatidylethanolamine (16:0_18:2)	-0.0038	0.029337	0.903216
fzRHpr	AR3cUO	Diabetic Nephropathy	Phosphatidylcholine (O-16:1_20:4)	0.028916	0.024083	0.44211
KiVsEh	rzihIK	Diabetic Nephropathy	Phosphatidylcholine (15:0_18:2)	-0.01336	0.024885	0.619925

gqNU3r	1d4x8n	Diabetic Nephropathy	Sterol ester (27:1/18:3)	0.021186	0.02963	0.526223
TfZx8e	iM3wrr	Diabetic Nephropathy	Retinol (Vitamin A) to linoleoyl-arachidonoyl-glycerol (18:2 to 20:4) [2]	0.011488	0.059625	0.878823
1GgMwx	qhK6WH	Diabetic Nephropathy	5,6-dihydrouridine	-0.03179	0.030308	0.404342
624Hjg	kPNPmj	Diabetic Nephropathy	Metabolonic lactone sulfate	0.025043	0.015079	0.140719
nvDrXF	JVrQx2	Diabetic Nephropathy	X-25371	-0.0344	0.02832	0.348408
VPey6M	Gns5D9	Diabetic Nephropathy	Aspartate to N-acetylglucosamine to N-acetylgalactosamine	-0.02061	0.040081	0.697583
lby2k7	cUWds7	Diabetic Nephropathy	Hydroxypalmitoyl sphingomyelin (d18:1/16:0(OH))	-0.01255	0.030461	0.751121
MQKGTO	JEdHr7	Diabetic Nephropathy	1,2-dilinoleoyl-GPE (18:2/18:2)	-0.00833	0.035926	0.854959
E7reDr	itw7YH	Diabetic Nephropathy	1-(1-enyl-stearoyl)-2-linoleoyl-GPE (p-18:0/18:2)	-0.05731	0.059217	0.510401
4rgjda	uAOeWQ	Diabetic Nephropathy	1-(1-enyl-palmitoyl)-GPC (p-16:0)	0.007048	0.053925	0.917265
4HOm9s	WiqhIz	Diabetic Nephropathy	1-(1-enyl-palmitoyl)-2-arachidonoyl-gpc (p-16:0/20:4)	0.009916	0.02502	0.759784
9dqLhO	piHxrh	Diabetic Nephropathy	X-26109	0.012552	0.014434	0.433584
hbRDqp	dLppa6	Diabetic Nephropathy	1-stearoyl-2-docosahexaenoyl-gpc (18:0/22:6)	-0.02259	0.04084	0.678311
jqwFI8	mhn6RG	Diabetic Nephropathy	1-stearoyl-2-linoleoyl-gpc (18:0/18:2)	-0.00965	0.030731	0.806247
7chK7S	cKr0c0	Diabetic Nephropathy	1-oleoyl-2-linoleoyl-GPE (18:1/18:2)	-0.02057	0.025048	0.471728
LMiCpT	l5T9GE	Diabetic Nephropathy	Cholesterol to linoleoyl-arachidonoyl-glycerol (18:2 to 20:4) [1]	0.017764	0.066331	0.833421
ybDRip	L2eZ9i	Diabetic Nephropathy	Retinol (Vitamin A) to linoleoyl-arachidonoyl-glycerol (18:2 to 20:4) [1]	-0.01947	0.048916	0.729069
h84UTY	R77p1m	Diabetic Nephropathy	1-arachidonoyl-GPE (20:4n6)	0.025661	0.023625	0.390881
kirmzQ	6r7ZWj	Diabetic Nephropathy	Dodecadienoate (12:2)	-0.00478	0.02476	0.85921
g70kVI	OYTDqq	Diabetic Nephropathy	Phosphate to linoleoyl-arachidonoyl-glycerol (18:2 to 20:4) [1]	0.016005	0.059398	0.832441
iQutaT	diVRYH	Diabetic Nephropathy	1-arachidonoyl-gpc (20:4n6)	0.01198	0.017612	0.545168

HE7N3U	5zswqr	Diabetic Nephropathy	X-26111	0.021924	0.031543	0.53702
B8DN0f	avqS5X	Diabetic Nephropathy	1-palmitoyl-2-linoleoyl-GPI (16:0/18:2)	0.01159	0.044946	0.813191
TI2anZ	BHYvte	Diabetic Nephropathy	N-stearoyl-sphingosine (d18:1 to 18:0) to N-palmitoyl-sphinganine (d18:0 to 16:0)	-0.00057	0.037326	0.988698
hmWi4k	AK7knv	Diabetic Nephropathy	Vanillic acid glycine	-0.00629	0.03618	0.878079
wlc5W3	PjxlH7	Diabetic Nephropathy	1-linoleoyl-GPG (18:2)	0.024617	0.03473	0.551928
y8jtl7	jLHq91	Diabetic Nephropathy	Tetradecanedioate (C14-DC)	0.025146	0.020324	0.283655
iH0eU6	8SByaG	Diabetic Nephropathy	X-18922	-0.00592	0.026492	0.834047
5OtKhJ	L7Hrog	Diabetic Nephropathy	Hexadecenedioate (C16:1-DC)	0.021456	0.035361	0.605709
Rkf61u	4A4T3s	Diabetic Nephropathy	Caffeine to paraxanthine	0.068627	0.065785	0.486544
fQECrK	oNe8Yz	Diabetic Nephropathy	1-oleoyl-GPG (18:1)	0.021176	0.023134	0.427507
9yvflm	tDiacc	Diabetic Nephropathy	N2-acetyl,N6,N6-dimethyllysine	0.01323	0.039507	0.754537
BFLBzj	aB2gv1	Diabetic Nephropathy	21-hydroxypregnenolone disulfate	0.026488	0.030338	0.422562
ijpHQ5	QNaR7f	Diabetic Nephropathy	Sphingomyelin (d18:2/21:0, d16:2/23:0)	-0.01639	0.02879	0.626512
l3zCQb	4JnX0c	Diabetic Nephropathy	1-palmitoyl-2-linoleoyl-GPE (16:0/18:2)	0.006864	0.02052	0.754816
C9rYc0	MJnXUf	Diabetic Nephropathy	5-methylthioadenosine (MTA) to phosphate	-0.02328	0.095958	0.830935
BMc4vp	uI4tyo	Diabetic Nephropathy	Linoleoyl-arachidonoyl-glycerol (18:2/20:4) [1]	0.013291	0.064195	0.870032
ke3P4X	P3ote7	Diabetic Nephropathy	Selenium	-0.008	0.019862	0.707585

Supplementary Table 3. Heterogeneity of immune cells, gut microbiota and plasma lipidome in diabetic nephropathy.

id.exposure	id.outcome	outcome	exposure	method	Q	Q_df	Q_pval
ebi-a-GCST90001869	yR62xi	Family Enterobacteriaceae	CD3 on resting Treg	MR Egger	3.752669	4	0.440512
ebi-a-GCST90001869	yR62xi	Family Enterobacteriaceae	CD3 on resting Treg	Inverse variance weighted	4.19399	5	0.521838
ebi-a-GCST90001869	bYElLL	Phosphatidylcholine (16:0_18:2)	CD3 on resting Treg	MR Egger	2.603254	4	0.626247
ebi-a-GCST90001869	bYElLL	Phosphatidylcholine (16:0_18:2)	CD3 on resting Treg	Inverse variance weighted	2.66961	5	0.750762
ebi-a-GCST90001697	TiG5y8	Phosphatidylcholine (18:1_20:4)	CD45RA- CD28- CD8br %T cell	MR Egger	28.13126	34	0.750211
ebi-a-GCST90001697	TiG5y8	Phosphatidylcholine (18:1_20:4)	CD45RA- CD28- CD8br %T cell	Inverse variance weighted	28.18944	35	0.785996

Supplementary Table 4. Pleiotropy of immune cells, gut microbiota and plasma lipidome in diabetic nephropathy.

id.exposure	id.outcome	outcome	exposure	egger_intercept	se	pval
**ebi-a-GCST90001869**	yR62xi	Family Enterobacteriaceae	CD3 on resting Treg	-0.00985	0.014823	0.542823
**ebi-a-GCST90001869**	bYElLL	Phosphatidylcholine (16:0_18:2)	CD3 on resting Treg	0.004804	0.01865	0.809427
**ebi-a-GCST90001697**	TiG5y8	Phosphatidylcholine (18:1_20:4)	CD45RA- CD28- CD8br %T cell	0.003282	0.013605	0.810842

Supplementary Table 5. Heterogeneity of immune cells, gut microbiota, inflammatory proteins, plasma lipidome, metabolites and diabetic retinopathy.

**IQdzWz**	**sa5RQS**	**Diabetic Retinopathy**	**CD20 on IgD+ CD38dim B cell**	**Inverse variance weighted**	**10.79796**	**6**	**0.094825**
**P1gIq6**	xGPxDn	Diabetic Retinopathy	CD25 on IgD- CD38- B cell	Inverse variance weighted	2.477014	2	0.289817
**W7f7y2**	eHuGhM	Diabetic Retinopathy	CD27 on CD24+ CD27+ B cell	Inverse variance weighted	6.735441	10	0.750163
**G9F1Bp**	UNhthV	Diabetic Retinopathy	CD27 on IgD- CD38dim B cell	Inverse variance weighted	6.422617	8	0.600003
**7Ag4jo**	oeq41x	Diabetic Retinopathy	CD27 on sw mem B cell	Inverse variance weighted	4.833309	9	0.84859
**qnXl7B**	0L7TPO	Diabetic Retinopathy	CD28 on CD28+ CD45RA+ CD8br Treg	Inverse variance weighted	1.844337	2	0.397656
**MRAjV7**	bc7B8W	Diabetic Retinopathy	CD45RA+ CD28- CD8br %T cell	Inverse variance weighted	62.67712	66	0.593247
**CZbABk**	tZJlRz	Diabetic Retinopathy	CD45RA+ CD28- CD8br AC Treg	Inverse variance weighted	422.653	425	0.523035

**gdaaCY**	k8DGKF	Diabetic Retinopathy	CD80 on CD62L+ myeloid DC	Inverse variance weighted	1.039761	2	0.594592
**49RlmT**	o0Tcfs	Diabetic Retinopathy	CD80 on monocyte cDC	Inverse variance weighted	2.827567	5	0.726549
**225R5H**	9mFPpR	Diabetic Retinopathy	CD80 on myeloid DC	Inverse variance weighted	1.460163	3	0.691495
**zreERZ**	hJd1Yr	Diabetic Retinopathy	CX3CR1 on CD14+ CD16- monocyte	Inverse variance weighted	0.291884	2	0.864208
**KvivHc**	CJAaTz	Diabetic Retinopathy	CX3CR1 on CD14+ CD16+ monocyte	Inverse variance weighted	0.047361	2	0.976598
**i6akJV**	l0SteX	Diabetic Retinopathy	CX3CR1 on monocyte	Inverse variance weighted	0.298276	2	0.86145
**8nFx2D**	D1uxkU	Diabetic Retinopathy	IgD on IgD+ CD38br B cell	Inverse variance weighted	0.33438	5	0.996946
**UEhDkV**	U01D9h	Diabetic Retinopathy	IgD on transitional B cell	Inverse variance weighted	0.184212	4	0.99601
**IQdzWz**	sa5RQS	Diabetic Retinopathy	CD20 on IgD+ CD38dim B cell	MR Egger	10.72197	5	0.05718
**P1gIq6**	xGPxDn	Diabetic Retinopathy	CD25 on IgD- CD38- B cell	MR Egger	0.000992	1	0.974869
**W7f7y2**	eHuGhM	Diabetic Retinopathy	CD27 on CD24+ CD27+ B cell	MR Egger	4.235617	9	0.895232
**G9F1Bp**	UNhthV	Diabetic Retinopathy	CD27 on IgD- CD38dim B cell	MR Egger	4.130826	7	0.764592
**7Ag4jo**	oeq41x	Diabetic Retinopathy	CD27 on sw mem B cell	MR Egger	4.026407	8	0.854733
**qnXl7B**	0L7TPO	Diabetic Retinopathy	CD28 on CD28+ CD45RA+ CD8br Treg	MR Egger	0.014732	1	0.903393
**MRAjV7**	bc7B8W	Diabetic Retinopathy	CD45RA+ CD28- CD8br %T cell	MR Egger	62.58675	65	0.561841
**CZbABk**	tZJlRz	Diabetic Retinopathy	CD45RA+ CD28- CD8br AC Treg	MR Egger	419.6699	424	0.550249
**gdaaCY**	k8DGKF	Diabetic Retinopathy	CD80 on CD62L+ myeloid DC	MR Egger	0.911705	1	0.339662
**49RlmT**	o0Tcfs	Diabetic Retinopathy	CD80 on monocyte cDC	MR Egger	2.281716	4	0.6841
**225R5H**	9mFPpR	Diabetic Retinopathy	CD80 on myeloid DC	MR Egger	1.299682	2	0.522129
**zreERZ**	hJd1Yr	Diabetic Retinopathy	CX3CR1 on CD14+ CD16- monocyte	MR Egger	0.121726	1	0.72717
**KvivHc**	CJAaTz	Diabetic Retinopathy	CX3CR1 on CD14+ CD16+ monocyte	MR Egger	0.045339	1	0.831382
**i6akJV**	l0SteX	Diabetic Retinopathy	CX3CR1 on monocyte	MR Egger	0.224082	1	0.635947
**8nFx2D**	D1uxkU	Diabetic Retinopathy	IgD on IgD+ CD38br B cell	MR Egger	0.260136	4	0.99224

**UEhDkV**	U01D9h	Diabetic Retinopathy	IgD on transitional B cell	MR Egger	0.171443	3	0.982062
**qv20wE**	GcoB5g	Diabetic Retinopathy	Order Actinomycetales	Inverse variance weighted	1.226477	3	0.746661
**qv20wE**	GcoB5g	Diabetic Retinopathy	Order Actinomycetales	MR Egger	1.166631	2	0.558045
**9PmFLE**	aRCG0o	Diabetic Retinopathy	Genus An181	Inverse variance weighted	4.248496	5	0.514221
**9PmFLE**	aRCG0o	Diabetic Retinopathy	Genus An181	MR Egger	3.499267	4	0.47799
**j07qK9**	Twqs55	Diabetic Retinopathy	Unclassified Bacilli A	Inverse variance weighted	10.3019	7	0.172101
**j07qK9**	Twqs55	Diabetic Retinopathy	Unclassified Bacilli A	MR Egger	9.346377	6	0.155015
**1e82KN**	bLaFve	Diabetic Retinopathy	Species Bacteroides stercoris	Inverse variance weighted	8.445116	6	0.207272
**1e82KN**	bLaFve	Diabetic Retinopathy	Species Bacteroides stercoris	MR Egger	8.400513	5	0.1355
**hZGxLU**	kVgIiB	Diabetic Retinopathy	Family Bifidobacteriaceae	Inverse variance weighted	5.290615	7	0.624545
**hZGxLU**	kVgIiB	Diabetic Retinopathy	Family Bifidobacteriaceae	MR Egger	5.21344	6	0.516744
**Q9eu8n**	czUXKx	Diabetic Retinopathy	Species Blautia hansenii	Inverse variance weighted	6.618845	10	0.760871
**Q9eu8n**	czUXKx	Diabetic Retinopathy	Species Blautia hansenii	MR Egger	6.251216	9	0.714536
**M6q4iF**	NiuuUO	Diabetic Retinopathy	Species CAG-177 sp003538135	Inverse variance weighted	4.758261	7	0.689436
**M6q4iF**	NiuuUO	Diabetic Retinopathy	Species CAG-177 sp003538135	MR Egger	4.581056	6	0.598552
**cvTKcn**	H21rHx	Diabetic Retinopathy	Unclassified CAG-475	Inverse variance weighted	4.293729	13	0.987647
**cvTKcn**	H21rHx	Diabetic Retinopathy	Unclassified CAG-475	MR Egger	4.233243	12	0.97885
**82ZVf5**	ArUaoV	Diabetic Retinopathy	Species Clostridium M sp001304855	Inverse variance weighted	7.616066	7	0.367662
**82ZVf5**	ArUaoV	Diabetic Retinopathy	Species Clostridium M sp001304855	MR Egger	6.136184	6	0.408109
**C5EmUe**	chqRE4	Diabetic Retinopathy	Unclassified Firmicutes A	Inverse variance weighted	5.523038	6	0.478677
**C5EmUe**	chqRE4	Diabetic Retinopathy	Unclassified Firmicutes A	MR Egger	4.292051	5	0.508178
**L3qiCU**	tNf1rM	Diabetic Retinopathy	Unclassified Geobacter C	Inverse variance weighted	4.337071	5	0.501974
**L3qiCU**	tNf1rM	Diabetic Retinopathy	Unclassified Geobacter C	MR Egger	4.001895	4	0.405749
**CJOz4c**	HMk589	Diabetic Retinopathy	Genus Holdemania	Inverse variance weighted	9.359479	11	0.588748

**CJOz4c**	HMk589	Diabetic Retinopathy	Genus Holdemania	MR Egger	8.663896	10	0.56427
**27WinM**	ASaoL1	Diabetic Retinopathy	Species Lachnospira rogo-sae	Inverse variance weighted	5.968201	9	0.743097
**27WinM**	ASaoL1	Diabetic Retinopathy	Species Lachnospira rogo-sae	MR Egger	5.737974	8	0.676556
**LSuOnJ**	RX32r3	Diabetic Retinopathy	Species Pseudomonas aeru-ginosa	Inverse variance weighted	2.781993	6	0.835669
**LSuOnJ**	RX32r3	Diabetic Retinopathy	Species Pseudomonas aeru-ginosa	MR Egger	1.396342	5	0.924713
**1VX5lC**	WSCHW9	Diabetic Retinopathy	FGF21	MR Egger	0.486168	2	0.784206
**1VX5lC**	WSCHW9	Diabetic Retinopathy	FGF21	Inverse variance weighted	0.812479	3	0.84648
**XuPoyn**	mZSALd	Diabetic Retinopathy	FGF19	MR Egger	1.732307	1	0.188117
**XuPoyn**	mZSALd	Diabetic Retinopathy	FGF19	Inverse variance weighted	1.995132	2	0.368776
**bsBOz-cI37D41A2:I35A2:H37A2:H38A2:I36A2:I35**	SFfuED	Diabetic Retinopathy	Triacylglycerol (52:6)	MR Egger	0.163658	2	0.92143
**bsBOzc**	SFfuED	Diabetic Retinopathy	Triacylglycerol (52:6)	Inverse variance weighted	0.58648	3	0.89952
**8ji37G**	2ds89P	Diabetic Retinopathy	Triacylglycerol (54:7)	MR Egger	0.118073	2	0.942672
**8ji37G**	2ds89P	Diabetic Retinopathy	Triacylglycerol (54:7)	Inverse variance weighted	0.722036	3	0.868009
**B8InWk**	cmbNsf	Diabetic Retinopathy	Triacylglycerol (52:4)	MR Egger	4.376047	5	0.496637
**B8InWk**	cmbNsf	Diabetic Retinopathy	Triacylglycerol (52:4)	Inverse variance weighted	4.875531	6	0.559874
**4iZDq0**	4H7aUp	Diabetic Retinopathy	Triacylglycerol (56:7)	MR Egger	1.836092	3	0.607113
**4iZDq0**	4H7aUp	Diabetic Retinopathy	Triacylglycerol (56:7)	Inverse variance weighted	2.879448	4	0.578198
**TL6Xt2**	zdtFFn	Diabetic Retinopathy	Phosphatidylethanolamine (16:0_20:4)	MR Egger	1.167776	3	0.760743
**TL6Xt2**	zdtFFn	Diabetic Retinopathy	Phosphatidylethanolamine (16:0_20:4)	Inverse variance weighted	2.900938	4	0.574538
**mdVeuH**	pCQrL2	Diabetic Retinopathy	Triacylglycerol (51:3)	MR Egger	2.685385	4	0.611774
**mdVeuH**	pCQrL2	Diabetic Retinopathy	Triacylglycerol (51:3)	Inverse variance weighted	4.798918	5	0.44091
**K96mi1**	Fyl8H6	Diabetic Retinopathy	Triacylglycerol (50:5)	MR Egger	0.146649	1	0.701759
**K96mi1**	Fyl8H6	Diabetic Retinopathy	Triacylglycerol (50:5)	Inverse variance	1.019417	2	0.600671

				weighted			
**2n0xIY**	TW6aB1	Diabetic Retinopathy	Triacylglycerol (50:3)	MR Egger	0.199178	1	0.655386
**2n0xIY**	TW6aB1	Diabetic Retinopathy	Triacylglycerol (50:3)	Inverse variance weighted	1.222742	2	0.542606
**uk8kGv**	VcgQ84	Diabetic Retinopathy	Triacylglycerol (50:4)	MR Egger	9.05E-05	1	0.992411
**uk8kGv**	VcgQ84	Diabetic Retinopathy	Triacylglycerol (50:4)	Inverse variance weighted	1.308979	2	0.519707
**0FQA5w**	vFaCv5	Diabetic Retinopathy	Triacylglycerol (56:8)	MR Egger	0.848899	3	0.837739
**0FQA5w**	vFaCv5	Diabetic Retinopathy	Triacylglycerol (56:8)	Inverse variance weighted	3.510608	4	0.476267
**Nki1Yj**	VmtgQY	Diabetic Retinopathy	Triacylglycerol (54:6)	MR Egger	0.166983	1	0.682807
**Nki1Yj**	VmtgQY	Diabetic Retinopathy	Triacylglycerol (54:6)	Inverse variance weighted	0.181934	2	0.913048
**TnDmSb**	HDtMyI	Diabetic Retinopathy	Sphingomyelin (d36:2)	MR Egger	1.585254	2	0.452654
**TnDmSb**	HDtMyI	Diabetic Retinopathy	Sphingomyelin (d36:2)	Inverse variance weighted	2.12039	3	0.547799
**WVQ2gk**	uqf4wh	Diabetic Retinopathy	Diacylglycerol (18:1_18:1)	MR Egger	1.829626	3	0.608509
**WVQ2gk**	uqf4wh	Diabetic Retinopathy	Diacylglycerol (18:1_18:1)	Inverse variance weighted	2.069982	4	0.722889
**TrtPa2**	vZkmNX	Diabetic Retinopathy	Triacylglycerol (52:3)	MR Egger	2.670961	4	0.614306
**TrtPa2**	vZkmNX	Diabetic Retinopathy	Triacylglycerol (52:3)	Inverse variance weighted	3.088195	5	0.686391
**i5F05p**	FhW29G	Diabetic Retinopathy	Triacylglycerol (52:5)	MR Egger	0.82455	2	0.662142
**i5F05p**	FhW29G	Diabetic Retinopathy	Triacylglycerol (52:5)	Inverse variance weighted	3.022338	3	0.388194
**45nC0u**	Qh2B95	Diabetic Retinopathy	Triacylglycerol (52:2)	MR Egger	2.550969	2	0.279296
**45nC0u**	Qh2B95	Diabetic Retinopathy	Triacylglycerol (52:2)	Inverse variance weighted	3.227296	3	0.35789
**6zzTBB**	xmknlS	Diabetic Retinopathy	Phosphatidylcholine (18:2_20:4)	MR Egger	2.909437	2	0.233466
**6zzTBB**	xmknlS	Diabetic Retinopathy	Phosphatidylcholine (18:2_20:4)	Inverse variance weighted	3.716646	3	0.293732
**DC7Vnd**	AGRZty	Diabetic Retinopathy	Sterol ester (27:1/18:3)	MR Egger	2.281581	3	0.516059
**DC7Vnd**	AGRZty	Diabetic Retinopathy	Sterol ester (27:1/18:3)	Inverse variance weighted	3.091935	4	0.54256
**zPK9E3**	pkSL2D	Diabetic Retinopathy	Phosphatidylinositol (16:0_20:4)	MR Egger	0.004384	1	0.947212
**zPK9E3**	pkSL2D	Diabetic Retinopathy	Phosphatidylinositol	Inverse variance	0.013977	2	0.993036

			(16:0_20:4)	weighted			
**iNRnRa**	mZ9qtc	Diabetic Retinopathy	Phosphatidylethanolamine (18:1_18:1)	MR Egger	6.58435	4	0.159553
**iNRnRa**	mZ9qtc	Diabetic Retinopathy	Phosphatidylethanolamine (18:1_18:1)	Inverse variance weighted	6.682943	5	0.245308
**HwBNPI**	1PTDAW	Diabetic Retinopathy	1-stearoyl-GPE (18:0)	MR Egger	2.618551	5	0.758545
**HwBNPI**	1PTDAW	Diabetic Retinopathy	1-stearoyl-GPE (18:0)	Inverse variance weighted	3.823035	6	0.70061
**ifjboY**	hydYxU	Diabetic Retinopathy	1-palmitoleoyl-GPC (16:1)	MR Egger	0.547278	2	0.760607
**ifjboY**	hydYxU	Diabetic Retinopathy	1-palmitoleoyl-GPC (16:1)	Inverse variance weighted	1.200563	3	0.752869
**kVjvuX**	Ut6oWB	Diabetic Retinopathy	5-hydroxylysine	MR Egger	2.936892	2	0.230283
**kVjvuX**	Ut6oWB	Diabetic Retinopathy	5-hydroxylysine	Inverse variance weighted	3.077375	3	0.379849
**gGZjiY**	td7C8N	Diabetic Retinopathy	2-stearoyl-GPE (18:0)	MR Egger	0.068805	1	0.793085
**gGZjiY**	td7C8N	Diabetic Retinopathy	2-stearoyl-GPE (18:0)	Inverse variance weighted	0.158108	2	0.92399
**w3WrlD**	4hFYiv	Diabetic Retinopathy	X-24544	MR Egger	4.044394	2	0.132364
**w3WrlD**	4hFYiv	Diabetic Retinopathy	X-24544	Inverse variance weighted	4.045657	3	0.256576
**C9AQJw**	dohi1W	Diabetic Retinopathy	X-26109	MR Egger	6.844168	4	0.144356
**C9AQJw**	dohi1W	Diabetic Retinopathy	X-26109	Inverse variance weighted	6.844441	5	0.232469
**j5ZPea**	0diN2w	Diabetic Retinopathy	1-palmitoyl-2-docosahexaenoyl-GPE (16:0/22:6)	MR Egger	1.887482	4	0.756444
**j5ZPea**	0diN2w	Diabetic Retinopathy	1-palmitoyl-2-docosahexaenoyl-GPE (16:0/22:6)	Inverse variance weighted	2.585736	5	0.763531
**CvKRL2**	oFTftw	Diabetic Retinopathy	Oleoyl-linoleoyl-glycerol (18:1/18:2) [2]	MR Egger	5.450868	5	0.363363
**CvKRL2**	oFTftw	Diabetic Retinopathy	Oleoyl-linoleoyl-glycerol (18:1/18:2) [2]	Inverse variance weighted	5.779808	6	0.448303
**MAgD91**	coYyr7	Diabetic Retinopathy	1-stearoyl-2-docosahexaenoyl-GPE (18:0/22:6)	MR Egger	1.397961	3	0.706013
**MAgD91**	coYyr7	Diabetic Retinopathy	1-stearoyl-2-docosahexaenoyl-GPE (18:0/22:6)	Inverse variance weighted	1.404566	4	0.843401
**LASv5h**	SODYTb	Diabetic Retinopathy	Metabolonic lactone sulfate	MR Egger	9.802383	7	0.200052

**LASv5h**	SODYTb	Diabetic Retinopathy	Metabolonic lactone sulfate	Inverse variance weighted	10.91363	8	0.206641
**31lzuG**	1HT8fv	Diabetic Retinopathy	1-palmitoyl-GPE (16:0)	MR Egger	1.318066	4	0.858303
**31lzuG**	1HT8fv	Diabetic Retinopathy	1-palmitoyl-GPE (16:0)	Inverse variance weighted	1.404607	5	0.923809
**6BKxSo**	MBRPkC	Diabetic Retinopathy	3-methylcytidine	MR Egger	1.324037	2	0.515809
**6BKxSo**	MBRPkC	Diabetic Retinopathy	3-methylcytidine	Inverse variance weighted	1.73347	3	0.629518
**5cDeDi**	yhEw55	Diabetic Retinopathy	1-stearoyl-gpc (18:0)	MR Egger	1.55168	1	0.212888
**5cDeDi**	yhEw55	Diabetic Retinopathy	1-stearoyl-gpc (18:0)	Inverse variance weighted	1.607937	2	0.447549
**YPOGmH**	65CuzL	Diabetic Retinopathy	(2 or 3)-decenoate (10:1n7 or n8)	MR Egger	0.233965	1	0.628599
**YPOGmH**	65CuzL	Diabetic Retinopathy	(2 or 3)-decenoate (10:1n7 or n8)	Inverse variance weighted	0.665985	2	0.716776
**kSTzk9**	VunMDW	Diabetic Retinopathy	Phosphate to oleoyl-linoleoyl-glycerol (18:1 to 18:2) [2]	MR Egger	3.574816	4	0.466594
**kSTzk9**	VunMDW	Diabetic Retinopathy	Phosphate to oleoyl-linoleoyl-glycerol (18:1 to 18:2) [2]	Inverse variance weighted	3.737901	5	0.587734
**YHG9kO**	UQyGwU	Diabetic Retinopathy	(S)-a-amino-omega-caprolactam	MR Egger	0.122601	1	0.726231
**YHG9kO**	UQyGwU	Diabetic Retinopathy	(S)-a-amino-omega-caprolactam	Inverse variance weighted	0.894218	2	0.639474
**YIxytW**	KWOfHz	Diabetic Retinopathy	16a-hydroxy DHEA 3-sulfate	MR Egger	1.066797	2	0.586608
**YIxytW**	KWOfHz	Diabetic Retinopathy	16a-hydroxy DHEA 3-sulfate	Inverse variance weighted	1.100249	3	0.777014
**K4SfSh**	eviNGY	Diabetic Retinopathy	N-stearoyl-sphingosine (d18:1 to 18:0) to N-palmitoyl-sphinganine (d18:0 to 16:0)	MR Egger	4.050388	3	0.256074
**K4SfSh**	eviNGY	Diabetic Retinopathy	N-stearoyl-sphingosine (d18:1 to 18:0) to N-palmitoyl-sphinganine (d18:0 to 16:0)	Inverse variance weighted	4.095041	4	0.393296
**hdrXzP**	TIFmxF	Diabetic Retinopathy	5alpha-androstan-3beta,17beta-diol disulfate	MR Egger	0.900758	3	0.825245
**hdrXzP**	TIFmxF	Diabetic Retinopathy	5alpha-androstan-3beta,17beta-diol disulfate	Inverse variance weighted	0.903449	4	0.924066
**fcJQqN**	q03RF0	Diabetic Retinopathy	Sphingomyelin (d18:1/20:1, d18:2/20:0)	MR Egger	0.481859	2	0.785897

**fcJQqN**	q03RF0	Diabetic Retinopathy	Sphingomyelin (d18:1/20:1, d18:2/20:0)	Inverse variance weighted	0.544396	3	0.909035
**PLHlWL**	5fFC2H	Diabetic Retinopathy	1-(1-enyl-palmitoyl)-2-palmitoyl-GPC (P-16:0/16:0)	MR Egger	7.326774	5	0.19745
**PLHlWL**	5fFC2H	Diabetic Retinopathy	1-(1-enyl-palmitoyl)-2-palmitoyl-GPC (P-16:0/16:0)	Inverse variance weighted	7.737149	6	0.258
**pYag6S**	0pM6w3	Diabetic Retinopathy	Glyco-beta-muricholate	MR Egger	0.488368	1	0.484656
**pYag6S**	0pM6w3	Diabetic Retinopathy	Glyco-beta-muricholate	Inverse variance weighted	0.526334	2	0.768614
**YBkqKq**	x760H2	Diabetic Retinopathy	1-palmitoyl-2-oleoyl-GPI (16:0/18:1)	MR Egger	1.06473	2	0.587214
**YBkqKq**	x760H2	Diabetic Retinopathy	1-palmitoyl-2-oleoyl-GPI (16:0/18:1)	Inverse variance weighted	1.094958	3	0.778291
**bxKoQE**	9UTfTP	Diabetic Retinopathy	Sphingomyelin (d18:1/18:1, d18:2/18:0)	MR Egger	1.474352	2	0.478463
**bxKoQE**	9UTfTP	Diabetic Retinopathy	Sphingomyelin (d18:1/18:1, d18:2/18:0)	Inverse variance weighted	1.475548	3	0.687925
**GRRaph**	ja5oi3	Diabetic Retinopathy	Homocitrulline	MR Egger	0.103978	2	0.949339
**GRRaph**	ja5oi3	Diabetic Retinopathy	Homocitrulline	Inverse variance weighted	0.334397	3	0.953435
**5xM8tN**	Lf89Eh	Diabetic Retinopathy	5alpha-androstan-3alpha,17alpha-diol mono-sulfate	MR Egger	1.692423	4	0.792094
**5xM8tN**	Lf89Eh	Diabetic Retinopathy	5alpha-androstan-3alpha,17alpha-diol mono-sulfate	Inverse variance weighted	4.571825	5	0.470331
**Ih7xQ7**	fQb79D	Diabetic Retinopathy	Epiandrosterone sulfate	MR Egger	1.592456	3	0.661102
**Ih7xQ7**	fQb79D	Diabetic Retinopathy	Epiandrosterone sulfate	Inverse variance weighted	1.706613	4	0.789516
**zBkdJi**	eCc5ol	Diabetic Retinopathy	Indoleacetylglutamine	MR Egger	2.043448	2	0.359974
**zBkdJi**	eCc5ol	Diabetic Retinopathy	Indoleacetylglutamine	Inverse variance weighted	2.09118	3	0.553699
**EVHw4G**	sPvrR7	Diabetic Retinopathy	1-palmitoyl-2-linoleoyl-GPI (16:0/18:2)	MR Egger	1.653875	3	0.647237
**EVHw4G**	sPvrR7	Diabetic Retinopathy	1-palmitoyl-2-linoleoyl-GPI (16:0/18:2)	Inverse variance weighted	1.709874	4	0.788923
**oruhso**	0SGyA4	Diabetic Retinopathy	Ceramide (d18:1/24:1)	MR Egger	0.356815	4	0.985857
**oruhso**	0SGyA4	Diabetic Retinopathy	Ceramide (d18:1/24:1)	Inverse variance weighted	0.410408	5	0.995038

**sVTua4**	pDVkcg	Diabetic Retinopathy	Dodecadienoate (12:2)	MR Egger	2.184225	3	0.535062
**sVTua4**	pDVkcg	Diabetic Retinopathy	Dodecadienoate (12:2)	Inverse variance weighted	2.224148	4	0.694611

Supplementary Table 6. Pleiotropy of immune cells, gut microbiota, inflammatory proteins, plasma lipidome, metabolites and diabetic retinopathy.

id.exposure	id.outcome	outcome	exposure	egger_intercept	se	pval
**IQdzWz**	sa5RQS	Diabetic Retinopathy	CD20 on IgD+ CD38dim B cell	-0.00874	0.046438	0.858082
**P1gIq6**	xGPxDn	Diabetic Retinopathy	CD25 on IgD- CD38- B cell	-0.05811	0.036927	0.360404
**W7f7y2**	eHuGhM	Diabetic Retinopathy	CD27 on CD24+ CD27+ B cell	-0.01935	0.012235	0.148317
**G9F1Bp**	UNhthV	Diabetic Retinopathy	CD27 on IgD- CD38dim B cell	-0.01933	0.012766	0.173828
**7Ag4jo**	oeq41x	Diabetic Retinopathy	CD27 on sw mem B cell	-0.01431	0.015928	0.395268
**qnXl7B**	0L7TPO	Diabetic Retinopathy	CD28 on CD28+ CD45RA+ CD8br Treg	-0.11539	0.085308	0.405284
**MRAjV7**	bc7B8W	Diabetic Retinopathy	CD45RA+ CD28- CD8br %T cell	-0.00318	0.010573	0.764662
**CZbABk**	tZJlRz	Diabetic Retinopathy	CD45RA+ CD28- CD8br AC Treg	-0.00537	0.003109	0.084869
**gdaaCY**	k8DGKF	Diabetic Retinopathy	CD80 on CD62L+ myeloid DC	-0.0118	0.032987	0.781226
**49RlmT**	o0Tcfs	Diabetic Retinopathy	CD80 on monocyte cDC	-0.0123	0.016643	0.501023
**225R5H**	9mFPpR	Diabetic Retinopathy	CD80 on myeloid DC	-0.01227	0.030641	0.727456
**zreERZ**	hJd1Yr	Diabetic Retinopathy	CX3CR1 on CD14+ CD16- mono-cyte	0.018293	0.044346	0.750931
**KvivHc**	CJAaTz	Diabetic Retinopathy	CX3CR1 on CD14+ CD16+ mon-ocyte	-0.00101	0.022505	0.971392
**i6akJV**	l0SteX	Diabetic Retinopathy	CX3CR1 on monocyte	0.010354	0.038012	0.830701
**8nFx2D**	D1uxkU	Diabetic Retinopathy	IgD on IgD+ CD38br B cell	0.003501	0.01285	0.798743
**UEhDkV**	U01D9h	Diabetic Retinopathy	IgD on transitional B cell	0.001986	0.017577	0.917169
**82ZVf5**	ArUaoV	Diabetic Retinopathy	Species Clostridium M sp001304855	0.021026	0.017479	0.274313
**LSuOnJ**	RX32r3	Diabetic Retinopathy	Species Pseudomonas aeruginosa	0.017279	0.014679	0.292119
**C5EmUe**	chqRE4	Diabetic Retinopathy	Unclassified Firmicutes A	-0.02395	0.021588	0.317706
**CJOz4c**	HMk589	Diabetic Retinopathy	Genus Holdemania	0.013391	0.016056	0.423746
**9PmFLE**	aRCG0o	Diabetic Retinopathy	Genus An181	0.015158	0.017512	0.435548
**j07qK9**	Twqs55	Diabetic Retinopathy	Unclassified Bacilli A	-0.01871	0.023888	0.463292
**Q9eu8n**	czUXKx	Diabetic Retinopathy	Species Blautia hansenii	0.008873	0.014634	0.55928
**L3qiCU**	tNf1rM	Diabetic Retinopathy	Unclassified Geobacter C	0.016354	0.028254	0.593748

**27WinM**	ASaoL1	Diabetic Retinopathy	Species Lachnospira rogosae	-0.00896	0.018665	0.644199
**M6q4iF**	NiuuUO	Diabetic Retinopathy	Species CAG-177 sp003538135	0.007377	0.017524	0.688448
**hZGxLU**	kVgIiB	Diabetic Retinopathy	Family Bifidobacteriaceae	-0.00571	0.020558	0.790487
**cvTKcn**	H21rHx	Diabetic Retinopathy	Unclassified CAG-475	0.00364	0.0148	0.809886
**qv20wE**	GcoB5g	Diabetic Retinopathy	Order Actinomycetales	0.008125	0.033213	0.829549
**1e82KN**	bLaFve	Diabetic Retinopathy	Species Bacteroides stercoris	-0.00365	0.022431	0.876951
**1VX5lC**	WSCHW9	Diabetic Retinopathy	FGF21	0.019517	0.034167	0.625474
**XuPoyn**	mZSALd	Diabetic Retinopathy	FGF19	0.014184	0.036415	0.763539
**bsBOzc**	SFfuED	Diabetic Retinopathy	Triacylglycerol (52:6)	0.019691	0.030282	0.582249
**8ji37G**	2ds89P	Diabetic Retinopathy	Triacylglycerol (54:7)	0.024233	0.031182	0.518399
**B8InWk**	cmbNsf	Diabetic Retinopathy	Triacylglycerol (52:4)	0.012934	0.0183	0.511292
**4iZDq0**	4H7aUp	Diabetic Retinopathy	Triacylglycerol (56:7)	0.023765	0.023266	0.382228
**TL6Xt2**	zdtFFn	Diabetic Retinopathy	Phosphatidylethanolamine (16:0_20:4)	0.02104	0.015982	0.279551
**mdVeuH**	pCQrL2	Diabetic Retinopathy	Triacylglycerol (51:3)	0.03098	0.02131	0.219674
**K96mi1**	Fyl8H6	Diabetic Retinopathy	Triacylglycerol (50:5)	0.049335	0.052809	0.521642
**2n0xIY**	TW6aB1	Diabetic Retinopathy	Triacylglycerol (50:3)	0.064625	0.063877	0.496293
**uk8kGv**	VcgQ84	Diabetic Retinopathy	Triacylglycerol (50:4)	0.085633	0.07485	0.457288
**0FQA5w**	vFaCv5	Diabetic Retinopathy	Triacylglycerol (56:8)	0.048463	0.029705	0.201289
**Nki1Yj**	VmtgQY	Diabetic Retinopathy	Triacylglycerol (54:6)	-0.00306	0.025035	0.922542
**TnDmSb**	HDtMyI	Diabetic Retinopathy	Sphingomyelin (d36:2)	-0.01991	0.027218	0.540557
**WVQ2gk**	uqf4wh	Diabetic Retinopathy	Diacylglycerol (18:1_18:1)	0.010505	0.021428	0.657566
**TrtPa2**	vZkmNX	Diabetic Retinopathy	Triacylglycerol (52:3)	0.011481	0.017774	0.55351
**i5F05p**	FhW29G	Diabetic Retinopathy	Triacylglycerol (52:5)	0.04041	0.027258	0.276426
**45nC0u**	Qh2B95	Diabetic Retinopathy	Triacylglycerol (52:2)	0.019927	0.027366	0.542218
**6zzTBB**	xmknlS	Diabetic Retinopathy	Phosphatidylcholine (18:2_20:4)	0.029439	0.03952	0.533966
**DC7Vnd**	AGRZty	Diabetic Retinopathy	Sterol ester (27:1/18:3)	0.01765	0.019606	0.434361
**zPK9E3**	pkSL2D	Diabetic Retinopathy	Phosphatidylinositol (16:0_20:4)	0.007382	0.075368	0.937844
**iNRnRa**	mZ9qtc	Diabetic Retinopathy	Phosphatidylethanolamine (18:1_18:1)	0.009463	0.038666	0.818703
**HwBNPI**	1PTDAW	Diabetic Retinopathy	1-stearoyl-GPE (18:0)	-0.02015	0.018363	0.322447
**ifjboY**	hydYxU	Diabetic Retinopathy	1-palmitoleoyl-GPC (16:1)	0.026301	0.03254	0.503797

**kVjvuX**	Ut6oWB	Diabetic Retinopathy	5-hydroxylysine	-0.01911	0.061792	0.786341
**gGZjiY**	td7C8N	Diabetic Retinopathy	2-stearoyl-GPE (18:0)	0.066257	0.221716	0.815133
**w3WrlD**	4hFYiv	Diabetic Retinopathy	X-24544	-0.00039	0.015745	0.982331
**C9AQJw**	dohi1W	Diabetic Retinopathy	X-26109	0.000158	0.012474	0.990525
**j5ZPea**	0diN2w	Diabetic Retinopathy	1-palmitoyl-2-docosahexaenoyl-GPE (16:0/22:6)	-0.01019	0.012193	0.450379
**CvKRL2**	oFTftw	Diabetic Retinopathy	Oleoyl-linoleoyl-glycerol (18:1/18:2) [2]	0.008473	0.015424	0.60643
**MAgD91**	coYyr7	Diabetic Retinopathy	1-stearoyl-2-docosahexaenoyl-GPE (18:0/22:6)	0.001441	0.017725	0.940344
**LASv5h**	SODYTb	Diabetic Retinopathy	Metabolonic lactone sulfate	0.010488	0.011773	0.402606
**31lzuG**	1HT8fv	Diabetic Retinopathy	1-palmitoyl-GPE (16:0)	0.007533	0.025608	0.783256
**6BKxSo**	MBRPkC	Diabetic Retinopathy	3-methylcytidine	0.013081	0.020444	0.587775
**5cDeDi**	yhEw55	Diabetic Retinopathy	1-stearoyl-gpc (18:0)	0.016054	0.084313	0.880216
**YPOGmH**	65CuzL	Diabetic Retinopathy	(2 or 3)-decenoate (10:1n7 or n8)	-0.03135	0.047693	0.62982
**kSTzk9**	VunMDW	Diabetic Retinopathy	Phosphate to oleoyl-linoleoyl-glycerol (18:1 to 18:2) [2]	-0.00644	0.015942	0.70699
**YHG9kO**	UQyGwU	Diabetic Retinopathy	(S)-a-amino-omega-caprolactam	0.026462	0.030124	0.541148
**YIxytW**	KWOfHz	Diabetic Retinopathy	16a-hydroxy DHEA 3-sulfate	0.00199	0.010878	0.871737
**K4SfSh**	eviNGY	Diabetic Retinopathy	N-stearoyl-sphingosine (d18:1 to 18:0) to N-palmitoyl-sphinganine (d18:0 to 16:0)	0.005217	0.028687	0.867287
**hdrXzP**	TIFmxF	Diabetic Retinopathy	5alpha-androstan-3beta,17beta-diol disulfate	0.000612	0.01179	0.961891
**fcJQqN**	q03RF0	Diabetic Retinopathy	Sphingomyelin (d18:1/20:1, d18:2/20:0)	-0.00463	0.018531	0.825872
**PLHlWL**	5fFC2H	Diabetic Retinopathy	1-(1-enyl-palmitoyl)-2-palmitoyl-GPC (P-16:0/16:0)	-0.0069	0.013044	0.619311
**pYag6S**	0pM6w3	Diabetic Retinopathy	Glyco-beta-muricholate	0.009003	0.046205	0.877491
**YBkqKq**	x760H2	Diabetic Retinopathy	1-palmitoyl-2-oleoyl-GPI (16:0/18:1)	-0.0057	0.032779	0.87798
**bxKoQE**	9UTfTP	Diabetic Retinopathy	Sphingomyelin (d18:1/18:1, d18:2/18:0)	-0.00083	0.02395	0.975546
**GRRaph**	ja5oi3	Diabetic Retinopathy	Homocitrulline	-0.00972	0.020247	0.678585
**5xM8tN**	Lf89Eh	Diabetic Retinopathy	5alpha-androstan-3alpha,17alpha-diol monosulfate	0.022404	0.013203	0.164957
**Ih7xQ7**	fQb79D	Diabetic Retinopathy	Epiandrosterone sulfate	-0.0039	0.011551	0.757721

**zBkdJi**	eCc5ol	Diabetic Retinopathy	Indoleacetylglutamine	0.0057	0.026371	0.848919
**EVHw4G**	sPvrR7	Diabetic Retinopathy	1-palmitoyl-2-linoleoyl-GPI (16:0/18:2)	-0.00512	0.021631	0.828173
**oruhso**	0SGyA4	Diabetic Retinopathy	Ceramide (d18:1/24:1)	0.003198	0.013816	0.828285
**sVTua4**	pDVkcg	Diabetic Retinopathy	Dodecadienoate (12:2)	-0.00328	0.01641	0.854409

Supplementary Table 7. Heterogeneity of immune cells, gut microbiota, plasma lipidome and metabolites in diabetic retinopathy.

id.exposure	id.outcome	outcome	exposure	method	Q	Q_df	Q_pval
**ebi-a-GCST90001752**	qAkveV	Unclassified Firmicutes A	CD20 on IgD+ CD38dim B cell	MR Egger	7.677337	5	0.174938
**ebi-a-GCST90001752**	qAkveV	Unclassified Firmicutes A	CD20 on IgD+ CD38dim B cell	Inverse variance weighted	7.67756	6	0.26269
**ebi-a-GCST90001700**	9cgFCQ	Unclassified CAG-475	CD45RA+ CD28- CD8br %T cell	MR Egger	31.25406	47	0.962548
**ebi-a-GCST90001700**	9cgFCQ	Unclassified CAG-475	CD45RA+ CD28- CD8br %T cell	Inverse variance weighted	31.31448	48	0.970164
**ebi-a-GCST90001700**	hO2Eh4	Species Clostridium M sp001304855	CD45RA+ CD28- CD8br %T cell	Inverse variance weighted	38.46489	48	0.835713
**ebi-a-GCST90001700**	hO2Eh4	Species Clostridium M sp001304855	CD45RA+ CD28- CD8br %T cell	MR Egger	37.05072	47	0.850595
**ebi-a-GCST90001997**	kg2CdM	Unclassified Bacilli A	CX3CR1 on CD14+ CD16- monocyte	Inverse variance weighted	2.179153	2	0.336359
**ebi-a-GCST90001997**	kg2CdM	Unclassified Bacilli A	CX3CR1 on CD14+ CD16- monocyte	MR Egger	0.009828	1	0.921031
**ebi-a-GCST90001997**	dDoicw	Species CAG-177 sp003538135	CX3CR1 on CD14+ CD16- monocyte	MR Egger	0.061349	1	0.804376
**ebi-a-GCST90001997**	dDoicw	Species CAG-177 sp003538135	CX3CR1 on CD14+ CD16- monocyte	Inverse variance weighted	0.067066	2	0.967023
**ebi-a-GCST90001996**	kg2CdM	Unclassified Bacilli A	CX3CR1 on CD14+ CD16+ monocyte	Inverse variance weighted	0.887348	2	0.641674
**ebi-a-GCST90001996**	kg2CdM	Unclassified Bacilli A	CX3CR1 on CD14+ CD16+ monocyte	MR Egger	0.193619	1	0.659922
**ebi-a-GCST90001996**	dDoicw	Species CAG-177 sp003538135	CX3CR1 on CD14+ CD16+ monocyte	Inverse variance weighted	0.335451	2	0.845586
**ebi-a-GCST90001996**	dDoicw	Species CAG-177 sp003538135	CX3CR1 on CD14+ CD16+ monocyte	MR Egger	0.027972	1	0.867176
**ebi-a-GCST90001995**	kg2CdM	Unclassified Bacilli A	CX3CR1 on mono-cyte	Inverse variance weighted	1.879898	2	0.390648
**ebi-a-GCST90001995**	kg2CdM	Unclassified Bacilli A	CX3CR1 on mono-cyte	MR Egger	0.045176	1	0.831681

**ebi-a-GCST90001995**	dDoicw	Species CAG-177 sp003538135	CX3CR1 on monocyte	MR Egger	0.097959	1	0.754292
**ebi-a-GCST90001995**	dDoicw	Species CAG-177 sp003538135	CX3CR1 on monocyte	Inverse variance weighted	0.141025	2	0.931916
**ebi-a-GCST90001828**	3Xezyd	Species Blautia hansenii	IgD on transitional B cell	Inverse variance weighted	4.429974	3	0.218622
**ebi-a-GCST90001828**	3Xezyd	Species Blautia hansenii	IgD on transitional B cell	MR Egger	1.629618	2	0.442724
**ebi-a-GCST90001787**	gOxg9i	1-stearoyl-gpc (18:0)	CD25 on IgD- CD38- B cell	Inverse variance weighted	0.7411	2	0.690355
**ebi-a-GCST90001787**	gOxg9i	1-stearoyl-gpc (18:0)	CD25 on IgD- CD38- B cell	MR Egger	0.669227	1	0.413321
**ebi-a-GCST90001808**	TAG0zE	Sphingomyelin (d18:1/18:1, d18:2/18:0)	CD27 on sw mem B cell	Inverse variance weighted	8.010918	9	0.533046
**ebi-a-GCST90001808**	TAG0zE	Sphingomyelin (d18:1/18:1, d18:2/18:0)	CD27 on sw mem B cell	MR Egger	7.236766	8	0.511316
**ebi-a-GCST90001700**	wCMWqI	16a-hydroxy DHEA 3-sulfate	CD45RA+ CD28- CD8br %T cell	Inverse variance weighted	61.81004	68	0.687987
**ebi-a-GCST90001700**	wCMWqI	16a-hydroxy DHEA 3-sulfate	CD45RA+ CD28- CD8br %T cell	MR Egger	61.66511	67	0.661176
**ebi-a-GCST90001700**	6zCzqz	Glyco-beta-muricholate	CD45RA+ CD28- CD8br %T cell	Inverse variance weighted	43.56974	68	0.990798
**ebi-a-GCST90001700**	6zCzqz	Glyco-beta-muricholate	CD45RA+ CD28- CD8br %T cell	MR Egger	43.23607	67	0.98937
**ebi-a-GCST90001698**	a2WqLz	1-palmitoyl-2-oleoyl-GPI (16:0/18:1)	CD45RA+ CD28- CD8br AC Treg	Inverse variance weighted	461.9175	428	0.124529
**ebi-a-GCST90001698**	a2WqLz	1-palmitoyl-2-oleoyl-GPI (16:0/18:1)	CD45RA+ CD28- CD8br AC Treg	MR Egger	460.0039	427	0.130466
**ebi-a-GCST90001700**	hSDaUT	Sphingomyelin (d36:2)	CD45RA+ CD28- CD8br %T cell	Inverse variance weighted	49.9017	57	0.736126
**ebi-a-GCST90001700**	hSDaUT	Sphingomyelin (d36:2)	CD45RA+ CD28- CD8br %T cell	MR Egger	49.63563	56	0.712988
**ebi-a-GCST90001700**	Qh7oiy	Triacylglycerol (56:7)	CD45RA+ CD28- CD8br %T cell	Inverse variance weighted	49.46168	57	0.750668
**ebi-a-GCST90001700**	Qh7oiy	Triacylglycerol (56:7)	CD45RA+ CD28- CD8br %T cell	MR Egger	48.90982	56	0.737869
**ebi-a-GCST90002039**	goDNzj	Triacylglycerol (56:8)	CD80 on monocyte cDC	Inverse variance weighted	2.75421	5	0.737816
**ebi-a-GCST90002039**	goDNzj	Triacylglycerol (56:8)	CD80 on monocyte cDC	MR Egger	2.693001	4	0.61044
**ebi-a-GCST90002039**	wxlV2R	Triacylglycerol (52:6)	CD80 on monocyte cDC	Inverse variance weighted	7.130968	5	0.211081

**ebi-a-GCST90002039**	wxlV2R	Triacylglycerol (52:6)	CD80 on monocyte cDC	MR Egger	6.042378	4	0.196006
**ebi-a-GCST90002039**	wYx68h	Phosphatidylinositol (16:0_20:4)	CD80 on monocyte cDC	Inverse variance weighted	1.558981	5	0.906165
**ebi-a-GCST90002039**	wYx68h	Phosphatidylinositol (16:0_20:4)	CD80 on monocyte cDC	MR Egger	1.218907	4	0.874976
**ebi-a-GCST90001997**	wxlV2R	Triacylglycerol (52:6)	CX3CR1 on CD14+ CD16- monocyte	Inverse variance weighted	1.084279	2	0.581503
**ebi-a-GCST90001997**	wxlV2R	Triacylglycerol (52:6)	CX3CR1 on CD14+ CD16- monocyte	MR Egger	0.029345	1	0.863985
**ebi-a-GCST90001996**	wxlV2R	Triacylglycerol (52:6)	CX3CR1 on CD14+ CD16+ monocyte	Inverse variance weighted	2.430712	2	0.296604
**ebi-a-GCST90001996**	wxlV2R	Triacylglycerol (52:6)	CX3CR1 on CD14+ CD16+ monocyte	MR Egger	0.055822	1	0.813226

Supplementary Table 8. Pleiotropy of immune cells, gut microbiota, plasma lipidome and metabolites in diabetic retinopathy.

id.exposure	id.outcome	outcome	exposure	egger_intercept	se	pval
**ebi-a-GCST90001752**	qAkveV	Unclassified Firmicutes A	CD20 on IgD+ CD38dim B cell	-0.00012	0.009759	0.990853
**ebi-a-GCST90001700**	hO2Eh4	Species Clostridium M sp001304855	CD45RA+ CD28- CD8br %T cell	-0.00711	0.005978	0.240338
**ebi-a-GCST90001700**	9cgFCQ	Unclassified CAG-475	CD45RA+ CD28- CD8br %T cell	0.002848	0.011585	0.806904
**ebi-a-GCST90001997**	kg2CdM	Unclassified Bacilli A	CX3CR1 on CD14+ CD16- monocyte	0.008623	0.005855	0.379717
**ebi-a-GCST90001997**	dDoicw	Species CAG-177 sp003538135	CX3CR1 on CD14+ CD16- monocyte	-0.00377	0.049921	0.951959
**ebi-a-GCST90001996**	kg2CdM	Unclassified Bacilli A	CX3CR1 on CD14+ CD16+ monocyte	0.002475	0.002972	0.557877
**ebi-a-GCST90001996**	dDoicw	Species CAG-177 sp003538135	CX3CR1 on CD14+ CD16+ monocyte	-0.01405	0.025341	0.677681
**ebi-a-GCST90001995**	kg2CdM	Unclassified Bacilli A	CX3CR1 on monocyte	0.006791	0.005014	0.404859
**ebi-a-GCST90001995**	dDoicw	Species CAG-177 sp003538135	CX3CR1 on monocyte	-0.00887	0.042751	0.869736
**ebi-a-GCST90001828**	3Xezyd	Species Blautia hansenii	IgD on transitional B cell	0.086244	0.051537	0.236217
**ebi-a-GCST90001787**	gOxg9i	1-stearoyl-gpc (18:0)	CD25 on IgD- CD38- B cell	0.011254	0.041977	0.833248
**ebi-a-GCST90001808**	TAG0zE	Sphingomyelin (d18:1/18:1, d18:2/18:0)	CD27 on sw mem B cell	0.014587	0.016578	0.40459
**ebi-a-GCST90001700**	wCMWqI	16a-hydroxy DHEA 3-	CD45RA+ CD28-	0.005627	0.014781	0.704624

		sulfate	CD8br %T cell			
**ebi-a-GCST90001700**	6zCzqz	Glyco-beta-muricholate	CD45RA+ CD28- CD8br %T cell	-0.00963	0.016667	0.565443
**ebi-a-GCST90001698**	a2WqLz	1-palmitoyl-2-oleoyl-GPI (16:0/18:1)	CD45RA+ CD28- CD8br AC Treg	0.005746	0.004311	0.183306
**ebi-a-GCST90001700**	hSDaUT	Sphingomyelin (d36:2)	CD45RA+ CD28- CD8br %T cell	-0.00698	0.013523	0.608011
**ebi-a-GCST90001700**	Qh7oiy	Triacylglycerol (56:7)	CD45RA+ CD28- CD8br %T cell	0.010076	0.013563	0.460664
**ebi-a-GCST90002039**	goDNzj	Triacylglycerol (56:8)	CD80 on monocyte cDC	-0.00542	0.021913	0.816775
**ebi-a-GCST90002039**	wxlV2R	Triacylglycerol (52:6)	CD80 on monocyte cDC	0.023313	0.027462	0.443753
**ebi-a-GCST90002039**	wYx68h	Phosphatidylinositol (16:0_20:4)	CD80 on monocyte cDC	-0.0142	0.024348	0.591083
**ebi-a-GCST90001997**	wxlV2R	Triacylglycerol (52:6)	CX3CR1 on CD14+ CD16- monocyte	0.061449	0.059828	0.49149
**ebi-a-GCST90001996**	wxlV2R	Triacylglycerol (52:6)	CX3CR1 on CD14+ CD16+ monocyte	0.046774	0.030352	0.36644

Supplementary Table 9. Heterogeneity of immune cells, gut microbiota, inflammatory proteins, plasma metabolites, micronutrients and diabetic neuropathy.

id.exposure	id.outcome	outcome	exposure	method	Q	Q_df	Q_pval
**Uaa00b**	cqMmIN	Diabetic Neuropathy	CD33dim HLA DR- AC Myeloid cell	MR Egger	0.094596	1	0.758413
**Uaa00b**	cqMmIN	Diabetic Neuropathy	CD33dim HLA DR- AC Myeloid cell	Inverse variance weighted	0.414615	2	0.81277
**C1I3Wx**	pZ9lK2	Diabetic Neuropathy	Basophil AC Myeloid cell	MR Egger	0.148662	1	0.699817
**C1I3Wx**	pZ9lK2	Diabetic Neuropathy	Basophil AC Myeloid cell	Inverse variance weighted	0.37723	2	0.828105
**pb3NSm**	ejvJec	Diabetic Neuropathy	Unclassified UBA8517	MR Egger	10.60143	7	0.156974
**pb3NSm**	ejvJec	Diabetic Neuropathy	Unclassified UBA8517	Inverse variance weighted	10.75669	8	0.21587
**nYFoOZ**	jkANud	Diabetic Neuropathy	Species Flavonifractor sp002159265	MR Egger	7.225518	7	0.405783
**fQTSMA**	U9Kq6N	Diabetic Neuropathy	Species Blautia sp000436935	MR Egger	11.76859	12	0.464439
**IP4Rpj**	iiCHBZ	Diabetic Neuropathy	Species CAG-273 sp003534295	MR Egger	4.560336	5	0.471852
**nYFoOZ**	jkANud	Diabetic Neuropathy	Species Flavonifractor	Inverse variance	7.375924	8	0.496669

			sp002159265	weighted			
**IP4Rpj**	iiCHBZ	Diabetic Neuropathy	Species CAG-273 sp003534295	Inverse variance weighted	5.042625	6	0.538358
**fQTSMA**	U9Kq6N	Diabetic Neuropathy	Species Blautia sp000436935	Inverse variance weighted	11.8073	13	0.543505
**jrWC33**	S63UE1	Diabetic Neuropathy	Genus Succinivibrio	Inverse variance weighted	17.19997	20	0.639953
**pfxhv2**	nIrSve	Diabetic Neuropathy	Species UBA1448	MR Egger	0.879024	2	0.644351
**Knkqpl**	VVh4Qs	Diabetic Neuropathy	Genus Enterococcus A	MR Egger	7.668141	10	0.661216
**jrWC33**	S63UE1	Diabetic Neuropathy	Genus Succinivibrio	MR Egger	15.85305	19	0.667059
**BHRmSw**	9pmIUW	Diabetic Neuropathy	Species GCA-900066135 sp900066135	MR Egger	3.185187	5	0.67146
**BHRmSw**	9pmIUW	Diabetic Neuropathy	Species GCA-900066135 sp900066135	Inverse variance weighted	3.802485	6	0.703385
**t3pZ7I**	21ocrb	Diabetic Neuropathy	Class Clostridia	Inverse variance weighted	2.900093	5	0.715385
**Knkqpl**	VVh4Qs	Diabetic Neuropathy	Genus Enterococcus A	Inverse variance weighted	7.806055	11	0.730565
**TZQXtB**	JQZFAe	Diabetic Neuropathy	Phylum Flavobacteriales	Inverse variance weighted	9.476781	13	0.736057
**pfxhv2**	nIrSve	Diabetic Neuropathy	Species UBA1448	Inverse variance weighted	1.093347	3	0.77868
**b3eRv2**	dFhA9B	Diabetic Neuropathy	Species Bacteroides A plebeius	Inverse variance weighted	4.771568	8	0.781689
**b3eRv2**	dFhA9B	Diabetic Neuropathy	Species Bacteroides A plebeius	MR Egger	3.847645	7	0.797149
**TZQXtB**	JQZFAe	Diabetic Neuropathy	Phylum Flavobacteriales	MR Egger	7.178412	12	0.845601
**t3pZ7I**	21ocrb	Diabetic Neuropathy	Class Clostridia	MR Egger	1.019317	4	0.906853
**DTRWO7**	5ioGU3	Diabetic Neuropathy	Species UBA1375 sp002305795	MR Egger	2.049488	6	0.915091
**DTRWO7**	5ioGU3	Diabetic Neuropathy	Species UBA1375 sp002305795	Inverse variance weighted	2.293678	7	0.941816
**URGVUV**	JiGuwT	Diabetic Neuropathy	TNFSF14	MR Egger	0.094273	2	0.953957
**URGVUV**	JiGuwT	Diabetic Neuropathy	TNFSF14	Inverse variance weighted	0.09777	3	0.992104
**NYToiX**	76QvJu	Diabetic Neuropathy	LIFR	MR Egger	0.024071	1	0.876705
**NYToiX**	76QvJu	Diabetic Neuropathy	LIFR	Inverse variance weighted	0.458793	2	0.795013
**8wBERf**	mHSaIA	Diabetic Neuropathy	CCL19	MR Egger	0.240213	1	0.624052

**8wBERf**	mHSaIA	Diabetic Neuropathy	CCL19	Inverse variance weighted	1.240587	2	0.537786
**JYwTo3**	tFS13s	Diabetic Neuropathy	Phosphate to serine	MR Egger	2.498355	1	0.113965
**JYwTo3**	tFS13s	Diabetic Neuropathy	Phosphate to serine	Inverse variance weighted	2.534873	2	0.281552
**fXJW1P**	uITbcg	Diabetic Neuropathy	Serine	MR Egger	2.794128	1	0.09461
**fXJW1P**	uITbcg	Diabetic Neuropathy	Serine	Inverse variance weighted	2.883872	2	0.236469
**Skbb8P**	VTal6Z	Diabetic Neuropathy	3-hydroxyhexanoylcarnitine (1)	MR Egger	0.234799	1	0.627988
**Skbb8P**	VTal6Z	Diabetic Neuropathy	3-hydroxyhexanoylcarnitine (1)	Inverse variance weighted	0.475147	2	0.788539
**r0WS7f**	T4qSFT	Diabetic Neuropathy	X-24556	MR Egger	0.285238	1	0.593288
**r0WS7f**	T4qSFT	Diabetic Neuropathy	X-24556	Inverse variance weighted	1.844069	2	0.397709
**kd2bJN**	pBxuqY	Diabetic Neuropathy	X-24328	MR Egger	5.796365	5	0.32654
**kd2bJN**	pBxuqY	Diabetic Neuropathy	X-24328	Inverse variance weighted	6.51649	6	0.367881
**T0OZ9C**	DR4wvS	Diabetic Neuropathy	3-hydroxybutyroylglycine	MR Egger	0.815644	1	0.366457
**T0OZ9C**	DR4wvS	Diabetic Neuropathy	3-hydroxybutyroylglycine	Inverse variance weighted	0.905423	2	0.635902
**DgrTR9**	2FR7Pr	Diabetic Neuropathy	N-acetylputrescine to (N(1) + N(8))-acetylspermidine	MR Egger	3.849225	3	0.278212
**DgrTR9**	2FR7Pr	Diabetic Neuropathy	N-acetylputrescine to (N(1) + N(8))-acetylspermidine	Inverse variance weighted	4.394393	4	0.355254
**RSlg0v**	X5afoD	Diabetic Neuropathy	2-butenoylglycine	MR Egger	1.431986	1	0.231441
**RSlg0v**	X5afoD	Diabetic Neuropathy	2-butenoylglycine	Inverse variance weighted	1.528799	2	0.465614
**yVATYA**	iMwxGT	Diabetic Neuropathy	1-oleoyl-2-docosahexaenoyl-GPC (18:1/22:6)	MR Egger	5.756007	3	0.124104
**yVATYA**	iMwxGT	Diabetic Neuropathy	1-oleoyl-2-docosahexaenoyl-GPC (18:1/22:6)	Inverse variance weighted	5.756379	4	0.218096
**mROcZV**	qGKbki	Diabetic Neuropathy	X-26054	MR Egger	3.706409	2	0.156734
**mROcZV**	qGKbki	Diabetic Neuropathy	X-26054	Inverse variance weighted	3.715827	3	0.29383

**3TrZD0**	JnttZ5	Diabetic Neuropathy	Indolelactate	MR Egger	0.687677	1	0.406956
**3TrZD0**	JnttZ5	Diabetic Neuropathy	Indolelactate	Inverse variance weighted	0.699598	2	0.70483
**mQRmKb**	DNEjUW	Diabetic Neuropathy	Paraxanthine to 5-acetylamino-6-formylamino-3-methyluracil	MR Egger	1.983844	2	0.370863
**mQRmKb**	DNEjUW	Diabetic Neuropathy	Paraxanthine to 5-acetylamino-6-formylamino-3-methyluracil	Inverse variance weighted	2.186575	3	0.534597
**UgbHYW**	1PAIdd	Diabetic Neuropathy	Argininate	MR Egger	0.680302	3	0.877827
**UgbHYW**	1PAIdd	Diabetic Neuropathy	Argininate	Inverse variance weighted	0.93249	4	0.919854
**5zSqTX**	nnzK4O	Diabetic Neuropathy	Orotate	MR Egger	0.131314	2	0.936452
**5zSqTX**	nnzK4O	Diabetic Neuropathy	Orotate	Inverse variance weighted	0.48709	3	0.921718
**Tr50ef**	4csuTh	Diabetic Neuropathy	X-25433	MR Egger	1.783639	1	0.181703
**Tr50ef**	4csuTh	Diabetic Neuropathy	X-25433	Inverse variance weighted	1.829808	2	0.400555
**itJClP**	jo2Vm5	Diabetic Neuropathy	Carnitine	MR Egger	0.898868	2	0.637989
**itJClP**	jo2Vm5	Diabetic Neuropathy	Carnitine	Inverse variance weighted	1.812413	3	0.612237
**U1UQVy**	KvXaPm	Diabetic Neuropathy	Carotene	MR Egger	6.111989	13	0.941982
**U1UQVy**	KvXaPm	Diabetic Neuropathy	Carotene	Inverse variance weighted	6.589818	14	0.94937

Supplementary Table 10. Pleiotropy of immune cells, gut microbiota, inflammatory proteins, plasma metabolites, micronutrients and diabetic neuropathy.

**id.exposure**	**id.outcome**	**outcome**	**exposure**	**egger_intercept**	**se**	**pval**
**Uaa00b**	cqMmIN	Diabetic Neuropathy	CD33dim HLA DR- AC Myeloid cell	-0.01622	0.028674	0.672256
**C1I3Wx**	pZ9lK2	Diabetic Neuropathy	Basophil AC Myeloid cell	-0.01381	0.028881	0.71609
**TZQXtB**	JQZFAe	Diabetic Neuropathy	Phylum Flavobacteriales	0.029177	0.019246	0.155401
**t3pZ7I**	21ocrb	Diabetic Neuropathy	Class Clostridia	-0.05515	0.040212	0.242146
**jrWC33**	S63UE1	Diabetic Neuropathy	Genus Succinivibrio	0.025418	0.021902	0.260193
**b3eRv2**	dFhA9B	Diabetic Neuropathy	Species Bacteroides A plebeius	-0.02782	0.028943	0.368466
**BHRmSw**	9pmIUW	Diabetic Neuropathy	Species GCA-900066135	-0.02267	0.028851	0.467633

			sp900066135			
**IP4Rpj**	iiCHBZ	Diabetic Neuropathy	Species CAG-273 sp003534295	-0.02141	0.030834	0.518327
**DTRWO7**	5ioGU3	Diabetic Neuropathy	Species UBA1375 sp002305795	0.012664	0.025627	0.638765
**pfxhv2**	nIrSve	Diabetic Neuropathy	Species UBA1448	-0.05151	0.11126	0.68889
**nYFoOZ**	jkANud	Diabetic Neuropathy	Species Flavonifractor sp002159265	0.014543	0.038098	0.71399
**Knkqpl**	VVh4Qs	Diabetic Neuropathy	Genus Enterococcus A	0.008192	0.02206	0.71811
**pb3NSm**	ejvJec	Diabetic Neuropathy	Unclassified UBA8517	0.010034	0.031337	0.758176
**fQTSMA**	U9Kq6N	Diabetic Neuropathy	Species Blautia sp000436935	-0.00514	0.026132	0.847314
**URGVUV**	JiGuwT	Diabetic Neuropathy	TNFSF14	0.001807	0.030549	0.958222
**NYToiX**	76QvJu	Diabetic Neuropathy	LIFR	0.022436	0.034028	0.628908
**8wBERf**	mHSaIA	Diabetic Neuropathy	CCL19	-0.04483	0.044822	0.49994
**U1UQVy**	KvXaPm	Diabetic Neuropathy	Carotene	0.013886	0.020089	0.501566

id.exposure	id.outcome	outcome	exposure	method	nsnp	b	se	pval	lo_ci	up_ci	or	or_lci95	or_uci95	FDR
ebi-a-GCST90001697	Ut2VMm	Diabetic Nephropathy	CD45RA- CD28- CD8br %T cell	IVW	41	-0.00103	0.000389	0.00833	-0.00179	-0.00026	0.998973	0.998211	0.999736	0.495973
ebi-a-GCST90001869	Ut2VMm	Diabetic Nephropathy	CD3 on resting Treg	IVW	6	0.050406	0.024762	0.041788	0.001873	0.09894	1.051698	1.001874	1.104	0.867392
tXchOq	L8xX0y	Diabetic Nephropathy	Family Enterobacteriaceae	IVW	10	-0.28135	0.096553	0.003569	-0.4706	-0.09211	0.754762	0.624629	0.912006	0.999206
mBXqB5	L8xX0y	Diabetic Nephropathy	Genus Desulfovibrionia	IVW	6	0.333647	0.137283	0.015083	0.064574	0.602721	1.396051	1.066704	1.827084	0.999206
2rt317	L8xX0y	Diabetic Nephropathy	Genus Citrobacter A	IVW	8	0.45902	0.195541	0.018903	0.07576	0.842281	1.582523	1.078703	2.321657	0.999206
reZW6G	L8xX0y	Diabetic Nephropathy	Genus Syntrophorhabdia	IVW	6	-1.21303	0.535879	0.023597	-2.26336	-0.16271	0.297294	0.104001	0.849838	0.999206
qcoHvy	L8xX0y	Diabetic Nephropathy	Genus Leuconostoc	IVW	5	0.358675	0.162801	0.027584	0.039585	0.677764	1.431431	1.040379	1.96947	0.999206
uRGp1t	L8xX0y	Diabetic Nephropathy	Family Desulfovibrionaceae	IVW	8	0.211657	0.100639	0.035455	0.014404	0.40891	1.235724	1.014508	1.505177	0.999206
zL970M	L8xX0y	Diabetic Nephropathy	Unclassified CAG-145	IVW	5	0.346362	0.169805	0.041374	0.013544	0.679179	1.413914	1.013636	1.972259	0.999206
GCST90277308	miIBxl	Diabetic Nephropathy	Phosphatidylcholine (18:1_18:1)	IVW	4	0.239854	0.07447	0.001278	0.093893	0.385814	1.271063	1.098443	1.470812	0.167939
GCST90277366	miIBxl	Diabetic Nephropathy	Phosphatidylinositol (18:1_18:2)	IVW	3	0.240176	0.08575	0.005096	0.072106	0.408247	1.271473	1.074769	1.504179	0.167939

GCST90277298	miIBxl	Diabetic Nephropathy	Phosphatidylcholine (17:0_20:4)	IVW	6	-0.08939	0.034108	0.008773	-0.15624	-0.02254	0.91449	0.855354	0.977714	0.167939
GCST90277282	miIBxl	Diabetic Nephropathy	Phosphatidylcholine (16:0_18:2)	IVW	7	0.118043	0.045981	0.010252	0.02792	0.208166	1.125293	1.028314	1.231417	0.171718
GCST90277309	miIBxl	Diabetic Nephropathy	Phosphatidylcholine (18:1_18:2)	IVW	5	0.120825	0.048422	0.012586	0.025919	0.215731	1.128427	1.026257	1.240769	0.187394
GCST90277313	miIBxl	Diabetic Nephropathy	Phosphatidylcholine (18:1_20:4)	IVW	7	-0.0847	0.035334	0.016521	-0.15396	-0.01545	0.918785	0.857308	0.984671	0.206668
GCST90277287	miIBxl	Diabetic Nephropathy	Phosphatidylcholine (16:0_20:4)	IVW	5	-0.07137	0.031591	0.023869	-0.13329	-0.00945	0.931117	0.875213	0.990592	0.206668
GCST90277345	miIBxl	Diabetic Nephropathy	Phosphatidylethanolamine (16:0_18:2)	IVW	6	0.083611	0.037358	0.025216	0.010389	0.156833	1.087206	1.010443	1.1698	0.206668
GCST90277330	miIBxl	Diabetic Nephropathy	Phosphatidylcholine (O-16:1_20:4)	IVW	3	-0.09326	0.043853	0.033453	-0.17921	-0.00731	0.910958	0.83593	0.99272	0.249036
GCST90277276	miIBxl	Diabetic Nephropathy	Phosphatidylcholine (15:0_18:2)	IVW	6	0.116421	0.057137	0.041593	0.004432	0.22841	1.123469	1.004442	1.256601	0.253341
GCST90277247	miIBxl	Diabetic Nephropathy	Sterol ester (27:1/18:3)	IVW	5	-0.14094	0.071288	0.048044	-0.28066	-0.00121	0.868545	0.755284	0.998791	0.274813
YRvUi4	iSWEwg	Diabetic Nephropathy	Retinol (Vitamin A) to linoleoyl-arachidonoyl-glycerol (18:2 to 20:4) [2]	IVW	3	0.148477	0.055282	0.007235	0.040124	0.25683	1.160066	1.04094	1.292825	0.494248
coUkT0	iSWEwg	Diabetic Nephropathy	5,6-dihydrouridine	IVW	4	-0.2612	0.100979	0.009689	-0.45912	-0.06329	0.770124	0.631838	0.938675	0.494248
rv3dxu	iSWEwg	Diabetic Nephropathy	Metabolonic lactone sulfate	IVW	9	-0.07155	0.028159	0.011052	-0.12675	-0.01636	0.930946	0.880957	0.983771	0.494248
Snu3Xv	iSWEwg	Diabetic Nephropathy	X-25371	IVW	4	-0.16844	0.066732	0.011598	-0.29924	-0.03765	0.84498	0.741384	0.963053	0.494248
uarAxE	iSWEwg	Diabetic Nephropathy	Aspartate to N-acetylglucosamine to N-acetylgalactosamine	IVW	3	-0.19288	0.07873	0.014287	-0.34719	-0.03857	0.824577	0.706668	0.96216	0.494248
ra3RHU	iSWEwg	Diabetic Nephropathy	Hydroxypalmitoyl sphingomyelin (d18:1/16:0(OH))	IVW	3	0.160493	0.065948	0.014948	0.031235	0.289752	1.17409	1.031728	1.336096	0.494248
MTO5rN	iSWEwg	Diabetic Nephropathy	1,2-dilinoleoyl-GPE (18:2/18:2)	IVW	3	0.172872	0.072026	0.016389	0.031702	0.314042	1.188714	1.03221	1.368947	0.494248
1eMa1l	iSWEwg	Diabetic Nephropathy	1-(1-enyl-stearoyl)-2-linoleoyl-GPE (p-18:0/18:2)	IVW	3	0.19777	0.08343	0.017765	0.034247	0.361293	1.218682	1.03484	1.435183	0.494248
Wt3ZoP	iSWEwg	Diabetic Nephropathy	1-(1-enyl-palmitoyl)-GPC (p-16:0)	IVW	3	-0.20641	0.088179	0.019241	-0.37924	-0.03358	0.813498	0.68438	0.966977	0.494248
n91Tzn	iSWEwg	Diabetic Nephropathy	1-(1-enyl-palmitoyl)-2-arachidonoyl-gpc (p-16:0/20:4)	IVW	3	-0.08788	0.037772	0.01999	-0.16191	-0.01384	0.915874	0.850519	0.986251	0.494248
JpLbOz	iSWEwg	Diabetic Nephropathy	X-26109	IVW	6	-0.06964	0.030051	0.020475	-0.12854	-0.01074	0.932726	0.879375	0.989313	0.494248
Dehpjt	iSWEwg	Diabetic Nephropathy	1-stearoyl-2-docosahexaenoyl-gpc (18:0/22:6)	IVW	3	-0.17499	0.075525	0.020507	-0.32302	-0.02696	0.839467	0.723961	0.973402	0.494248
qpoBMf	iSWEwg	Diabetic Nephropathy	1-stearoyl-2-linoleoyl-gpc (18:0/18:2)	IVW	3	0.177585	0.078064	0.022913	0.024581	0.33059	1.19433	1.024885	1.391789	0.494248
NXkYyo	iSWEwg	Diabetic Nephropathy	1-oleoyl-2-linoleoyl-GPE (18:1/18:2)	IVW	5	0.089571	0.039492	0.023324	0.012167	0.166974	1.093705	1.012241	1.181724	0.494248

qaoUHO	iSWEwg	Diabetic Nephropathy	Cholesterol to linoleoyl-arachidonoyl-glycerol (18:2 to 20:4) [1]	IVW	3	0.140721	0.062066	0.023373	0.019072	0.262371	1.151104	1.019255	1.300009	0.494248
v8PJsP	iSWEwg	Diabetic Nephropathy	Retinol (Vitamin A) to linoleoyl-arachidonoyl-glycerol (18:2 to 20:4) [1]	IVW	4	0.127688	0.056574	0.024008	0.016803	0.238574	1.136199	1.016945	1.269437	0.494248
DSygCD	iSWEwg	Diabetic Nephropathy	1-arachidonoyl-GPE (20:4n6)	IVW	4	-0.09501	0.0421	0.024029	-0.17752	-0.01249	0.909367	0.837341	0.987588	0.494248
mTmMwi	iSWEwg	Diabetic Nephropathy	Dodecadienoate (12:2)	IVW	5	0.180195	0.079879	0.02408	0.023632	0.336759	1.197451	1.023913	1.400401	0.494248
8XQY7L	iSWEwg	Diabetic Nephropathy	Phosphate to linoleoyl-arachidonoyl-glycerol (18:2 to 20:4) [1]	IVW	3	0.127851	0.057231	0.025487	0.015678	0.240024	1.136384	1.015801	1.27128	0.494248
y0DlUg	iSWEwg	Diabetic Nephropathy	1-arachidonoyl-gpc (20:4n6)	IVW	5	-0.08258	0.03753	0.027772	-0.15614	-0.00903	0.920734	0.855438	0.991015	0.494248
sFTSwZ	iSWEwg	Diabetic Nephropathy	X-26111	IVW	5	-0.13309	0.060666	0.028254	-0.25199	-0.01418	0.87539	0.777251	0.98592	0.494248
6XVwdY	iSWEwg	Diabetic Nephropathy	1-palmitoyl-2-linoleoyl-GPI (16:0/18:2)	IVW	5	0.170117	0.079058	0.031413	0.015164	0.32507	1.185443	1.015279	1.384128	0.494248
XBLY8d	iSWEwg	Diabetic Nephropathy	N-stearoyl-sphingosine (d18:1 to 18:0) to N-palmitoyl-sphinganine (d18:0 to 16:0)	IVW	5	-0.16461	0.07679	0.032066	-0.31511	-0.0141	0.848229	0.729706	0.986002	0.494248
fz6Z1H	iSWEwg	Diabetic Nephropathy	Vanillic acid glycine	IVW	4	-0.19579	0.091343	0.032077	-0.37482	-0.01676	0.822185	0.687411	0.983382	0.494248
JGu6Jo	iSWEwg	Diabetic Nephropathy	1-linoleoyl-GPG (18:2)	IVW	4	-0.09868	0.046442	0.033604	-0.18971	-0.00765	0.906033	0.827203	0.992376	0.494248
mPvTc3	iSWEwg	Diabetic Nephropathy	Tetradecanedioate (C14-DC)	IVW	6	-0.08116	0.038844	0.036664	-0.1573	-0.00503	0.922042	0.854448	0.994983	0.494248
XxxFFN	iSWEwg	Diabetic Nephropathy	X-18922	IVW	6	-0.11552	0.055723	0.038168	-0.22473	-0.0063	0.890907	0.798731	0.993721	0.494248
sHOk2I	iSWEwg	Diabetic Nephropathy	Hexadecenedioate (C16:1-DC)	IVW	4	-0.06593	0.031844	0.038424	-0.12834	-0.00351	0.9362	0.879555	0.996494	0.494248
MZUqvW	iSWEwg	Diabetic Nephropathy	Caffeine to paraxanthine	IVW	3	-0.15149	0.073748	0.039953	-0.29604	-0.00695	0.859423	0.743758	0.993075	0.494248
BmdUs4	iSWEwg	Diabetic Nephropathy	1-oleoyl-GPG (18:1)	IVW	5	-0.09019	0.044056	0.040639	-0.17654	-0.00384	0.913756	0.838164	0.996166	0.494248
WpyQmq	iSWEwg	Diabetic Nephropathy	N2-acetyl,N6,N6-dimethyllysine	IVW	6	0.052065	0.025504	0.041205	0.002078	0.102052	1.053444	1.00208	1.107441	0.494248
cTiRBD	iSWEwg	Diabetic Nephropathy	21-hydroxypregnenolone disulfate	IVW	7	-0.13305	0.065272	0.041513	-0.26098	-0.00512	0.875423	0.770295	0.994898	0.494248
2mQRTz	iSWEwg	Diabetic Nephropathy	Sphingomyelin (d18:2/21:0, d16:2/23:0)	IVW	4	-0.1998	0.099102	0.043794	-0.39404	-0.00556	0.818898	0.67433	0.99446	0.494248
sRrNSM	iSWEwg	Diabetic Nephropathy	1-palmitoyl-2-linoleoyl-GPE (16:0/18:2)	IVW	6	0.065678	0.032954	0.046257	0.001089	0.130267	1.067883	1.001089	1.139132	0.497704
gqZODE	iSWEwg	Diabetic Nephropathy	5-methylthioadenosine (MTA) to phosphate	IVW	4	0.207452	0.105384	0.049007	0.000899	0.414005	1.230539	1.000899	1.512865	0.497704
SHDhX0	iSWEwg	Diabetic Nephropathy	Linoleoyl-arachidonoyl-glycerol (18:2/20:4) [1]	IVW	3	-0.10365	0.052833	0.049779	-0.20721	-9.80E-05	0.901539	0.812853	0.999902	0.497704
ieu-a-1077	AosMqV	Diabetic Nephropathy	Selenium	IVW	6	-0.09601	0.044382	0.030521	-0.183	-0.00902	0.908456	0.832771	0.991019	0.457813

ebi-a-GCST90001787	WISnV1	Diabetic Retinopathy	CD25 on IgD- CD38- B cell	IVW	3	0.137703	0.045215	0.002323	0.049082	0.226324	1.147635	1.050307	1.253982	0.128906
ebi-a-GCST90001798	WISnV1	Diabetic Retinopathy	CD27 on CD24+ CD27+ B cell	IVW	11	-0.05445	0.018796	0.00377	-0.09129	-0.01761	0.947008	0.912755	0.982546	0.136757
ebi-a-GCST90001896	WISnV1	Diabetic Retinopathy	CD28 on CD28+ CD45RA+ CD8br Treg	IVW	3	0.093392	0.032266	0.003798	0.030151	0.156633	1.097892	1.03061	1.169566	0.136757
ebi-a-GCST90001808	WISnV1	Diabetic Retinopathy	CD27 on sw mem B cell	IVW	10	-0.0506	0.017714	0.00428	-0.08532	-0.01588	0.950655	0.918216	0.984241	0.136757
ebi-a-GCST90001752	WISnV1	Diabetic Retinopathy	CD20 on IgD+ CD38dim B cell	IVW	7	0.109125	0.038231	0.004312	0.034193	0.184058	1.115302	1.034784	1.202085	0.136757
ebi-a-GCST90002039	WISnV1	Diabetic Retinopathy	CD80 on monocyte cDC	IVW	6	0.054759	0.021416	0.010561	0.012783	0.096735	1.056286	1.012865	1.101568	0.264644
ebi-a-GCST90001804	WISnV1	Diabetic Retinopathy	CD27 on IgD- CD38dim B cell	IVW	9	-0.04677	0.018331	0.010729	-0.0827	-0.01084	0.954306	0.920627	0.989217	0.264644
ebi-a-GCST90001996	WISnV1	Diabetic Retinopathy	CX3CR1 on CD14+ CD16+ monocyte	IVW	3	-0.06113	0.025577	0.01685	-0.11126	-0.011	0.940703	0.894707	0.989063	0.298233
ebi-a-GCST90001997	WISnV1	Diabetic Retinopathy	CX3CR1 on CD14+ CD16- monocyte	IVW	3	-0.05463	0.023378	0.019461	-0.10045	-0.0088	0.94684	0.904433	0.991235	0.299053
ebi-a-GCST90001995	WISnV1	Diabetic Retinopathy	CX3CR1 on monocyte	IVW	3	-0.06011	0.025739	0.019533	-0.11055	-0.00966	0.941665	0.895339	0.990389	0.299053
ebi-a-GCST90001698	WISnV1	Diabetic Retinopathy	CD45RA+ CD28- CD8br AC Treg	IVW	426	8.05E-06	3.53E-06	0.022501	1.14E-06	1.50E-05	1.000008	1.000001	1.000015	0.322896
ebi-a-GCST90002035	WISnV1	Diabetic Retinopathy	CD80 on myeloid DC	IVW	4	0.082194	0.036033	0.022545	0.011569	0.152818	1.085666	1.011636	1.165113	0.322896
ebi-a-GCST90002036	WISnV1	Diabetic Retinopathy	CD80 on CD62L+ myeloid DC	IVW	3	0.091145	0.040189	0.023335	0.012374	0.169915	1.095428	1.012451	1.185204	0.323777
ebi-a-GCST90001824	WISnV1	Diabetic Retinopathy	IgD on IgD+ CD38br B cell	IVW	6	-0.03706	0.016641	0.025935	-0.06968	-0.00445	0.963615	0.932692	0.995564	0.338681
ebi-a-GCST90001828	WISnV1	Diabetic Retinopathy	IgD on transitional B cell	IVW	5	-0.04653	0.021601	0.031217	-0.08887	-0.0042	0.954532	0.914963	0.995812	0.396013
ebi-a-GCST90001700	WISnV1	Diabetic Retinopathy	CD45RA+ CD28- CD8br %T cell	IVW	67	0.000359	0.000169	0.034251	2.66E-05	0.00069	1.000359	1.000027	1.000691	0.411008
A8IUc9	Gr249B	Diabetic Retinopathy	Unclassified Bacilli A	IVW	8	1.959912	0.664374	0.003178	0.657739	3.262085	7.098701	1.930423	26.1039	0.983698
fS7KV7	Gr249B	Diabetic Retinopathy	Family Bifidobacteriaceae	IVW	8	-0.15672	0.056372	0.005433	-0.26721	-0.04624	0.85494	0.76551	0.954817	0.983698
RzQClC	Gr249B	Diabetic Retinopathy	Species CAG-177 sp003538135	IVW	8	0.182157	0.066656	0.00628	0.051511	0.312803	1.199802	1.052861	1.367252	0.983698

D1XMKf	Gr249B	Diabetic Retinopathy	Genus An181	IVW	6	0.331333	0.128306	0.009812	0.079855	0.582812	1.392824	1.08313	1.791068	0.983698
COcXXQ	Gr249B	Diabetic Retinopathy	Species Pseudomonas aeruginosa	IVW	7	0.463317	0.187802	0.013623	0.095224	0.831409	1.589337	1.099905	2.296553	0.983698
7JQHVW	Gr249B	Diabetic Retinopathy	Species Lachnospira rogosae	IVW	10	0.129634	0.05303	0.014504	0.025695	0.233572	1.138411	1.026028	1.263104	0.983698
h1l8Fe	Gr249B	Diabetic Retinopathy	Species Blautia hansenii	IVW	11	0.194747	0.081391	0.016724	0.03522	0.354274	1.215004	1.035848	1.425146	0.983698
MY5ejN	Gr249B	Diabetic Retinopathy	Genus Holdemania	IVW	12	0.150078	0.066286	0.023569	0.020156	0.279999	1.161925	1.020361	1.323129	0.983698
2ZKx87	Gr249B	Diabetic Retinopathy	Unclassified Geobacter C	IVW	6	0.5319	0.237508	0.025123	0.066385	0.997416	1.702164	1.068638	2.711266	0.983698
GOJ1Bi	Gr249B	Diabetic Retinopathy	Species Bacteroides stercoris	IVW	7	-0.13175	0.061136	0.031162	-0.25157	-0.01192	0.876562	0.777576	0.98815	0.983698
XxnkAU	Gr249B	Diabetic Retinopathy	Unclassified Firmicutes A	IVW	7	0.649523	0.305134	0.033284	0.05146	1.247586	1.914627	1.052807	3.481928	0.983698
UcIDhn	Gr249B	Diabetic Retinopathy	Species Clostridium M sp001304855	IVW	8	-0.2536	0.126989	0.045823	-0.5025	-0.0047	0.776002	0.605017	0.995309	0.983698
qH71DW	Gr249B	Diabetic Retinopathy	Order Actinomycetales	IVW	4	-0.18097	0.090686	0.045979	-0.35872	-0.00323	0.834458	0.698571	0.996778	0.983698
tU2Sl8	Gr249B	Diabetic Retinopathy	Unclassified CAG-475	IVW	14	-0.10034	0.050739	0.047976	-0.19979	-0.00089	0.90453	0.818904	0.999108	0.983698
GCST90274788	Z5sxMQ	Diabetic Retinopathy	FGF21	IVW	4	-0.12294	0.054748	0.02473	-0.23025	-0.01564	0.884316	0.794338	0.984486	0.362116
GCST90274787	Z5sxMQ	Diabetic Retinopathy	FGF19	IVW	3	-0.13436	0.063246	0.033635	-0.25832	-0.0104	0.874275	0.772347	0.989655	0.362116
GCST90277400	9ATSFc	Diabetic Retinopathy	Triacylglycerol (52:6)	IVW	4	-0.16359	0.050107	0.001096	-0.2618	-0.06538	0.849091	0.769666	0.936713	0.076104
GCST90277408	9ATSFc	Diabetic Retinopathy	Triacylglycerol (54:7)	IVW	4	-0.18338	0.058448	0.001704	-0.29794	-0.06882	0.832449	0.742344	0.933491	0.076104
GCST90277398	9ATSFc	Diabetic Retinopathy	Triacylglycerol (52:4)	IVW	7	-0.1187	0.040276	0.003207	-0.19764	-0.03976	0.888075	0.820665	0.961023	0.080681
GCST90277413	9ATSFc	Diabetic Retinopathy	Triacylglycerol (56:7)	IVW	5	-0.13072	0.045355	0.003951	-0.21961	-0.04182	0.877466	0.802829	0.959042	0.080681
GCST90277346	9ATSFc	Diabetic Retinopathy	Phosphatidylethanolamine (16:0_20:4)	IVW	5	-0.06963	0.024467	0.004426	-0.11759	-0.02168	0.932735	0.889062	0.978554	0.080681
GCST90277394	9ATSFc	Diabetic Retinopathy	Triacylglycerol (51:3)	IVW	6	-0.12531	0.044674	0.005032	-0.21287	-0.03775	0.882226	0.808263	0.962957	0.080681
GCST90277391	9ATSFc	Diabetic Retinopathy	Triacylglycerol (50:5)	IVW	3	-0.15715	0.056775	0.00564	-0.26843	-0.04588	0.854572	0.764576	0.95516	0.080681
GCST90277389	9ATSFc	Diabetic Retinopathy	Triacylglycerol (50:3)	IVW	3	-0.15086	0.05524	0.006313	-0.25914	-0.04259	0.859964	0.771719	0.958301	0.080681
GCST90277390	9ATSFc	Diabetic Retinopathy	Triacylglycerol (50:4)	IVW	3	-0.14756	0.054347	0.006623	-0.25408	-0.04104	0.862807	0.775627	0.959787	0.080681
GCST90277414	9ATSFc	Diabetic Retinopathy	Triacylglycerol (56:8)	IVW	5	-0.12723	0.049465	0.01011	-0.22418	-0.03027	0.880534	0.799172	0.970179	0.099488
GCST90277407	9ATSFc	Diabetic Retinopathy	Triacylglycerol (54:6)	IVW	3	-0.15911	0.062218	0.010547	-0.28106	-0.03717	0.852899	0.754982	0.963515	0.099488

GCST90277373	9ATSFc	Diabetic Retinopathy	Sphingomyelin (d36:2)	IVW	4	-0.11978	0.046944	0.010721	-0.21179	-0.02778	0.887111	0.809131	0.972607	0.099488
GCST90277261	9ATSFc	Diabetic Retinopathy	Diacylglycerol (18:1_18:1)	IVW	5	-0.1171	0.047962	0.014625	-0.21111	-0.02309	0.889495	0.809687	0.97717	0.120182
GCST90277397	9ATSFc	Diabetic Retinopathy	Triacylglycerol (52:3)	IVW	6	-0.09764	0.040475	0.015852	-0.17697	-0.01831	0.906977	0.837805	0.981859	0.120182
GCST90277399	9ATSFc	Diabetic Retinopathy	Triacylglycerol (52:5)	IVW	4	-0.11617	0.048289	0.016144	-0.21081	-0.02152	0.890326	0.809924	0.97871	0.120182
GCST90277396	9ATSFc	Diabetic Retinopathy	Triacylglycerol (52:2)	IVW	4	-0.11976	0.054773	0.028784	-0.22711	-0.0124	0.887135	0.796831	0.987674	0.173299
GCST90277317	9ATSFc	Diabetic Retinopathy	Phosphatidylcholine (18:2_20:4)	IVW	4	-0.12269	0.059336	0.038663	-0.23899	-0.00639	0.884536	0.787423	0.993626	0.173299
GCST90277247	9ATSFc	Diabetic Retinopathy	Sterol ester (27:1/18:3)	IVW	5	-0.09759	0.04722	0.038772	-0.19014	-0.00503	0.907025	0.826846	0.994979	0.173299
GCST90277360	9ATSFc	Diabetic Retinopathy	Phosphatidylinositol (16:0_20:4)	IVW	3	-0.13052	0.065525	0.046373	-0.25895	-0.0021	0.877635	0.771859	0.997907	0.173299
GCST90277349	9ATSFc	Diabetic Retinopathy	Phosphatidylethanolamine (18:1_18:1)	IVW	6	-0.07552	0.038314	0.048727	-0.15061	-0.00042	0.927265	0.860182	0.99958	0.173299
NWyUlf	bgsOMo	Diabetic Retinopathy	1-stearoyl-GPE (18:0)	IVW	7	-0.12726	0.038878	0.001063	-0.20346	-0.05106	0.880504	0.815901	0.950222	0.335891
qTq8oZ	bgsOMo	Diabetic Retinopathy	1-palmitoleoyl-GPC (16:1)	IVW	4	-0.19598	0.064934	0.002543	-0.32325	-0.06871	0.822027	0.723791	0.933596	0.406016
sz4Gjs	bgsOMo	Diabetic Retinopathy	5-hydroxylysine	IVW	4	0.10623	0.035353	0.002657	0.036939	0.175522	1.112078	1.03763	1.191868	0.406016
bovWvm	bgsOMo	Diabetic Retinopathy	2-stearoyl-GPE (18:0)	IVW	3	-0.20194	0.068532	0.003212	-0.33627	-0.06762	0.817142	0.714433	0.934617	0.406016
bAg65q	bgsOMo	Diabetic Retinopathy	X-24544	IVW	4	0.094578	0.033102	0.004275	0.029697	0.159458	1.099194	1.030142	1.172875	0.43959
JpLbOz	bgsOMo	Diabetic Retinopathy	X-26109	IVW	6	-0.06225	0.023021	0.006847	-0.10737	-0.01713	0.939644	0.898189	0.983013	0.43959
l48p4Z	bgsOMo	Diabetic Retinopathy	1-palmitoyl-2-docosahexaenoyl-GPE (16:0/22:6)	IVW	6	-0.07089	0.027592	0.010189	-0.12497	-0.01681	0.931562	0.882521	0.983328	0.43959
qvDQyh	bgsOMo	Diabetic Retinopathy	Oleoyl-linoleoyl-glycerol (18:1/18:2) [2]	IVW	7	-0.11049	0.04302	0.01022	-0.19481	-0.02617	0.895396	0.822992	0.974171	0.43959
O7rOPP	bgsOMo	Diabetic Retinopathy	1-stearoyl-2-docosahexaenoyl-GPE (18:0/22:6)	IVW	5	-0.08848	0.035956	0.013862	-0.15896	-0.01801	0.91532	0.853034	0.982153	0.43959
rv3dxu	bgsOMo	Diabetic Retinopathy	Metabolonic lactone sulfate	IVW	9	-0.05114	0.021622	0.018014	-0.09352	-0.00876	0.950142	0.910716	0.991274	0.450085
CCvMjz	bgsOMo	Diabetic Retinopathy	1-palmitoyl-GPE (16:0)	IVW	6	-0.10563	0.044739	0.018228	-0.19332	-0.01794	0.89976	0.824221	0.982222	0.450085
kiOFp8	bgsOMo	Diabetic Retinopathy	3-methylcytidine	IVW	4	0.064908	0.02756	0.018516	0.01089	0.118926	1.067061	1.010949	1.126287	0.450085
nWThM1	bgsOMo	Diabetic Retinopathy	1-stearoyl-gpc (18:0)	IVW	3	-0.16469	0.070509	0.019506	-0.30289	-0.02649	0.848157	0.738682	0.973857	0.456594
2gpdY5	bgsOMo	Diabetic Retinopathy	(2 or 3)-decenoate (10:1n7 or n8)	IVW	3	0.172741	0.075163	0.02155	0.025421	0.320061	1.188558	1.025747	1.377211	0.458414
MFZR2W	bgsOMo	Diabetic Retinopathy	Phosphate to oleoyl-linoleoyl-glycerol (18:1 to 18:2) [2]	IVW	6	0.106821	0.046667	0.022078	0.015354	0.198287	1.112735	1.015473	1.219313	0.458414

11AVUy	bgsOMo	Diabetic Retinopathy	(S)-a-amino-omega-caprolactam	IVW	3	-0.20266	0.088662	0.02227	-0.37643	-0.02888	0.816558	0.686304	0.971533	0.458414
8vdUya	bgsOMo	Diabetic Retinopathy	16a-hydroxy DHEA 3-sulfate	IVW	4	0.045599	0.021012	0.029992	0.004416	0.086782	1.046655	1.004426	1.090659	0.477316
XBLY8d	bgsOMo	Diabetic Retinopathy	N-stearoyl-sphingosine (d18:1 to 18:0) to N-palmitoyl-sphinganine (d18:0 to 16:0)	IVW	5	-0.11152	0.05141	0.030071	-0.21228	-0.01075	0.894476	0.808737	0.989305	0.477316
JeOlr0	bgsOMo	Diabetic Retinopathy	5alpha-androstan-3beta,17beta-diol disulfate	IVW	5	-0.0661	0.030967	0.032795	-0.1268	-0.00541	0.936036	0.880913	0.994608	0.477316
uGLKq3	bgsOMo	Diabetic Retinopathy	Sphingomyelin (d18:1/20:1, d18:2/20:0)	IVW	4	-0.09676	0.045423	0.03315	-0.18579	-0.00773	0.907771	0.830446	0.992296	0.477316
WlmWdG	bgsOMo	Diabetic Retinopathy	1-(1-enyl-palmitoyl)-2-palmitoyl-GPC (P-16:0/16:0)	IVW	7	-0.06985	0.03285	0.033487	-0.13423	-0.00546	0.932538	0.874388	0.994555	0.477316
VCfnXS	bgsOMo	Diabetic Retinopathy	Glyco-beta-muricholate	IVW	3	0.052362	0.024733	0.03425	0.003886	0.100838	1.053757	1.003893	1.106097	0.477316
BSdLQQ	bgsOMo	Diabetic Retinopathy	1-palmitoyl-2-oleoyl-GPI (16:0/18:1)	IVW	4	0.104961	0.04983	0.035172	0.007294	0.202628	1.110667	1.00732	1.224617	0.477316
iyY6VS	bgsOMo	Diabetic Retinopathy	Sphingomyelin (d18:1/18:1, d18:2/18:0)	IVW	4	-0.11215	0.053304	0.035387	-0.21662	-0.00767	0.893914	0.805235	0.992359	0.477316
2Iat3i	bgsOMo	Diabetic Retinopathy	Homocitrulline	IVW	4	-0.08408	0.040032	0.035691	-0.16255	-0.00562	0.919353	0.849975	0.994394	0.477316
7nPRpG	bgsOMo	Diabetic Retinopathy	5alpha-androstan-3alpha,17alpha-diol monosulfate	IVW	6	-0.04861	0.023256	0.0366	-0.09419	-0.00303	0.952552	0.910107	0.996977	0.477316
vblnRy	bgsOMo	Diabetic Retinopathy	Epiandrosterone sulfate	IVW	5	-0.03537	0.016936	0.036736	-0.06857	-0.00218	0.965244	0.933728	0.997823	0.477316
3qxNmb	bgsOMo	Diabetic Retinopathy	Indoleacetylglutamine	IVW	4	-0.07153	0.034295	0.037007	-0.13874	-0.00431	0.930971	0.87045	0.995699	0.477316
6XVwdY	bgsOMo	Diabetic Retinopathy	1-palmitoyl-2-linoleoyl-GPI (16:0/18:2)	IVW	5	0.089244	0.043474	0.040088	0.004036	0.174452	1.093348	1.004044	1.190594	0.478121
mOnDnC	bgsOMo	Diabetic Retinopathy	Ceramide (d18:1/24:1)	IVW	6	0.076792	0.037543	0.040813	0.003207	0.150377	1.079818	1.003212	1.162272	0.478121
mTmMwi	bgsOMo	Diabetic Retinopathy	Dodecadienoate (12:2)	IVW	5	0.106767	0.053026	0.044064	0.002836	0.210697	1.112674	1.00284	1.234538	0.488564
ebi-a-GCST90001531	WlsRdR	Diabetic Neuropathy	CD33dim HLA DR- AC Myeloid cell	IVW	3	-0.04941	0.020557	0.016234	-0.0897	-0.00912	0.951791	0.914204	0.990922	0.591424
ebi-a-GCST90001532	WlsRdR	Diabetic Neuropathy	Basophil AC Myeloid cell	IVW	3	-0.04855	0.020568	0.018247	-0.08887	-0.00824	0.952607	0.914968	0.991795	0.591424
kSb5G1	rYBKu8	Diabetic Neuropathy	Species UBA1375 sp002305795	IVW	8	0.670921	0.211216	0.001491	0.256938	1.084904	1.956038	1.292965	2.959154	0.705138
KYXv9z	rYBKu8	Diabetic Neuropathy	Species Flavonifractor sp002159265	IVW	9	-0.65395	0.225655	0.003755	-1.09624	-0.21167	0.519987	0.334127	0.809233	0.888132
1pcbtU	rYBKu8	Diabetic Neuropathy	Phylum Flavobacteriales	IVW	14	-0.84856	0.31158	0.006461	-1.45926	-0.23786	0.428031	0.232409	0.788312	0.965903
teZAMJ	rYBKu8	Diabetic Neuropathy	Species Blautia sp000436935	IVW	14	0.236262	0.090138	0.008764	0.059592	0.412933	1.266507	1.061403	1.511244	0.965903
ONqPAv	rYBKu8	Diabetic Neuropathy	Species UBA1448	IVW	4	0.921921	0.369174	0.012516	0.198339	1.645503	2.514114	1.219375	5.183614	0.965903

SisCXl	rYBKu8	Diabetic Neuropathy	Species CAG-273 sp003534295	IVW	7	0.246272	0.099466	0.013288	0.051319	0.441225	1.279248	1.052659	1.55461	0.965903
yk68Jl	rYBKu8	Diabetic Neuropathy	Species Bacteroides A plebeius	IVW	9	0.212565	0.086769	0.014295	0.042497	0.382633	1.236847	1.043413	1.466139	0.965903
1jYqJ0	rYBKu8	Diabetic Neuropathy	Species GCA-900066135 sp900066135	IVW	7	-0.6476	0.280102	0.020777	-1.1966	-0.0986	0.523299	0.302219	0.906103	0.996485
1BHknX	rYBKu8	Diabetic Neuropathy	Genus Enterococcus A	IVW	12	0.552828	0.262581	0.03526	0.038169	1.067487	1.738161	1.038907	2.908061	0.996485
MZ3oef	rYBKu8	Diabetic Neuropathy	Genus Succinivibrio	IVW	21	-0.2173	0.104976	0.038457	-0.42305	-0.01154	0.804692	0.655046	0.988524	0.996485
aSPBdS	rYBKu8	Diabetic Neuropathy	Class Clostridia	IVW	6	0.895035	0.437482	0.040768	0.03757	1.7525	2.447421	1.038284	5.769008	0.996485
LKakmo	rYBKu8	Diabetic Neuropathy	Unclassified UBA8517	IVW	9	-0.64717	0.330072	0.049914	-1.29411	-0.00023	0.523524	0.274141	0.999768	0.996485
GCST90274842	Ef6GPW	Diabetic Neuropathy	TNFSF14	IVW	4	-0.20384	0.086222	0.018073	-0.37283	-0.03484	0.815594	0.688779	0.965757	0.574746
GCST90274820	Ef6GPW	Diabetic Neuropathy	LIFR	IVW	3	0.197037	0.083936	0.018901	0.032523	0.36155	1.217789	1.033057	1.435553	0.574746
GCST90274765	Ef6GPW	Diabetic Neuropathy	CCL19	IVW	3	-0.14517	0.069411	0.036492	-0.28121	-0.00912	0.864879	0.754869	0.990921	0.574746
bBTd9n	PO7ZNY	Diabetic Neuropathy	Phosphate to serine	IVW	3	0.3444	0.100288	0.000594	0.147836	0.540964	1.411143	1.159322	1.717662	0.147179
67ZWOs	PO7ZNY	Diabetic Neuropathy	Serine	IVW	3	-0.29925	0.093971	0.00145	-0.48343	-0.11507	0.741373	0.616662	0.891306	0.229103
8T7iMz	PO7ZNY	Diabetic Neuropathy	3-hydroxyhexanoylcarnitine (1)	IVW	3	-0.35505	0.121412	0.003452	-0.59302	-0.11709	0.701136	0.552656	0.889509	0.436279
xdxcHi	PO7ZNY	Diabetic Neuropathy	X-24556	IVW	3	-0.24091	0.08608	0.005132	-0.40962	-0.07219	0.785915	0.6639	0.930355	0.540579
uVrSDL	PO7ZNY	Diabetic Neuropathy	X-24328	IVW	7	-0.25237	0.101098	0.012551	-0.45052	-0.05421	0.77696	0.637297	0.947229	0.710531
s4ctla	PO7ZNY	Diabetic Neuropathy	3-hydroxybutyroylglycine	IVW	3	-0.1598	0.067847	0.018512	-0.29278	-0.02681	0.852318	0.746189	0.973542	0.710531
JcPwzk	PO7ZNY	Diabetic Neuropathy	N-acetylputrescine to (N(1) + N(8))-acetylspermidine	IVW	5	0.105885	0.045198	0.019145	0.017297	0.194474	1.111694	1.017447	1.214672	0.710531
Ub4VMX	PO7ZNY	Diabetic Neuropathy	2-butenoylglycine	IVW	3	-0.18064	0.077724	0.020117	-0.33298	-0.0283	0.834733	0.716783	0.972092	0.710531
aa5Vyh	PO7ZNY	Diabetic Neuropathy	1-oleoyl-2-docosahexaenoyl-GPC (18:1/22:6)	IVW	5	-0.30944	0.133369	0.020329	-0.57085	-0.04804	0.733854	0.565046	0.953094	0.710531
6m7Q6j	PO7ZNY	Diabetic Neuropathy	X-26054	IVW	4	0.120839	0.053634	0.024256	0.015717	0.225961	1.128444	1.015842	1.253527	0.712564
xIK9X5	PO7ZNY	Diabetic Neuropathy	Indolelactate	IVW	3	-0.24999	0.113157	0.027161	-0.47178	-0.0282	0.77881	0.623893	0.972195	0.712564
GKHDzV	PO7ZNY	Diabetic Neuropathy	Paraxanthine to 5-acetylamino-6-formylamino-3-methyluracil	IVW	4	-0.07472	0.034901	0.032281	-0.14313	-0.00631	0.928002	0.866643	0.993706	0.712564
TI3QmT	PO7ZNY	Diabetic Neuropathy	Argininate	IVW	5	0.19039	0.089356	0.033114	0.015252	0.365527	1.209721	1.015369	1.441274	0.712564
6GevTF	PO7ZNY	Diabetic Neuropathy	Orotate	IVW	4	0.088407	0.042549	0.037729	0.005012	0.171803	1.092433	1.005024	1.187444	0.769178

8Fsdwg	PO7ZNY	Diabetic Neuropathy	X-25433	IVW	3	-0.13364	0.065982	0.042825	-0.26296	-0.00432	0.874905	0.768769	0.995693	0.820164
h3VOoN	PO7ZNY	Diabetic Neuropathy	Carnitine	IVW	4	-0.12595	0.062799	0.044905	-0.24903	-0.00286	0.881662	0.779554	0.997144	0.834712
ukb-b-16202	DqkEkJ	Diabetic Neuropathy	Carotene	IVW	15	0.489069	0.22305	0.028333	0.051891	0.926247	1.630798	1.053261	2.525015	0.392796

Supplementary Table 11: Genes significantly associated with immune cells and DMiVD.

**id**	**chr**	**start**	**end**	**strand**	**gene_ids**	**gene_names**	**3_prime_utr_variant**	**5_prime_utr_variant**	**nmd_transcript_variant**	**intron_variant**	**missense_variant**	**non_coding_transcript_exon_variant**	**non_coding_transcript_variant**	**splice_polypyrimidine_tract_variant**	**splice_region_variant**	**synonymous_variant**
**rs114590598**	1	1.86E+08	1.86E+08	+	ENSG00000143341	HMCN1	0	0	0	2	0	0	0	0	0	0
**rs339786222**	19	51233128	51233128	+	ENSG00000105383	CD33	0	0	0	6	0	0	2	0	0	0
**rs559714 73**	1	1.17E+08	1.17E+08	+	ENSG00000116815	CD58	1	0	1	2	0	0	0	0	0	0
**rs114763811**	2	1.9E+08	1.9E+08	+	ENSG00000151690,ENSG00000272979	MFSD6	0	0	0	4	0	1	1	0	0	0
**rs79190516**	2	1.5E+08	1.5E+08	+	ENSG00000230645	LINC01818	0	0	0	4	0	0	4	0	0	0
**rs114135956**	2	8790400	8790400	+	ENSG00000134313	KIDINS220	0	0	4	27	0	0	8	0	0	0
**rs150146567**	2	12644082	12644082	+	ENSG00000224184	MIR3681HG	0	0	0	2	0	0	2	0	0	0
**rs149994650**	3	18509372	18509372	+	ENSG00000228956	SATB1-AS1	0	0	0	120	0	0	120	0	0	0

**rs62409210**	4	25411151	25411151	+	ENSG00000053900	ANAPC4	0	0	0	4	0	0	2	0	0	0
**rs76506268**	4	1.87E+08	1.87E+08	+	ENSG00000250971	ENSG00000250971	0	0	0	5	0	0	5	0	0	0
**rs12656604**	5	73383229	73383229	+	ENSG00000293193	ENSG00000293193	0	0	0	2	0	0	2	0	0	0
**rs115546994**	5	1.6E+08	1.6E+08	+	ENSG00000249738	ENSG00000249738	0	0	0	1	0	0	1	0	0	0
**rs76343453**	5	82058101	82058101	+	ENSG00000152348	ATG10	0	0	1	6	0	0	0	0	0	0
**rs130071**	6	31148433	31148433	+	ENSG00000204536	CCHCR1	2	0	2	0	0	1	0	0	0	4
**rs2977034**	8	87491486	87491486	+	ENSG00000176571	CNBD1	0	0	0	2	0	0	0	0	0	0
**rs62488802**	8	7305173	7305173	+	ENSG00000215374	FAM66B	0	0	0	6	0	0	6	0	0	0
**rs143930177**	10	68783949	68783949	+	ENSG00000060339	CCAR1	0	0	2	14	0	0	2	0	0	0
**rs11233425**	11	83021647	83021647	+	ENSG00000137502,ENSG00000254698	RAB30	0	0	0	13	0	0	1	0	0	0
**rs61908026**	12	2699000	2699000	+	ENSG00000256150	ITFG2-AS1	0	0	0	1	0	0	1	0	0	0
**rs189825578**	12	31602630	31602630	+	ENSG00000255867	DENND5B-AS1	0	0	0	4	0	0	4	0	0	0

**rs117975109**	12	52014358	52014358	+	ENSG00000161835	TA-MA-LIN	0	0	0	5	0	1	2	0	0	0
**rs538264691**	12	96646243	96646243	+	ENSG00000188596	CFAP54	1	0	0	2	0	0	0	0	0	0
**rs117551202**	14	45056966	45056966	+	ENSG00000198718	TO-GARAM1	0	0	1	3	0	0	0	0	0	0
**rs742867**	14	67751007	67751007	+	ENSG00000072121	ZFYVE26	1	0	1	7	0	0	2	0	0	0
**rs62021713**	15	50073192	50073192	+	ENSG00000104043	ATP8B4	0	0	3	8	0	0	1	0	0	0
**rs149716976**	16	75028727	75028727	+	ENSG00000186187	ZNRF1	0	0	0	6	0	0	1	0	0	0
**rs34375694**	16	19182041	19182041	+	ENSG00000103528,ENSG00000286790	SYT17	0	0	0	8	0	0	2	0	0	0
**rs192302673**	16	85651534	85651534	+	ENSG00000131149	GSE1	0	0	0	10	0	0	0	0	0	0
**rs75420474**	18	42248753	42248753	+	ENSG00000267586	LINC00907	0	0	0	10	0	0	10	0	0	0
**rs117428784**	19	42918805	42918805	+	ENSG00000170848	PSG6	0	2	0	0	0	0	0	0	0	0
**rs12145596**	1	1.67E+08	1.67E+08	+	ENSG00000198821,ENSG00000233411,ENSG00000273160,ENSG0	CD247	0	0	2	23	0	1	8	0	0	0

					0000287218											
**rs145583539**	1	66024420	66024420	+	ENSG00000184588	PDE4B	0	0	0	6	0	0	3	0	0	0
**rs41299575**	1	1.13E+08	1.13E+08	+	ENSG00000198799	LRIG2	0	0	0	3	0	0	2	0	0	0
**rs144942838**	1	1.11E+08	1.11E+08	+	ENSG00000134216	CHIA	0	0	0	3	0	0	0	0	0	0
**rs147591823**	1	47129431	47129431	+	ENSG00000225506	CYP4A22-AS1	0	0	0	1	0	0	1	0	0	0
**rs567145695**	1	2.46E+08	2.46E+08	+	ENSG00000185420	SMYD3	0	0	0	10	0	0	4	0	0	0
**rs114021246**	1	46433117	46433117	+	ENSG00000232022	FAAHP1	0	0	0	2	0	0	2	0	0	0
**rs115765563**	1	6058633	6058633	+	ENSG00000069424	KCNAB2	0	0	2	37	0	0	7	0	0	0
**rs75255396**	1	35427338	35427338	+	ENSG00000142687	KI-AA0319L	0	0	1	1	0	0	0	0	0	0
**rs140963286**	1	24313977	24313977	+	ENSG00000158055,ENSG00000232298	GRHL3	0	0	0	2	0	0	1	0	0	0
**rs115175419**	1	99994099	99994099	+	ENSG00000117620,ENSG00000283761	SLC35A3	0	0	14	32	0	0	2	0	0	0
**rs114230155**	1	1.02E+08	1.02E+08	+	ENSG00000233359	ENSG00000233359	0	0	0	2	0	0	2	0	0	0

**rs149050426**	1	1.61E+08	1.61E+08	+	ENSG00000117091	CD48	0	0	0	3	0	0	0	0	0	0
**rs76636736**	1	99077250	99077250	+	ENSG00000117598,ENSG00000232825	PLPPR5	0	0	0	6	0	0	5	0	0	0
**rs595254**	1	12168753	12168753	+	ENSG00000028137	TNFRSF1B	0	0	0	9	0	0	3	0	0	0
**rs78656354**	1	1.67E+08	1.67E+08	+	ENSG00000143195	ILDR2	0	0	0	7	0	0	0	0	0	0
**rs114507999**	1	21565773	21565773	+	ENSG00000162551	ALPL	0	0	0	4	0	0	0	0	0	0
**rs34772471**	1	1.12E+08	1.12E+08	+	ENSG00000197852	INKA2	0	0	0	2	0	1	1	0	0	1
**rs373989598**	1	2.06E+08	2.06E+08	+	ENSG00000266028	SRGAP2	0	0	0	8	0	0	0	0	0	0
**rs12750174**	1	1.85E+08	1.85E+08	+	ENSG00000135842	NIBAN1	0	0	0	1	1	1	0	0	0	0
**rs148484526**	1	2.04E+08	2.04E+08	+	ENSG00000133056	PIK3C2B	0	0	0	2	0	0	0	0	0	0
**rs141644068**	1	2.21E+08	2.21E+08	+	ENSG00000116141	MARK1	0	0	6	39	0	0	15	0	0	0
**rs143940797**	1	52179211	52179211	+	ENSG00000157077	ZFYVE9	0	0	0	4	0	0	1	0	0	0
**rs77538429**	1	1.1E+08	1.1E+08	+	ENSG00000197106	SLC6A17	1	0	0	0	0	0	0	0	0	0

**rs113698814**	1	2.08E+08	2.08E+08	+	ENSG00000203710	CR1	0	0	1	13	0	0	5	0	0	0
**rs138152896**	2	40319640	40319640	+	ENSG00000183023	SLC8A1	0	0	1	15	0	0	0	0	0	0
**rs3821186**	2	1.72E+08	1.72E+08	+	ENSG00000144355,ENSG00000288958	DLX1	4	0	0	2	0	2	2	0	0	0
**rs148947489**	2	10186292	10186292	+	ENSG00000171848	RRM2	0	0	2	4	0	0	2	0	0	0
**rs79027575**	2	1.46E+08	1.46E+08	+	ENSG00000286225	ENSG00000286225	0	0	0	1	0	0	1	0	0	0
**rs112785730**	2	1.27E+08	1.27E+08	+	ENSG00000136717	BIN1	0	0	0	12	0	0	0	0	0	0
**rs183895386**	2	22354071	22354071	+	ENSG00000228999	LINC01830	0	0	0	1	0	0	1	0	0	0
**rs190665193**	2	1.06E+08	1.06E+08	+	ENSG00000071051	NCK2	0	0	0	4	0	0	0	0	0	0
**rs35948774**	2	1.18E+08	1.18E+08	+	ENSG00000290590	ENSG00000290590	0	0	0	2	0	0	2	0	0	0
**rs377446032**	2	97097248	97097248	+	ENSG00000279791	ENSG00000279791	0	0	0	0	0	1	0	0	0	0
**rs7597139**	2	1.07E+08	1.07E+08	+	ENSG00000286218	ENSG00000286218	0	0	0	2	0	0	2	0	0	0
**rs1508064**	2	72752043	72752043	+	ENSG00000144036	EXOC6B	0	0	2	8	0	0	0	0	0	0

**rs72931475**	2	1.58E+08	1.58E+08	+	ENSG00000144283	PKP4	0	0	5	8	0	0	1	0	0	0
**rs188021268**	2	67562747	67562747	+	ENSG00000224173	LINC02831	0	0	0	2	0	0	2	0	0	0
**rs567695407**	2	1.07E+08	1.07E+08	+	ENSG00000229457	LINC01789	0	0	0	4	0	0	4	0	0	0
**rs75561723**	2	1.99E+08	1.99E+08	+	ENSG00000225421	ENSG00000225421	0	0	0	2	0	0	2	0	0	0
**rs13010105**	2	41880840	41880840	+	ENSG00000214691	LINC01913	0	0	0	8	0	1	8	0	0	0
**rs12616335**	2	1.59E+08	1.59E+08	+	ENSG00000115183	TANC1	0	0	0	1	0	0	0	0	0	0
**rs76568972**	2	1588500	1588500	+	ENSG00000228613	ENSG00000228613	0	0	0	1	0	0	1	0	0	0
**rs138567976**	2	8012691	8012691	+	ENSG00000235665,ENSG00000236790	LINC00298,LINC00299	0	0	0	7	0	0	7	0	0	0
**rs149131245**	2	2.34E+08	2.34E+08	+	ENSG00000185038	MROH2A	0	0	0	4	0	0	0	0	0	0
**rs4853078**	2	74920954	74920954	+	ENSG00000204792,ENSG00000236209	LINC01291	0	0	0	2	0	0	2	0	0	0
**rs77678807**	2	2.08E+08	2.08E+08	+	ENSG00000178385	PLEKHM3	0	0	0	2	0	0	0	0	0	0
**rs729964**	2	2.1E+08	2.1E+08	+	ENSG0000016853	MYL1	0	0	0	1	0	0	0	0	0	0

**38**					0											
**rs115945729**	2	1.99E+08	1.99E+08	+	ENSG00000231557	ENSG00000231557	0	0	0	1	0	0	1	0	0	0
**rs149876155**	2	23944807	23944807	+	ENSG00000173960	UBXN2A	0	0	0	3	0	0	1	0	0	0
**rs771566 03**	2	1.04E+08	1.04E+08	+	ENSG00000256637	LINC01965	0	0	0	3	0	0	3	0	0	0
**rs62272737**	3	1.52E+08	1.52E+08	+	ENSG00000152601	MBNL1	0	0	0	36	0	0	0	0	0	0
**rs16864501**	3	1.53E+08	1.53E+08	+	ENSG00000287706	ENSG00000287706	0	0	0	6	0	0	6	0	0	0
**rs75660425**	3	1.3E+08	1.3E+08	+	ENSG00000163913	IFT122	0	0	29	87	0	1	20	0	0	0
**rs146756366**	3	76610917	76610917	+	ENSG00000185008	RO-BO2	0	0	0	6	0	0	0	0	0	0
**rs115660429**	3	1.96E+08	1.96E+08	+	ENSG00000145113	MUC4	0	0	16	24	0	0	0	0	0	0
**rs116719160**	3	1.3E+08	1.3E+08	+	ENSG00000172752	COL6A5	0	0	1	3	0	0	0	0	0	0
**rs79418578**	3	1.42E+08	1.42E+08	+	ENSG00000175066	GK5	0	0	3	5	0	0	1	0	0	0
**rs78741146**	3	1.83E+08	1.83E+08	+	ENSG00000243378	PLA2G10P1	0	0	0	0	0	2	0	0	0	0
**rs148658406**	3	1.54E+08	1.54E+08	+	ENSG00000238755	LINC02006	0	0	0	1	0	0	1	0	0	0
**rs557874**	3	1.86	1.86	+	ENSG00000	ETV5	4	0	0	0	0	2	0	0	0	0

**44**		E+08	E+08		244405											
**rs78193714**	3	1.86E+08	1.86E+08	+	ENSG00000224406,ENSG00000226859	LINC02052	0	0	0	6	0	0	6	0	0	0
**rs62247421**	3	64404791	64404791	+	ENSG00000163637	PRICKLE2	0	0	0	6	0	0	2	0	0	0
**rs115212782**	3	74478625	74478625	+	ENSG00000113805	CNTN3	0	0	0	1	0	0	0	0	0	0
**rs111902471**	3	14417591	14417591	+	ENSG00000131389	SLC6A6	0	0	4	9	0	0	2	0	0	0
**rs115040341**	3	64557936	64557936	+	ENSG00000163638	ADAMTS9	0	0	0	4	0	0	1	0	0	0
**rs178786699**	3	3091070	3091070	+	ENSG00000091181	IL5RA	0	0	0	6	0	0	0	0	0	0
**rs111745963**	3	67809792	67809792	+	ENSG00000241316	SU-CLG2-DT	0	0	0	3	0	0	3	0	0	0
**rs115879183**	3	1.95E+08	1.95E+08	+	ENSG00000145014	TMEM44	0	0	2	8	0	0	1	0	0	0
**rs78313301**	3	11033498	11033498	+	ENSG00000157103	SLC6A1	0	0	1	31	0	1	2	0	0	0
**rs184977394**	3	81750550	81750550	+	ENSG00000114480	GBE1	0	0	0	1	0	0	0	0	0	0
**rs13104493**	4	32689249	32689249	+	ENSG00000286784	ENSG00000286784	0	0	0	2	0	0	2	0	0	0
**rs116399**	4	1.76E+08	1.76E+08	+	ENSG0000015062	GPM6A	0	0	0	11	0	0	0	0	0	0

**750**					5											
**rs114619811**	4	71009967	71009967	+	ENSG00000156136,ENSG00000173542	DCK, MOB1B	0	0	1	5	0	0	1	0	0	0
**rs138943622**	4	1.13E+08	1.13E+08	+	ENSG00000145362	ANK2	0	0	2	89	0	5	9	0	0	0
**rs114559319**	4	84292649	84292649	+	ENSG00000250546	LINC02994	0	0	0	6	0	0	6	0	0	0
**rs76859704**	4	1.48E+08	1.48E+08	+	ENSG00000151623	NR3C2	0	0	1	6	0	0	0	0	0	0
**rs114421150**	4	16985307	16985307	+	ENSG00000249998	ENSG00000249998	0	0	0	1	0	0	1	0	0	0
**rs138954494**	4	32183072	32183072	+	ENSG00000251129	LINC02506	0	0	0	7	0	0	7	0	0	0
**rs76679968**	4	1.29E+08	1.29E+08	+	ENSG00000077684	JADE1	0	0	1	13	0	0	0	0	0	0
**rs12499962**	4	98108306	98108306	+	ENSG00000163116	STPG2	0	0	0	1	0	0	0	0	0	0
**rs149844272**	4	86223609	86223609	+	ENSG00000109339	MAPK10	0	0	66	324	0	0	34	0	0	0
**rs77434296**	4	22138688	22138688	+	ENSG00000250039	ENSG00000250039	0	0	0	1	0	0	1	0	0	0
**rs28622225**	4	1.65E+08	1.65E+08	+	ENSG00000052802	MSMO1	0	0	0	4	0	0	0	0	0	0
**rs116498781**	4	1.82E+08	1.82E+08	+	ENSG0000021833	TENM3	0	0	0	1	0	0	0	0	0	0

					6											
**rs148590919**	4	1.87 E+08	1.87 E+08	+	ENSG00000083857	FAT1	0	0	0	1	0	0	0	0	0	0
**rs72723889**	4	1.46 E+08	1.46 E+08	+	ENSG00000151612	ZNF827	0	0	1	14	0	0	5	0	0	0
**rs77146551**	4	1.07 E+08	1.07 E+08	+	ENSG00000155011	DKK2	0	0	0	2	0	0	0	0	0	0
**rs3822406**	5	1.81 E+08	1.81 E+08	+	ENSG00000037280	FLT4	0	0	0	12	0	0	4	0	0	0
**rs142922198**	5	1.46 E+08	1.46 E+08	+	ENSG00000250025,ENSG00000275740	ENSG00000250025	0	0	0	4	0	0	2	0	0	0
**rs189369117**	5	1.44 E+08	1.44 E+08	+	ENSG00000251205	ENSG00000251205	0	0	0	0	0	1	0	0	0	0
**rs176425 70**	5	1.49 E+08	1.49 E+08	+	ENSG00000157510	AFAP1L1	0	0	0	6	0	0	2	0	0	0
**rs192556004**	5	1.36 E+08	1.36 E+08	+	ENSG00000069018,ENSG00000250947	TRPC7	0	0	2	6	0	1	0	0	0	0
**rs189412340**	5	76269118	76269118	+	ENSG00000122012	SV2C	0	0	0	2	0	0	0	0	0	0
**rs114871166**	5	24165328	24165328	+	ENSG00000248874,ENSG00000286134	LINC02899	0	0	0	2	0	0	2	0	0	0
**rs114425**	5	1.77 E+08	1.77 E+08	+	ENSG0000019692	PDLIM7	0	0	2	5	0	0	1	0	0	0

**563**					3											
**rs62337106**	5	1957289	1957289	+	ENSG00000248994	ENSG00000248994	0	0	0	7	0	0	7	0	0	0
**rs112973846**	5	1.29E+08	1.29E+08	+	ENSG00000113396	SLC27A6	0	0	0	3	0	0	0	0	0	0
**rs150287087**	6	1.25E+08	1.25E+08	+	ENSG00000188580,ENSG00000236548	NKAIN2	0	0	0	16	0	0	14	0	0	0
**rs149801155**	6	1.62E+08	1.62E+08	+	ENSG00000185345	PRKN	0	0	8	22	0	0	6	0	0	0
**rs138327786**	6	1730748	1730748	+	ENSG00000112699	GMDS	0	0	0	8	0	0	4	0	0	0
**rs117822024**	6	85636891	85636891	+	ENSG00000135316,ENSG00000271793	SYNCRIP	0	0	10	32	0	0	3	0	0	0
**rs9479093**	6	1.52E+08	1.52E+08	+	ENSG00000091831	ESR1	0	0	0	3	0	0	3	0	0	0
**rs116431337**	6	65706679	65706679	+	ENSG00000188107	EYS	0	0	0	4	0	0	1	0	0	0
**rs6916896**	6	5734653	5734653	+	ENSG00000145982	FARS2	0	0	1	3	0	0	0	0	0	0
**rs180745907**	6	20965554	20965554	+	ENSG00000145996	CDKAL1	0	0	0	2	0	0	0	0	0	0
**rs145336406**	6	78035458	78035458	+	ENSG00000230309	ENSG00000230309	0	0	0	1	0	0	1	0	0	0

**rs782752 21**	6	9019 0825	9019 0825	+	ENSG 00000 11218 2	BAC H2	0	0	0	11	0	0	6	0	0	0
**rs249615 0**	6	1337 3900	1337 3900	+	ENSG 00000 14599 0	GFO D1	0	0	0	9	0	0	0	0	0	0
**rs117185 099**	6	1.19 E+08	1.19 E+08	+	ENSG 00000 11188 5	MAN 1A1	0	0	0	1	0	0	0	0	0	0
**rs754243 81**	6	1180 4891	1180 4891	+	ENSG 00000 11186 3	AD-TRP	0	0	0	1	0	0	0	0	0	0
**rs111867 376**	6	1.6E +08	1.6E +08	+	ENSG 00000 28653 3	ENSG 00000 28653 3	0	0	0	1	0	0	1	0	0	0
**rs149208 831**	6	1001 7443	1001 7443	+	ENSG 00000 29338 5	ENSG 00000 29338 5	0	0	0	3	0	0	3	0	0	0
**rs145604 620**	6	1149 1017	1149 1017	+	ENSG 00000 24275 3	ENSG 00000 24275 3	0	0	0	2	0	0	2	0	0	0
**rs755106 26**	6	6369 1241	6369 1241	+	ENSG 00000 11848 2	PHF3	0	0	1	7	0	0	0	0	0	0
**rs149828 559**	6	8418 8405	8418 8405	+	ENSG 00000 13531 5	CEP1 62	0	0	0	5	0	0	2	0	0	0
**rs189131 343**	6	1.67 E+08	1.67 E+08	+	ENSG 00000 07124 2	RPS6 KA2	0	0	0	9	0	0	0	0	0	0
**rs947101 6**	6	3922 8616	3922 8616	+	ENSG 00000 16462 6	KCN K5	0	0	0	1	0	0	0	0	0	0
**rs800188 78**	7	4694 5723	4694 5723	+	ENSG 00000 23207 2	ENSG 00000 23207 2	0	0	0	1	0	0	1	0	0	0
**rs137997 289**	7	1.16 E+08	1.16 E+08	+	ENSG 00000 10596	TFEC	0	0	0	1	0	0	0	0	0	0

					7											
**rs77465871**	7	47464441	47464441	+	ENSG00000136205	TNS3	0	0	1	7	0	0	0	0	0	0
**rs80305889**	7	34641989	34641989	+	ENSG00000197085	NPSR1-AS1	0	0	0	8	0	0	8	0	0	0
**rs554574739**	7	1.02E+08	1.02E+08	+	ENSG00000257923	CUX1	0	0	0	22	0	0	5	0	0	0
**rs117469911**	7	80162028	80162028	+	ENSG00000127955	GNAI1	0	0	5	23	0	0	1	0	0	0
**rs78470553**	7	1.59E+08	1.59E+08	+	ENSG00000155093	PTPRN2	0	0	0	4	0	0	0	0	0	0
**rs186251573**	7	13518955	13518955	+	ENSG00000229618	ENSG00000229618	0	0	0	14	0	0	14	0	0	0
**rs138449036**	7	28985668	28985668	+	ENSG00000272568	CPVL-AS2	0	0	0	2	0	0	2	0	0	0
**rs79647440**	7	1.43E+08	1.43E+08	+	ENSG00000286831	ENSG00000286831	0	0	0	1	0	0	1	0	0	0
**rs35706199**	7	1.32E+08	1.32E+08	+	ENSG00000128567	PODXL	0	0	1	4	0	0	1	0	0	0
**rs150400236**	7	1.37E+08	1.37E+08	+	ENSG00000234352	ENSG00000234352	0	0	0	2	0	0	2	0	0	0
**rs117079032**	7	70045144	70045144	+	ENSG00000158321	AUTS2	0	0	1	9	0	0	1	0	0	0
**rs185754925**	7	63456632	63456632	+	ENSG00000197990	ZNF734P	0	0	0	0	0	1	0	0	0	0
**rs111399**	7	1.47	1.47	+	ENSG00000	CNT	0	0	0	5	0	0	4	0	0	0

**817**		E+08	E+08		174469	NAP2									
**rs73206236**	7	1.14 E+08	1.14 E+08	+	ENSG00000154415	PPP1R3A	0	0	0	1	0	0	0	0	0
**rs116921833**	7	1.54 E+08	1.54 E+08	+	ENSG00000130226	DPP6	0	0	0	8	0	0	0	0	0
**rs8178689**	8	42202554	42202554	+	ENSG00000104368	PLAT	0	0	1	11	0	0	1	0	0
**rs34039708**	8	12643323	12643323	+	ENSG00000283674	ENSG00000283674	0	0	0	6	0	0	6	0	0
**rs181660070**	8	1.16 E+08	1.16 E+08	+	ENSG00000249917	LINC00536	0	0	0	1	0	0	1	0	0
**rs117741105**	8	1.11 E+08	1.11 E+08	+	ENSG00000253103	LINC01609	0	0	0	10	0	0	10	0	0
**rs117626443**	8	68951927	68951927	+	ENSG00000253658	LINC01592	0	0	0	14	0	0	14	0	0
**rs75361870**	8	9345099	9345099	+	ENSG00000248538	PPP1R3B-DT	0	0	0	62	0	0	62	0	0
**rs147959386**	8	1.23 E+08	1.23 E+08	+	ENSG00000147689	FAM83A	0	0	0	6	0	0	1	0	0
**rs117123362**	8	1.29 E+08	1.29 E+08	+	ENSG00000229140	CCDC26	0	0	0	4	0	0	4	0	0
**rs117983267**	8	1.23 E+08	1.23 E+08	+	ENSG00000287657	ENSG00000287657	0	0	0	1	0	0	1	0	0
**rs144258173**	8	3001878	3001878	+	ENSG00000183117	CSMD1	0	0	0	7	0	0	1	0	0

**rs118076041**	8	1.43E+08	1.43E+08	+	ENSG00000141664	ZC3H3	0	0	0	2	0	0	0	0	0	0
**rs117448261**	8	81487785	81487785	+	ENSG00000253859	ENSG00000253859	0	0	0	1	0	0	1	0	0	0
**rs374964617**	9	504491	504491	+	ENSG00000107104	KANK1	0	0	0	3	0	0	2	0	0	0
**rs77733172**	9	1.37E+08	1.37E+08	+	ENSG00000187609	EXD3	0	0	1	3	0	0	1	0	0	0
**rs148380088**	9	28759556	28759556	+	ENSG00000174482	LIN-GO2	0	0	0	8	0	0	0	0	0	0
**rs74336249**	9	1.33E+08	1.33E+08	+	ENSG00000165695	AK8	0	0	0	6	0	0	4	0	0	0
**rs148835086**	9	10278984	10278984	+	ENSG00000153707	PTPRD	0	0	0	4	0	0	0	0	0	0
**rs76017116**	9	1.05E+08	1.05E+08	+	ENSG00000186943	OR13C8	0	0	0	0	2	0	0	0	0	0
**rs559632285**	9	41279573	41279573	+	ENSG00000275450	PTGER4P2-CDK2AP2P2	0	0	0	0	0	1	0	0	0	0
**rs34609592**	9	1.34E+08	1.34E+08	+	ENSG00000169925	BRD3	0	0	0	0	2	0	0	0	0	0
**rs7850783**	9	98205944	98205944	+	ENSG00000095383	TBC1D2	0	0	0	5	0	0	0	0	0	0
**rs12378893**	9	76442727	76442727	+	ENSG00000187210	GCNT1	0	0	0	2	0	0	1	0	0	0
**rs188376**	9	99628603	99628603	+	ENSG0000023746	ENSG0000023746	0	0	0	3	0	0	3	0	0	0

**248**					1,ENSG00000255145	1										
**rs10122523**	9	96749469	96749469	+	ENSG00000224848	ENSG00000224848	0	0	0	5	0	0	5	0	0	0
**rs140322068**	10	1723599	1723599	+	ENSG00000185736	ADARB2	0	0	0	1	0	0	0	0	0	0
**rs72810820**	10	90841156	90841156	+	ENSG00000148680	HTR7	0	0	0	3	0	0	0	0	0	0
**rs78622624**	10	33070497	33070497	+	ENSG00000229656	ITGB1-DT	0	0	0	6	0	0	6	0	0	0
**rs191325818**	10	1.19E+08	1.19E+08	+	ENSG00000229272	LINC03036	0	0	0	2	0	0	2	0	0	0
**rs77390603**	10	4646000	4646000	+	ENSG00000231298	MANCR	0	0	0	2	0	0	2	0	0	0
**rs145743664**	10	16953518	16953518	+	ENSG00000107611	CUBN	0	0	0	4	0	0	0	0	0	0
**rs112517700**	10	89254078	89254078	+	ENSG00000107798	LIPA	0	0	0	2	0	0	0	0	0	0
**rs143772909**	10	99734361	99734361	+	ENSG00000119929	CUTC	0	0	2	8	0	0	2	0	0	0
**rs10824158**	10	52156108	52156108	+	ENSG00000185532	PRKG1	0	0	0	14	0	0	4	0	0	0
**rs11202534**	10	87740998	87740998	+	ENSG00000198682	PAPSS2	0	0	0	4	0	0	0	0	0	0
**rs575794055**	10	1.18E+08	1.18E+08	+	ENSG0000017764	CASC2	0	0	0	3	0	2	3	0	0	0

					0											
**rs76385522**	11	10599444	10599444	+	ENSG00000072952,ENSG00000133800,ENSG00000177112	IRAG1	0	0	2	15	0	0	6	0	0	0
**rs78524989**	11	94550968	94550968	+	ENSG00000134627,ENSG00000255929	PIWIL4	0	0	2	4	0	0	2	0	0	0
**rs78085398**	11	33099620	33099620	+	ENSG00000176102,ENSG00000176148	CSTF3	0	0	1	2	0	0	0	0	0	2
**rs186370342**	11	92347966	92347966	+	ENSG00000165323	FAT3	0	0	0	1	0	0	0	0	0	0
**rs1106305**	11	12043540	12043540	+	ENSG00000254486	LINC02547	0	0	0	1	0	0	1	0	0	0
**rs117270364**	11	60926903	60926903	+	ENSG00000006118	TMEM132A	0	0	0	4	0	0	1	0	0	0
**rs7130130**	11	59568856	59568856	+	ENSG00000255139	ENSG00000255139	0	0	0	1	0	0	1	0	0	0
**rs118014538**	11	3809128	3809128	+	ENSG00000148985	PGAP2	0	0	6	33	0	0	9	0	0	0
**rs74540476**	11	29880551	29880551	+	ENSG00000254530,ENSG00000254734	LINC02755	0	0	0	6	0	0	6	0	0	0
**rs776954**	11	7075	7075	+	ENSG00000	SHA	0	0	0	5	0	0	3	0	0	0

**06**		3383	3383		162105	NK2										
**rs79741386**	11	1.1E+08	1.1E+08	+	ENSG00000254659	LINC02715	0	0	0	6	0	0	6	0	0	0
**rs189796120**	11	1.09E+08	1.09E+08	+	ENSG00000255028	ENSG00000255028	0	0	0	1	0	0	1	0	0	0
**rs117420444**	11	31609819	31609819	+	ENSG00000109911	ELP4	0	0	6	21	0	0	1	0	0	0
**rs12279408**	11	84558929	84558929	+	ENSG00000150672	DLG2	0	0	1	16	0	0	3	0	0	0
**rs116916598**	11	1.22E+08	1.22E+08	+	ENSG00000286044	ENSG00000286044	0	0	0	2	0	0	2	0	0	0
**rs11060236**	12	1.29E+08	1.29E+08	+	ENSG00000151952	TMEM132D	0	0	0	1	0	0	0	0	0	0
**rs77577050**	12	1.16E+08	1.16E+08	+	ENSG00000257407	ENSG00000257407	0	0	0	3	0	0	3	0	0	0
**rs144682449**	12	53008854	53008854	+	ENSG00000063046	EIF4B	0	0	3	11	0	0	2	0	0	0
**rs117785622**	12	1.32E+08	1.32E+08	+	ENSG00000061936	SFSWAP	0	0	0	0	4	4	0	0	8	0
**rs10505739**	12	9890919	9890919	+	ENSG00000256797	KLRF2	0	0	0	1	0	0	0	0	0	0
**rs188074116**	12	19158302	19158302	+	ENSG00000052126	PLEKHA5	0	0	2	17	0	0	10	0	0	0
**rs112529428**	12	1.29E+08	1.29E+08	+	ENSG00000151948	GLT1D1	0	0	9	15	0	0	0	0	0	0

**rs714660 29**	12	8167 1655	8167 1655	+	ENSG 00000 13922 0	PPFI A2	0	0	0	8	0	0	1	0	0	0
**rs787738 52**	12	7078 8150	7078 8150	+	ENSG 00000 15323 3	PTPR R	0	0	0	2	0	0	0	0	0	0
**rs147107 195**	12	4779 6492	4779 6492	+	ENSG 00000 06127 3	HDA C7	0	0	3	10	0	0	1	0	0	0
**rs352756 93**	12	8816 7702	8816 7702	+	ENSG 00000 13932 4	TMT C3	0	0	1	2	0	0	0	0	0	0
**rs117613 430**	12	9314 7158	9314 7158	+	ENSG 00000 25732 2	ENSG 00000 25732 2	0	0	0	1	0	0	1	0	0	0
**rs117293 367**	12	5204 3654	5204 3654	+	ENSG 00000 12335 8	NR4A 1	0	0	0	18	0	3	0	0	0	0
**rs146661 200**	12	1E+0 8	1E+0 8	+	ENSG 00000 11164 7	BLTP 3B	0	0	0	2	0	0	0	0	0	0
**rs753919 39**	12	1.33 E+08	1.33 E+08	+	ENSG 00000 07260 9	CHFR	0	0	1	3	0	0	0	0	0	6
**rs384801 8**	13	9838 2298	9838 2298	+	ENSG 00000 15276 7	FARP 1	0	0	0	12	0	1	6	0	0	0
**rs117075 995**	13	1929 5115	1929 5115	+	ENSG 00000 23763 6	ANK RD26 P3	0	0	0	2	0	0	2	0	0	0
**rs111648 746**	13	2791 1208	2791 1208	+	ENSG 00000 24738 1	PLUT	0	0	0	1	0	0	1	0	0	0
**rs186400 376**	13	3123 1021	3123 1021	+	ENSG 00000 18767 6	B3GL CT	0	0	0	1	0	0	0	0	0	0
**rs113007 178**	13	4034 0420	4034 0420	+	ENSG 00000 21548	LINC 00598	0	0	0	8	0	0	8	0	0	0

					3											
**rs74565504**	14	88740803	88740803	+	ENSG00000165521	EML5	0	0	0	2	0	0	0	0	0	0
**rs113699161**	14	58104871	58104871	+	ENSG00000139971	ARMH4	0	0	0	1	0	0	0	0	0	0
**rs80334718**	14	33529313	33529313	+	ENSG00000151322	NPAS3	0	0	3	33	0	0	3	0	0	0
**rs78750136**	14	25839738	25839738	+	ENSG00000257986	LINC02306	0	0	0	2	0	0	2	0	0	0
**rs150889386**	14	1E+08	1E+08	+	ENSG00000140105	WARS1	0	0	0	20	0	0	5	0	0	0
**rs71419987**	14	34450511	34450511	+	ENSG00000129521,ENSG00000165389	EGLN3,SPTSSA	0	0	0	2	0	0	1	0	0	0
**rs61755651**	14	71964882	71964882	+	ENSG00000182732	RGS6	0	0	2	18	0	0	0	0	18	0
**rs146937045**	14	46271299	46271299	+	ENSG00000258700	LINC00871	0	0	0	5	0	0	5	0	0	0
**rs192467351**	14	76360262	76360262	+	ENSG00000119715	ESRRB	0	0	0	1	0	0	0	0	0	0
**rs10484068**	14	99186786	99186786	+	ENSG00000127152	BCL11B	0	0	0	3	0	0	0	0	0	0
**rs117801457**	15	29128910	29128910	+	ENSG00000104059	EN-TREP2	0	0	0	2	0	0	1	0	0	0
**rs149438077**	15	29942170	29942170	+	ENSG0000010406	TJP1	0	0	0	2	0	0	1	0	0	0

					7											
**rs149908314**	15	54052722	54052722	+	ENSG00000137766	UNC13C	0	0	0	2	0	0	0	0	0	0
**rs75669615**	15	39376559	39376559	+	ENSG00000259345	ENSG00000259345	0	0	0	3	0	0	3	0	0	0
**rs117211386**	15	51357402	51357402	+	ENSG00000186417	GLDN	0	0	0	4	0	0	2	0	0	0
**rs79548047**	16	11520982	11520982	+	ENSG00000188897	ENSG00000188897	0	0	0	2	0	0	0	0	0	0
**rs117555426**	16	13034319	13034319	+	ENSG00000237515	SHISA9	0	0	0	1	0	0	0	0	0	0
**rs151246819**	16	23362634	23362634	+	ENSG00000168447	SCNN1B	0	0	1	6	0	0	0	0	0	0
**rs34265859**	16	25920469	25920469	+	ENSG00000182601,ENSG00000285882	HS3ST4	0	0	0	3	0	0	2	0	0	0
**rs117898578**	16	50437373	50437373	+	ENSG00000260573	ENSG00000260573	0	0	0	1	0	0	1	0	0	0
**rs143946135**	16	68989786	68989786	+	ENSG00000103047	TANGO6	0	0	0	3	0	0	2	0	0	0
**rs142076427**	16	5578077	5578077	+	ENSG00000078328	RBFOX1	0	0	0	6	0	0	2	0	0	0
**rs55856613**	17	81120362	81120362	+	ENSG00000181409	AATK	4	0	4	2	4	0	2	0	0	0
**rs143393137**	17	32640367	32640367	+	ENSG00000176658,ENS	MYO1D	0	0	0	6	0	0	3	0	0	0

					G000002644 58											
**rs144104650**	17	5120262	5120262	+	ENSG00000129204,ENSG00000167840	USP6, ZNF232	0	0	2	4	0	0	1	0	0	0
**rs78100215**	17	8395555	8395555	+	ENSG000001890 51	RNF222	0	0	0	1	0	0	0	0	0	0
**rs79757812**	17	67900873	67900873	+	ENSG00000171634	BPTF	0	0	0	7	0	0	0	0	0	0
**rs139736375**	17	69629497	69629497	+	ENSG00000227517	LINC01483	0	0	0	2	0	0	2	0	0	0
**rs34539402**	17	41803375	41803375	+	ENSG00000141696	P3H4	0	0	1	0	0	1	0	0	0	3
**rs17882143**	17	43758714	43758714	+	ENSG00000167941	SOST	0	0	0	0	2	0	0	0	0	0
**rs77725246**	17	57561851	57561851	+	ENSG00000153944	MSI2	0	0	2	14	0	0	4	0	0	0
**rs150994087**	18	59041560	59041560	+	ENSG00000224367,ENSG00000293539	OACYLP	0	0	0	2	0	0	2	0	0	0
**rs117745825**	18	3305230	3305230	+	ENSG00000266578	ENSG00000266578	0	0	0	1	0	0	1	0	0	0
**rs116618 82**	18	72837233	72837233	+	ENSG00000166342	NE-TO1	0	0	0	12	0	0	3	0	0	0
**rs145978106**	18	7839217	7839217	+	ENSG0000017348	PTPRM	0	0	0	8	0	0	0	0	0	0

					2											
**rs76808079**	18	25155975	25155975	+	ENSG00000198795	ZNF521	0	0	1	5	0	0	0	0	0	0
**rs147136626**	18	76189743	76189743	+	ENSG00000266743	ENSG00000266743	0	0	0	4	0	0	4	0	0	0
**rs146989801**	19	45127102	45127102	+	ENSG00000007047,ENSG00000104866	MARK4	0	0	0	4	0	0	1	0	0	0
**rs200198801**	19	45884683	45884683	+	ENSG00000170604	IRF2BP1	0	0	0	0	0	0	0	0	0	1
**rs77955766**	19	17196293	17196293	+	ENSG00000099331	MYO9B	0	0	0	5	0	0	0	0	0	0
**rs140800343**	19	49497913	49497913	+	ENSG00000142534	RPS11	0	0	1	7	0	2	2	7	7	0
**rs57121931**	19	54506474	54506474	+	ENSG00000167618	LAIR2	0	0	0	4	0	0	0	0	0	0
**rs143871291**	19	1191668	1191668	+	ENSG00000118046	STK11	0	0	0	2	0	0	0	0	0	0
**rs146101423**	19	6752989	6752989	+	ENSG00000125731	SH2D3A	0	0	0	3	0	0	1	0	0	0
**rs78703636**	19	33462689	33462689	+	ENSG00000124299	PEPD	4	0	8	48	0	2	14	0	0	0
**rs73034498**	19	44433260	44433260	+	ENSG00000267188,ENSG00000278318	ENSG00000267188	0	0	2	12	0	0	4	0	0	0

**rs147572731**	19	48912914	48912914	+	ENSG00000104805,ENSG00000235191	NUCB1	0	0	6	26	0	0	7	0	0	0
**rs180972485**	20	24476394	24476394	+	ENSG00000101463	SYNDIG1	0	0	0	1	0	0	0	0	0	0
**rs137942933**	20	34516317	34516317	+	ENSG00000289720	ENSG00000289720	0	0	5	5	0	0	0	0	0	0
**rs138246394**	20	55653903	55653903	+	ENSG00000235166,ENSG00000287478	LINC01440	0	0	0	6	0	0	6	0	0	0
**rs41276984**	20	38952027	38952027	+	ENSG00000101447	FAM83D	0	0	0	0	2	0	0	0	0	0
**rs11538695**	20	51388023	51388023	+	ENSG00000101096	NFATC2	2	0	0	0	0	0	0	0	0	0
**rs145250558**	20	15264932	15264932	+	ENSG00000172264	MACROD2	0	0	0	4	0	0	0	0	0	0
**rs17791728**	20	17575942	17575942	+	ENSG00000125868	DSTN	0	0	2	6	0	0	0	0	0	0
**rs75600477**	20	41569289	41569289	+	ENSG00000124177	CHD6	0	0	0	4	0	0	1	0	0	0
**rs117922572**	21	25917559	25917559	+	ENSG00000142192	APP	0	0	0	22	0	0	6	0	0	0
**rs6517382**	21	36783869	36783869	+	ENSG00000159267	HLCS	0	0	3	18	0	0	0	0	0	0
**rs111714**	21	34159937	34159937	+	ENSG0000022745	LINC00310	0	0	0	2	0	0	2	0	0	0

**479**					6,ENSG00000272657											
**rs146461857**	22	23367771	23367771	+	ENSG00000215478,ENSG00000290449	CES5AP1	0	0	0	2	0	0	2	0	0	0
**rs482184 5**	22	38958386	38958386	+	ENSG00000128383	APOBEC3A	0	0	0	9	0	0	3	0	0	0
**rs114581990**	2	2.35E+08	2.35E+08	+	ENSG00000130147	SH3BP4	0	0	0	3	0	0	0	0	0	0
**rs117692894**	7	39547409	39547409	+	ENSG00000227172	YAE1-DT	0	0	0	1	0	0	1	0	0	0
**rs117478414**	8	55168468	55168468	+	ENSG00000206579	XKR4	0	0	0	1	0	0	0	0	0	0
**rs117173207**	11	24522517	24522517	+	ENSG00000187398	LUZP2	0	0	0	12	0	0	6	0	0	0
**rs77113660**	12	82856037	82856037	+	ENSG00000179104	TMTC2	0	0	1	5	0	0	1	0	0	0
**rs35068665**	14	1.01E+08	1.01E+08	+	ENSG00000258460	ENSG00000258460	0	0	0	1	0	0	1	0	0	0
**rs72758450**	15	69518710	69518710	+	ENSG00000245750	DRAIC	0	0	0	24	0	0	24	0	0	0
**rs80331554**	15	58721444	58721444	+	ENSG00000137845	AD-AM10	0	0	0	20	0	0	8	0	0	0
**rs147093663**	15	81130561	81130561	+	ENSG00000156206	CFAP161	0	0	0	4	0	0	0	0	0	0

**rs357723 93**	16	47693603	47693603	+	ENSG000001028 93	PHKB	0	0	2	6	0	0	1	0	0	0
**rs774673 80**	16	78628189	78628189	+	ENSG000001861 53	WWOX	0	0	2	8	0	0	1	0	0	0
**rs191923 221**	19	18747944	18747944	+	ENSG000001056 62	CRTC1	0	0	0	4	0	0	0	0	0	0
**rs493938 4**	11	60506169	60506169	+	ENSG000000712 03	MS4A12	0	0	0	6	0	0	0	0	0	0
**rs740489 0**	16	10913336	10913336	+	ENSG000001795 83	CII-TA	0	0	2	12	0	0	2	0	0	0
**rs344867 65**	19	49412654	49412654	+	ENSG000001616 09	KASH5	0	0	0	6	0	0	2	0	0	0
**rs123425 39**	9	4327960	4327960	+	ENSG000001072 49	GLIS3	0	0	0	2	0	0	2	0	0	0
**rs127225 31**	10	6034113	6034113	+	ENSG000001344 60,ENSG000002296 64	IL2RA	0	0	0	7	0	0	1	0	0	0
**rs180127 4**	1	1.62 E+08	1.62 E+08	+	ENSG000001432 26	FCGR2A	3	0	6	0	21	6	0	0	0	0
**rs341015 71**	1	30739047	30739047	+	ENSG000001625 11	LAPTM5	0	0	0	0	1	1	0	0	0	0
**rs762149 2**	3	32260964	32260964	+	ENSG000001702 93	CMTM8	0	0	0	2	0	0	0	0	0	0
**rs355748 03**	3	32491754	32491754	+	ENSG000000913 17	CMTM6	0	0	0	0	2	2	0	0	0	0

**rs25680**	12	6445462	6445462	+	ENSG00000139193,ENSG00000215039	CD27	0	0	0	26	4	2	26	0	0	0
**rs147714617**	12	6496133	6496133	+	ENSG00000010292	NCAPD2	0	0	1	5	0	0	1	0	0	0
**rs110869 84**	20	45882988	45882988	+	ENSG00000168612	ZSWIM1	0	0	0	0	0	0	0	0	0	1
**rs1883832**	20	46118343	46118343	+	ENSG00000101017	CD40	0	15	3	0	0	9	0	0	0	0
**rs62442539**	7	2312959	2312959	+	ENSG00000106266	SNX8	0	0	0	12	0	0	3	0	0	0
**rs709589**	14	1.06E+08	1.06E+08	+	ENSG00000211898	IGHD	0	0	0	0	2	0	0	0	0	1
**rs2018404**	14	1.05E+08	1.05E+08	+	ENSG00000185024	BRF1	0	0	0	36	0	0	0	0	0	0
**rs28669075**	15	40564322	40564322	+	ENSG00000128891	CCDC32	0	0	2	26	0	0	2	0	0	0
**rs78307830**	2	2.04E+08	2.04E+08	+	ENSG00000178562	CD28	0	0	0	3	0	0	0	0	0	0
**rs9823718**	3	39252266	39252266	+	ENSG00000287958	ENSG00000287958	0	0	0	2	0	0	2	0	0	0
**rs72865957**	3	39314428	39314428	+	ENSG00000287780	ENSG00000287780	0	0	0	2	0	0	2	0	0	0
**rs71632979**	1	1.62E+08	1.62E+08	+	ENSG00000224515,ENSG0000002731	ENSG00000224515	0	0	0	8	0	0	4	0	0	0

					12,ENSG00000289768											
**rs139795227**	1	92376810	92376810	+	ENSG00000122484	RPAP2	0	0	0	2	0	0	1	0	0	0
**rs6445484**	3	52469784	52469784	+	ENSG00000010322	NISCH	0	0	0	10	0	0	2	0	0	0
**rs76064946**	1	93461140	93461140	+	ENSG00000137942	FNBP1L	0	0	0	6	0	0	0	0	0	0
**rs79015439**	1	94337568	94337568	+	ENSG00000226835	ARHGAP29-AS1	0	0	0	2	0	0	2	0	0	0
**rs35075155**	3	53239179	53239179	+	ENSG00000163931	TKT	0	0	3	7	0	0	1	0	0	0

Table 12. A table listing all significant immune, microbiota, lipid, and metabolite associations in DMiVD, including effect sizes, confidence intervals, and FDR-adjusted *p*-values.

**id.exposure**	**id.outcome**	**outcome**	**exposure**	**method**	**nsnp**	**b**	**se**	**pval**	**lo_ci**	**up_ci**	**or**	**or_lci95**	**or_uci95**	**FDR**
ebi-a-GCST90001697	Ut2VMm	Diabetic Nephropathy	CD45RA- CD28- CD8br %T cell	IVW	41	-0.00103	0.000389	0.00833	-0.00179	-0.00026	0.998973	0.998211	0.999736	0.495973
ebi-a-GCST90001869	Ut2VMm	Diabetic Nephropathy	CD3 on resting Treg	IVW	6	0.050406	0.024762	0.041788	0.001873	0.09894	1.051698	1.001874	1.104	0.867392
tXchOq	L8xX0y	Diabetic Nephropathy	Family Enterobacteriaceae	IVW	10	-0.28135	0.096553	0.003569	-0.4706	-0.09211	0.754762	0.624629	0.912006	0.999206
mBXqB5	L8xX0y	Diabetic Nephropathy	Genus Desulfovibrionia	IVW	6	0.333647	0.137283	0.015083	0.064574	0.602721	1.396051	1.066704	1.827084	0.999206
2rt317	L8xX0y	Diabetic Nephropathy	Genus Citrobacter A	IVW	8	0.45902	0.195541	0.018903	0.07576	0.842281	1.582523	1.078703	2.321657	0.999206
reZW6G	L8xX0y	Diabetic Nephropathy	Genus Syntrophorhabdia	IVW	6	-1.21303	0.535879	0.023597	-2.26336	-0.16271	0.297294	0.104001	0.849838	0.999206

qcoHvy	L8xX0y	Diabetic Nephropathy	Genus Leuconostoc	IVW	5	0.358675	0.162801	0.027584	0.039585	0.677764	1.431431	1.040379	1.96947	0.999206
uRGp1t	L8xX0y	Diabetic Nephropathy	Family Desulfovibrionaceae	IVW	8	0.211657	0.100639	0.035455	0.014404	0.40891	1.235724	1.014508	1.505177	0.999206
zL970M	L8xX0y	Diabetic Nephropathy	Unclassified CAG-145	IVW	5	0.346362	0.169805	0.041374	0.013544	0.679179	1.413914	1.013636	1.972259	0.999206
GCST90277308	miIBxl	Diabetic Nephropathy	Phosphatidylcholine (18:1_18:1)	IVW	4	0.239854	0.07447	0.001278	0.093893	0.385814	1.271063	1.098443	1.470812	0.167939
GCST90277366	miIBxl	Diabetic Nephropathy	Phosphatidylinositol (18:1_18:2)	IVW	3	0.240176	0.08575	0.005096	0.072106	0.408247	1.271473	1.074769	1.504179	0.167939
GCST90277298	miIBxl	Diabetic Nephropathy	Phosphatidylcholine (17:0_20:4)	IVW	6	-0.08939	0.034108	0.008773	-0.15624	-0.02254	0.91449	0.855354	0.977714	0.167939
GCST90277282	miIBxl	Diabetic Nephropathy	Phosphatidylcholine (16:0_18:2)	IVW	7	0.118043	0.045981	0.010252	0.02792	0.208166	1.125293	1.028314	1.231417	0.171718
GCST90277309	miIBxl	Diabetic Nephropathy	Phosphatidylcholine (18:1_18:2)	IVW	5	0.120825	0.048422	0.012586	0.025919	0.215731	1.128427	1.026257	1.240769	0.187394
GCST90277313	miIBxl	Diabetic Nephropathy	Phosphatidylcholine (18:1_20:4)	IVW	7	-0.0847	0.035334	0.016521	-0.15396	-0.01545	0.918785	0.857308	0.984671	0.206668
GCST90277287	miIBxl	Diabetic Nephropathy	Phosphatidylcholine (16:0_20:4)	IVW	5	-0.07137	0.031591	0.023869	-0.13329	-0.00945	0.931117	0.875213	0.990592	0.206668
GCST90277345	miIBxl	Diabetic Nephropathy	Phosphatidylethanolamine (16:0_18:2)	IVW	6	0.083611	0.037358	0.025216	0.010389	0.156833	1.087206	1.010443	1.1698	0.206668
GCST90277330	miIBxl	Diabetic Nephropathy	Phosphatidylcholine (O-16:1_20:4)	IVW	3	-0.09326	0.043853	0.033453	-0.17921	-0.00731	0.910958	0.83593	0.99272	0.249036
GCST90277276	miIBxl	Diabetic Nephropathy	Phosphatidylcholine (15:0_18:2)	IVW	6	0.116421	0.057137	0.041593	0.004432	0.22841	1.123469	1.004442	1.256601	0.253341
GCST90277247	miIBxl	Diabetic Nephropathy	Sterol ester (27:1/18:3)	IVW	5	-0.14094	0.071288	0.048044	-0.28066	-0.00121	0.868545	0.755284	0.998791	0.274813
YRvUi4	iSWEwg	Diabetic Nephropathy	Retinol (Vitamin A) to linoleoyl-arachidonoyl-glycerol (18:2 to 20:4) [2]	IVW	3	0.148477	0.055282	0.007235	0.040124	0.25683	1.160066	1.04094	1.292825	0.494248
coUkT0	iSWEwg	Diabetic Nephropathy	5,6-dihydrouridine	IVW	4	-0.2612	0.100979	0.009689	-0.45912	-0.06329	0.770124	0.631838	0.938675	0.494248

rv3dxu	iSWEwg	Diabetic Nephropathy	Metabolonic lactone sulfate	IVW	9	-0.07155	0.028159	0.011052	-0.12675	-0.01636	0.930946	0.880957	0.983771	0.494248
Snu3Xv	iSWEwg	Diabetic Nephropathy	X-25371	IVW	4	-0.16844	0.066732	0.011598	-0.29924	-0.03765	0.84498	0.741384	0.963053	0.494248
uarAxE	iSWEwg	Diabetic Nephropathy	Aspartate to N-acetylglucosamine to N-acetylgalactosamine	IVW	3	-0.19288	0.07873	0.014287	-0.34719	-0.03857	0.824577	0.706668	0.96216	0.494248
ra3RHU	iSWEwg	Diabetic Nephropathy	Hydroxypalmitoyl sphingomyelin (d18:1/16:0(OH))	IVW	3	0.160493	0.065948	0.014948	0.031235	0.289752	1.17409	1.031728	1.336096	0.494248
MTO5rN	iSWEwg	Diabetic Nephropathy	1,2-dilinoleoyl-GPE (18:2/18:2)	IVW	3	0.172872	0.072026	0.016389	0.031702	0.314042	1.188714	1.03221	1.368947	0.494248
1eMa1l	iSWEwg	Diabetic Nephropathy	1-(1-enyl-stearoyl)-2-linoleoyl-GPE (p-18:0/18:2)	IVW	3	0.19777	0.08343	0.017765	0.034247	0.361293	1.218682	1.03484	1.435183	0.494248
Wt3ZoP	iSWEwg	Diabetic Nephropathy	1-(1-enyl-palmitoyl)-GPC (p-16:0)	IVW	3	-0.20641	0.088179	0.019241	-0.37924	-0.03358	0.813498	0.68438	0.966977	0.494248
n91Tzn	iSWEwg	Diabetic Nephropathy	1-(1-enyl-palmitoyl)-2-arachidonoyl-gpc (p-16:0/20:4)	IVW	3	-0.08788	0.037772	0.01999	-0.16191	-0.01384	0.915874	0.850519	0.986251	0.494248
JpLbOz	iSWEwg	Diabetic Nephropathy	X-26109	IVW	6	-0.06964	0.030051	0.020475	-0.12854	-0.01074	0.932726	0.879375	0.989313	0.494248
Dehpjt	iSWEwg	Diabetic Nephropathy	1-stearoyl-2-docosahexaenoyl-gpc (18:0/22:6)	IVW	3	-0.17499	0.075525	0.020507	-0.32302	-0.02696	0.839467	0.723961	0.973402	0.494248
qpoBMf	iSWEwg	Diabetic Nephropathy	1-stearoyl-2-linoleoyl-gpc (18:0/18:2)	IVW	3	0.177585	0.078064	0.022913	0.024581	0.33059	1.19433	1.024885	1.391789	0.494248
NXkYyo	iSWEwg	Diabetic Nephropathy	1-oleoyl-2-linoleoyl-GPE (18:1/18:2)	IVW	5	0.089571	0.039492	0.023324	0.012167	0.166974	1.093705	1.012241	1.181724	0.494248
qaoUHO	iSWEwg	Diabetic Nephropathy	Cholesterol to linoleoyl-arachidonoyl-glycerol (18:2 to 20:4) [1]	IVW	3	0.140721	0.062066	0.023373	0.019072	0.262371	1.151104	1.019255	1.300009	0.494248
v8PJsP	iSWEwg	Diabetic Nephropathy	Retinol (Vitamin A) to linoleoyl-arachidonoyl-glycerol (18:2 to 20:4) [1]	IVW	4	0.127688	0.056574	0.024008	0.016803	0.238574	1.136199	1.016945	1.269437	0.494248
DSygCD	iSWEwg	Diabetic Nephropathy	1-arachidonoyl-GPE (20:4n6)	IVW	4	-0.09501	0.0421	0.024029	-0.17752	-0.01249	0.909367	0.837341	0.987588	0.494248
mTmMwi	iSWEwg	Diabetic Nephropathy	Dodecadienoate (12:2)	IVW	5	0.180195	0.079879	0.02408	0.023632	0.336759	1.197451	1.023913	1.400401	0.494248

8XQY7L	iSWEwg	Diabetic Nephropathy	Phosphate to linoleoyl-arachidonoyl-glycerol (18:2 to 20:4) [1]	IVW	3	0.127851	0.057231	0.025487	0.015678	0.240024	1.136384	1.015801	1.27128	0.494248
y0DlUg	iSWEwg	Diabetic Nephropathy	1-arachidonoyl-gpc (20:4n6)	IVW	5	-0.08258	0.03753	0.027772	-0.15614	-0.00903	0.920734	0.855438	0.991015	0.494248
sFTSwZ	iSWEwg	Diabetic Nephropathy	X-26111	IVW	5	-0.13309	0.060666	0.028254	-0.25199	-0.01418	0.87539	0.777251	0.98592	0.494248
6XVwdY	iSWEwg	Diabetic Nephropathy	1-palmitoyl-2-linoleoyl-GPI (16:0/18:2)	IVW	5	0.170117	0.079058	0.031413	0.015164	0.32507	1.185443	1.015279	1.384128	0.494248
XBLY8d	iSWEwg	Diabetic Nephropathy	N-stearoyl-sphingosine (d18:1 to 18:0) to N-palmitoyl-sphinganine (d18:0 to 16:0)	IVW	5	-0.16461	0.07679	0.032066	-0.31511	-0.0141	0.848229	0.729706	0.986002	0.494248
fz6Z1H	iSWEwg	Diabetic Nephropathy	Vanillic acid glycine	IVW	4	-0.19579	0.091343	0.032077	-0.37482	-0.01676	0.822185	0.687411	0.983382	0.494248
JGu6Jo	iSWEwg	Diabetic Nephropathy	1-linoleoyl-GPG (18:2)	IVW	4	-0.09868	0.046442	0.033604	-0.18971	-0.00765	0.906033	0.827203	0.992376	0.494248
mPvTc3	iSWEwg	Diabetic Nephropathy	Tetradecanedioate (C14-DC)	IVW	6	-0.08116	0.038844	0.036664	-0.1573	-0.00503	0.922042	0.854448	0.994983	0.494248
XxxFFN	iSWEwg	Diabetic Nephropathy	X-18922	IVW	6	-0.11552	0.055723	0.038168	-0.22473	-0.0063	0.890907	0.798731	0.993721	0.494248
sHOk2I	iSWEwg	Diabetic Nephropathy	Hexadecenedioate (C16:1-DC)	IVW	4	-0.06593	0.031844	0.038424	-0.12834	-0.00351	0.9362	0.879555	0.996494	0.494248
MZUqvW	iSWEwg	Diabetic Nephropathy	Caffeine to paraxanthine	IVW	3	-0.15149	0.073748	0.039953	-0.29604	-0.00695	0.859423	0.743758	0.993075	0.494248
BmdUs4	iSWEwg	Diabetic Nephropathy	1-oleoyl-GPG (18:1)	IVW	5	-0.09019	0.044056	0.040639	-0.17654	-0.00384	0.913756	0.838164	0.996166	0.494248
WpyQmq	iSWEwg	Diabetic Nephropathy	N2-acetyl,N6,N6-dimethyllysine	IVW	6	0.052065	0.025504	0.041205	0.002078	0.102052	1.053444	1.00208	1.107441	0.494248
cTiRBD	iSWEwg	Diabetic Nephropathy	21-hydroxypregnenolone disulfate	IVW	7	-0.13305	0.065272	0.041513	-0.26098	-0.00512	0.875423	0.770295	0.994898	0.494248
2mQRTz	iSWEwg	Diabetic Nephropathy	Sphingomyelin (d18:2/21:0, d16:2/23:0)	IVW	4	-0.1998	0.099102	0.043794	-0.39404	-0.00556	0.818898	0.67433	0.99446	0.494248
sRrNSM	iSWEwg	Diabetic Nephropathy	1-palmitoyl-2-linoleoyl-GPE (16:0/18:2)	IVW	6	0.065678	0.032954	0.046257	0.001089	0.130267	1.067883	1.001089	1.139132	0.497704

gqZODE	iSWEwg	Diabetic Nephropathy	5-methylthioadenosine (MTA) to phosphate	IVW	4	0.207452	0.105384	0.049007	0.000899	0.414005	1.230539	1.000899	1.512865	0.497704
SHDhX0	iSWEwg	Diabetic Nephropathy	Linoleoyl-arachidonoyl-glycerol (18:2/20:4) [1]	IVW	3	-0.10365	0.052833	0.049779	-0.20721	-9.80E-05	0.901539	0.812853	0.999902	0.497704
ieu-a-1077	AosMqV	Diabetic Nephropathy	Selenium	IVW	6	-0.09601	0.044382	0.030521	-0.183	-0.00902	0.908456	0.832771	0.991019	0.457813
ebi-a-GCST90001787	WISnV1	Diabetic Retinopathy	CD25 on IgD- CD38- B cell	IVW	3	0.137703	0.045215	0.002323	0.049082	0.226324	1.147635	1.050307	1.253982	0.128906
ebi-a-GCST90001798	WISnV1	Diabetic Retinopathy	CD27 on CD24+ CD27+ B cell	IVW	11	-0.05445	0.018796	0.00377	-0.09129	-0.01761	0.947008	0.912755	0.982546	0.136757
ebi-a-GCST90001896	WISnV1	Diabetic Retinopathy	CD28 on CD28+ CD45RA+ CD8br Treg	IVW	3	0.093392	0.032266	0.003798	0.030151	0.156633	1.097892	1.03061	1.169566	0.136757
ebi-a-GCST90001808	WISnV1	Diabetic Retinopathy	CD27 on sw mem B cell	IVW	10	-0.0506	0.017714	0.00428	-0.08532	-0.01588	0.950655	0.918216	0.984241	0.136757
ebi-a-GCST90001752	WISnV1	Diabetic Retinopathy	CD20 on IgD+ CD38dim B cell	IVW	7	0.109125	0.038231	0.004312	0.034193	0.184058	1.115302	1.034784	1.202085	0.136757
ebi-a-GCST90002039	WISnV1	Diabetic Retinopathy	CD80 on monocyte cDC	IVW	6	0.054759	0.021416	0.010561	0.012783	0.096735	1.056286	1.012865	1.101568	0.264644
ebi-a-GCST90001804	WISnV1	Diabetic Retinopathy	CD27 on IgD- CD38dim B cell	IVW	9	-0.04677	0.018331	0.010729	-0.0827	-0.01084	0.954306	0.920627	0.989217	0.264644
ebi-a-GCST90001996	WISnV1	Diabetic Retinopathy	CX3CR1 on CD14+ CD16+ monocyte	IVW	3	-0.06113	0.025577	0.01685	-0.11126	-0.011	0.940703	0.894707	0.989063	0.298233
ebi-a-GCST90001997	WISnV1	Diabetic Retinopathy	CX3CR1 on CD14+ CD16- monocyte	IVW	3	-0.05463	0.023378	0.019461	-0.10045	-0.0088	0.94684	0.904433	0.991235	0.299053
ebi-a-GCST90001995	WISnV1	Diabetic Retinopathy	CX3CR1 on monocyte	IVW	3	-0.06011	0.025739	0.019533	-0.11055	-0.00966	0.941665	0.895339	0.990389	0.299053
ebi-a-GCST90001698	WISnV1	Diabetic Retinopathy	CD45RA+ CD28- CD8br AC Treg	IVW	426	8.05E-06	3.53E-06	0.022501	1.14E-06	1.50E-05	1.000008	1.000001	1.000015	0.322896
ebi-a-GCST90002035	WISnV1	Diabetic Retinopathy	CD80 on myeloid DC	IVW	4	0.082194	0.036033	0.022545	0.011569	0.152818	1.085666	1.011636	1.165113	0.322896
ebi-a-GCST90002036	WISnV1	Diabetic Retinopathy	CD80 on CD62L+ myeloid DC	IVW	3	0.091145	0.040189	0.023335	0.012374	0.169915	1.095428	1.012451	1.185204	0.323777

ebi-a-GCST90001824	WISnV1	Diabetic Retinopathy	IgD on IgD+ CD38br B cell	IVW	6	-0.03706	0.016641	0.025935	-0.06968	-0.00445	0.963615	0.932692	0.995564	0.338681
ebi-a-GCST90001828	WISnV1	Diabetic Retinopathy	IgD on transitional B cell	IVW	5	-0.04653	0.021601	0.031217	-0.08887	-0.0042	0.954532	0.914963	0.995812	0.396013
ebi-a-GCST90001700	WISnV1	Diabetic Retinopathy	CD45RA+ CD28- CD8br %T cell	IVW	67	0.000359	0.000169	0.034251	2.66E-05	0.00069	1.000359	1.000027	1.000691	0.411008
A8IUc9	Gr249B	Diabetic Retinopathy	Unclassified Bacilli A	IVW	8	1.959912	0.664374	0.003178	0.657739	3.262085	7.098701	1.930423	26.1039	0.983698
fS7KV7	Gr249B	Diabetic Retinopathy	Family Bifidobacteriaceae	IVW	8	-0.15672	0.056372	0.005433	-0.26721	-0.04624	0.85494	0.76551	0.954817	0.983698
RzQClC	Gr249B	Diabetic Retinopathy	Species CAG-177 sp003538135	IVW	8	0.182157	0.066656	0.00628	0.051511	0.312803	1.199802	1.052861	1.367252	0.983698
D1XMKf	Gr249B	Diabetic Retinopathy	Genus An181	IVW	6	0.331333	0.128306	0.009812	0.079855	0.582812	1.392824	1.08313	1.791068	0.983698
COcXXQ	Gr249B	Diabetic Retinopathy	Species Pseudomonas aeruginosa	IVW	7	0.463317	0.187802	0.013623	0.095224	0.831409	1.589337	1.099905	2.296553	0.983698
7JQHVW	Gr249B	Diabetic Retinopathy	Species Lachnospira rogosae	IVW	10	0.129634	0.05303	0.014504	0.025695	0.233572	1.138411	1.026028	1.263104	0.983698
h1l8Fe	Gr249B	Diabetic Retinopathy	Species Blautia hansenii	IVW	11	0.194747	0.081391	0.016724	0.03522	0.354274	1.215004	1.035848	1.425146	0.983698
MY5ejN	Gr249B	Diabetic Retinopathy	Genus Holdemania	IVW	12	0.150078	0.066286	0.023569	0.020156	0.279999	1.161925	1.020361	1.323129	0.983698
2ZKx87	Gr249B	Diabetic Retinopathy	Unclassified Geobacter C	IVW	6	0.5319	0.237508	0.025123	0.066385	0.997416	1.702164	1.068638	2.711266	0.983698
GOJ1Bi	Gr249B	Diabetic Retinopathy	Species Bacteroides stercoris	IVW	7	-0.13175	0.061136	0.031162	-0.25157	-0.01192	0.876562	0.777576	0.98815	0.983698
XxnkAU	Gr249B	Diabetic Retinopathy	Unclassified Firmicutes A	IVW	7	0.649523	0.305134	0.033284	0.05146	1.247586	1.914627	1.052807	3.481928	0.983698
UcIDhn	Gr249B	Diabetic Retinopathy	Species Clostridium M sp001304855	IVW	8	-0.2536	0.126989	0.045823	-0.5025	-0.0047	0.776002	0.605017	0.995309	0.983698
qH71DW	Gr249B	Diabetic Retinopathy	Order Actinomycetales	IVW	4	-0.18097	0.090686	0.045979	-0.35872	-0.00323	0.834458	0.698571	0.996778	0.983698

tU2Sl8	Gr249B	Diabetic Retinopathy	Unclassified CAG-475	IVW	14	-0.10034	0.050739	0.047976	-0.19979	-0.00089	0.90453	0.818904	0.999108	0.983698
GCST90274788	Z5sxMQ	Diabetic Retinopathy	FGF21	IVW	4	-0.12294	0.054748	0.02473	-0.23025	-0.01564	0.884316	0.794338	0.984486	0.362116
GCST90274787	Z5sxMQ	Diabetic Retinopathy	FGF19	IVW	3	-0.13436	0.063246	0.033635	-0.25832	-0.0104	0.874275	0.772347	0.989655	0.362116
GCST90277400	9ATSFc	Diabetic Retinopathy	Triacylglycerol (52:6)	IVW	4	-0.16359	0.050107	0.001096	-0.2618	-0.06538	0.849091	0.769666	0.936713	0.076104
GCST90277408	9ATSFc	Diabetic Retinopathy	Triacylglycerol (54:7)	IVW	4	-0.18338	0.058448	0.001704	-0.29794	-0.06882	0.832449	0.742344	0.933491	0.076104
GCST90277398	9ATSFc	Diabetic Retinopathy	Triacylglycerol (52:4)	IVW	7	-0.1187	0.040276	0.003207	-0.19764	-0.03976	0.888075	0.820665	0.961023	0.080681
GCST90277413	9ATSFc	Diabetic Retinopathy	Triacylglycerol (56:7)	IVW	5	-0.13072	0.045355	0.003951	-0.21961	-0.04182	0.877466	0.802829	0.959042	0.080681
GCST90277346	9ATSFc	Diabetic Retinopathy	Phosphatidylethanolamine (16:0_20:4)	IVW	5	-0.06963	0.024467	0.004426	-0.11759	-0.02168	0.932735	0.889062	0.978554	0.080681
GCST90277394	9ATSFc	Diabetic Retinopathy	Triacylglycerol (51:3)	IVW	6	-0.12531	0.044674	0.005032	-0.21287	-0.03775	0.882226	0.808263	0.962957	0.080681
GCST90277391	9ATSFc	Diabetic Retinopathy	Triacylglycerol (50:5)	IVW	3	-0.15715	0.056775	0.00564	-0.26843	-0.04588	0.854572	0.764576	0.95516	0.080681
GCST90277389	9ATSFc	Diabetic Retinopathy	Triacylglycerol (50:3)	IVW	3	-0.15086	0.05524	0.006313	-0.25914	-0.04259	0.859964	0.771719	0.958301	0.080681
GCST90277390	9ATSFc	Diabetic Retinopathy	Triacylglycerol (50:4)	IVW	3	-0.14756	0.054347	0.006623	-0.25408	-0.04104	0.862807	0.775627	0.959787	0.080681
GCST90277414	9ATSFc	Diabetic Retinopathy	Triacylglycerol (56:8)	IVW	5	-0.12723	0.049465	0.01011	-0.22418	-0.03027	0.880534	0.799172	0.970179	0.099488
GCST90277407	9ATSFc	Diabetic Retinopathy	Triacylglycerol (54:6)	IVW	3	-0.15911	0.062218	0.010547	-0.28106	-0.03717	0.852899	0.754982	0.963515	0.099488
GCST90277373	9ATSFc	Diabetic Retinopathy	Sphingomyelin (d36:2)	IVW	4	-0.11978	0.046944	0.010721	-0.21179	-0.02778	0.887111	0.809131	0.972607	0.099488
GCST90277261	9ATSFc	Diabetic Retinopathy	Diacylglycerol (18:1_18:1)	IVW	5	-0.1171	0.047962	0.014625	-0.21111	-0.02309	0.889495	0.809687	0.97717	0.120182

GCST90277397	9ATSFc	Diabetic Retinopathy	Triacylglycerol (52:3)	IVW	6	-0.09764	0.040475	0.015852	-0.17697	-0.01831	0.906977	0.837805	0.981859	0.120182
GCST90277399	9ATSFc	Diabetic Retinopathy	Triacylglycerol (52:5)	IVW	4	-0.11617	0.048289	0.016144	-0.21081	-0.02152	0.890326	0.809924	0.97871	0.120182
GCST90277396	9ATSFc	Diabetic Retinopathy	Triacylglycerol (52:2)	IVW	4	-0.11976	0.054773	0.028784	-0.22711	-0.0124	0.887135	0.796831	0.987674	0.173299
GCST90277317	9ATSFc	Diabetic Retinopathy	Phosphatidylcholine (18:2_20:4)	IVW	4	-0.12269	0.059336	0.038663	-0.23899	-0.00639	0.884536	0.787423	0.993626	0.173299
GCST90277247	9ATSFc	Diabetic Retinopathy	Sterol ester (27:1/18:3)	IVW	5	-0.09759	0.04722	0.038772	-0.19014	-0.00503	0.907025	0.826846	0.994979	0.173299
GCST90277360	9ATSFc	Diabetic Retinopathy	Phosphatidylinositol (16:0_20:4)	IVW	3	-0.13052	0.065525	0.046373	-0.25895	-0.0021	0.877635	0.771859	0.997907	0.173299
GCST90277349	9ATSFc	Diabetic Retinopathy	Phosphatidylethanolamine (18:1_18:1)	IVW	6	-0.07552	0.038314	0.048727	-0.15061	-0.00042	0.927265	0.860182	0.99958	0.173299
NWyUlf	bgsOMo	Diabetic Retinopathy	1-stearoyl-GPE (18:0)	IVW	7	-0.12726	0.038878	0.001063	-0.20346	-0.05106	0.880504	0.815901	0.950222	0.335891
qTq8oZ	bgsOMo	Diabetic Retinopathy	1-palmitoleoyl-GPC (16:1)	IVW	4	-0.19598	0.064934	0.002543	-0.32325	-0.06871	0.822027	0.723791	0.933596	0.406016
sz4Gjs	bgsOMo	Diabetic Retinopathy	5-hydroxylysine	IVW	4	0.10623	0.035353	0.002657	0.036939	0.175522	1.112078	1.03763	1.191868	0.406016
bovWvm	bgsOMo	Diabetic Retinopathy	2-stearoyl-GPE (18:0)	IVW	3	-0.20194	0.068532	0.003212	-0.33627	-0.06762	0.817142	0.714433	0.934617	0.406016
bAg65q	bgsOMo	Diabetic Retinopathy	X-24544	IVW	4	0.094578	0.033102	0.004275	0.029697	0.159458	1.099194	1.030142	1.172875	0.43959
JpLbOz	bgsOMo	Diabetic Retinopathy	X-26109	IVW	6	-0.06225	0.023021	0.006847	-0.10737	-0.01713	0.939644	0.898189	0.983013	0.43959
l48p4Z	bgsOMo	Diabetic Retinopathy	1-palmitoyl-2-docosahexaenoyl-GPE (16:0/22:6)	IVW	6	-0.07089	0.027592	0.010189	-0.12497	-0.01681	0.931562	0.882521	0.983328	0.43959
qvDQyh	bgsOMo	Diabetic Retinopathy	Oleoyl-linoleoyl-glycerol (18:1/18:2) [2]	IVW	7	-0.11049	0.04302	0.01022	-0.19481	-0.02617	0.895396	0.822992	0.974171	0.43959
O7rOPP	bgsOMo	Diabetic Retinopathy	1-stearoyl-2-docosahexaenoyl-GPE (18:0/22:6)	IVW	5	-0.08848	0.035956	0.013862	-0.15896	-0.01801	0.91532	0.853034	0.982153	0.43959

rv3dxu	bgsOMo	Diabetic Retinopathy	Metabolonic lactone sulfate	IVW	9	-0.05114	0.021622	0.018014	-0.09352	-0.00876	0.950142	0.910716	0.991274	0.450085
CCvMjz	bgsOMo	Diabetic Retinopathy	1-palmitoyl-GPE (16:0)	IVW	6	-0.10563	0.044739	0.018228	-0.19332	-0.01794	0.89976	0.824221	0.982222	0.450085
kiOFp8	bgsOMo	Diabetic Retinopathy	3-methylcytidine	IVW	4	0.064908	0.02756	0.018516	0.01089	0.118926	1.067061	1.010949	1.126287	0.450085
nWThM1	bgsOMo	Diabetic Retinopathy	1-stearoyl-gpc (18:0)	IVW	3	-0.16469	0.070509	0.019506	-0.30289	-0.02649	0.848157	0.738682	0.973857	0.456594
2gpdY5	bgsOMo	Diabetic Retinopathy	(2 or 3)-decenoate (10:1n7 or n8)	IVW	3	0.172741	0.075163	0.02155	0.025421	0.320061	1.188558	1.025747	1.377211	0.458414
MFZR2W	bgsOMo	Diabetic Retinopathy	Phosphate to oleoyl-linoleoyl-glycerol (18:1 to 18:2) [2]	IVW	6	0.106821	0.046667	0.022078	0.015354	0.198287	1.112735	1.015473	1.219313	0.458414
11AVUy	bgsOMo	Diabetic Retinopathy	(S)-a-amino-omega-caprolactam	IVW	3	-0.20266	0.088662	0.02227	-0.37643	-0.02888	0.816558	0.686304	0.971533	0.458414
8vdUya	bgsOMo	Diabetic Retinopathy	16a-hydroxy DHEA 3-sulfate	IVW	4	0.045599	0.021012	0.029992	0.004416	0.086782	1.046655	1.004426	1.090659	0.477316
XBLY8d	bgsOMo	Diabetic Retinopathy	N-stearoyl-sphingosine (d18:1 to 18:0) to N-palmitoyl-sphinganine (d18:0 to 16:0)	IVW	5	-0.11152	0.05141	0.030071	-0.21228	-0.01075	0.894476	0.808737	0.989305	0.477316
JeOlr0	bgsOMo	Diabetic Retinopathy	5alpha-androstan-3beta,17beta-diol disulfate	IVW	5	-0.0661	0.030967	0.032795	-0.1268	-0.00541	0.936036	0.880913	0.994608	0.477316
uGLKq3	bgsOMo	Diabetic Retinopathy	Sphingomyelin (d18:1/20:1, d18:2/20:0)	IVW	4	-0.09676	0.045423	0.03315	-0.18579	-0.00773	0.907771	0.830446	0.992296	0.477316
WlmWdG	bgsOMo	Diabetic Retinopathy	1-(1-enyl-palmitoyl)-2-palmitoyl-GPC (P-16:0/16:0)	IVW	7	-0.06985	0.03285	0.033487	-0.13423	-0.00546	0.932538	0.874388	0.994555	0.477316
VCfnXS	bgsOMo	Diabetic Retinopathy	Glyco-beta-muricholate	IVW	3	0.052362	0.024733	0.03425	0.003886	0.100838	1.053757	1.003893	1.106097	0.477316
BSdLQQ	bgsOMo	Diabetic Retinopathy	1-palmitoyl-2-oleoyl-GPI (16:0/18:1)	IVW	4	0.104961	0.04983	0.035172	0.007294	0.202628	1.110667	1.00732	1.224617	0.477316
iyY6VS	bgsOMo	Diabetic Retinopathy	Sphingomyelin (d18:1/18:1, d18:2/18:0)	IVW	4	-0.11215	0.053304	0.035387	-0.21662	-0.00767	0.893914	0.805235	0.992359	0.477316
2Iat3i	bgsOMo	Diabetic Retinopathy	Homocitrulline	IVW	4	-0.08408	0.040032	0.035691	-0.16255	-0.00562	0.919353	0.849975	0.994394	0.477316

7nPRpG	bgsOMo	Diabetic Retinopathy	5alpha-androstan-3alpha,17alpha-diol monosulfate	IVW	6	-0.04861	0.023256	0.0366	-0.09419	-0.00303	0.952552	0.910107	0.996977	0.477316
vblnRy	bgsOMo	Diabetic Retinopathy	Epiandrosterone sulfate	IVW	5	-0.03537	0.016936	0.036736	-0.06857	-0.00218	0.965244	0.933728	0.997823	0.477316
3qxNmb	bgsOMo	Diabetic Retinopathy	Indoleacetylglutamine	IVW	4	-0.07153	0.034295	0.037007	-0.13874	-0.00431	0.930971	0.87045	0.995699	0.477316
6XVwdY	bgsOMo	Diabetic Retinopathy	1-palmitoyl-2-linoleoyl-GPI (16:0/18:2)	IVW	5	0.089244	0.043474	0.040088	0.004036	0.174452	1.093348	1.004044	1.190594	0.478121
mOnDnC	bgsOMo	Diabetic Retinopathy	Ceramide (d18:1/24:1)	IVW	6	0.076792	0.037543	0.040813	0.003207	0.150377	1.079818	1.003212	1.162272	0.478121
mTmMwi	bgsOMo	Diabetic Retinopathy	Dodecadienoate (12:2)	IVW	5	0.106767	0.053026	0.044064	0.002836	0.210697	1.112674	1.00284	1.234538	0.488564
ebi-a-GCST90001531	WlsRdR	Diabetic Neuropathy	CD33dim HLA DR- AC Myeloid cell	IVW	3	-0.04941	0.020557	0.016234	-0.0897	-0.00912	0.951791	0.914204	0.990922	0.591424
ebi-a-GCST90001532	WlsRdR	Diabetic Neuropathy	Basophil AC Myeloid cell	IVW	3	-0.04855	0.020568	0.018247	-0.08887	-0.00824	0.952607	0.914968	0.991795	0.591424
kSb5G1	rYBKu8	Diabetic Neuropathy	Species UBA1375 sp002305795	IVW	8	0.670921	0.211216	0.001491	0.256938	1.084904	1.956038	1.292965	2.959154	0.705138
KYXv9z	rYBKu8	Diabetic Neuropathy	Species Flavonifractor sp002159265	IVW	9	-0.65395	0.225655	0.003755	-1.09624	-0.21167	0.519987	0.334127	0.809233	0.888132
1pcbtU	rYBKu8	Diabetic Neuropathy	Phylum Flavobacteriales	IVW	14	-0.84856	0.31158	0.006461	-1.45926	-0.23786	0.428031	0.232409	0.788312	0.965903
teZAMJ	rYBKu8	Diabetic Neuropathy	Species Blautia sp000436935	IVW	14	0.236262	0.090138	0.008764	0.059592	0.412933	1.266507	1.061403	1.511244	0.965903
ONqPAv	rYBKu8	Diabetic Neuropathy	Species UBA1448	IVW	4	0.921921	0.369174	0.012516	0.198339	1.645503	2.514114	1.219375	5.183614	0.965903
SisCXl	rYBKu8	Diabetic Neuropathy	Species CAG-273 sp003534295	IVW	7	0.246272	0.099466	0.013288	0.051319	0.441225	1.279248	1.052659	1.55461	0.965903
yk68Jl	rYBKu8	Diabetic Neuropathy	Species Bacteroides A plebeius	IVW	9	0.212565	0.086769	0.014295	0.042497	0.382633	1.236847	1.043413	1.466139	0.965903
1jYqJ0	rYBKu8	Diabetic Neuropathy	Species GCA-900066135 sp900066135	IVW	7	-0.6476	0.280102	0.020777	-1.1966	-0.0986	0.523299	0.302219	0.906103	0.996485
1BHknX	rYBKu8	Diabetic Neuropathy	Genus Enterococcus A	IVW	12	0.552828	0.262581	0.03526	0.038169	1.067487	1.738161	1.038907	2.908061	0.996485

MZ3oef	rYBKu8	Diabetic Neuropathy	Genus Succinivibrio	IVW	21	-0.2173	0.104976	0.038457	-0.42305	-0.01154	0.804692	0.655046	0.988524	0.996485
aSPBdS	rYBKu8	Diabetic Neuropathy	Class Clostridia	IVW	6	0.895035	0.437482	0.040768	0.03757	1.7525	2.447421	1.038284	5.769008	0.996485
LKakmo	rYBKu8	Diabetic Neuropathy	Unclassified UBA8517	IVW	9	-0.64717	0.330072	0.049914	-1.29411	-0.00023	0.523524	0.274141	0.999768	0.996485
GCST90274842	Ef6GPW	Diabetic Neuropathy	TNFSF14	IVW	4	-0.20384	0.086222	0.018073	-0.37283	-0.03484	0.815594	0.688779	0.965757	0.574746
GCST90274820	Ef6GPW	Diabetic Neuropathy	LIFR	IVW	3	0.197037	0.083936	0.018901	0.032523	0.36155	1.217789	1.033057	1.435553	0.574746
GCST90274765	Ef6GPW	Diabetic Neuropathy	CCL19	IVW	3	-0.14517	0.069411	0.036492	-0.28121	-0.00912	0.864879	0.754869	0.990921	0.574746
bBTd9n	PO7ZNY	Diabetic Neuropathy	Phosphate to serine	IVW	3	0.3444	0.100288	0.000594	0.147836	0.540964	1.411143	1.159322	1.717662	0.147179
67ZWOs	PO7ZNY	Diabetic Neuropathy	Serine	IVW	3	-0.29925	0.093971	0.00145	-0.48343	-0.11507	0.741373	0.616662	0.891306	0.229103
8T7iMz	PO7ZNY	Diabetic Neuropathy	3-hydroxyhexanoylcarnitine (1)	IVW	3	-0.35505	0.121412	0.003452	-0.59302	-0.11709	0.701136	0.552656	0.889509	0.436279
xdxcHi	PO7ZNY	Diabetic Neuropathy	X-24556	IVW	3	-0.24091	0.08608	0.005132	-0.40962	-0.07219	0.785915	0.6639	0.930355	0.540579
uVrSDL	PO7ZNY	Diabetic Neuropathy	X-24328	IVW	7	-0.25237	0.101098	0.012551	-0.45052	-0.05421	0.77696	0.637297	0.947229	0.710531
s4ctla	PO7ZNY	Diabetic Neuropathy	3-hydroxybutyroylglycine	IVW	3	-0.1598	0.067847	0.018512	-0.29278	-0.02681	0.852318	0.746189	0.973542	0.710531
JcPwzk	PO7ZNY	Diabetic Neuropathy	N-acetylputrescine to (N(1) + N(8))-acetylspermidine	IVW	5	0.105885	0.045198	0.019145	0.017297	0.194474	1.111694	1.017447	1.214672	0.710531
Ub4VMX	PO7ZNY	Diabetic Neuropathy	2-butenoylglycine	IVW	3	-0.18064	0.077724	0.020117	-0.33298	-0.0283	0.834733	0.716783	0.972092	0.710531
aa5Vyh	PO7ZNY	Diabetic Neuropathy	1-oleoyl-2-docosahexaenoyl-GPC (18:1/22:6)	IVW	5	-0.30944	0.133369	0.020329	-0.57085	-0.04804	0.733854	0.565046	0.953094	0.710531
6m7Q6j	PO7ZNY	Diabetic Neuropathy	X-26054	IVW	4	0.120839	0.053634	0.024256	0.015717	0.225961	1.128444	1.015842	1.253527	0.712564
xIK9X5	PO7ZNY	Diabetic Neuropathy	Indolelactate	IVW	3	-0.24999	0.113157	0.027161	-0.47178	-0.0282	0.77881	0.623893	0.972195	0.712564

GKHDzV	PO7ZNY	Diabetic Neuropathy	Paraxanthine to 5-acetylamino-6-formylamino-3-methyluracil	IVW	4	-0.07472	0.034901	0.032281	-0.14313	-0.00631	0.928002	0.866643	0.993706	0.712564
TI3QmT	PO7ZNY	Diabetic Neuropathy	Argininate	IVW	5	0.19039	0.089356	0.033114	0.015252	0.365527	1.209721	1.015369	1.441274	0.712564
6GevTF	PO7ZNY	Diabetic Neuropathy	Orotate	IVW	4	0.088407	0.042549	0.037729	0.005012	0.171803	1.092433	1.005024	1.187444	0.769178
8Fsdwg	PO7ZNY	Diabetic Neuropathy	X-25433	IVW	3	-0.13364	0.065982	0.042825	-0.26296	-0.00432	0.874905	0.768769	0.995693	0.820164
h3VOoN	PO7ZNY	Diabetic Neuropathy	Carnitine	IVW	4	-0.12595	0.062799	0.044905	-0.24903	-0.00286	0.881662	0.779554	0.997144	0.834712
ukb-b-16202	DqkEkJ	Diabetic Neuropathy	Carotene	IVW	15	0.489069	0.22305	0.028333	0.051891	0.926247	1.630798	1.053261	2.525015	0.392796
